# Enantioselective Access to Decahydroquinolines Bearing a C_4a_ or C_8a_ Quaternary Stereocenter from a Common Intermediate Total Synthesis of (−)-Myrioxazine A

**DOI:** 10.1021/acs.joc.5c00321

**Published:** 2025-04-11

**Authors:** Arnau Calbó, Miriam Piccichè, Llorenç Rubert, Eisuke Comas-Iwasita, Rosa Griera, Carolina Estarellas, F. Javier Luque, Joan Bosch, Mercedes Amat

**Affiliations:** † Laboratory of Organic Chemistry, Faculty of Pharmacy and Food Sciences, and Institute of Biomedicine (IBUB), 16724 University of Barcelona, 08028 Barcelona, Spain; ‡ Departament de Nutrició, Cieǹcies de l′Alimentació i Gastronomia, Facultat de Farmàcia i Ciències de l′Alimentació, Institut de Biomedicina (IBUB) and Institut de Química Teòrica i Computacional (IQTCUB), 08921 Santa Coloma de Gramenet, Spain

## Abstract

(*R*)-Phenylglycinol-derived perhydrooxazoloquinoline **2** provides stereoselective access to angularly substituted enantiopure decahydroquinolines (DHQs). Reaction of **2** with appropriate Grignard reagents leads to *cis*-DHQs bearing a C_8a_ aza-quaternary stereocenter, whereas reaction of **2** with Michael acceptors followed by reductive removal of the 2-phenylethanol of the chiral inductor gives rise to *cis*- or *trans*-DHQs bearing a C4a all-carbon quaternary stereocenter depending on the hydride used for the cleavage of the oxazolidine C–O bond. Theoretical studies have clarified the mechanistic intricacies of the reaction of **2** with Michael acceptors, providing arguments for a proper understanding of the observed stereoselectivity. Finally, the reaction of **2** with formaldehyde is reversible, allowing the regioselective formation of either the angularly substituted hydroxymethyl derivative **12** or the C_8_-substituted derivative **13** depending on the reaction temperature. An expeditious synthesis of the Myrioneuron-type alkaloid (−)-myrioxazine A from **13** is reported.

## Graphical abstract

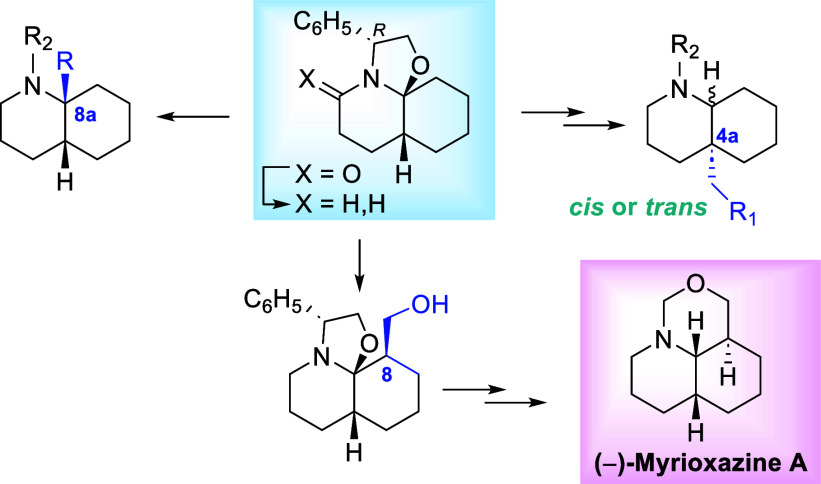

## Introduction

Organic compounds containing quaternary stereocenters can be found in numerous natural products and synthetic drugs. As a consequence of the enhanced selectivity and metabolic stability conferred by the rigidity of their three-dimensional structures, they are often endowed with interesting biological activities.^1^ However, the stereocontrolled generation of quaternary centers of well-defined configuration is a challenging issue in organic synthesis, particularly in the case of all-carbon quaternary centers due to the steric congestion imposed by four different carbon substituents.^2^

A number of natural products containing a decahydroquinoline (DHQ) nucleus with a quaternary stereocenter at the C_4a_ or C_8a_ positions have been isolated from different natural sources. For instance, cylindricine- and lepadiformine-type marine alkaloids,^3^ such as (−)-cylindricine H, and the Lycopodium alkaloid (+)-lycopodine display an aza-quaternary stereocenter in their polycyclic structures. On the other, hand, (−)-lannotinidine B, also belonging to the Lycopodium class^4^ of natural products, and some Myrioneuron alkaloids,^5^ such as (+)-myrioneurinol, have an all-carbon quaternary stereocenter at the C_4a_ of the DHQ framework. This structural feature can also be found in most monoterpene indole alkaloids of the Aspidosperma group,^6^ such as (+)-limaspermine and (+)-kopsihainanine A (Figure 1).

**Figure 1:**
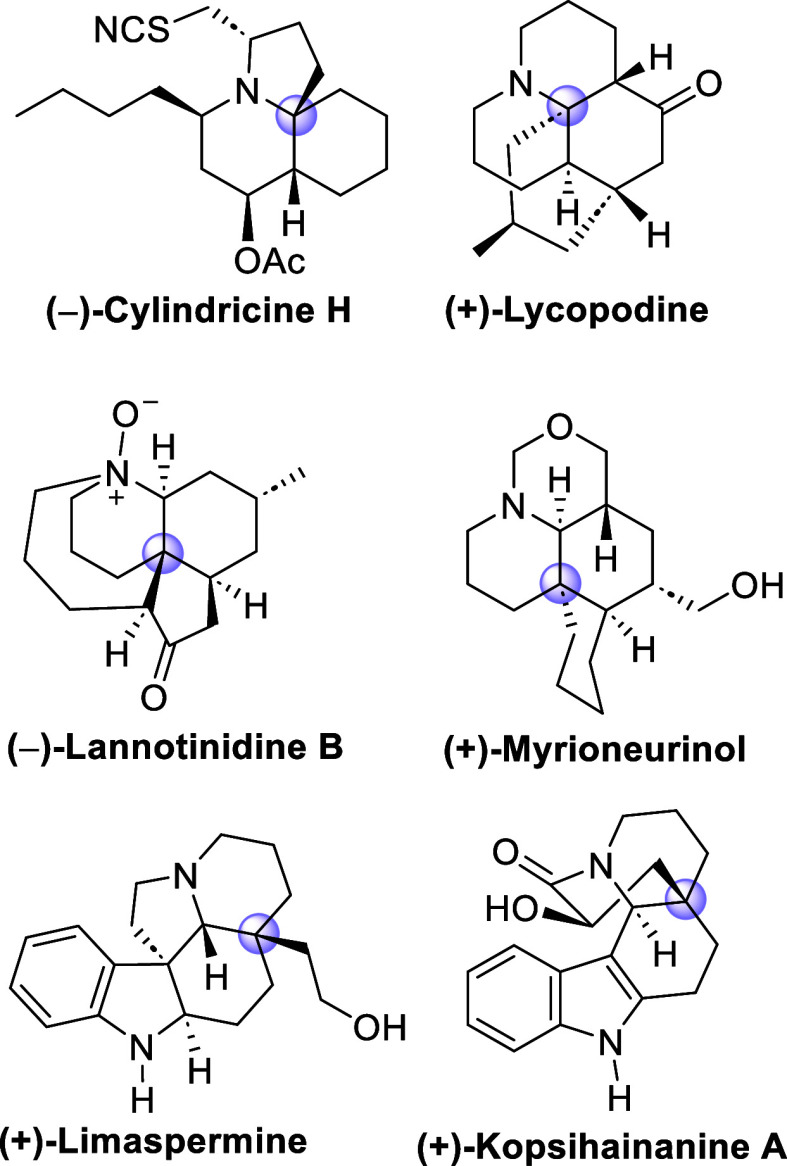
Alkaloids bearing a quaternary stereocenter on the decahydroquinoline nucleus.

In previous work we have demonstrated the synthetic utility of phenylglycinol-derived tricyclic lactams as enantiopure platforms for the total synthesis of DHQ alkaloids. This was accomplished from a simple unsubstituted lactam **1** by sequential introduction of substituents on the piperidine ring, taking advantage of the lactam functionality,^7^ or from lactams already substituted on the carbocyclic ring, prepared by a stereoselective cyclocondensation reaction from racemic or achiral cyclohexanone-2-propionate derivatives.^8^ However, all attempts to introduce allyl substituents at the C_8a_ position (DHQ numbering) under Hosomi–Sakurai conditions^9^ from lactam **1** were unsuccessful,^7^ probably due to the attenuated reactivity of the hemiaminal function associated with the amide-like nature of the nitrogen atom.

For this reason, we decided to explore the behavior of the reduced analogue **2**, which can be considered a latent form of an iminium salt **A** and could therefore allow the generation of aza-quaternary stereocenters at the C_8a_ position of the DHQ nucleus following treatment with appropriate nucleophiles (Scheme 1). On the other hand, compound **2** can also be regarded as a latent form of two regioisomeric enamines (**B** and **C**) generated from the above iminium salt by loss of a proton either from C_4a_ or C_8_. We expected that treatment of **2** with electrophiles, such as Michael acceptors, aldehydes or alkylating reagents, would enable the introduction of substituents at these positions, thus generating precursors of DHQs either with an all-carbon quaternary stereocenter at C_4a_ or substituted at C_8_. In this context, the main goal of this work is to analyze the reactivity of perhydrooxazoloquinoline **2** with the final purpose of establishing procedures for the stereoselective preparation of enantiopure decahydroquinolines with a quaternary stereocenter.

**Scheme 1:**
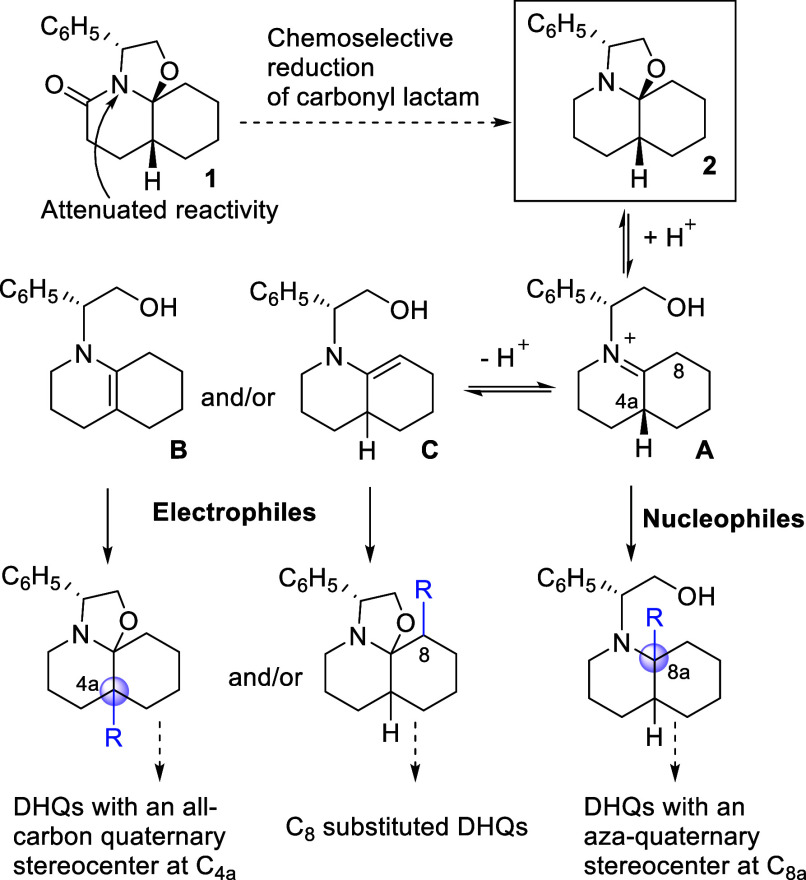
Synthetic Potential of Perhydrooxazoloquinoline **2**

## Results and Discusion

Compound **2** was readily obtained in 84% overall yield^10^ from tricyclic lactam **1** by its conversion into the corresponding thiolactam followed by treatment with NaBH_4_ (Scheme 2). As expected, the generation of an aza-quaternary stereocenter at the C_8a_ position of the DHQ nucleus was satisfactorily accomplished by addition of allylmagnesium bromide or ethynylmagnesium bromide to a THF solution of compound **2** at −78 °C followed by stirring for 18 h at room temperature. The respective *cis*-DHQs **3** and **4** were isolated in good yield and excellent stereoselectivity. However, treatment of **2** with vinylmagnesium bromide required additional heating at reflux temperature to give compound **5** in 43% yield. All attempts to introduce alkyl substituents at the C_8a_ position by reaction of **2** with alkyl Grignard reagents were unsuccessful, probably due to the higher steric requirements of alkyl groups compared to their unsaturated analogues.

**Scheme 2:**
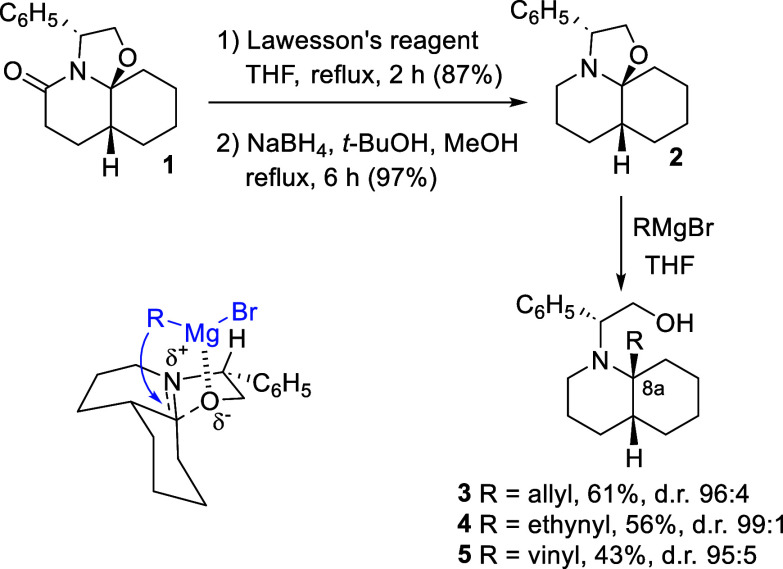
Stereoselective Generation of 8a-Substituted DHQs

The stereochemical outcome of the above reactions could be rationalized by considering that the Grignard reagent generates an incipient iminium ion by coordination with the oxygen atom of the oxazolidine ring, thus promoting the intramolecular attack of the nucleophile from the same face of the C–O bond.

The unsaturated substituents present in **3**–**5** could be further elaborated into a variety of functionalities, thus making these compounds versatile precursors of DHQ derivatives containing a C_8a_ aza-quaternary stereocenter. In fact, the allyl derivative **3** is a known synthetic precursor of the marine DHQ alkaloid (−)-cylindricine H^7^ (See Figure 1).

Next, we decided to explore the scope and limitations of oxazolodecahydroquinoline **2** as a platform for the preparation of C_4a_- or C_8_-substituted DHQs. The reaction of a simple (*R*)-phenylglycinol-derived oxazolopiperidine with methyl vinyl ketone (MVK) has been reported by Husson et al. to give the corresponding addition product in good yield and excellent stereoselectivity^11^ (Scheme 3). The stereochemical outcome observed in the attack of the intermediate enamine to the enone was rationalized in terms of stereoelectronic and steric effects.^11a^ However, the isomerization of the stereocenters through the corresponding enamine to give the thermodynamically more stable stereoisomer cannot be ruled out.

**Scheme 3:**
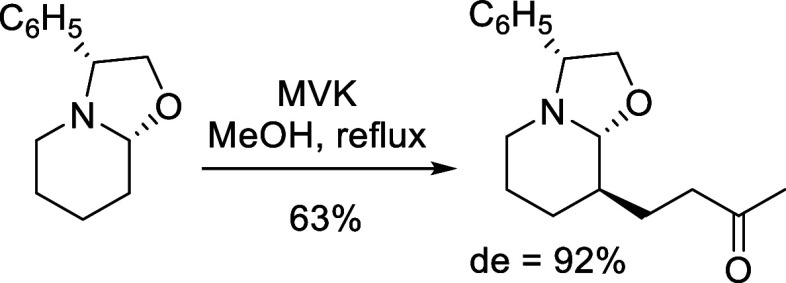
Reaction of a Phenylglycinol-Derived Oxazolopiperidine with Methyl Vinyl Ketone

These results prompted us to use this Michael acceptor in our first assays. When a solution of perhydrooxazoloquinoline **2** in MeOH was heated at reflux temperature with an excess of MVK (**6**), the addition compound **9**, resulting from the attack at the DHQ C_4a_ position, was isolated as the only regio- and stereoisomer detectable by spectroscopic methods (Table 1, entry 1). A similar result was obtained when using dioxane as the solvent (entry 2), whereas in refluxing toluene compound **9** was obtained in a lower yield (entry 3). Similarly, in the reaction of **2** with phenyl vinyl ketone (**7**) to give the C_4a_-substituted DHQ **10** the best results were observed when using MeOH as the solvent (entries 4–6). Encouraged by these results, we decided to explore alternative Michael acceptors. However, when compound **2** was treated with phenyl vinyl sulfone (**8**) under the above optimized conditions, the corresponding C_4a_ alkylated compound **11** was isolated in only a modest 27% yield (entry 7) due to a competitive addition of MeOH to sulfone **8**. Changing the solvent to dioxane provided the desired product **11** in 51% yield (entry 8), whereas in toluene a moderate 45% yield was obtained (entry 9).

**Table 1:**
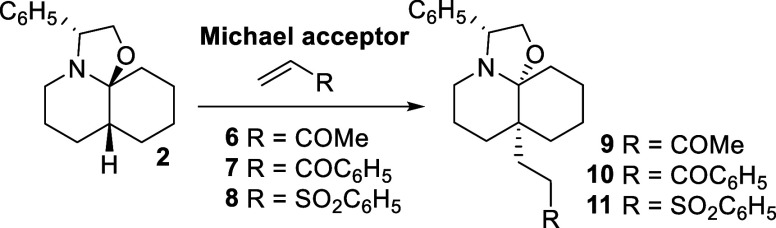
Reaction of Perhydrooxazoloquinoline **2** with Michael Acceptors

entry	reagent^a^	solvent	*T* (h)	product	yield (%)
1	**6**	MeOH	24	**9**	69
2	**6**	dioxane	24	**9**	65
3	**6**	toluene	24	**9**	44
4	**7**	MeOH	24	**10**	76
5	**7**	dioxane	24	**10**	57
6	**7**	toluene	24	**10**	48
7	**8**	MeOH	48	**11**	27
8	**8**	dioxane	48	**11**	51
9	**8**	toluene	48	**11**	45

a All reactions were carried out using 5 equiv of the reagent at reflux temperature.

All attempts to induce the reaction using other Michael acceptors such as acrylonitrile or methyl acrylate resulted in failure. Nor was the expected product obtained when using the alkylating reagent methyl bromoacetate. Surprisingly, however, refluxing a methanolic solution of perhydrooxazoloquinoline **2** with formalin (CH_2_O 37% w/w in H_2_O solution) for 24 h afforded a mixture of the expected C_4a_-substituted DHQ **12** along with its C_8_ regioisomer **13** in 51% yield (17:83 ratio) as the only stereoisomers detectable by spectroscopic methods (Scheme 4). Interestingly, when the reaction was performed at room temperature, the angularly substituted compound **12** was regio- and stereoselectively isolated in 73% yield. The absolute configuration of the stereocenters present in **13** was unambiguously determined by X-ray crystallographic analysis.

**Scheme 4:**
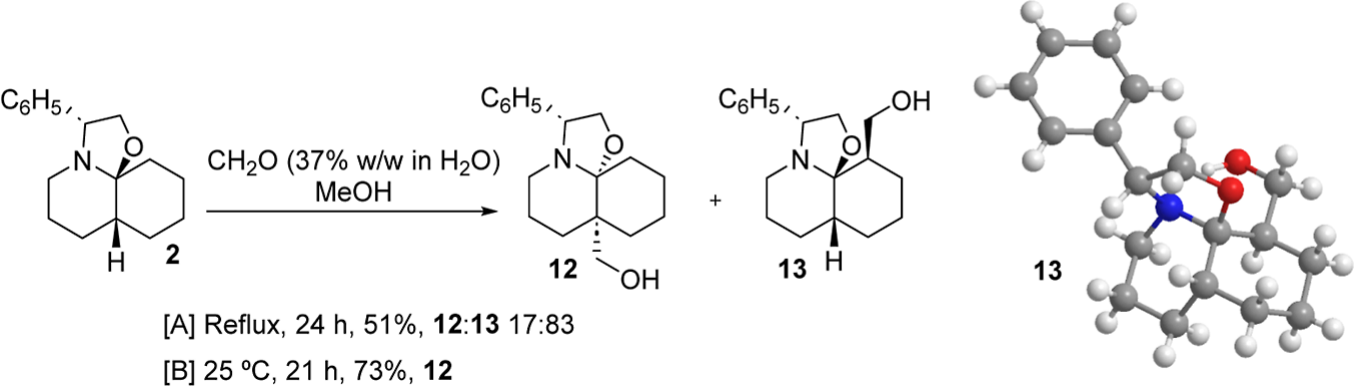
Reaction of Perhydrooxazoloquinoline **2** with Formaldehyde

The above results can be rationalized by considering that the reaction of **2** with formaldehyde is reversible and that **12** is the product formed under kinetically controlled conditions. To prove the reversibility of the reaction, we heated at reflux temperature a solution of **12** in toluene in the absence of formalin. After 16 h we observed that **12** had lost the hydroxymethyl appendage and that the solution only contained the unsubstituted compound **2**. Also in accordance with the reversibility of the process, refluxing a methanolic solution of **12** containing 5 equiv of formaldehyde for 16 h provided an equimolecular mixture of both regioisomers **12** and **13**.

Notably, the highly stereoselective formation of compounds **9**–**12** from **2** involves a 1,4-asymmetric induction from a single stereocenter located in a conformationally flexible position: an all-carbon quaternary stereocenter is generated by a Michael addition of an enamine intermediate whose only stereocenter is in an exocyclic appendage of the DHQ ring with a free conformational rotation around the C–N bond.

On the basis of these findings, density functional theory calculations at the M062X/6-31G­(d,p) level^12^ in the gas phase and in solution (dioxane and methanol)^13^ were performed to examine the reaction mechanism of the above transformations. First, the conformational stability of the regioisomeric enamine **B** was analyzed, taking into consideration different chair conformations of the DHQ ring (**B**_**1**_–**B**_**4**_, Figure 2A). For each chair conformation (**B**_**X**_), we examined the conformational preferences of the exocyclic N-substituted group (Figure 2B) and determined the energetic stability of the distinct conformational states. The results indicated that the most stable conformers obtained for the four distinct chair conformations of the DHQ ring differ by 2 kcal/mol, although conformations **B**_**1**_, **B**_**2**_ and **B**_**3**_ exhibit a similar stability (energy difference less than 0.8 kcal/mol; Figure 2C). Notably, the arrangement of the exocyclic N-substituted group is similar in all cases. Thus, the hydroxy group is pointing toward the *re* face of the DHQ core, whereas the phenyl ring is oriented toward the *si* face. We hypothesize that this structural feature could be critical for the stereoselective formation of the adduct due to the steric hindrance of the incoming reactant with the phenyl ring and the hydrogen atoms in axial position of the DHQ nucleus, which in turn would favor the addition through the *re* face.

**Figure 2:**
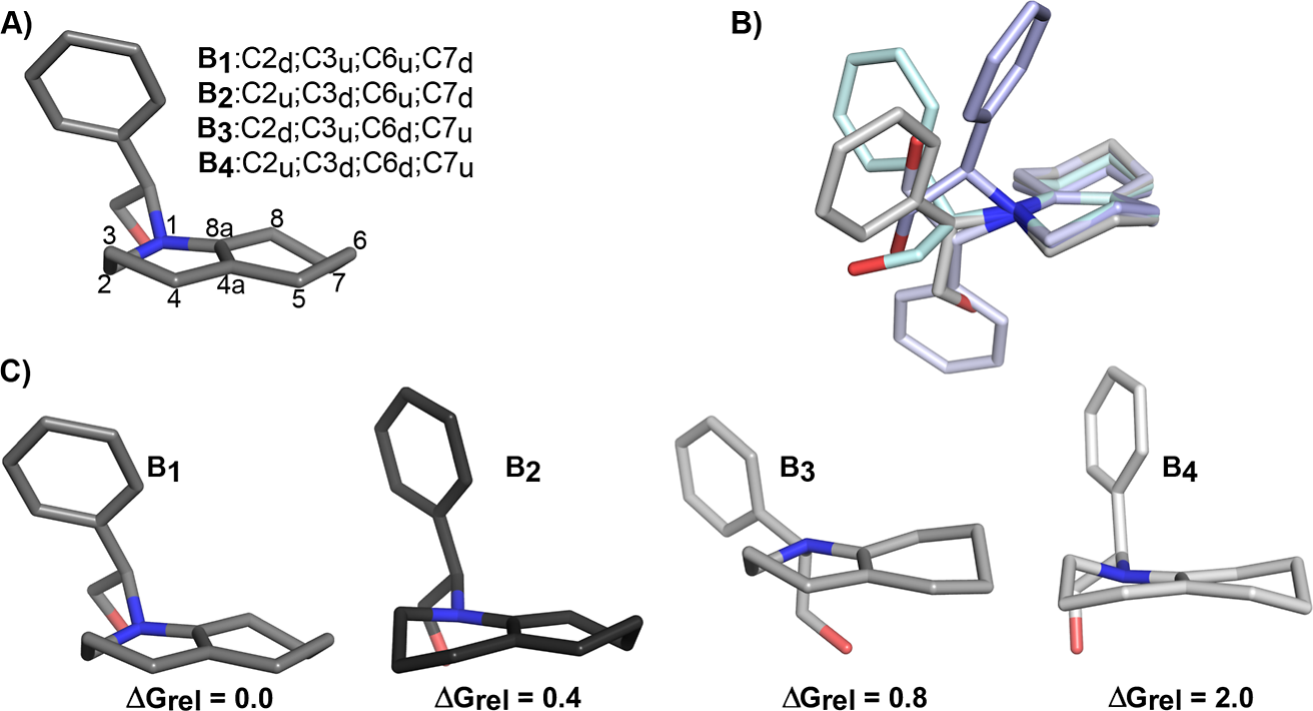
(A) Molecular geometry of enamine **B**. (B) Different conformations explored for species **B**_**1**_. (C) Representation of the four most stable conformations obtained from M062X calculations determined for species **B**_**1**_–**B**_**4**_. Relative stabilities (ΔGrel) given in kcal/mol. For the sake of clarity, hydrogen atoms are not displayed.

To examine the reaction mechanism, MVK was selected as the Michael acceptor for the addition on the *re* face of enamine **B** (Figure 3). Indeed, this reaction pathway leads to a reactant complex between **B** and MVK, where the hydroxy group forms a hydrogen bond interaction (distance H···O­(=C) of 1.9 Å) with the carbonyl group of MVK. This facilitates the adoption of an arrangement suitable for the formation of the bond between the C_4a_ atom of **B** and the vinyl moiety of MVK.

**Figure 3:**
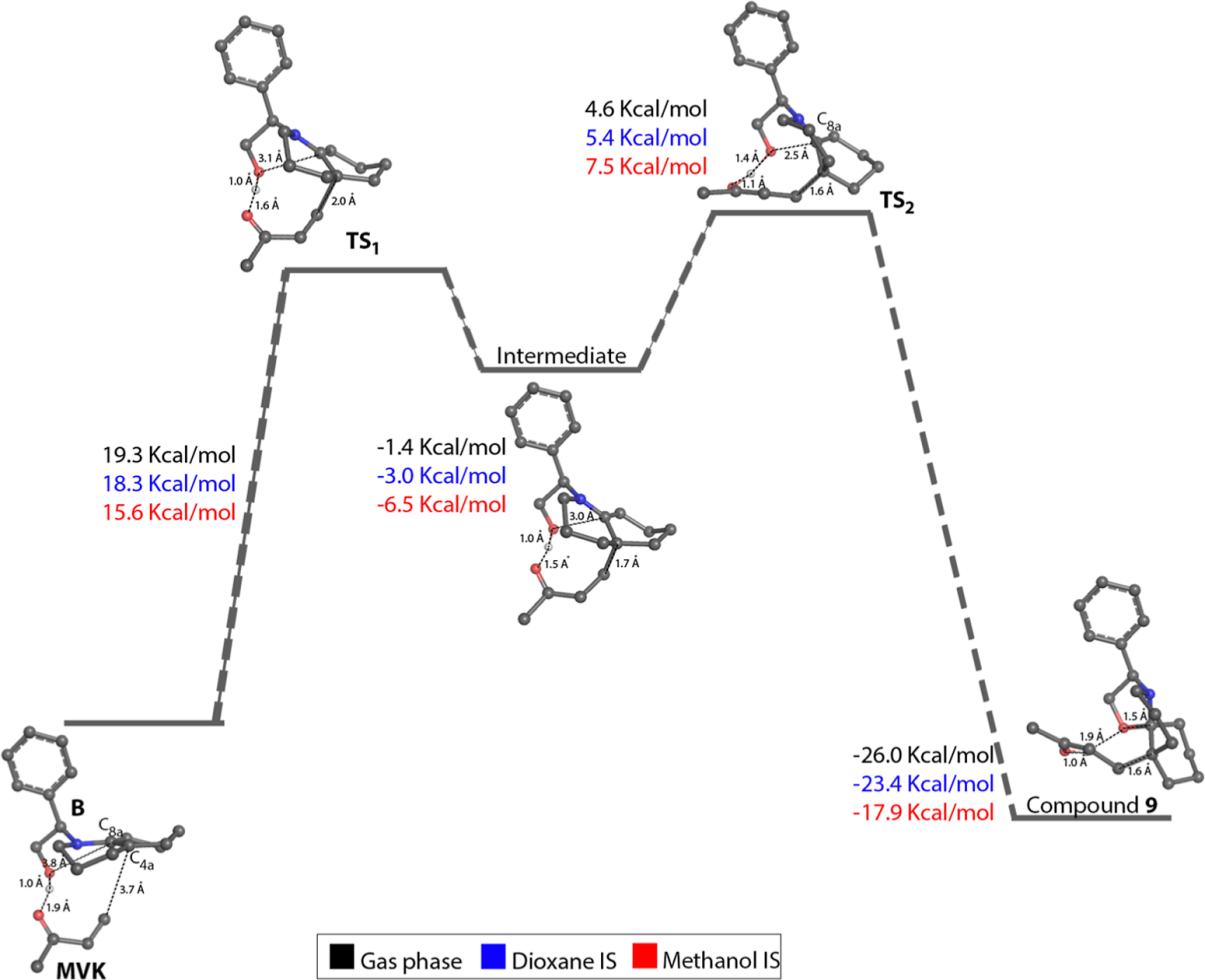
Mechanistic pathway for the 1,4-addition of enamine **B** and methyl vinyl ketone leading to the formation of product **9**. Values (kcal/mol) determined in the gas phase, dioxane and methanol.

The first transition state (TS_1_) involves the bond formation between C_4a_ of the DHQ ring and the Michael acceptor. The distance between the C_4a_ and vinyl atoms is reduced from 3.7 Å in the reactant state to 2.0 Å in TS_1_. Additionally, the distances between the hydroxy hydrogen of **B** and the carbonyl oxygen of MVK is shortened from 1.9 to 1.6 Å, whereas the distance between the hydroxy oxygen and C_8a_ is reduced from 3.9 to 3.1 Å. The intermediate generated from TS_1_ features an arrangement of the interaction (**B**)­O···H···O^–^(MVK) that enables the transfer of the proton from the hydroxy group to the carbonyl oxygen to generate the enol species of **B** in the second transition state (TS_2_). The proton transfer in TS_2_ also reduces the distance from the oxygen atom to C_8a_, thus facilitating the formation of the oxazolo ring in the final product **9** (Figure 3).

From an energetic point of view, the limiting step is the approximation of both reactants to generate TS_1_, which would require 19.3 kcal/mol in the gas phase. Additionally, the connection between the reactant, intermediate and product corresponding to both TS_1_ and TS_2_ was confirmed upon inspection of the energy profiles determined from intrinsic reaction coordinate (IRC) calculations (Figure S3), which yielded similar structural geometries and energetic values to those reported in the mechanistic pathway shown in Figure 3. The energetic stabilization of TS_1_ obtained upon addition of the solvation effect in both dioxane (18.3 kcal/mol) and methanol (15.6 kcal/mol) indicates that the reaction would be favored relative to the gas phase, in agreement with the experimental findings.

Overall, this reaction mechanism provides a basis to explain the high stereoselectivity of the products formed as well as to justify why Michael acceptors like acrylonitrile are not successful in the reaction process.

The access to C_4a_-substituted *cis*- or *trans*-DHQs from compounds **9**–**12** requires the stereoselective removal of the phenylethanol moiety of the chiral inductor. This transformation was performed by reductive cleavage of the C–O bond of the oxazolidine ring, which generates the stereocenter at the C_8a_ position, and a subsequent debenzylation.

In our initial assays we used NaBH_3_CN and NaBH­(OAc)_3_ to avoid the undesired reduction of the ketone function present in **9**. Addition of NaBH_3_CN to a CH_2_Cl_2_ solution of **9** containing 0.5 equiv of TFA afforded DHQ *trans*-**14b** in moderate yield and stereoselectivity (Table 2, entry 1). Using similar reaction conditions, sulfone **11** provided an almost equimolecular mixture of the isomers *cis*-**15a** and *trans*-**15b** (entry 2). Alternatively, the reduction of compounds **9** and **11** with NaBH­(OAc)_3_ in the presence of TFA gave the corresponding isomers *cis*-**14a** and *cis*-**15a** as the major products (entries 3 and 4). With the aim of improving the stereoselectivity of this transformation, we explored alternative reaction conditions. However, only a moderate stereoselectivity was observed when using NaBH_4_: compounds **9** and **10** provided alcohols *trans*-**16b** and *trans*-**17b**, respectively, as the major products (entries 5 and 6), arising from the selective *trans* reductive opening of the oxazolidine ring and the reduction of the ketone in the C_4a_ substituent. A similar *trans* stereoselectivity was observed from sulfone **11** and the hydroxymethyl substituted DHQ **12** (entries 7 and 8). Finally, the best stereochemical results were achieved when using DIBAL as the reducing reagent: stirring a THF solution of compounds **9**–**12** at room temperature afforded in good to moderate yields the respective DHQs *cis*-**15a**–*cis*-**18a** as the only isomers detectable by spectroscopic methods (entries 9–12).

**Table 2:**
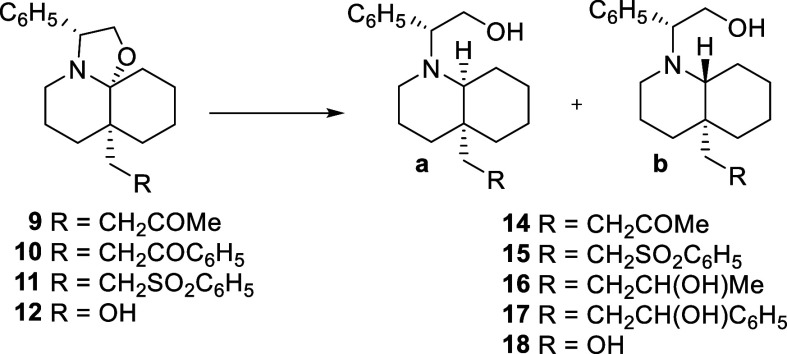
Reductive Opening of the Oxazolidine Ring

entry	substrate	hydride	solvent	*T* (h)	product	yield (%)	ratio a/b
1	**9**	NaBH_3_CN^a^	CH_2_Cl_2_	4	**14**	69	30:70
2	**11**	NaBH_3_CN^a^	CH_2_Cl_2_	72	**15**	74	46:54
3	**9**	NaBH(OAc)_3_ ^a^	CH_2_Cl_2_	4	**14**	77	81:19
4	**11**	NaBH(OAc)_3_ ^a^	CH_2_Cl_2_	72	**15**	56	84:16
5	**9**	NaBH_4_	MeOH	1	**16**	83	24:76
6	**10**	NaBH_4_	MeOH	24	**17**	62	30:70
7	**11**	NaBH_4_	MeOH	72	**15**	54	20:80
8	**12**	NaBH_4_	MeOH	4	**18**	45	36:64
9	**9**	DIBAL	THF	4	**16**	60	99:1
10	**10**	DIBAL	THF	4	**17**	63	99:1
11	**11**	DIBAL	THF	4	**15**	88	99:1
12	**12**	DIBAL	THF	2	**18**	54	99:1

a The reactions were carried out at 25 °C in the presence of 0.5 equiv of TFA.

The absolute configuration of the C_4a_ and C_8a_ stereocenters of sulfone *cis*-**15a** was unambiguously determined by X-ray crystallography (Figure 4). This result also confirmed that the attack of phenyl vinyl sulfone (**8**) to the nucleophilic carbon of the intermediate enamine **B** resulting from the opening of the oxazolidine ring of **2** (see Table 1) took place stereoselectively by the *re* face, affording product **11** with an *S* configuration at the quaternary stereocenter. The synthetic versatility of the sulfone group renders compounds *cis*-**15a** and *trans*-**15b** valuable precursors of *cis*- and *trans*-DHQs bearing diversely functionalized C_4a_ substituents.^14^

**Figure 4:**
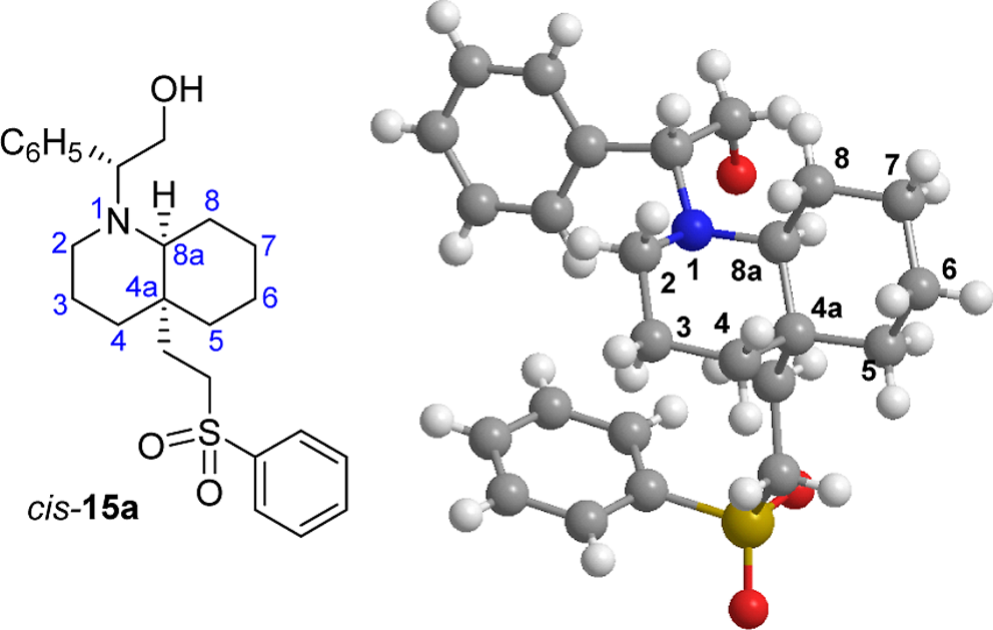
X-ray crystal structure of sulfone *cis*-**15a**.

The differences in the stereoselectivity shown in Table 2 could be rationalized as follows. The reactions with NaBH_3_CN, NaBH_4_, and NaBH­(OAc)_3_ take place through a presumed iminium ion intermediate resulting from cleavage of the oxazolidine C–O bond. This bicyclic iminium cation would adopt a conformation with the C_4a_ substituent axial, and the reduction with NaBH_3_CN or NaBH_4_ would take place mainly under stereoelectronic control,^15^ with axial delivery of the hydride on the (less hindered) *si* face of the electrophilic carbon, affording the *trans* isomer, although in moderate stereoselectivity (Figure 5). However, with NaBH­(OAc)_3_, an intermediate arising from the substitution of an acetoxy group by the oxygen of the chiral inductor would be formed and the delivery of the hydride would take place intramolecularly.^16^ Therefore, in this case, the facial selectivity of the reduction depends on the relative stability of the conformations around the exocyclic C–N bond. The most stable conformation in which the bulky phenyl group lies away from the C-8 carbon, thus avoiding steric interactions, would induce the intramolecular attack of the reductant on the *re* face to predominantly give the *cis* isomer. Finally, DIBAL, an electrophilic reducing reagent, coordinates with the oxygen atom of the oxazolidine ring to generate an incipient iminium ion. The delivery of the hydride takes place from the same face of the C–O bond, leading to the *cis* isomer in excellent stereoselectivity.

**Figure 5:**
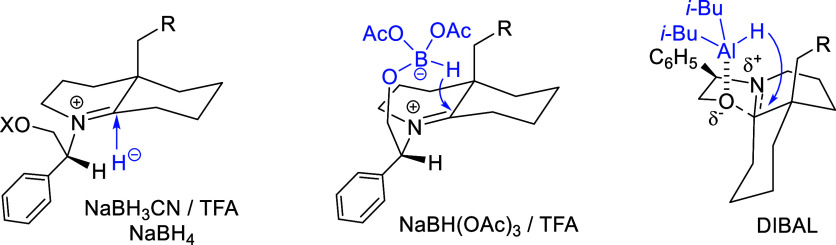
Stereoselectivity of the hydride reductions of perhydrooxazoloquinolines **9**–**12**.

Removal of the 2-phenylethanol moiety of the chiral inductor by reductive cleavage of the benzylic C–N bond under catalytic hydrogenation conditions allowed the isolation of a variety of enantiopure 4a-substituted *cis*- and *trans*-DHQs. Compounds *cis*-**19**, *cis*-**21**, *cis*-**22**, and *cis*-**23** were obtained from *cis*-**14a**, *cis*-**16a**, *cis*-**17a**, and *cis*-**18a**, respectively, by catalytic hydrogenation with simultaneous protection of the resulting secondary amines as *N*-Boc derivatives (Scheme 5). Due to the technical difficulties encountered in the separation of the *N*-Boc derivative *cis*-**20** from 2-phenylethanol in the hydrogenolysis of *cis*-**15a**, in this case the reduction was carried out in absence of Boc_2_O under acidic conditions (AcOH) to give the secondary amine *cis*-**24**. A subsequent protection as *N*-Boc derivative under conventional reaction conditions afforded *cis*-**20**. On the other hand, oxidation of the secondary alcohol present in *cis*-**21** and *cis*-**22** with Dess–Martin periodinane afforded ketones *cis*-**19** and *cis*-**25** in good yield.

**Scheme 5:**
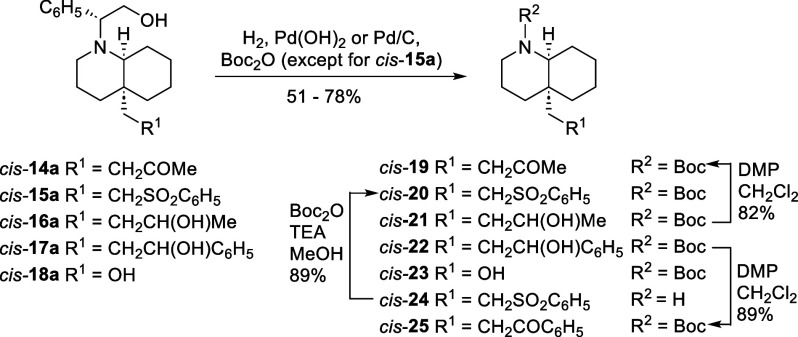
Access to Enantioenriched **4a**-Substituted *cis*-Decahydroquinolines

Similarly, removal of the *N*-benzylic substituent of DHQs *trans*-**14b**, *trans*-**15b**, and *trans*-**18b** by catalytic hydrogenation afforded the *N*-Boc protected DHQ *trans*-**19** and the secondary amines *trans*-**24** and *trans*-**26**, respectively (Scheme 6).

**Scheme 6:**
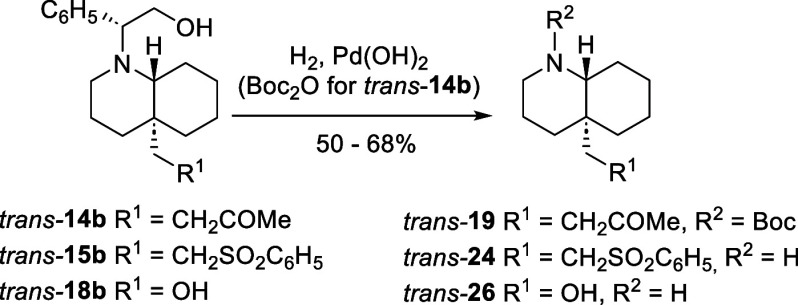
Access to Enantioenriched **4a**-Substituted *trans*-Decahydroquinolines

Finally, perhydrooxazoloquinoline **13** was envisaged as an advanced precursor of the *Myrioneuron* alkaloid myrioxazine A by simply removal of the 2-phenylethanol moiety and closure of the oxazinane ring. The reductive cleavage of the oxazolidine ring of **13** with DIBAL took place with excellent stereoselectivity and good yield to give *cis*-DHQ **27** (Scheme 7). The subsequent removal of the benzylic substituent under catalytic hydrogenation conditions afforded 8-hydroxymethyl-DHQ **28**. This alcohol is a known synthetic precursor of several *Myrioneuron*-type alkaloids^17^ isolated in North Vietnam from the small shrub *Myrioneuron nutans*, some of them showing interesting antimalarial activity. In our hands, treatment of **28** with formalin in methanol provided (−)-myrioxazine A. The spectroscopic data of our synthetic (−)-myrioxazine A matched those previously reported for the natural product,^18^ and its optical rotation was [α]_D_^20^ = – 24.4 (*c* 1.2, MeOH), [(+)-myrioxazine A^18^ [α]_D_^20^ = +21 (*c* 1.0, MeOH)].

**Scheme 7:**
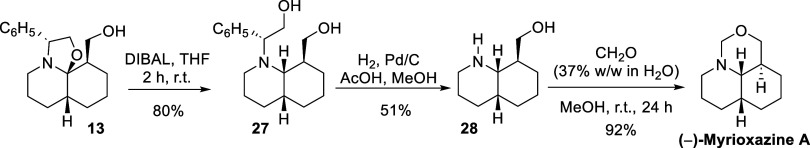
Enantioselective Synthesis of (−)-Myrioxazine A from Perhydrooxazoloquinoline **13**

## Conclusion

In summary, we have demonstrated the potential of perhydrooxazoloquinoline **2** for the stereoselective generation of DHQs containing a C_8a_ aza-quaternary stereocenter or a C_4a_ all-carbon quaternary stereocenter by using appropriate Grignard reagents or Michael acceptors and formaldehyde, respectively. Particularly notable is the excellent regio and stereoselectivity observed in the reaction of **2** with Michael acceptors, a transformation that involves a 1,4-asymmetric induction through a bicyclic enamine in which the only stereocenter is in a conformationally flexible appendage. Theoretical studies have clarified the mechanistic intricacies of this transformation, providing arguments for a proper understanding of the observed stereoselectivity. Interestingly, the reaction of **2** with formaldehyde is reversible, allowing the regioselective formation of either the angularly substituted hydroxymethyl derivative **12** or the C_8_-substituted derivative **13** depending on the reaction temperature. The stereoselective reduction of the C–O bond of the oxazolidine ring present in perhydrooxazoloquinolines **9**–**12** provides a procedure for the preparation of *cis* and *trans* DHQs bearing a C_4a_ all-carbon quaternary stereocenter. The best results were obtained when using DIBAL, which afforded the corresponding *cis* isomers in excellent stereoselectivities, increasing the synthetic value of the procedure. Finally, it is noteworthy that the formation of **13** involves the functionalization of the DHQ C_8_ position, thus opening an expeditious synthetic route to the Myrioneuron alkaloid (−)-myrioxazine A.

## Supplementary Material



## Data Availability

The data underlying this study are available in the published article and its Supporting Information.

## Notes

Complete experimental procedures including full characterization data for all new compounds, copies of ^1^H and ^13^C NMR spectra, X-ray crystallographic data for compounds **13** and *cis*-**15a**, details of the DFT calculations, and coordinates of stationary points shown in Figure 3 (PDF)

## References

[1] Li C., Ragab S. S., Liu G., Tang W. (2020). Enantioselective
Formation of Quaternary Carbon Stereocenters in Natural Product Synthesis:
A Recent Update. Nat. Prod. Rep..

[2] f Christoffers, J. ; Baro, A. Quaternary Stereocenters: Challenges and Solutions for Organic Synthesis; Wiley VCH: Weinheim, 2005.

[3] Kaga A., Chiba S. (2018). Synthesis of Tricyclic
Marine Alkaloids, Cylindricines, Lepadiformines,
Fasicularin, and Polycitorols: A Recent Update. Synthesis.

[4] b Kobayashi, J. ; Morita, H. . In The Alkaloids; Cordell, G. A. , Ed.; Academic Press: New York, 2005; Vol. 61, pp 1–57.

[5] Aquilina J. M., Smith M. W. (2023). Synthetic Studies toward the Myrioneuron Alkaloids. Synthesis.

[6] a Zhao, S. ; Sirasani, G. ; Andrade, R. B. Aspidosperma and Strychnos alkaloids: Chemistry and biology. In The Alkaloids: Chemistry and Biology; Knolker, H.-J. , Ed.; Academic Press, 2021; Vol. 86, pp 1–143. 10.1016/bs.alkal.2021.05.001. 34565505

[7] Piccichè M., Pinto A., Griera R., Bosch J., Amat M. (2022). Total Synthesis
of (−)-Cylindricine H. Org. Lett..

[8] Pinto A., Piccichè M., Griera R., Molins E., Bosch J., Amat M. (2018). Studies on
the Synthesis of Phlegmarine-Type
Lycopodium Alkaloids: Enantioselective Synthesis of (−)-Cermizine
B, (+)-Serratezomine E, and (+)-Luciduline. J. Org. Chem..

[9] See also: b Lin, C. H. ; Haadsma-Svensson, S. R. ; Mccall, R. B. ; Romero, A. G. ; Darlington, W. H. ; Ennis, M. D. Carboxamido-(1,2N)-carbocyclic-2-aminotetralin derivatives. WO 1992020655 A1, 1992.

[10] The overall yield in the transformation of lactam **1** into the chemoselectively reduced compound **2** has been optimized in the present work with respect to the 64% yield described in ref 7.

[11] Poupon E., François D., Kunesch N., Husson H.-P. (2004). New Piperidine Scaffolds via Nucleophilic
Reactivity of (−)-Phenyloxazolopiperidine. J. Org. Chem..

[12] Zhao Y., Truhlar D. G. (2008). The M06 Suite of
Density Functionals for Main Group Thermochemistry, Thermochemical
Kinetics, Noncovalent Interactions, Excited States, and Transition
Elements: Two New Functionals and Systematic Testing of Four M06-Class
Functionals and 12 Other Functionals. Theor.
Chem. Acc..

[13] Marenich A. V., Cramer C. J., Truhlar D. G. (2009). Universal
Solvation Model Based on
Solute Electron Density and on a Continuum Model of the Solvent Defined
by the Bulk Dielectric Constant and Atomic Surface Tensions. J. Phys. Chem. B.

[14] Lo J. C., Kim D., Pan C.-M., Edwards J. T., Yabe Y., Gui J., Qin T., Gutiérrez S., Giacoboni J., Smith M. W., Holland P. L., Baran P. S. (2017). Fe-Catalyzed C-C
Bond Construction from Olefins via Radicals. J. Am. Chem. Soc..

[15] Deslongchamps, P. Stereoelectronic Effects in Organic Chemistry. In Organic Chemistry Series; Baldwin, J. E. , Ed.; Pergamon Press, 1983; Vol. 1. Chapter 6.

[16] Canesi S., Bouchu D., Ciufolini M. A. (2004). Fully Stereocontrolled Total Synthesis of (−)-Cylindricine
C and (−)-2-Epicylindricine C: A Departure in Sulfonamide Chemistry. Angew. Chem., Int. Ed..

[17] Pham V. C., Jossang A., Grellier P., Sévenet T., Nguyen V. H., Bodo B. (2008). Structure and Total Synthesis of
(−)-Myrionidine and (−)-Schoberine, Antimalarial Alkaloids
from Myrioneuron nutans. J. Org. Chem..

[18] Pham V. C., Jossang A., Chiaroni A., Sévenet T., Bodo B. (2002). Asymmetric Synthesis of Myrioxazines A and B, Novel Alkaloids of
Myrioneuron nutans. Tetrahedron Lett..

