# Supplementary material for: Enantioselective Access to Decahydroquinolines Bearing a C4a or C8a Quaternary Stereocenter from a Common Intermediate Total Synthesis of (−)-Myrioxazine A
Source: J Org Chem. 2025 Apr 11;90(16):5642–50. doi: 10.1021/acs.joc.5c00321 (PMC12173215; doi:10.1021/acs.joc.5c00321)
Supplement: Supplementary file 1 [file jo5c00321_si_001.pdf]

Supporting Information

Enantioselective Access to Decahydroquinolines Bearing a C_{4a} or C_{8a} Quaternary Stereocenter from a Common Intermediate. Total Synthesis of (–)-Myrioxazine A

Arnau Calbó,[†] Miriam Piccichè,[†] Llorenç Rubert,[†] Eisuke Comas-Iwasita,[†] Rosa Griera,[†] Carolina Estarellas,[‡] F. Javier Luque,*[‡] Joan Bosch,[†] and Mercedes Amat*[†]

[†]Laboratory of Organic Chemistry, Faculty of Pharmacy and Food Sciences, and Institute of Biomedicine (IBUB), University of Barcelona, 08028-Barcelona, Spain

[‡]Departament de Nutrició, Ciències de l'Alimentació i Gastronomia, Facultat de Farmàcia i Ciències de l'Alimentació, Institut de Biomedicina (IBUB) and Institut de Química Teòrica i Computacional (IQTCUB), 08921 Santa Coloma de Gramenet, Spain

I) Experimental procedures and spectroscopic data: pages S2-S17

II) Copies of ^{1}H and ^{13}C NMR spectra: pages S18-S73

III) X- ray crystallographic data for compounds **13** and *cis*-**15a**: pages S74-S100

IV) Computational data: pages S101-S107

V) References: pages S108-S109

I) Experimental procedures and spectroscopic data

General Information. All air-sensitive reactions were performed under a dry argon or nitrogen atmosphere with dry, freshly distilled solvents using standard procedures. Evaporation of solvent was accomplished with a rotatory evaporator. Drying of organic extracts during the workup of reactions was performed over anhydrous Na_2SO_4. Thin-layer chromatography was done on SiO_2 (silica gel 60 F_{254}), and the spots were located by UV light and a 1% $KMnO_4$ solution. Chromatography refers to flash column chromatography and was carried out on SiO_2 (silica gel 60, 230−400 mesh). NMR spectra were recorded on a Varian VNMRS-400 or Mercury 400 spectrometer [400 MHz (^{1}H) and 100.6 MHz (^{13}C)], and chemical shifts are reported in δ values, in parts per million (ppm) relative to Me_4Si (0 ppm) or relative to residual chloroform (7.26 ppm, 77.0 ppm) as an internal standard. Data are reported in the following manner: chemical shift, multiplicity, coupling constant (*J*) in hertz (Hz), integrated intensity, and assignment. Structural assignments were made with additional information from gCOSY and gHSQC experiments. IR spectra were performed in a spectrophotometer Nicolet Avatar 320 FTIR, and only noteworthy IR absorptions (cm^{-1}) are listed. Optical rotations were measured on a Perkin-Elmer 241 polarimeter. $[\alpha]^D$ values are given in 10^{-1} deg. cm^2 g^{-1}. High-resolution mass spectra (HRMS) were performed by Centres Científics i Tecnològics de la Universitat de Barcelona.

(3*R*,7a*R*,11a*S*)-3-Phenylperhydrooxazolo[2,3-*j*]quinoline (2). MeOH (12 mL, 295.7 mmol) was added dropwise via a syringe pump over a period of 6 h to a refluxing solution of the thiolactam derived from **1**[1] (1.04 g, 3.61 mmol) and $NaBH_4$ (2.18 g, 55.6 mmol) in *t*-BuOH (28 mL). Water was added to the cooled mixture and the resulting solution was extracted with CH_2Cl_2. The combined organic phases were dried and concentrated. Flash chromatography (95:5 hexane–EtOAc, SiO_2 treated with Et_3N) afforded hemiaminal **2** (904 mg, 97%) as a colorless oil: $[\alpha]^{23}_D$ + 258.3 (*c* 1.0, $CHCl_3$); ^{1}H-NMR (400 MHz, $CDCl_3$, COSY, *g*-HSQC) δ 7.40-7.36 (m, 2H, ArH), 7.34-7.30 (m, 2H, ArH), 7.28-7.23 (m, 1H, ArH), 4.39 (t, *J* = 7.6 Hz, 1H, H-3), 4.21 (t, *J* = 7.6 Hz, 1H, H-2), 3.67-3.62 (m, 1H, H-2), 2.85-2.74 (m, 2H, H-5), 2.04 (td, *J* = 13.2, 4.8 Hz, 1H, H-11), 1.92-1.82 (m, 2H), 1.74-1.56 (m, 4H), 1.53-1.33 (m, 5H), 1.29-1.23 (m, 1H); ^{13}C{^{1}H} NMR (100.6 MHz, $CDCl_3$) δ 140.8 (C-Ar), 128.4 (CH-Ar), 127.8 (CH-Ar), 127.7 (CH-Ar), 127.5 (CH-Ar), 94.7 (C-11a), 72.2 (C-2), 62.4 (C-3), 43.4 (C-5), 36.1 (C-7a), 31.4 (C-11), 28.9 (CH_2), 27.7 (CH_2), 23.9 (CH_2), 20.4 ($2CH_2$); HRMS (ESI-TOF) m/z: $[M + H]^+$ Calcd for $C_{17}H_{24}NO$ 258.1852; Found 258.1853.

General Procedure for the Reaction of Perhydrooxazoloquinoline 2 with Grignard Reagents. The Grignard reagent (1.0 M solution in Et_2O, 2.0 mmol) was slowly added to a stirring solution of

2 (1.0 mmol) in anhydrous THF (9 mL) at –78 °C under an argon atmosphere, and the stirring was continued at this temperature for 30 min and at room temperature for 18 h. Then, aqueous saturated NH_4Cl and CH_2Cl_2 were added, the phases were separated, and the aqueous phase was extracted with CH_2Cl_2. The combined organic extracts were dried and concentrated, and the resulting residue was purified by flash chromatography.

*(4aR,8aS)-8a-Allyl-1-[(R)-1-phenyl-2-hydroxyethyl]decahydroquinoline (**3**) and its 8a-epimer.* Following the general procedure, from **2** (900 mg, 3.5 mmol) and allylmagnesium bromide (7.0 mL of a 1.0 M solution in Et_2O, 7.0 mmol) in anhydrous THF (18.3 mL), **3** (635 mg, 61%) and its 8a-epimer (31 mg, 3%) were obtained as yellowish oils after flash chromatography (9.5:0.5 hexane–EtOAc). **3**: $[\alpha]^{20}_D$ –10.9 (*c* 1.0, $CHCl_3$); IR (NaCl): 3415 (OH), 3067 (C=CH), 1457 (C=C) cm^{-1}; ^{1}H-NMR ($CDCl_3$, 400MHz, COSY, HETCOR) δ 7.22-7.30 (m, 5H, ArH), 5.60 (ddd, J = 7.6, 9.8, 17.6 Hz, 1H, C*H*=CH_2), 4.90 (dd, J = 1.2, 10.0 Hz, 1H, CH=C*H*$_2$), 4.82 (d, J = 18.0 Hz, 1H, CH=C*H*$_2$), 4.27 (dd, J = 6.0, 10.8 Hz, 1H, H-1'), 3.87 (t, J = 10.6 Hz, 1H, H-2'), 3.70 (bs, 1H, OH), 3.43-3.48 (m, 1H, H-2'), 3.02-3.06 (m, 1H, H-2), 2.81 (td, J = 3.2, 12.0 Hz, 1H, H-2), 2.52 (dd, J = 7.6, 13.8 Hz 1H, H-1''), 2.08-2.14 (m, 1H), 1.98-2.05 (m, 1H), 1.73-1.91 (m, 3H), 1.63-1.72 (m, 1H), 1.45-1.53 (m, 4H, H-4a), 1.32 (dd, J = 7.2, 13.6 Hz, 1H, H-1''), 1.20- 1.27 (m, 3H); ^{13}C{^{1}H} NMR ($CDCl_3$, 100.6MHz) δ 139.5 (C*q*-Ar), 135.2 (*C*=CH_2), 129.1 (CH-Ar), 128.2 (CH-Ar), 127.5 (CH-Ar), 116.9 (C=*C*H_2), 61.2 (C-2'), 59.4 (C-8a), 59.0 (C-1'), 39.2 (C-2), 37.6 (C-4a), 36.7 (C-1''), 33.1 (CH_2), 27.1 (CH_2), 26.4 (CH_2), 25.5 (CH_2), 21.4 (CH_2), 20.7 (CH_2); HRMS (ESI-TOF) m/z: $[M + H]^+$ Calcd for $C_{20}H_{30}NO$ 300.2332, Found 300.2330. 8a-epimer of **3**: $[\alpha]^{20}_D$ –83.1 (*c* 1.04, $CHCl_3$); IR (NaCl): 3417, 2931 cm^{-1}; ^{1}H-NMR ($CDCl_3$, 400MHz,) δ 7.30-7.38 (m, 5H, ArH), 5.86-5.98 (m, 1H, C*H*=CH_2), 5.19 (d, J = 13.6 Hz, 2H, CH=C*H*$_2$), 4.36-4.38 (m, 1H), 3.93 (t, J = 8.4 Hz, 1H), 3.38-3.48 (m, 1H), 3.18 (bs, 1H, OH), 2.94-3.06 (m, 2H), 2.72-2.78 (m, 1H), 2.35 (dd, J = 4.8, 13.8 Hz, 1H), 1.86-1.92 (m, 1H), 1.70-1.78 (m, 3H), 1.57-1.64 (m, 1H), 1.21-1.38 (m, 5H), 1.06-1.19 (m, 2H), 0.68-0.75 (m, 1H); ^{13}C{^{1}H} NMR ($CDCl_3$, 100.6MHz) δ 142.2 (C*q*-Ar), 135.1 (*C*=CH_2), 128.4 (CH-Ar), 128.3 (CH-Ar), 127.1 (CH-Ar), 117.7 (C=*C*H_2), 61.5 (C-2'), 59.7 (C-8a), 58.4 (C-1'), 43.8 (C-4a), 38.5 (C-2), 33.7 (CH_2), 30.5 (CH_2), 30.0 (CH_2), 27.0 (CH_2), 26.1 (CH_2), 24.3 (CH_2), 23.0 (CH_2); HRMS (ESI-TOF) m/z: $[M + H]^+$ Calcd for $C_{20}H_{30}NO$ 300.2332; Found 300.2331.

*(4aR,8aS)-8a-Ethynyl-1-[(R)-1-phenyl-2-hydroxyethyl]decahydroquinoline (**4**).* Following the general procedure, from **2** (206 mg, 0.8 mmol) and ethynylmagnesium chloride (1.6 mL of a 1.0 M solution in THF, 1.6 mmol) in anhydrous THF (4.3 mL), compound **4** (127 mg, 56%) was obtained as a yellow oil after flash chromatography (9.5:0.5 hexane–EtOAc): $[\alpha]^{20}_D$ –15.1 (*c* 0.465, $CHCl_3$); IR (NaCl): 3293, 2926 cm^{-1}; ^{1}H-NMR ($CDCl_3$, 400MHz, COSY, HETCOR) δ 7.43 (d, J = 7.2 Hz,

2H, ArH), 7.32 (t, *J* = 7.6 Hz, 2H, ArH), 7.22 (t, *J* = 7.2 Hz, 1H, ArH), 4.42 (dd, *J* = 6.8, 5.2 Hz, 1H, H-1'), 4.22 (dd, *J* = 7.0, 11.6 Hz, 1H, H-2'), 4.05 (dd, *J* = 4.8, 11.6 Hz, 1H, H-2'), 2.90-2.96 (m, 1H, H-2), 2.74-2.78 (m, 1H, H-2), 2.62 (s, 1H, C≡CH), 2.31 (dt, *J* = 3.4, 12.0 Hz, 1H), 2.19 (bs, 1H, OH), 1.64-1.77 (m, 3H), 1.52-1.56 (m, 1H), 1.35-1.50 (m, 6H, H-4a), 1.18-1.33 (m, 2H, H-2); $^{13}C\{^1H\}$ NMR ($CDCl_3$, 100.6MHz) δ 142.5 (C*q*-Ar), 128.1 (CH-Ar), 127.7 (CH-Ar), 126.4 (CH-Ar), 85.7 (*C*≡CH), 76.5 (C≡*C*H), 62.1 (C-2'), 61.1 (C-8a), 60.1 (C-1'), 47.2 (C-4a), 42.8 (C-2), 36.3 (CH_2), 30.4 (CH_2), 29.1 (CH_2), 26.4 (CH_2), 25.6 (CH_2), 23.5 (CH_2); HRMS (ESI-TOF) m/z: $[M + H]^+$ Calcd for $C_{19}H_{26}NO$ 284.2009; Found 284.2006.

*(4aR,8aS)-1-[(R)-1-Phenyl-2-hydroxyethyl]-8a-vinyldecahydroquinoline (**5**).* Following the general procedure (reaction time: 3 h at room temperature, then 7 h at reflux), from **2** (180 mg, 0.7 mmol) and vinylmagnesium bromide (1.4 mL of a 1.0 M solution in THF, 1.4 mmol) in anhydrous THF (3.7 mL), compound **5** (86 mg, 43%) was obtained as a yellow oil after flash chromatography (9:1 hexane–EtOAc): $[\alpha]^{20}_D$ +50.2 (*c* 0.305, $CHCl_3$); IR (NaCl): 3402, 2926 cm^{-1}; ^1H-NMR ($CDCl_3$, 400MHz, COSY, HETCOR) δ 7.43 (d, *J* = 7.6 Hz, 2H, ArH), 7.32 (t, *J* = 7.6 Hz, 2H, ArH), 7.22 (t, *J* = 7.2 Hz, 1H, ArH), 6.3 (dddd, *J* = 18.6, 11.8, 1.2 Hz, 1H, C*H*=CH_2), 5.44 (dd, *J* = 1.6, 11.2 Hz, 1H, CH=C*H*2), 5.29 (dd, *J* = 1.6, 18.0 Hz, 1H, CH=C*H*2), 4.29 (t, *J* = 6.2 Hz, 1H, H-1'), 3.95 (dd, *J* = 7.2, 11.2 Hz, 1H, H-2'), 3.81 (dd, *J* = 5.6, 11.2 Hz, 1H, H-2'), 2.89-3.03 (m, 2H, H-2), 2.18 (dt, *J* = 3.2, 12.8 Hz, 1H), 1.95 (bs, 1H, OH), 1.43-1.64 (m, 7H, H-4a), 1.34-1.38 (m, 1H), 1.21-1.32 (m, 4H, H-2), 1.08- 1.19 (m, 1H); $^{13}C\{^1H\}$ NMR ($CDCl_3$, 100.6MHz) δ 143.5 (C*q*-Ar), 137.6 (*C*H=CH_2), 128.2 (CH-Ar), 127.5 (CH-Ar), 126.5 (CH-Ar), 116.3 (CH=*C*H2), 63.7 (C-2'), 61.2 (C-8a), 59.6 (C-1'), 46.6 (C-4a), 42.2 (C-2), 34.6 (CH_2), 30.2 (CH_2), 27.3 (CH_2), 26.7 (CH_2), 26.5 (CH_2), 23.3 (CH_2); HRMS (ESI-TOF) m/z: $[M + H]^+$ Calcd for $C_{19}H_{28}NO$ 286.2165; Found 286.2165.

General Procedure for the Reaction of Perhydrooxazoloquinoline 2 with Michael Acceptors. The Michael acceptor (**6**, **7** or **8**; 5 mmol) was added to a stirring solution of amine **2** (1 mmol) in MeOH or dioxane, and the resulting mixture was stirred at reflux temperature for 24 h. After cooling at room temperature, the solvent was evaporated and the resulting residue was purified by flash chromatography.

*(3R,7aS,11aR)-7a-(3-Oxobutyl)-3-phenyldecahydrooxazolo[2,3-j]quinoline (**9**).* Following the general procedure, from **2** (100 mg, 0.39 mmol) and methyl vinyl ketone (**6**; 0.16 mL, 1.95 mmol) in MeOH (2.0 mL), compound **9** (100 mg, 69%) was obtained as a colorless gum after flash chromatography (from 98:2 to 85:15 hexane–EtOAc): $[\alpha]^{23}_D$ –84.1 (c 1.0, $CHCl_3$); IR (NaCl): 1713 (CO) cm^{-1}; ^1H NMR (400 MHz, $CDCl_3$, COSY, *g*-HSQC) δ 7.38-7.31 (m, 4H, ArH), 7.29-7.23 (m,

1H, ArH), 4.14 (t, J = 7.6 Hz, 1H, H-2), 3.92 (t, J = 7.6 Hz, 1H, H-3), 3.51 (t, J = 7.6 Hz, 1H, H-2), 2.58 (dd, J = 11.6, 5.6 Hz, 1H, H-5), 2.47-2.36 (m, 3H, H-2', H-5 and H-6) , 2.34-2.25 (m, 1H, H-2'), 2.20 (s, 3H, CH_3), 2.02-1.96 (m, 1H, H-6), 1.90-1.79 (m, 2H), 1.75-1.64 (m, 2H), 1.57-1.37 (m, 7H), 1.25-1.18 (m, 1H); $^{13}C\{^1H\}$ NMR (100.6 MHz, $CDCl_3$) δ 210.6 (CO), 140.8 (C-Ar), 128.5 (CH-Ar), 127.8 (CH-Ar), 127.6 (CH-Ar), 95.9 (C-11a), 72.6 (C-2), 62.1 (C-3), 42.1 (C-5), 39.3 (C-7a), 38.8 (C-2'), 33.3 (CH_2), 29.8 (CH_3), 27.5 (CH_2), 26.8 (C-6), 22.5 (CH_2), 21.7 (CH_2), 21.4 (CH_2), 21.0 (CH_2); HRMS (ESI-TOF) m/z: $[M + H]^+$ Calcd for $C_{21}H_{30}NO_2$ 328.2271; Found 328.2275.

*(3R,7aS,11aR)-7a-(3-oxo-3-phenylpropyl)-3-phenyldecahydrooxazolo[2,3-j]quinoline (**10**).* Following the general procedure, from **2** (78 mg, 0.30 mmol) and phenyl vinyl ketone (**7**; 200 mg, 1.52 mmol) in MeOH (2 mL), compound **10** (90 mg, 76%) was obtained as a yellowish oil after flash chromatography (from 99:1 to 95:5 hexane–EtOAc): $[\alpha]^{23}_D$ – 29.6 (c 1.0, $CHCl_3$); IR (NaCl): 1685 (CO) cm^{-1}; ^1H NMR (400 MHz, $CDCl_3$, COSY, g-HSQC) δ 8.03-7.98 (m, 2H, ArH), 7.59-7.53 (m, 1H, ArH), 7.50-7.43 (m, 2H, ArH), 7.34-7.22 (m, 5H, ArH), 4.13 (t, J = 7.6 Hz, 1H, H-2), 3.92 (t, J = 7.6 Hz, 1H, H-3), 3.49 (t, J = 7.6 Hz, 1H, H-2), 3.03 (ddd, J = 14.8, 12.0, 4.8 Hz, 1H, H-2'), 2.80 (ddd, J = 14.8, 12.0, 5.2 Hz, 1H, H-2'), 2.63-2.42 (m, 3H, H-5 and H-1'), 2.21-2.12 (m, 1H, H-1'), 1.97-1.82 (m, 2H), 1.79-1.41 (m, 9H), 1.37-1.30 (m, 1H); $^{13}C\{^1H\}$ NMR (100.6 MHz, $CDCl_3$) δ 202.0 (CO), 140.7 (C-Ar), 137.1 (C-Ar), 132.7 (CH-Ar), 128.5 (CH-Ar), 128.5 (CH-Ar), 128.2 (CH-Ar), 127.8 (CH-Ar), 127.5 (CH-Ar), 96.0 (C-11a), 72.6 (C-2), 62.1 (C-3), 42.1 (C-5), 39.6 (C-7a), 33.7 (C-2'), 33.5 (CH_2), 28.0 (C-1'), 27.9 (CH_2), 22.5 (CH_2), 21.7 (CH_2), 21.6 (CH_2), 21.0 (CH_2); HRMS (ESI-TOF) m/z: $[M + H]^+$ Calcd for $C_{26}H_{32}NO_2$ 390.2428; Found 390.2426.

*(3R,7aS)- 3-Phenyl -7a-[2-(phenylsulfonyl)ethyl]decahydrooxazolo[2,3-j]quinoline (**11**).* Following the general procedure (reaction time 48 h), from **2** (0.10 g, 0.39 mmol) and phenyl vinyl sulfone (**8**; 0.34 g, 1.98 mmol) in anhydrous dioxane (2 mL), compound **11** (84 mg, 51%; somewhat unstable and difficult to purify) was obtained as a yellowish gum after flash chromatography (from 98:2 to 8:2 hexane-AcOEt): ^1H NMR (400 MHz, $CDCl_3$): δ 7.97–7.93 (m, 2H, ArH), 7.68–7.63 (m, 1H, ArH), 7.59–7.55 (m, 2H, ArH), 7.30-7.23 (m, 3H, ArH), 7.17 (d, J = 5.8 Hz, 2H, ArH), 4.08 (t, J = 6.2 Hz, 1H, H-3), 3.85 (t, J = 6.2 Hz, 1H, H-2), 3.35 (t, J = 6.2 Hz, 1H, H-2), 3.30–3.24 (m, 1H, H-2'), 3.05 (td, J = 10.7, 3.5 Hz, 1H, H-2'), 2.47 (dd, J = 9.0, 4.3 Hz, 1H, H-5), 2.37 (td, J = 9.6 and 3.0 Hz, 1H, H-5), 2.25 (td, J = 10.7 and 3.6 Hz, 2H, H-1'), 1.88 (td, J = 11.2 and 4.1 Hz, 1H, H-7), 1.76 (dd, J = 10.2 and 3.0 Hz, 1H, H-9), 1.66–1.62 (m, 2H, H-11), 1.54–1.38 (m, 7H, H-8, H-6, H-9, H-10), 1.24 (d, J = 5.68 Hz, 1H, H-7); $^{13}C\{^1H\}$ NMR (100.6 MHz, $CDCl_3$): δ 140.4 (C-Ar), 133.5 (C-Ar), 129.3 (CH-Ar), 128.7 (CH-Ar), 128.3 (CH-Ar), 127.8 (CH-Ar),126.5 (CH-Ar), 125.6 (CH-Ar), 95.7 (C11a), 72.6 (C2), 62.1 (C3), 52.8 (C2'), 42.0 (C5), 39.5 (C7a), 33.2 (C8), 28.6 (C7),

26.2 (C1’), 22.5 (C11), 21.8 (C9), 21.3 (C6), 20.9 (C10); HRMS (ESI-TOF) m/z: $[M+H]^+$ Calcd for $C_{25}H_{32}NO_3S$, 426.2097; Found 426.2093.

(3*R*,7a*S*,11a*R*)-7a-(Hydroxymethyl)-3-phenyldecahydrooxazolo[2,3-*j*]quinoline (12) and (3*R*,7a*R*, 11*R*, 11a*R*)-11-(Hydroxymethyl)-3-phenyldecahydrooxazolo[2,3-*j*]quinoline (13). Formalin (0.8 mL of a 37% w/w in H_2O solution, 10.6 mmol) was added to a stirring solution of **2** (545 mg, 2.11 mmol) in MeOH (10.6 mL), and the resulting mixture was stirred at reflux temperature for 24 h. After cooling at room temperature, the solvent was evaporated. Flash chromatography (from 95:5 to 8:2 hexane–EtOAc) afforded compound **12** (53.5 mg, 9%) as a colorless oil and compound **13** (257 mg, 42%) as a white solid. When the reaction was carried out at room temperature, compound **12** was obtained in 73% yield. **12**: $[\alpha]^{23}_D$ –73.2 (*c* 0.8, $CHCl_3$); IR (NaCl): 3500 (OH) cm^{-1}; ^1H NMR (400 MHz, $CDCl_3$, COSY, *g*-HSQC) δ 7.32-7.37 (m, 4H, ArH), 7.27-7.31 (m ,1H, ArH), 4.47-4.49 (m, 1H, OH), 4.17-4.25 (m, 2H, H-2 and H-1’), 3.94 (t, *J* = 8.0 Hz, 1H, H-3), 3.70 (t, *J* = 8.0 Hz, 1H, H-2), 3.59 (dd, *J* = 10.4, 6.0 Hz, 1H, H-1’), 2.61-2.67 (m, 1H, H-5), 2.46-2.54 (m, 1H, H-5), 1.87-2.00 (m, 3H), 1.67-1.82 (m, 2H), 1.43-1.66 (m, 5H), 1.24-1.32 (m ,1H), 1.16-1.21 (m, 1H); $^{13}C\{^1H\}$ NMR (100.6 MHz, $CDCl_3$) δ 139.9 (C-Ar), 128.7 (CH-Ar), 127.9 (CH-Ar), 127.8 (CH-Ar), 97.6 (C-11a), 71.7 (C-2), 70.2 (C-1’), 62.4 (C-3), 42.4 (C-5), 40.1 (C-7a), 31.0 (CH_2), 29.6 (CH_2), 22.3 (CH_2), 22.0 (CH_2), 21.3 (CH_2), 20.7 (CH_2); HRMS (ESI-TOF) m/z: $[M + H]^+$ Calcd for $C_{18}H_{26}NO_2$ 288.1958; Found 288.1964. **13**: mp 76-78 °C (hexane–CH_2Cl_2); $[\alpha]^{23}_D$ –77.1 (*c* 0.9, $CHCl_3$); IR (NaCl): 3284 (OH) cm^{-1}; ^1H NMR (400 MHz, $CDCl_3$, COSY, *g*-HSQC) δ 7.38-7.28 (m, 5H, ArH), 5.92 (d, *J* = 9.6 Hz, 1H, OH), 4.47 (dd, *J* = 9.0, 7.6 Hz, 1H, H-3), 4.34 (t, *J* = 7.6 Hz, 1H, H-2), 4.01 (t, *J* = 10.4 Hz, 1H, H-1’), 3.89 (dd, *J* = 9.0, 7.6 Hz, 1H, H-2), 3.42 (td, *J* = 10.4, 3.2 Hz, 1H, H-1’), 2.98-2.89 (m, 1H, H-5), 2.82-2.75 (m, 1H, H-5), 2.39-2.30 (m, 1H, H-11), 2.00-1.93 (H-7a), 1.87-1.72 (m, 3H), 1.56-1.32 (m, 6H), 1.29-1.17 (m, 1H); $^{13}C\{^1H\}$ NMR (100.6 MHz, $CDCl_3$) δ 138.2 (C-Ar), 128.9 (CH-Ar), 128.3 (CH-Ar), 128.1 (CH-Ar), 97.3 (C-11a), 72.8 (C-2), 65.1 (C-1’), 61.7 (C-3), 42.6 (C-5), 36.8 (C-11), 36.5 (C-7a), 28.4 (CH_2), 27.4 (CH_2), 26.5 (CH_2), 20.2 (CH_2), 19.8 (CH_2); HRMS (ESI-TOF) m/z: $[M + H]^+$Calcd for $C_{18}H_{26}NO_2$ 288.1958; Found 288.1962.

General Procedure for the Reductive Opening of the Oxazolidine Ring.

A. Using $NaBH_3CN$. TFA (0.5 mmol) and $NaBH_3CN$ (5 mmol) were sequentially added to a stirring solution of **9** or **11** (1.0 mmol) in anhydrous CH_2Cl_2 at 0 °C, and the stirring was continued at room temperature for 4 h or 72 h. Then water was added, and the mixture was extracted with

CH_2Cl_2. The combined organic extracts were dried and concentrated, and the resulting residue was purified by flash chromatography.

*(4aS,8aS)- and (4aS,8aR)-1-[(R)-2-Hydroxy-1-phenylethyl]-4a-(3-oxobutyl)decahydroquinoline (cis-**14a** and trans-**14b**)*. Following the general procedure (reaction time 4 h), from **9** (173 mg, 0.53 mmol), TFA (20 µL, 0.26 mmol), and $NaBH_3CN$ (166 mg, 2.64 mmol) in anhydrous CH_2Cl_2 (6.4 mL), compounds *cis*-**14a** (36.5 mg, 21%) and *trans*-**14b** (83.7 mg, 48%) were obtained as viscous colorless liquids after flash chromatography (from 9:1 to 5:5 hexane–EtOAc). *cis*-**14a**: $[\alpha]^{23}_D$ + 5.2 (*c* 1.0, $CHCl_3$); IR (NaCl): 1712 (CO), 3454 (OH) cm^{-1}; ^1H NMR (400 MHz, $CDCl_3$, COSY, *g*-HSQC) δ 7.33-7.29 (m, 3H, ArH), 7.23-7.29 (m, 2H, ArH), 3.70-3.77 (m, 1H, H-1'), 3.62-3.69 (m, 2H, H-1' and H-2'), 2.64 (dd, *J* = 11.6, 3.6 Hz, 1H, H-8a), 2.51-2.46 (m, 1H), 2.45-2.39 (m, 1H), 2.37-2.30 (m, 2H), 2.19 (s, 3H, H-4''), 2.17-2.11 (m, 1H), 1.91-1.83 (m, 1H), 1.79 (dd, *J* = 16.6, 4.4 Hz, 1H), 1.75-1.66 (m, 2H), 1.64-1.60 (m, 1H), 1.48-1.30 (m, 5H), 1.28-1.20 (m, 1H), 1.18-1.08 (m, 1H), 1.03-0.99 (m, 1H); $^{13}C\{^1H\}$ NMR (100.6 MHz, $CDCl_3$) δ 209.9 (CO), 141.2 (C-Ar), 128.6 (CH-Ar), 128.4 (CH-Ar), 127.4 (CH-Ar), 68.0 (C-1'), 63.1 (C-2'), 62.2 (C-8a), 41.9 (CH_2), 37.8 (CH_2), 36.4 (C-4a), 36.4 (CH_2), 32.1 (CH_2), 30.0 (C-4''), 25.6 (CH_2), 25.3 (CH_2), 21.8 (CH_2), 21.2 (CH_2), 19.1 (CH_2); HRMS (ESI-TOF) m/z: $[M + H]^+$ Calcd for $C_{21}H_{32}NO_2$ 330.2428; Found 330.2428. *trans*-**14b**: $[\alpha]^{23}_D$ –55.6 (*c* 1.0, $CHCl_3$); IR (NaCl): 1713 (CO), 3446 (OH) cm^{-1}; ^1H NMR (400 MHz, $CDCl_3$, COSY, *g*-HSQC) δ 7.28-7.35 (m, 3H, ArH), 7.13-7.15 (m, 2H, ArH), 4.30 (dd, *J* = 5.0, 10.9 Hz, 1H), 3.98 (t, *J* = 10.5 Hz, 1H, H-2'), 3.57 (dd, *J* = 5.0, 10.2 Hz, 1H, H-2'), 2.98 (d, *J* = 11.24 Hz, 1H, H-2), 2.20-2.32 (m, 4H), 2.18 (s, 4H, H-3''/H-8a), 2.00-2.08 (m, 1H), 1.85-1.92 (m, 2H), 1.77 (dt, *J* = 2.8, 12.0 Hz, 1H, H-2), 1.59-1.68 (m, 1H), 1.45-1.51 (m, 3H), 1.35-1.40 (m, 4H), 0.78-0.91 (m, 2H); $^{13}C\{^1H\}$ NMR (100.6 MHz, $CDCl_3$) δ 209.5 (CO), 136.3 (C-Ar) 128.7 (CH-Ar), 128.3 (CH-Ar), 127.5 (CH-Ar), 67.4 (C-8a), 60.2 (CH_2), 59.0 (C-1'), 47.3 (CH_2), 37.5 (CH_2), 37.1 (C-4a), 37.1 (CH_2), 35.8 (CH_2), 30.1 (C-3''), 25.7 (CH_2), 24.3 (CH_2), 21.9 (CH_2), 20.5 (CH_2), 20.4 (CH_2); HRMS (ESI-TOF) m/z: $[M + H]^+$ Calcd for $C_{21}H_{32}NO_2$ 330.2428; Found 330.2426.

*(4aS,8aS)- and (4aS,8aR)-1-[(R)-2-Hydroxy-1-phenylethyl]-4a-[2-(phenylsulfonyl)ethyl]decahydroquinoline (cis-**15a** and trans-**15b**)*. Following de general procedure (reaction time 72 h), from **11** (235 mg, 0.55 mmol), TFA (21 µl, 0.28 mmol) and $NaBH_3CN$ (174 mg, 2.75 mmol) in anhydrous CH_2Cl_2 (5 mL), decahydroquinolines *cis*-**15a** (80 mg, 34%) as a white solid and *trans*-**15b** (95.4 mg, 40%) as a viscous liquid were obtained after flash chromatography (from 9:1 to 5:5 hexane-EtOAc). *cis*-**15a**: mp152-153 ºC; $[\alpha]^{D}_{23}$=+10.0 (c 1.0, $CHCl_3$); ^1H NMR (400 MHz, $CDCl_3$) δ (ppm): 7.91 – 8.02 (m, 2H, H-Ar''), 7.64–7.74 (m, 1H, H-Ar''), 7.56–7.64 (m, 2H, H-Ar''), 7.26–7.34 (m, 3H, H-Ar'), 7.21–7.24 (m, 2H, H-Ar'), 3.72 (dd, *J*

= 11.2, 5.0 Hz, 1H, H-2’), 3.64 (dd, J = 11.5, 4.4 Hz, 1H, H-2’), 3.52-3.58 (m, 1H, H-1’), 3.32 (ddd, J = 13.6, 12.5, 4.8 Hz, 1H, H-2’’), 3.03 (ddd, J = 13.6, 12.5, 4.8 Hz, 1H, H-2’’), 2.63 (d, J = 10.3 Hz, 1H, H-8a), 2.25 –2.36 (m, 2H, H-2), 2.07–2.23 (m, 2H, H-1’’), 1.85 (td, J = 13.7, 5.1 Hz, 1H), 1.77-1.80 (m, 1H, OH), 1.70-1.77 (m, 1H, H-6), 1.67 (td, J = 12.5, 3.95, 1H, H-8), 1.38-1.50 (m, 2H, H-7/H-8), 1.32-1.35 (m, 1H, H-5), 1.28-1.31 (m, 1H, H-7/H-3), 1.20 (d, J = 4.5 Hz, 1H, H-5), 1.10 (dt, J = 12.6, 3.7 Hz, 1H, H-6), 0.90-0.97 (m, 3.2 Hz, 1H, H-4); $^{13}C\{^1H\}$ NMR (101 MHz, $CDCl_3$) δ (ppm): 141.6 (CAr), 139.3 (CAr), 133.5 (CAr), 129.2 (CAr), 128.6 (CAr), 128.1 (CAr), 127.5 (CAr), 67.4 (C1’), 63.4 (C2’), 60.1 (C8a), 51.5 (C2’’), 42.5 (C2), 36.5 (C4a), 36.3 (C5), 30.5 (C1’’), 26.0 (C4), 25.3 (C6), 21.4 (C3), 21.1 (C7), 18.4 (C8); HRMS (ESI-TOF) m/z: Calcd for $C_{25}H_{34}NO_3S$ 428.2254; Found 428.2245. *trans*-**15b**: $[\alpha]^{23}_D$ –92.0 (*c* 1.0, $CHCl_3$); IR (NaCl): 3503 (OH), 1146, 1306 (SO_2) cm^{-1}; ^1H NMR (400 MHz, CD_3OD, COSY, *g*-HSQC) δ 8.00-7.94 (m, 2H, ArH), 7.79-7.72 (m, 1H, ArH), 7.71-7.62 (m, 2H, ArH), 7.33-7.27 (m, 2H, ArH), 7.27-7.15 (m, 3H, ArH), 4.19 (dd, J = 8.6, 6.6 Hz, 1H, H-1’), 3.89 (dd, J = 10.6, 8.6 Hz, 1H, H-2’), 3.67 (dd, J = 10.6, 6.6 Hz, 1H, H-2’), 3.05 (tt, J = 9.2, 8.0 Hz, 2H, H-2’’), 2.93-2.86 (dm, J = 8.4 Hz, 1H, H-2), 2.30-2.17 (m, 2H, H-1’’, H-8), 2.07-2.01 (m, 1H, H-8a), 1.91-1.72 (m, 3H, H-1’, H-2, H-5), 1.45-1.23 (m, 7H), 1.19-1.09 (m, 1H, H-1’’), 0.90-0.86 (m, 1H, H-4), 0.83-0.73 (m, 1H, H-4); $^{13}C\{^1H\}$ NMR (100.6 MHz, CD_3OD) δ 140.1 (C-Ar), 138.0 (C-Ar), 135.0 (CH-Ar), 130.6 (CH-Ar), 129.8 (CH-Ar), 129.3 (CH-Ar), 129.1 (CH-Ar), 128.2 (CH-Ar), 68.6 (C-8a), 62.6 (C-2’’), 61.0 (C-1’), 51.8 (C-2’’), 49.4 (C-2), 38.1 (C-4a), 36.7 (C-4), 36.3 (C-3), 26.5 (C-5), 25.4 (C-8), 22.8 (C-6), 21.2 (C-7), 21.0 (C-1’’); HRMS (ESI-TOF) m/z: $[M + H]^+$ Calcd for $C_{25}H_{34}NO_3S$ 428.2254; Found 428.2254.

B. Using $NaBH(OAc)_3$. TFA (0.5 mmol) and $NaBH(OAc)_3$ (5 mmol) were sequentially added to a stirring solution of **9** or **11** (1.0 mmol) in anhydrous CH_2Cl_2 at 0 °C, and the stirring was continued at room temperature for 4 h or 72 h. Then water was added, and the mixture was extracted with CH_2Cl_2. The combined organic extracts were dried and concentrated, and the resulting residue was purified by flash chromatography.

Following the general procedure (reaction time 4 h), from **9** (201 mg, 0.61 mmol), TFA (23 μL, 0.31 mmol) and $NaBH(OAc)_3$ (646 mg, 3.05 mmol) in anhydrous CH_2Cl_2 (7.8 mL), decahydroquinolines *cis*-**14a** (128 mg, 62%) and *trans*-**14b** (49 mg, 15%) were obtained after flash chromatography (from 9:1 to 1:1 hexane–EtOAc).

Following the general procedure (reaction time 72 h), from **11** (182 mg, 0.42 mmol), TFA (16 μl, 0.21 mmol) and $NaBH(OAc)_3$ (423 mg, 2.13 mmol) in anhydrous CH_2Cl_2 (5.3 ml), decahydroquinolines *cis*-**15a** (85 mg, 47%) and *trans*-**15b** (16 mg, 9%) were obtained after flash chromatography (from 9:1 to 5:5 hexane-EtOAc).

C. Using $NaBH_4$. $NaBH_4$ (5 mmol) was added to a solution of **9**, **10**, **11** or **12** (1 mmol) in MeOH, and the mixture was stirred for the period indicated. Then water was added, and the mixture was extracted with CH_2Cl_2. The combined organic extracts were dried and concentrated, and the resulting residue was purified by flash chromatography.

*(4aS,8aS)- and (4aS,8aR)-4a-(3-Hydroxybutyl)-1-[(R)-2-hydroxy-1-phenylethyl]decahydroquinoline (cis-**16a** and trans-**16b**).* Following the general procedure (time reaction 1 h), from **9** (636 mg, 1.94 mmol) and $NaBH_4$ (367 mg, 9.71 mmol) in MeOH (21.6 mL), decahydroquinolines *cis*-**16a** (128 mg, 20%) and *trans*-**16b** (408 mg, 63%) were obtained as colorless liquids after flash chromatography (from 8:2 to 1:1 hexane–EtOAc). *cis*-**16a**: IR (NaCl): 3384 (OH) cm^{-1}; ^1H NMR (500 MHz, $CDCl_3$, COSY, *g*-HSQC) δ 7.32-7.24 (m, 5H, ArH), 3.84-3.62 (m, 4H, H-1' and H-2' and H-3''), 2.69-2.60 (m, 1H, H-8a), 2.58-2.52 (m, 1H, H-2), 2.39 (td, J = 9.6, 2.4 Hz, 1H), 2.01-1.93 and 1.85-1.52 [(m) and (m), 7H], 1.47-1.36 (m, 5H), 1.34-1.26 (m, 2H), 1.26-1.21 (m, 3H, H-4''), 1.15-1.00 (m, 2H); $^{13}C\{^1H\}$ NMR (125 MHz, $CDCl_3$) δ 141.1 (C-Ar), 128.6 (CH-Ar), 128.3 (CH-Ar), 127.4 (CH-Ar), 68.8 (C-3''), 68.3 (C-1'), 63.2 and 62.8 (C-8a), 62.7 (C-2'), 41.4 (C-2), 36.6 (C-4a and CH_2), 34.3 and 34.0 (CH_2), 32.7 and 32.4 (CH_2), 26.2 and 25.9 (CH_2), 25.6 (CH_2), 23.8 and 23.4 (C-4''), 21.9 (CH_2), 21.3 (CH_2), 19.6 and 19.5 (CH_2); HRMS (ESI-TOF) m/z: $[M + H]^+$ Calcd for $C_{21}H_{34}NO_2$ 332.2584; Found 332.2580. *trans*-**16b**: IR (NaCl): 3390 (OH) cm^{-1}; ^1H NMR (400 MHz, $CDCl_3$, COSY, *g*-HSQC) δ 7.37-7.28 (m, 3H, ArH), 7.17-7.13 (m, 2H, ArH), 4.38-4.31 (m, 1H, H-1'), 4.06-3.97 (m, 1H, H-2'), 3.83-3.74 (m, 1H, H-3''), 3.63-3.57 (m, 1H, H-2'), 3.05-2.98 (m, 1H), 2.28-2.19 (m, 2H), 1.99-1.88 (m, 1H), 1.87-1.66 (m, 3H), 1.65-1.45 (m, 4H), 1.44-1.26 (m, 6H), 1.25-1.19 (m, 4H), 0.90-0.73 (m, 2H); $^{13}C\{^1H\}$ NMR (100.6 MHz, $CDCl_3$) δ 136.3 (C-Ar), 128.5 (CH-Ar), 128.2 (CH-Ar), 127.4 (CH-Ar), 68.9 (C-1'), 67.4 (C-8a), 59.9 (C-2'), 58.5 (C-3''), 47.1 (C-2), 37.2 (C-4a), 35.8 (CH_2), 35.7 (CH_2), 32.1 (C-2''), 25.7 (CH_2), 24.2 (CH_2), 23.8 (C-4''), 23.5 (CH_2), 22.2 (CH_2), 20.2 (CH_2); HRMS (ESI-TOF) m/z: $[M + H]^+$ Calcd for $C_{21}H_{34}NO_2$ 332.2584; Found 332.2588.

*(4aS,8aS)- and (4aS,8aR)-1-[(R)-2-Hydroxy-1-phenylethyl]-4a-[3-hydroxy-3-phenylpropyl]decahydroquinoline (cis-**17a** and trans-**17b**).* Following the general procedure (reaction time 24 h), from **10** (50 mg, 0.13 mmol) and $NaBH_4$ (24 mg, 0.63 mmol) in MeOH (1.4 mL), decahydroquinolines *cis*-**17a** (9.5 mg, 19%) as a colorless oil and *trans*-**17b** (22 mg, 43%) as a yellowish oil were obtained after flash chromatography (from 9:1 to 7:3 hexane–EtOAc). *cis*-**17a**: IR (NaCl): 3418 (OH) cm^{-1}; ^1H NMR (500 MHz, $CDCl_3$, COSY, *g*-HSQC) δ 7.41-7.36 (m, 4H, ArH), 7.33-7.22 (m, 6H, ArH), 4.73 and 4.66-4.62 [(t, J = 6.4 Hz) and (m), 1H, H-3''], 3.73-3.59 (m, 3H, H-1' and H-2'), 2.67-2.56 (m, 1H, H-8a), 2.53-2.41 (m, 1H, H-2), 2.40-2.28 (m, 1H, H-2), 1.86-1.59 (m, 7H), 1.59-1.47 (m, 1H), 1.45-1.19 (m, 8H), 1.14-0.97 (m, 2H); $^{13}C\{^1H\}$ NMR (125

MHz, $CDCl_3$) δ 145.2 and 144.9 (C-Ar), 141.3 (C-Ar), 128.6 (CH-Ar), 128.5 (CH-Ar), 128.4 (CH-Ar), 128.3 (CH-Ar), 127.5 (CH-Ar), 127.4 (CH-Ar), 127.3 (CH-Ar), 125.9 (CH-Ar), 75.4 and 75.3 (C-3'), 68.2 and 68.1 (C-1'), 62.9 (C-2'), 62.4 (C-8a), 41.6 and 41.4 (CH_2), 36.7 and 36.6 (C-4a and CH_2), 34.5 and 34.0 (CH_2), 32.8 and 32.5 (CH_2), 29.7 (CH_2), 25.9 (CH_2), 25.6 (CH_2), 21.9 and 21.8 (CH_2), 21.3 (CH_2), 19.4 and 19.3 (CH_2); HRMS (ESI-TOF) m/z: $[M + H]^+$ Calcd for $C_{26}H_{36}NO_2$ 394.2741; Found 394.2745. *trans*-**17b**: IR (NaCl): 3435 (OH) cm^{-1}; ^1H NMR (400 MHz, $CDCl_3$, COSY, *g*-HSQC) δ 7.40-7.27 (m, 8H, ArH), 7.15-7.10 (m, 2H, ArH), 4.67 (t, *J* = 6.0 Hz, 1H, H-3"), 4.32-4.22 (m, 1H, H-1'), 4.02-3.88 (m, 1H, H-2'), 3.60-3.51 (m, 1H, H-2'), 3.01-2.84 (m, 1H, H-2), 2.21-2.12 (m, 3H), 2.00-1.87 (m, 1H), 1.83-1.72 (m, 1H), 1.69-1.50 (m, 6H), 1.47-1.28 (m, 5H), 1.16-1.06 (m, 1H), 0.84-0.70 (m, 2H); $^{13}C\{^1H\}$ NMR (100.6 MHz, $CDCl_3$) δ 144.8 (C-Ar), 128.6 (CH-Ar), 128.5 (CH-Ar), 128.2 (CH-Ar), 127.6 (CH-Ar), 125.9 (CH-Ar), 125.8 (CH-Ar), 75.3 (C-3"), 67.4 (C-8a), 60.0 (C-2'), 58.8 (C-1'), 47.3 (C-2), 37.2 (C-4a), 35.7 (CH_2), 35.6 (CH_2), 32.1 (CH_2), 32.0 (CH_2), 25.6 (CH_2), 24.2 (CH_2), 22.0 (CH_2), 21.6 (CH_2), 20.1 (CH_2); HRMS (ESI-TOF) m/z: $[M + H]^+$ Calcd for $C_{26}H_{36}NO_2$ 394.2741; Found 394.2746.

Following the general procedure (reaction time 72 h), from **11** (100 mg, 0.23 mmol) and $NaBH_4$ (45 mg, 1.18 mmol) in MeOH (2.5 mL), decahydroquinolines *cis*-**15a** (10,6 mg, 11%) and *trans*-**15b** (42.4 mg, 43%) were obtained after flash column chromatography (from 9:1 to 1:1 hexane–EtOAc).

*(4aR,8aS)- and (4aR,8aR)-4a-(Hydroxymethyl)-1-[(R)-2-hydroxy-1-phenylethyl]decahydroquinoline (cis-**18a** and trans-**18b**).* Following the general procedure (reaction time 4 h), from **12** (180 mg, 0.57 mmol) and $NaBH_4$ (108 mg, 2.85 mmol) in MeOH (3 mL), decahydroquinolines *cis*-**18a** (26 mg, 16%) as a colorless solid and *trans*-**18b** (48 mg, 29%) as an oily liquid were obtained after flash chromatography (from 9:1 to 4:6 hexane-EtOAc). *cis*-**18a**: $[\alpha]^{23}_D$ –22.6 (*c* 1.0, $CHCl_3$); IR (NaCl): 3382 (OH) cm^{-1}; ^1H NMR (400 MHz, $CDCl_3$, COSY, *g*-HSQC) δ 7.34 (d, *J* = 4.4 Hz, 4H, ArH), 7.32-7.23 (m, 1H, ArH), 4.10-3.98 (m, 2H, H-2' and H-1''), 3.75-3.62 (m, 2H, H-1' and H-2'), 3.44 (d, *J* = 10.5 Hz, 1H, H-1''), 3.20 (dd, *J* = 11.8, 4.2 Hz, 1H, H-8a), 2.44-2.36 (m, 2H, H-2), 2.03-1.84 (m, 2H), 1.86-1.69 (m, 2H), 1.65-1.52 (m, 1H), 1.55-1.30 (m, 4H), 1.22-1.15 (m, 2H), 1.16-1.07 (m, 1H); $^{13}C\{^1H\}$ NMR (100.6 MHz, $CDCl_3$) δ 141.1 (C-Ar), 128.7 (CH-Ar), 128.2 (CH-Ar), 127.6 (CH-Ar), 73.0 (C-1"), 66.7 (C1'), 63.4 (C-2'), 57.9 (C-8a), 42.5 (C-2), 37.6 (C-4a), 34.8 (CH_2), 25.9 (CH_2), 25.2 (CH_2), 22.1 (CH_2), 21.3 (CH_2), 17.8 (CH_2); HRMS (ESI-TOF) m/z: $[M + H]^+$ Calcd for $C_{18}H_{28}NO_2$ 290.2115; Found 290.2112. *trans*-**18b**: $[\alpha]^{23}_D$ –79.5 (c 1.0, $CHCl_3$); ^1H NMR (400 MHz, $CDCl_3$, COSY, *g*-HSQC) δ 7.37–7.26 (m, 3H, H-Ar), 7.18–7.14 (m, 2H, H-Ar), 4.34 (dd, *J* = 9.9 and 5.4 Hz, 1H, H-1'), 4.24 (d, *J* = 10.9 Hz, 1H, H-1'), 3.99 (t, *J* = 10.2 Hz, 1H, H-2'), 3.74–3.63 (m, 2H, H-2'/H-1''), 3.07 (ddt, *J* = 11.2, 4.2 and 2.0 Hz, 1H, H-2), 2.36–2.27 (m, 2H, H-8a), 2.14–2.01 (m, 1H), 1.95 (dt, *J* = 13.3, 3.0 Hz, 1H),

1.89 (ddd, J = 12.8, 11.2, 3.1 Hz, 1H, H-2), 1.73 (ddt, J = 13.2, 4.1 and 1.9 Hz, 1H), 1.59–1.41 (m, 6H), 1.40–1.30 (m, 1H), 1.28–1.23 (m, 1H), 1.00–0.85 (m, 2H); $^{13}C\{^1H\}$ NMR (100.6 MHz, $CDCl_3$) δ 135.8 (C-Ar), 128.9 (CH-Ar), 128.4 (CH-Ar), 127.7 (CH-Ar), 67.6 (C8a), 64.8 (C1''), 61.3 (C2'), 59.8 (C1'), 48.0 (C2), 38.2 (CH_2), 38.1 (C4a), 35.5 (CH_2), 25.8 (CH_2), 24.9 (CH_2), 22.9 (CH_2), 20.8 (CH_2); HRMS (ESI-TOF) m/z: $[M+H]^+$ Calcd for $C_{18}H_{28}NO_2$ 290.2115; Found, 290.2118.

D. Using DIBAL. DIBAL (1M solution in THF, 10 mmol) was added to a stirring solution of **9**, **10**, **11** or **12** (1 mmol) in anhydrous THF, and the stirring was continued at room temperature for 4 h. Then a 2M solution of NaOH was added, and the mixture was extracted with CH_2Cl_2. The combined organic extracts were dried and concentrated, and the resulting residue was purified by flash chromatography.

Following the general procedure, from **9** (55.6 mg, 0.17 mmol) and DIBAL (0.85 mL, 0.85 mmol) in anhydrous THF (1.9 mL), decahydroquinoline *cis*-**16a** (34 mg, 60%) was obtained as a colorless liquid after flash chromatography (from 8:2 to 1:1 hexane–EtOAc).

Following the general procedure, from **10** (144 mg, 0.37 mmol) and DIBAL (3.7 mL, 3.7 mmol) in anhydrous THF (4.1 mL), decahydroquinoline *cis*-**17a** (92 mg, 63%) was obtained as a colorless oil after flash chromatography (from 95:5 to 8:2 hexane–EtOAc).

Following the general procedure, from **11** (300 mg, 1.17 mmol) and DIBAL (11.7 mL, 11.7 mmol) in anhydrous THF (12 mL), decahydroquinoline *cis*-**15a** (440 mg, 88%) was obtained after flash chromatography (from 9:1 to 7:3 hexane–EtOAc).

Following the general procedure (reaction time 2 h), from **12** (50 mg, 0.17 mmol) and DIBAL (1.74 mL, 1.74 mmol) in anhydrous THF (1.93 mL), decahydroquinoline *cis*-**18a** (27.2 mg, 54%) was obtained as a colorless solid after flash chromatography (from 7:3 hexane–EtOAc to EtOAc).

General Procedure for the Debenzylation Reactions. A solution of the decahydroquinoline (1 mmol) and di-*tert*-butyl dicarbonate (except for *cis*-**15a**, *trans*-**15b** and *trans*-**18b**; 1.2 mmol) in MeOH containing the catalyst (Pd/C or $Pd(OH)_2$) was stirred under hydrogen at rt for 24 h. The catalyst was removed by filtration, the filtrate was concentrated, and the resulting residue was purified.

*(4aS,8aS)-1-(tert-Butoxycarbonyl)-4a-(3-oxobutyl)decahydroquinoline (cis-**19**).* Following the general procedure, from *cis*-**14a** (56 mg, 0.19 mmol), di-*tert*-butyl dicarbonate (51 mg, 0.23 mmol) and $Pd(OH)_2$ (22 mg) in MeOH (3.8 mL), decahydroquinoline *cis*-**19** (41 mg, 65%) was obtained as a colorless oil after flash chromatography (from 99:1 to 9:1 hexane-Et_2O): $[\alpha]^{23}_D$ +12.0 (c 1.0,

$CHCl_3$); IR (NaCl): 1715 and 1676 (CO) cm^{-1}; ^1H NMR (400 MHz, CD_3OD, COSY, *g*-HSQC) δ 3.95-3.88 (m, 1H, H-2), 3.82-3.70 (m, 1H, H-8a), 2.96-2.78 (m, 1H, H-2), 2.53-2.32 (m, 2H, H-2'), 2.14 (s, 3H, H-4'), 1.94 (td, *J* = 13.8, 4.6 Hz, 2H, H-3, H-4), 1.84-1.75 (m, 2H, H-5, H-7), 1.72-1.59 (m, 1H, H-3), 1.45 [s, 9H, $C(CH_3)_3$], 1.50-1.36 (m, 6H, H-6, H-7, H-8, H-1'), 1.33-1.19 (m, 2H, H-5, H-6), 1.17-1.09 (m, 1H, H-4); $^{13}C\{^1H\}$ NMR (101 MHz, CD_3OD) δ 211.7 (C-3'), 157.0 (CO), 80.8 [$\mathit{C}(CH_3)_3$], 59.2 and 57.5 (C-8a), 39.7 and 38.6 (C-2), 38.2 (C-2'), 37.1 (C-6), 36.3 (C-4a), 32.5 (C-7), 29.9 (C-4'), 28.8 [$C(\mathit{C}H_3)_3$], 26.7 (C-5), 26.2 (C-4), 25.8 (C-8), 22.1 (C-1'), 21.7 (C-3); HRMS (ESI-TOF) m/z: $[M + H]^+$ Calcd for $C_{18}H_{32}NO_3$ 310.2377; Found 310.2372.

*(4aS,8aR)-1-(tert-Butoxycarbonyl)-4a-(3-Oxobutyl)decahydroquinoline (trans-**19**).* Following the general procedure, from *trans*-**14b** (205 mg, 0.62 mmol), di-*tert*-butyl dicarbonate (163 mg, 0.75 mmol) and $Pd(OH)_2$ (82 mg) in MeOH (12.4 mL), decahydroquinoline *trans*-**19** (124 mg, 65%) was obtained as an oil after flash chromatography (from 99:1 to 9:1 hexane-Et_2O): $[\alpha]^D{}_{23}$ +57.2 (c 1.0, $CHCl_3$); ^1H NMR (400 MHz, $CDCl_3$) δ (ppm): 4.28 (ddt, *J* = 13.0, 3.6, 1.8 Hz, 1H, H-2), 2.91 (dd, *J* = 12.5, 3.4 Hz, 1H, H-8a), 2.57-2.67 (m, 1H, H-2), 2.35-2.49 (m, 1H, H-8), 2.21-2.28 (m, 2H, H-2'), 2.16 (s, 3H, H-4'), 1.88-2.01 (m, 1H, H-1'), 1.76-1.88 (m, 2H, H-7/H-8), 1.51-1.71 (m, 4H, H-1'/H-4/H-5/H-6), 1.46 [m, 9H, $C(CH_3)_3$], 1.24-1.42 (m, 3H, H-3/H-6), 1.10-1.23 (m, 1H, H-7), 1.05 (td, *J* = 13.9, 4.0 Hz, 1H, H-4), 0.86-0.98 (td, *J* = 13.7, 4.2, 1H, H-5); $^{13}C\{^1H\}$ NMR (101 MHz, $CDCl_3$) δ (ppm): 209.1 (C3'), 155.5 (CO), 79.4 [$\mathit{C}(CH_3)_3$], 68.5 (C8a), 49.4 (C2), 38.1 (C4a), 37.9 (C2'), 37.1 (C4), 36.3 (C5), 29.8 (C4'), 28.6 [$C(\mathit{C}H_3)_3$], 27.7(C8), 27.5 (C7), 22.1 (C6), 21.3 (C3), 20.1 (C1'); HRMS (ESI-TOF) m/z: Calcd for $C_{18}H_{32}NO_3$ 310.2377; Found 310.2379.

*(4aS,8aS)-1-(tert-Butoxycarbonyl)-4a-(3-hydroxybutyl)decahydroquinoline (cis-**21**).* Following the general procedure, from *cis*-**16a** (54.4 mg, 0.17 mmol), di-*tert*-butyl dicarbonate (44 mg, 0.20 mmol) and Pd/C (22 mg) in MeOH (3.5 mL), decahydroquinoline *cis*-**21** (27 mg, 51%) was obtained as a colorless oil after flash chromatography (from 9:1 to 85:15 hexane–EtOAc): IR (NaCl): 3446 (OH), 1689, 1668 (CO) cm^{-1}; ^1H NMR (400 MHz, $CDCl_3$, COSY, *g*-HSQC) δ 4.05-3.62 (m, 3H, H-2, H-8a and H-3'), 2.90-2.73 (m, 1H, H-2), 1.89-1.58 (m, 6H), 1.54-1.22 (m, 18H), 1.18 (d, *J* = 6.4 Hz, 3H, CH_3), 1.14-1.06 (m, 1H); $^{13}C\{^1H\}$ NMR (100.6 MHz, $CDCl_3$) δ 155.6 (CO), 79.1 [$\mathit{C}(CH_3)_3$], 68.8 and 68.5 (C-3'), 57.9, 57.5, 55.9 and 55.2 (C-8a), 38.7 and 37.5 (C-2), 36.1 (CH_2), 35.2 (C-4a), 33.7 and 32.9 (CH_2), 32.4 (CH_2), 28.5 [$C(\mathit{C}H_3)_3$], 25.7 (2 CH_2), 25.1 and 24.9 (CH_2), 23.7 (CH_3), 21.1 (CH_2), 20.7 (CH_2); HRMS (ESI-TOF) m/z: $[M + H]^+$ Calcd for $C_{18}H_{34}NO_3$ 312.2533; Found 312.2525.

*(4aS,8aS)-1-(tert-Butoxycarbonyl)-4a-(3-hydroxy-3-phenylpropyl)decahydroquinoline (cis-**22**).* Following the general procedure, from *cis*-**17a** (72.7 mg, 0.19 mmol), di-*tert*-butyl dicarbonate (48.3 mg, 0.22 mmol) and Pd/C (29 mg) in MeOH (3.8 mL), decahydroquinoline *cis*-**22** (38 mg,

54%) was obtained as a yellowish oil after flash chromatography (from 95:5 to 8:2 hexane–EtOAc): IR (NaCl): 3444 (OH), 1687, 1667 (CO) cm^{-1}; ^{1}H NMR (400 MHz, $CDCl_3$, COSY, *g*-HSQC) δ 7.31-7.13 (m, 5H, ArH), 4.62-4.42 (m, 1H, H-3'), 3.97-3.46 (m, 2H, H-2 and H-8a), 2.82-2.59 (m, 1H, H-2), 1.83-1.51 (m, 7H), 1.47-1.25 (m, 14H), 1.23-0.97 (m, 4H); $^{13}C\{^{1}H\}$ NMR (100.6 MHz, $CDCl_3$) δ 155.6 and 155.4 (CO), 144.8 (C-Ar), 128.5 and 128.4 (CH-Ar), 127.6 and 127.3 (CH-Ar), 125.8 and 125.7 (CH-Ar), 79.1 and 78.9 [$C(CH_3)_3$], 75.4, 75.4 and 74.9 (C-3'), 57.7 and 55.0 (C-8a), 38.7 and 38.2 and 37.4 and 36.9 (C-2), 36.2 and 36.0 (C-4a), 35.7 and 35.5 (CH_2), 35.3 (CH_2), 33.9 and 33.4 (CH_2), 33.2 and 32.4 (CH_2), 28.5 [$C(CH_3)$], 25.6 (CH_2), 25.0 and 24.7 (CH_2), 21.1 (CH_2), 20.7 (CH_2); HRMS (ESI-TOF) m/z: $[M + H]^+$ Calcd for $C_{23}H_{36}NO_3$ 374.2690; Found 374.2691.

*(4aR,8aS)-1-(tert-Butoxycarbonyl)-4a-(hydroxymethyl)decahydroquinoline (cis-**23**)*. Following the general procedure, from *cis*-**18a** (27.2 mg, 0.09 mmol), di-*tert*-butyl dicarbonate (30 mg, 0.14 mmol) and Pd/C (11 mg) in MeOH (1.9 mL), decahydroquinoline *cis*-**23** (13 mg, 53%) was obtained as a yellowish oil after flash chromatography (from 95:5 to 8:2 hexane–EtOAc): $[\alpha]^{23}_{D}$ –10.6 (*c* 0.7, $CHCl_3$); IR (NaCl): 1666, 1688 (CO), 3449 (OH) cm^{-1}; ^{1}H NMR (400 MHz, $CDCl_3$, COSY, *g*-HSQC) δ 3.97-4.11 (m, 1H, H-8a), 3.78-3.88 (m, 1H, H-2), 3.62-3.71 (m, 1H, H-1'), 2.88-3.04 (m, 2H, H-1' and H-2), 1.73-1.96 (m, 4H), 1.57-1.63 (m, 1H), 1.48-1.56 (m, 2H), 1.46 [s, 9H, $(CH_3)_3$], 1.14-1.42 (m, 4H), 0.98-1.05 (m, 1H); $^{13}C\{^{1}H\}$ NMR (100.6 MHz, $CDCl_3$) δ 156.4 (CO), 79.8 [$C(CH_3)_3$], 66.6 (C-1'), 51.2 (C-8a), 38.8 (C-2), 38.2 (Cq), 33.5 (CH_2), 28.4 [$C(CH_3)_3$], 25.6 (CH_2), 25.3 (CH_2), 24.5 (CH_2), 21.1 (CH_2), 21.0 (CH_2); HRMS (ESI-TOF) m/z: $[M + H]^+$ Calcd for $C_{15}H_{28}NO_3$ 270.2064; Found 270.2068.

*(4aS,8aS)-4a-[2-(Phenylsulfonyl)ethyl]decahydroquinoline (cis-**24**)*. Following the general procedure, from *cis*-**15a** (58 mg, 0.136 mmol), AcOH (0.53 mL, 9.38 mmol) and Pd/C (23 mg) in MeOH (3 mL), decahydroquinoline *cis*-**24** (32 mg, 78%) was obtained as a yellowish oil after flash chromatography (from 1:1 hexane–EtOAc to EtOAc, then 9:1 EtOAc–Et_3N): $[\alpha]^{23}_{D}$ –5.8 (*c* 1.4, $CHCl_3$); IR (NaCl): 3334 (NH), 1146, 1306 (SO_2) cm^{-1}; ^{1}H NMR (500 MHz, $CDCl_3$, COSY, *g*-HSQC) δ 7.89-7.82 (m, 2H, ArH), 7.63-7.57 (m, 1H, ArH), 7.54-7.49 (m, 2H, ArH), 3.01-2.88 (m, 3H, H-2 and H-2'), 2.51-2.44 (m, 1H, H-2), 2.36-2.33 (m, 1H, H-8a), 1.98-1.89 (m, 1H), 1.85-1.75 (m, 1H), 1.69-1.43 (m, 4H), 1.42-1.16 (m, 7H), 1.06- 0.97 (m, 1H), 0.87-0.80 (m, 1H); $^{13}C\{^{1}H\}$ NMR (125 MHz, $CDCl_3$) δ 139.1 (C-Ar), 133.6 (CH-Ar), 129.3 (CH-Ar), 128.0 (CH-Ar), 58.9 (C-8a), 51.2 (C-2'), 34.4 (C-4a), 29.7 (CH_2), 29.6 ($2CH_2$), 27.7 (CH_2), 22.3 ($2CH_2$), 21.0 ($2CH_2$); HRMS (ESI-TOF) m/z: $[M + H]^+$ Calcd for $C_{17}H_{26}NO_2S$ 308.1679; Found 308.1687.

*(4aS,8aR)-4a-[2-(phenylsulfonyl)ethyl]decahydroquinoline (trans-**24**).* Following the general procedure (reaction time 72 h), from *trans*-**15b** (0.21 g, 0.49 mmol) and $Pd(OH)_2$ (84 mg) in MeOH (10 mL), a crude residue was obtained. The residue was taken up with CH_2Cl_2 and the organic solution was extracted with 2M HCl. The aqueous phase was basified with 2M NaOH and extracted with CH_2Cl_2. The organic extracts were dried and concentrated to give *trans*-**24** (75 mg, 50%) as a white solid : mp 110 – 112 °C; $[\alpha]^D{}_{23}$ + 6.7 (c 1.0, $CHCl_3$); IR (NaCl): 1306, 2927 cm^{-1}; ^{1}H NMR (400 MHz, $CDCl_3$) δ 7.95 (dd, *J* = 8.24 and 1.32 Hz, 2H, ArH), 7.68–7.64 (m, 1H, ArH), 7.60–7.56 (m, 2H, ArH), 3.03–2.97 (m, 1H, H-2'), 2.97–2.92 (m, 2H, H-2), 2.65–2.58 (ddd, *J* = 23.2, 11.6 and 4.72 Hz, 1H, H-2'), 2.32–2.28 (m, 1H, H-8a), 2.18–2.06 (m, 1H, H1'), 1.88–1.79 (m, 1H, H1'), 1.68 (d, *J* = 5 Hz, 1H, H-8), 1.43–1.29 (m, 8H, H-4 H-5 H-6 H-7), 1.27–1.25 (m, 1H, H-8), 1.20–1.07 (m, 1H), 1.00–0.84 (m, 1H); $^{13}C\{^1H\}$ NMR (101 MHz, $CDCl_3$) δ 139.1 (C-Ar), 133.6 (CH-Ar), 129.2 (CH-Ar), 128.1 (CH-Ar), 64.9 (C-8a), 51.4 (C2), 47.6 (C2'), 35.7 (Cq), 35.37 (CH_2), 34.8 (CH_2), 27.7 (CH_2), 25.2 (C8), 22.1 (CH_2), 20.6 (CH_2), 18.9 (CH_2); HRMS (ESI-TOF) m/z: $[M+H]^+$ Calcd for $C_{17}H_{26}NO_2S$, 308.1679; Found 308.1685.

*(4aR,8aR)-4a-(Hydroxymethyl)decahydroquinoline (trans-**26**).* Following the general procedure (reaction time 72 h), from *trans*-**18b** (0.18 g, 0.61 mmol) and $Pd(OH)_2$ (72 mg) in MeOH (12 mL), a crude residue was obtained, which was purified as in the above preparation of *trans*-**24** to give *trans*-**26** (70 mg, 68%) as a yellow oil: $[\alpha]^D{}_{23}$ + 9.6 (c 1.0, $CHCl_3$); IR (NaCl) *v*: 3283, 2928 cm^{-1}; ^{1}H NMR (400 MHz, $CDCl_3$): δ 4.32 (dd, *J* = 10.9 and 2.2 Hz, 1H, H-1'), 3.53 (dd, *J* = 10.8 and 1.1 Hz, 1H, H-1'), 3.12 (dt, , *J* = 10.9 and 1.6 Hz, 1H, H-7), 2.78–2.72 (m, 1H, H-7), 2.50 (dd, *J* = 12.4 and 2.7 Hz, 1H, H-8a), 2.31–2.19 (m, 1H, H-6), 1.83–1.78 (m, 1H, H-2), 1.76–1.66 (m, 1H, H-5), 1.76–1.66 (m, 1H, H-8), 1.60–1.54 (m, 1H, H-6), 1.51–1.40 (m, 2H, H-4), 1.51–1.40 (m, 1H, H-8), 1.39-1.32 (m, 1H, H-2), 1.27–1.25 (m, 1H, H-3), 1.20–1.07 (m, 1H, H-5), 1.00–0.84 (m, 1H, H-3); $^{13}C\{^1H\}$ NMR (101 MHz, $CDCl_3$) δ 67.9 (C1'), 66.0 (C8a), 47.6 (C7), 40.2 (C5), 36.1 (Cq), 35.4 (C3), 28.4 (C8), 25.2 (C2), 23.5 (C6), 21.5 (C4); HRMS (ESI-TOF) m/z: $[M + H]^+$ Calcd for $C_{10}H_{20}NO$ 170.1539; Found 170.1537.

(4a*S*,8a*S*)-1-(*tert*-Butoxycarbonyl)-4a-[2-(phenylsulfonyl)ethyl]decahydroquinoline (*cis*-20). Di-*tert*-butyl dicarbonate (10.6 mg, 0.05 mmol) and Et_3N (9 µL, 0.06 mmol) were added to a solution of amine *cis*-**24** (14 mg, 0.05 mmol) in MeOH (1.3 mL) and the mixture was stirred at room temperature for 16 h. The resulting mixture was washed with saturated aqueous $NaHCO_3$, dried and concentrated. Flash chromatography (from 9:1 to 1:1 hexane–EtOAc) afforded carbamate *cis*-**20** (18 mg, 89%) as a yellowish oil: $[\alpha]^{23}{}_D$ +11.0 (*c* 0.8, $CHCl_3$); IR (NaCl): 1685 (CO), 1146 (SO_2) cm^{-1}; ^{1}H NMR (400 MHz, $CDCl_3$, COSY, *g*-HSQC) δ 7.91-7.88 (m, 2H, ArH), 7.68-7.62 (m, 1H, ArH), 7.58-7.53 (m, 2H, ArH), 3.97-3.54 (m, 2H, H-2 and H-8a), 3.24-3.01 (m, 1H, H-2'), 2.94

(td, J = 12.8, 4.0 Hz, 1H, H-2'), 2.85-2.67 (m ,1H, H-2), 1.99-1.67 (m, 4H), 1.48-1.35 (m, 13H), 1.34-1.10 (m, 3H), 1.05-0.94 (m, 1H); $^{13}C\{^1H\}$ NMR (100.6 MHz, $CDCl_3$) δ 155.0 (CO), 139.2 (C-Ar), 133.6 (CH-Ar), 129.2 (CH-Ar), 128.0 (CH-Ar), 79.4 [$C(CH_3)_3$], 57.4 and 55.2 (C-8a), 51.4 (C-2'), 38.3 and 37.2 (C-2), 35.9 (CH_2), 35.3 (C-4a), 30.0 and 29.6 (CH_2), 28.5 [$C(CH_3)_3$], 25.3 (CH_2), 25.0 (CH_2), 24.5 (CH_2), 20.9 (CH_2), 20.5 (CH_2); HRMS (ESI-TOF) m/z: $[M + H]^+$ Calcd for $C_{22}H_{34}NO_4S$ 408.2203; Found 408.2196.

(4aS,8aS)-1-(*tert*-Butoxycarbonyl)-4a-(3-oxo-3-phenylpropyl)decahydroquinoline (*cis*-25). Dess–Martin periodinane (31.3 mg, 0.05 mmol) was added to a stirred solution of *cis*-**22** (19.7 mg, 0.053 mmol) in anhydrous CH_2Cl_2 (0.4 mL) at room temperature. After 16 h, 10% aqueous NaOH was added and the resulting mixture was extracted with CH_2Cl_2. The combined organic extracts were dried and concentrated. Flash chromatography (8:2 hexane–EtOAc) afforded decahydroquinoline *cis*-**25** (17.5 mg, 89%) as a yellowish oil: $[\alpha]^{23}_D$ + 17.6 (c 0.7, $CHCl_3$); IR (NaCl): 1686 (CO) cm^{-1};^1H NMR (400 MHz, $CDCl_3$, COSY, *g*-HSQC) δ 7.98-7.93 (m, 2H, ArH), 7.58-7.52 (m, 1H, ArH), 7.49-7.42 (m, 2H, ArH), 4.09-3.68 (m, 2H, H-2 and H-8a), 3.18-2.69 (m, 3H, H-2 and H-2'), 2.03- 1.68 (m, 6H), 1.58-1.34 (m, 16H), 1.23-1.13 (m, 1H); $^{13}C\{^1H\}$ NMR (100.6 MHz, $CDCl_3$) δ 201.2 and 200.7 (CO), 155.4 and 155.2 (NCO), 137.0 (C-Ar), 132.9 (CH-Ar), 128.4 (CH-Ar), 128.1 (CH-Ar), 79.2 [$C(CH_3)_3$], 57.5 and 55.3 (C-8a), 38.5 and 37.4 (C-2), 36.1 (CH_2), 35.4 (C-4a), 32.5 (CH_2), 32.2 (CH_2), 28.4 [$C(CH_3)_3$], 25.8 (CH_2), 25.6 (CH_2), 25.1 and 24.7 (CH_2), 22.6 (CH_2), 21.1 and 20.7 (CH_2); HRMS (ESI-TOF) m/z: $[M + H]^+$ Calcd for $C_{23}H_{34}NO_3$ 372.2533; Found 372.2529.

Operating as above, from *cis*-**21** (20.9 mg, 0.07 mmol), DMP (40 mg, 0.09 mmol) in anhydrous CH_2Cl_2 (0.5 mL), decahydroquinoline *cis*-**19** (17 mg, 82%) was obtained as a colorless oil after flash chromatography (8:2 hexane–EtOAc).

(4a*R*, 8*S*, 8a*S*)-8-(Hydroxymethyl)-1-[(*R*)-2-hydroxy-1-phenylethyl]decahydroquinoline (27). DIBAL (9.5 mL of a 1M solution in THF, 9.5 mmol) was added to a stirring solution of **13** (272 mg, 0.95 mmol) in anhydrous THF (10.5 mL), and the stirring was continued at room temperature for 2 h. Then, a 2M solution of NaOH was added, and the mixture was extracted with CH_2Cl_2. The combined organic extracts were dried and concentrated. Flash chromatography (from 7:3 hexane–EtOAc to EtOAc) afforded **27** (219 mg, 80%) as a colorless solid: mp 101–106 °C (hexane–CH_2Cl_2); $[\alpha]^{23}_D$ –7.4 (c 1.0, $CHCl_3$); IR (NaCl): 3356 (OH) cm^{-1}; ^1H NMR (400 MHz, $CDCl_3$, COSY, *g*-HSQC) δ 7.38-7.27 (m, 5H, ArH), 4.19-4.15 (m, 1H, H-1'), 4.02 (dd, J = 11.6, 6.0 Hz, 1H, H-2'), 3.93 (dd, J = 11.6, 4.4 Hz, 1H, H-2'), 3.76 (dd, J = 10.4, 3.6 Hz, 1H, H-1''), 3.58 (dd, J

= 10.4, 8.4 Hz, 1H, H-1''), 3.09 (dd, J = 6.8, 4.4 Hz, 1H, H-8a), 2.83 (td, J = 14.8, 3.2 Hz, 1H, H-2), 2.47-2.39 (m, 1H, H-2), 2.34-2.24 (m, 1H, H-8), 2.19-2.12 (m, 1H, H-4a), 1.78 (qd, J = 13.2, 4.0 Hz, 1H), 1.65-1.58 (m, 2H), 1.58-1.53 (m, 1H), 1.51-1.39 (m, 4H), 1.22-1.16 (m, 1H), 1.04-0.92 (m, 1H); $^{13}C\{^1H\}$ NMR (100.6 MHz, $CDCl_3$) δ 140.5 (C-Ar), 128.9 (CH-Ar), 128.4 (CH-Ar), 127.9 (CH-Ar), 70.0 (C-1''), 64.9 (C-2'), 64.5 (C-1'), 62.3 (C-8a), 42.4 (C-2), 33.9 (C-8), 31.4 (CH_2), 29.3 (C-4a), 29.2 (CH_2), 25.1 (CH_2), 20.2 (CH_2), 20.1 (CH_2); HRMS (ESI-TOF) m/z: $[M + H]^+$ Calcd for $C_{18}H_{28}NO_2$ 290.2115; Found 290.2111.

(4a*R*,8*S*,8a*S*)-8-(Hydroxymethyl)decahydroquinoline (28). A solution of **27** (219 mg, 0.76 mmol), and AcOH (2.99 mL, 52.21 mmol) in MeOH (15 mL) containing 40% Pd/C (87 mg) was stirred under hydrogen at room temperature for 16 h. The catalyst was removed by filtration over Celite®, and the filtrate was basified with a 2M solution of NaOH and extracted with CH_2Cl_2. The organic extracts were dried and concentrated. Flash chromatography (CH_2Cl_2, then from 8:2:0.1 to 8:2:0.25 CH_2Cl_2–MeOH–NH_4OH) afforded **28** (65 mg, 51%) as a colorless solid: mp 108–109 °C (CH_2Cl_2); $[\alpha]^{23}_D$ +2.8 (c 0.3, $CHCl_3$); ^1H NMR (400 MHz, $CDCl_3$) δ 3.60 (dd, J = 7.6, 3.6 Hz, 1H), 3.52-3.46 (m, 1H), 2.85-2.71 (m, 3H), 2.19-2.08 (m, 1H), 1.81-1.64 (m, 3H), 1.59-1.52 (m, 1H), 1.50-1.31 (m, 6H), 0.88-0.74 (m, 1H). The spectral data were consistent with those previously reported.[2]

(–)-Myrioxazine A. Formalin (1 mL of a 37% w/w in H_2O solution, 13 mmol) was added to a solution of aminoalcohol **28** (108.8 mg, 0.64 mmol) in MeOH (2 mL). After 2 h, 2M aqueous solution of NaOH was added and the mixture was extracted with CH_2Cl_2. The organic extracts were dried and concentrated. Flash chromatography (from 1:1 to 3:7 hexane–EtOAc) afforded (–)-myrioxazine A (106.8 mg, 92%) as a colorless oil: $[\alpha]^{23}_D$ – 24.4 (c 1.2, MeOH); ^1H NMR (400 MHz, $CDCl_3$) δ 4.50 (d, J = 10.4 Hz, 1H), 4.44 (d, J = 10.4 Hz, 1H), 3.89 (dd, J = 10.8, 4.4 Hz, 1H), 3.21 (t, J = 10.8 Hz, 1H), 3.17 (td, J = 12.0, 3.2 Hz, 1H), 2.81 (dd, J = 10.8, 4.8 Hz, 1H), 2.67-2.61 (m, 1H), 2.21 (qt, J = 11.2, 4.0 Hz, 1H), 1.91-1.83 (m, 1H), 1.82-1.76 (m, 1H), 1.64-1.52 (m, 4H), 1.50-1.31 (m, 4H), 0.79 (qd, J = 12.0, 3.2 Hz, 1H). The spectral data were consistent with those previously reported.[2]

II) Copies of ^{1}H and ^{13}C NMR spectra

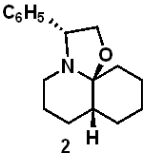

2

400 MHz, $CDCl_3$

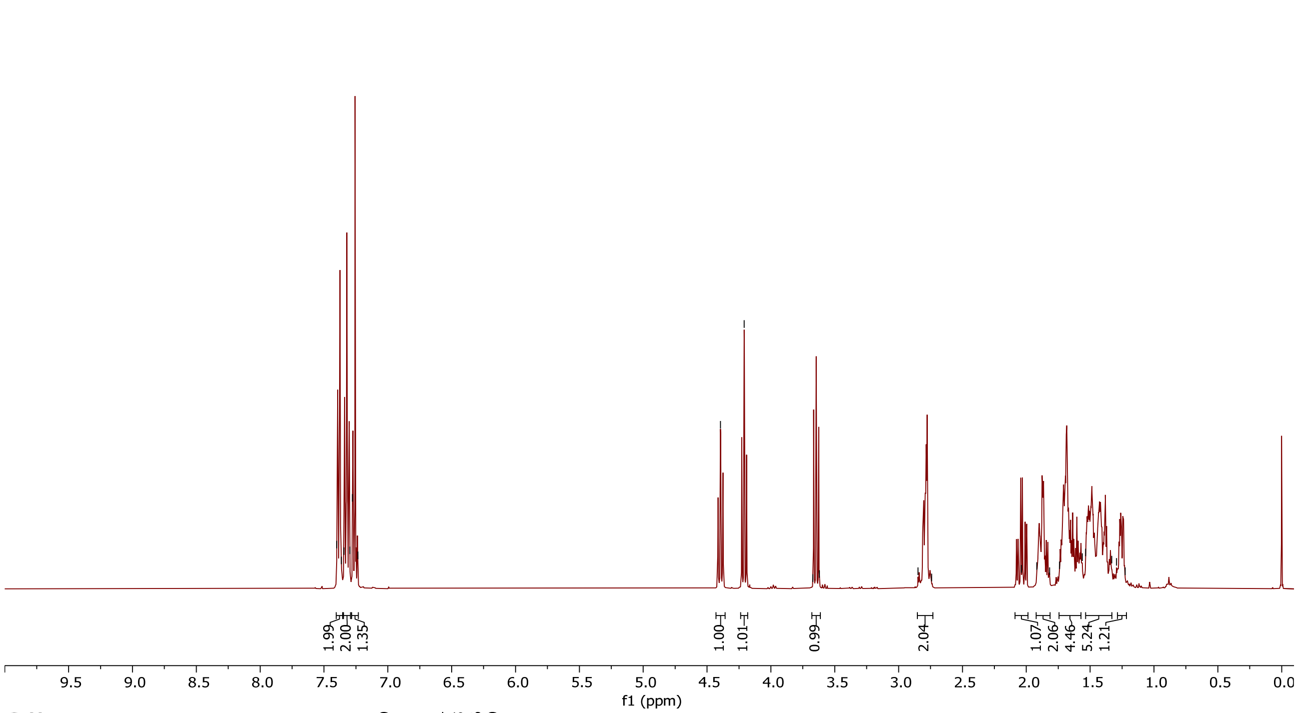

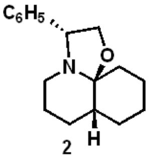

2

100.6 MHz, $CDCl_3$

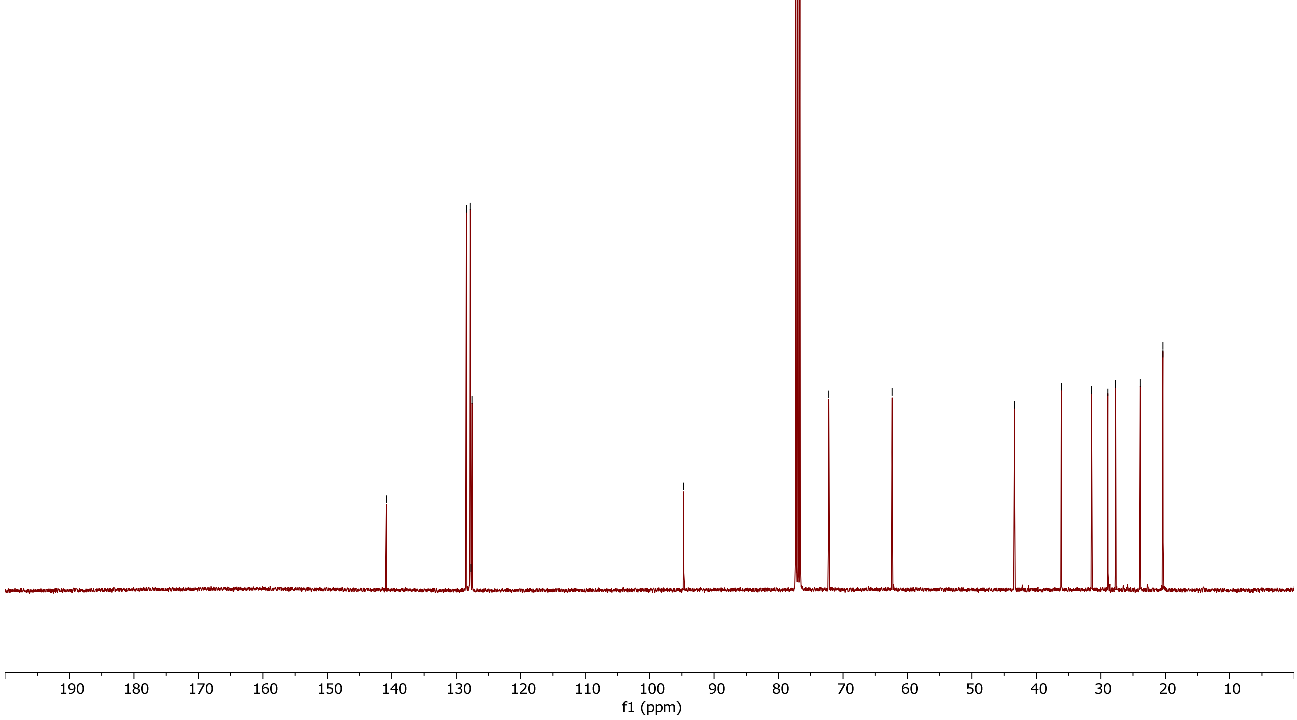

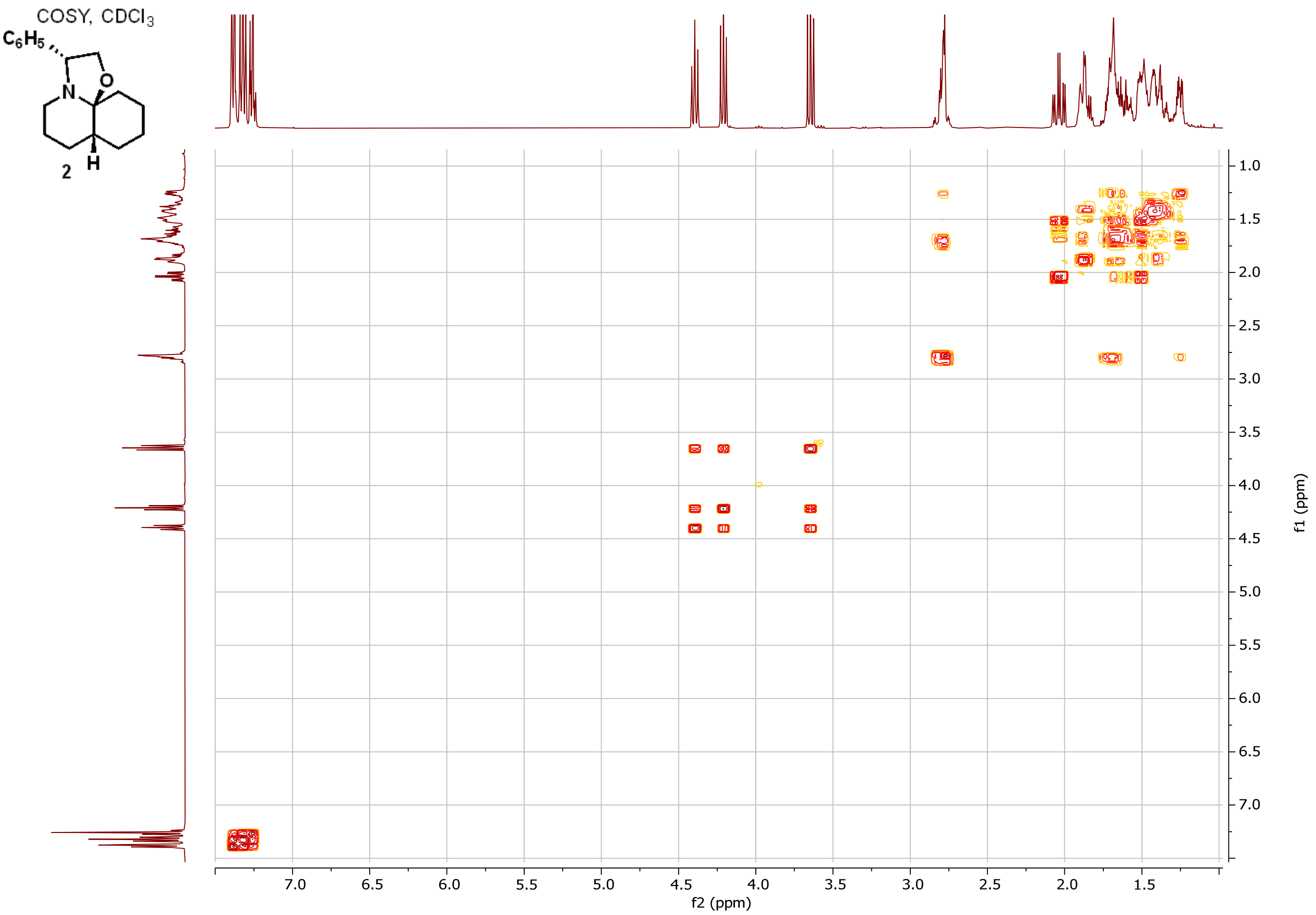
COSY, CDCl3
C6H5
N
O
2
H
f2 (ppm)
f1 (ppm)

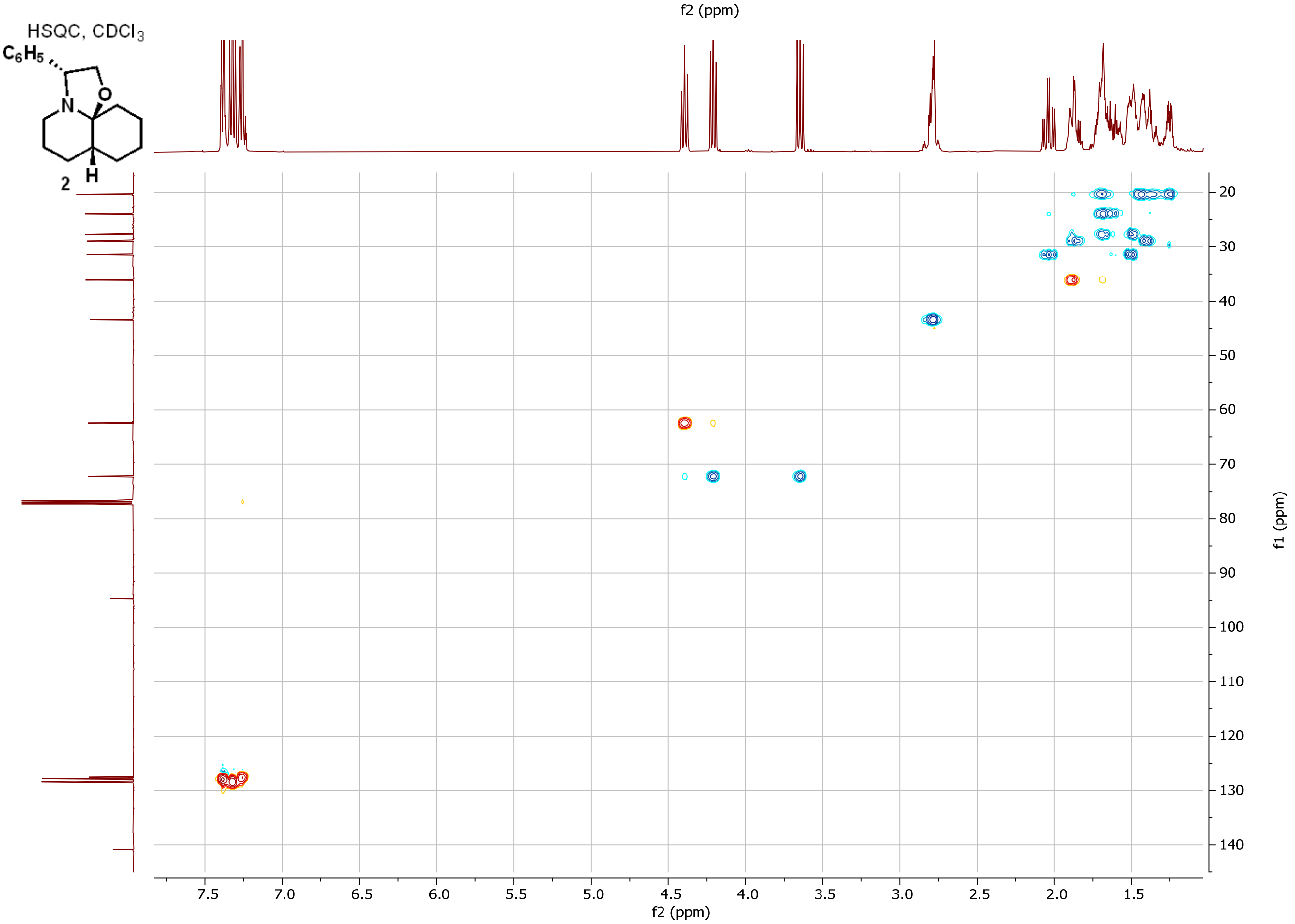
HSQC, CDCl3
C6H5
N
O
2
H
f2 (ppm)
f1 (ppm)

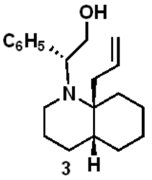

3

400 MHz, $CDCl_3$

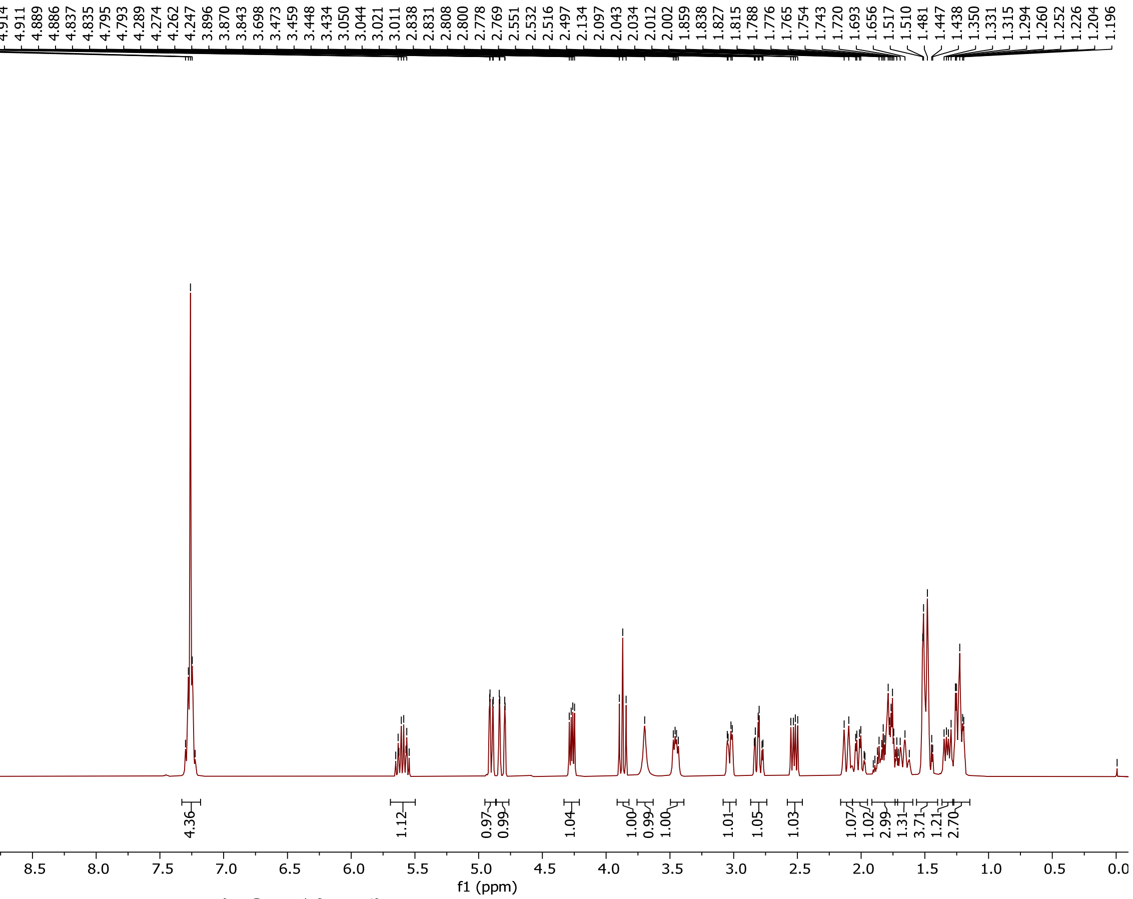

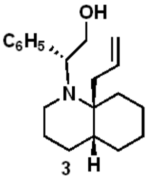

3

100.6 MHz, $CDCl_3$

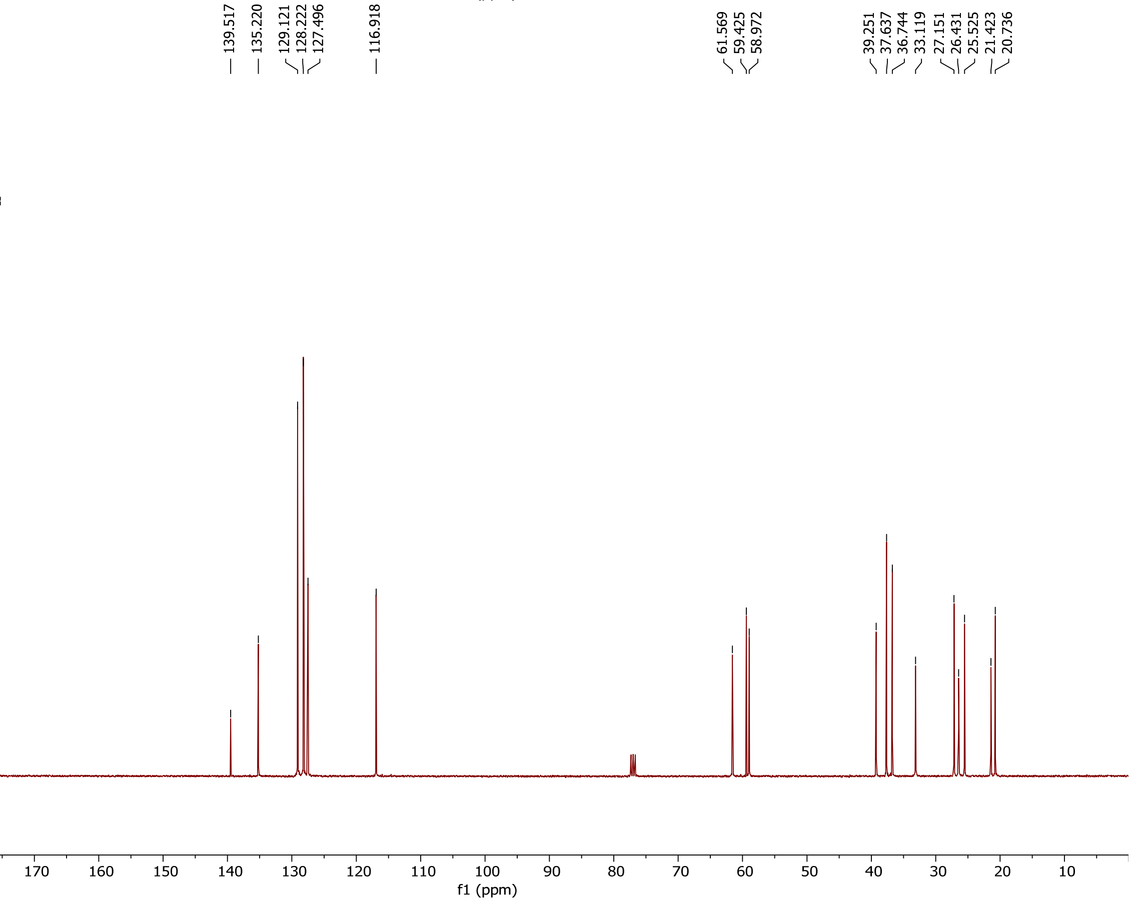

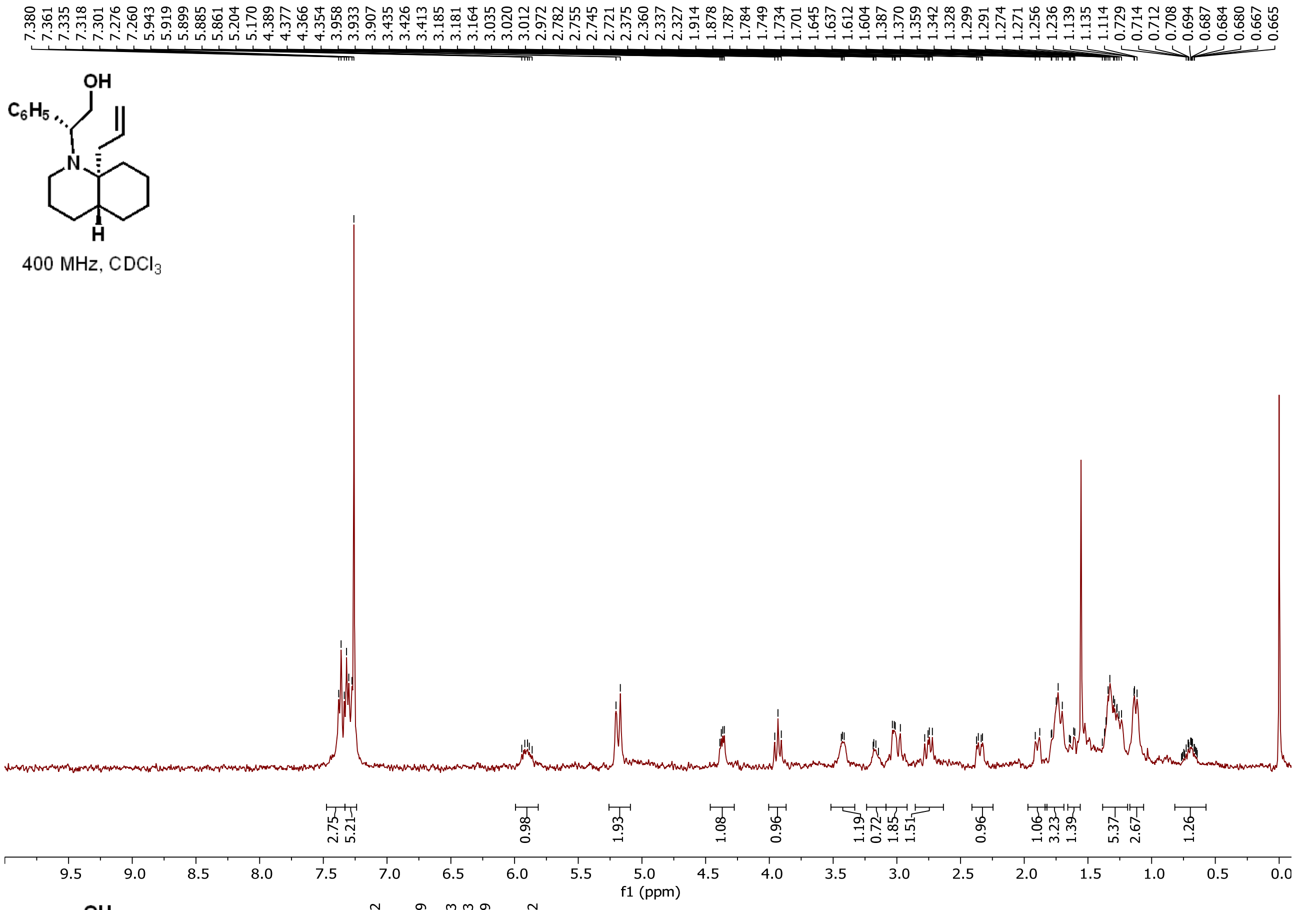

OH
C_6H_5
N
H
400 MHz, $CDCl_3$
7.380 7.361 7.335 7.318 7.301 7.276 7.260 5.943 5.919 5.899 5.885 5.861 5.204 5.170 4.389 4.377 4.366 4.354 3.958 3.933 3.907 3.435 3.426 3.413 3.185 3.181 3.164 3.035 3.020 3.012 2.972 2.782 2.755 2.745 2.721 2.375 2.360 2.337 2.327 1.914 1.878 1.787 1.784 1.749 1.734 1.701 1.645 1.637 1.612 1.604 1.387 1.370 1.359 1.342 1.328 1.299 1.291 1.274 1.271 1.256 1.236 1.139 1.135 1.114 0.729 0.714 0.712 0.708 0.694 0.687 0.684 0.680 0.667 0.665
2.75 5.21 0.98 1.93 1.08 0.96 1.19 0.72 1.85 1.51 0.96 1.06 3.23 1.39 5.37 2.67 1.26
9.5 9.0 8.5 8.0 7.5 7.0 6.5 6.0 5.5 5.0 4.5 4.0 3.5 3.0 2.5 2.0 1.5 1.0 0.5 0.0
f1 (ppm)

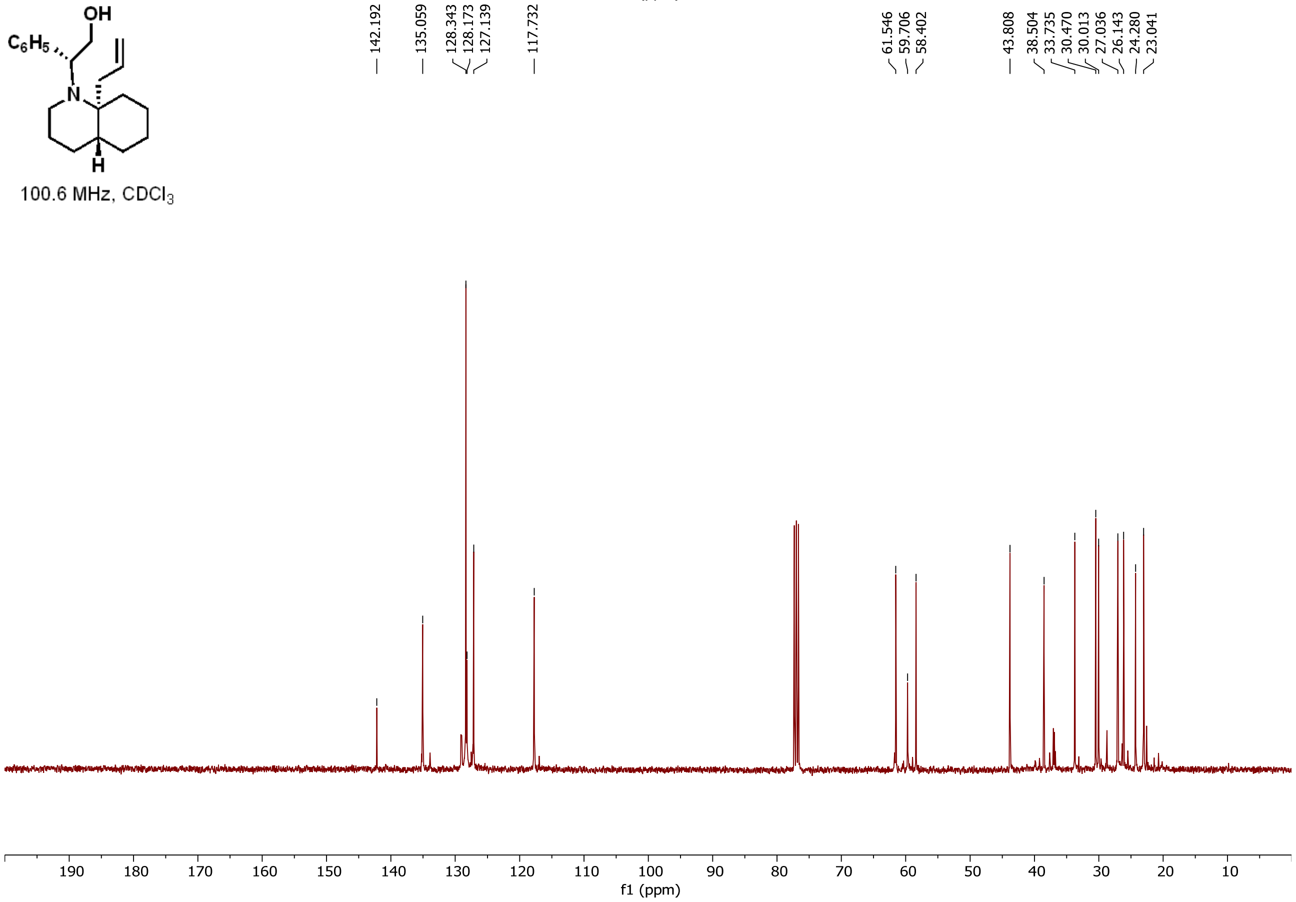

OH
C_6H_5
N
H
100.6 MHz, $CDCl_3$
142.192 135.059 128.343 128.173 127.139 117.732 61.546 59.706 58.402 43.808 38.504 33.735 30.470 30.013 27.036 26.143 24.280 23.041
190 180 170 160 150 140 130 120 110 100 90 80 70 60 50 40 30 20 10
f1 (ppm)

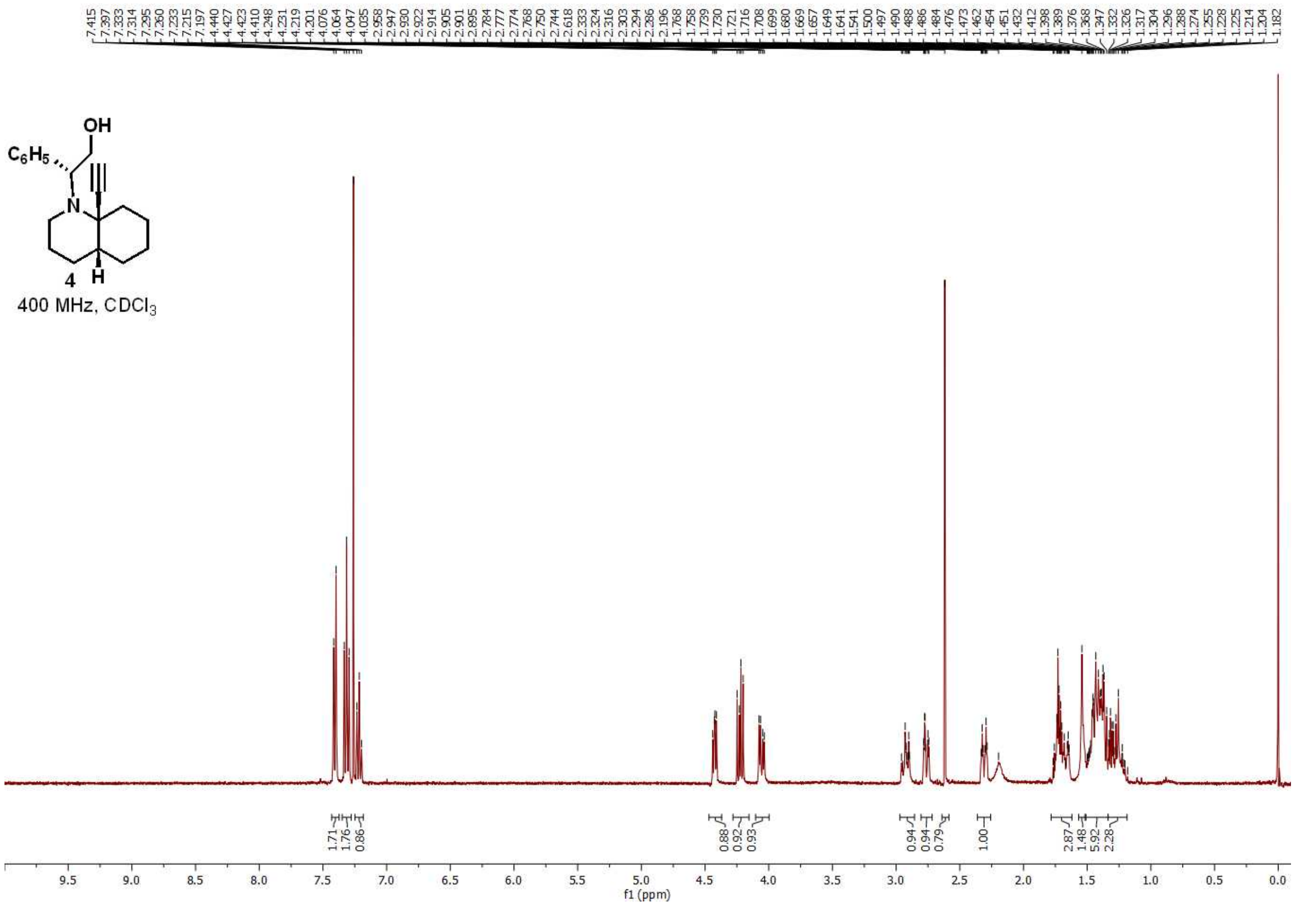
7.415 7.397 7.333 7.314 7.295 7.260 7.233 7.215 7.197 4.440 4.427 4.423 4.410 4.248 4.231 4.219 4.201 4.076 4.064 4.047 4.035 2.958 2.947 2.930 2.922 2.914 2.905 2.901 2.895 2.784 2.777 2.774 2.768 2.750 2.744 2.618 2.333 2.324 2.316 2.303 2.294 2.286 2.196 1.768 1.758 1.739 1.730 1.721 1.716 1.708 1.699 1.680 1.669 1.657 1.649 1.641 1.541 1.500 1.497 1.490 1.488 1.486 1.484 1.476 1.473 1.462 1.454 1.451 1.432 1.412 1.398 1.389 1.376 1.368 1.347 1.332 1.326 1.317 1.304 1.296 1.288 1.274 1.255 1.228 1.225 1.214 1.204 1.182
OH
C_6H_5
N
H
4
400 MHz, $CDCl_3$
1.71 1.76 0.86
0.88 0.92 0.93
0.94 0.94 0.79
1.00
2.87 1.48 5.92 2.28
9.5 9.0 8.5 8.0 7.5 7.0 6.5 6.0 5.5 5.0 4.5 4.0 3.5 3.0 2.5 2.0 1.5 1.0 0.5 0.0
f1 (ppm)

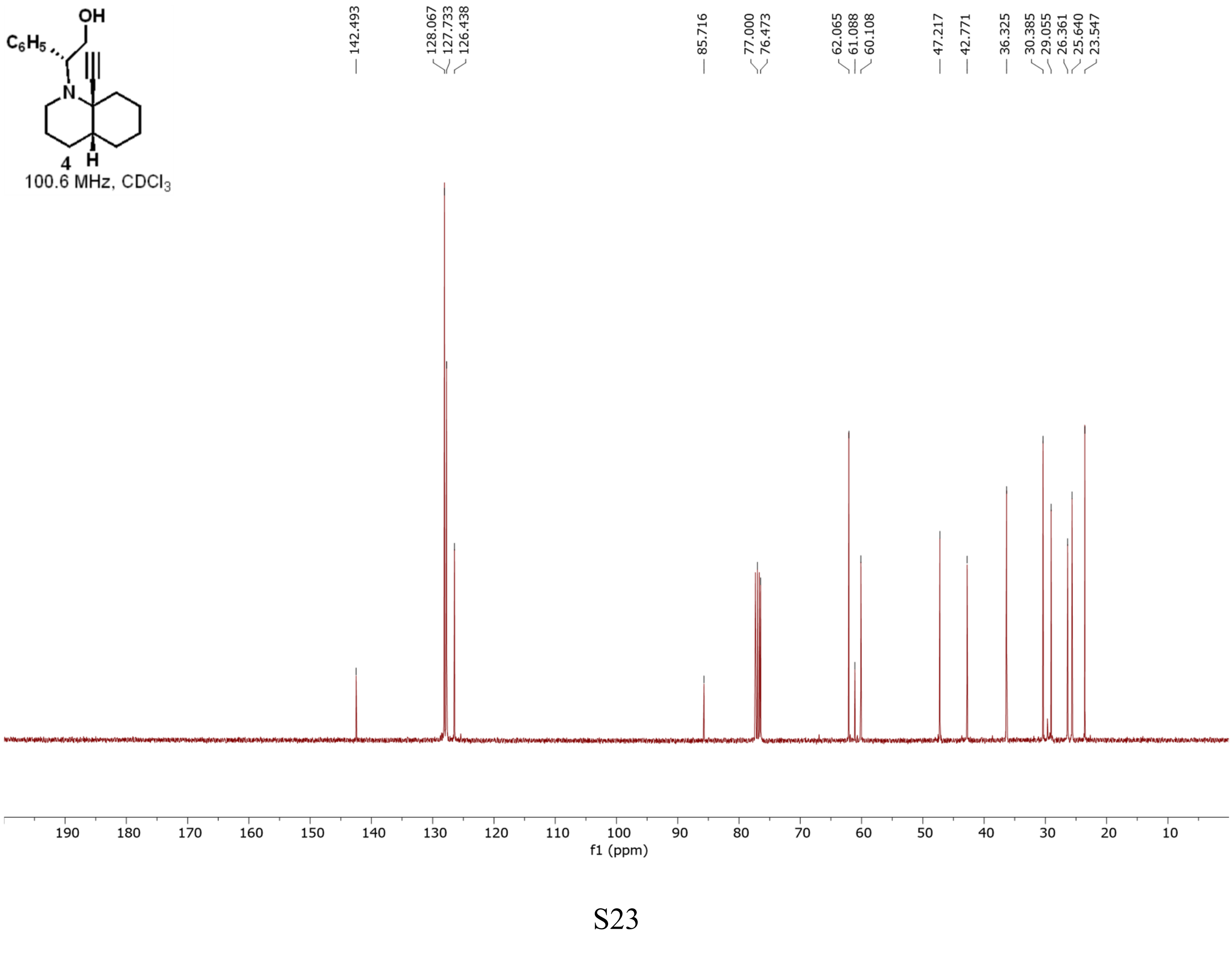
142.493
128.067 127.733 126.438
85.716
77.000 76.473
62.065 61.088 60.108
47.217
42.771
36.325
30.385 29.055 26.361 25.640 23.547
OH
C_6H_5
N
H
4
100.6 MHz, $CDCl_3$
190 180 170 160 150 140 130 120 110 100 90 80 70 60 50 40 30 20 10
f1 (ppm)

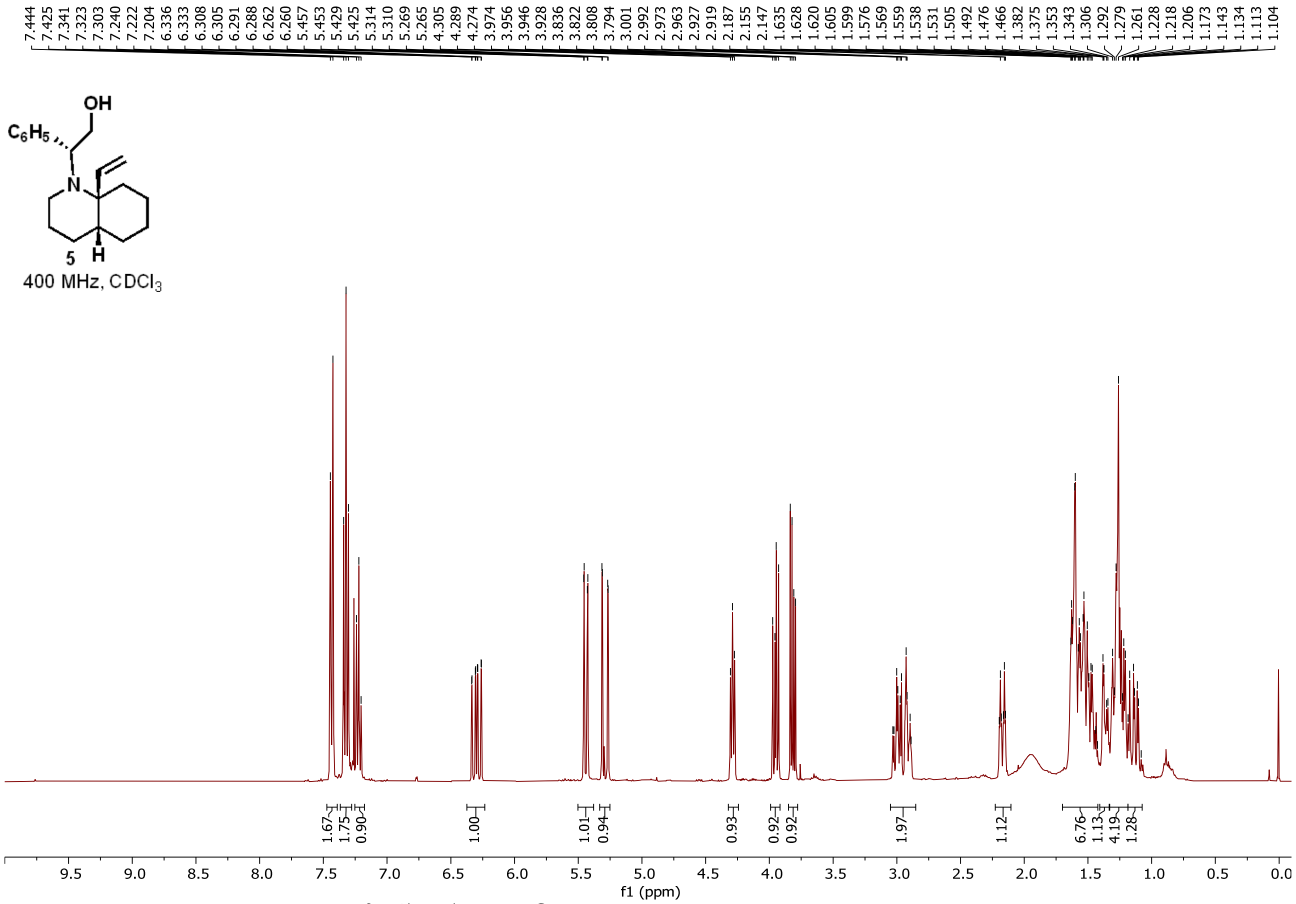
7.444 7.425 7.341 7.323 7.303 7.240 7.222 7.204 6.336 6.333 6.308 6.305 6.291 6.288 6.262 6.260 5.457 5.453 5.429 5.425 5.314 5.310 5.269 5.265 4.305 4.289 4.274 3.974 3.956 3.946 3.928 3.836 3.822 3.808 3.794 3.001 2.992 2.973 2.963 2.927 2.919 2.187 2.155 2.147 1.635 1.628 1.620 1.605 1.599 1.576 1.569 1.559 1.538 1.531 1.505 1.492 1.476 1.466 1.382 1.375 1.353 1.343 1.306 1.292 1.279 1.261 1.228 1.218 1.206 1.173 1.143 1.134 1.113 1.104
OH
C6H5
N
5
H
400 MHz, CDCl3
1.67 1.75 0.90 1.00 1.01 0.94 0.93 0.92 0.92 1.97 1.12 6.76 1.13 4.19 1.28
9.5 9.0 8.5 8.0 7.5 7.0 6.5 6.0 5.5 5.0 4.5 4.0 3.5 3.0 2.5 2.0 1.5 1.0 0.5 0.0
f1 (ppm)

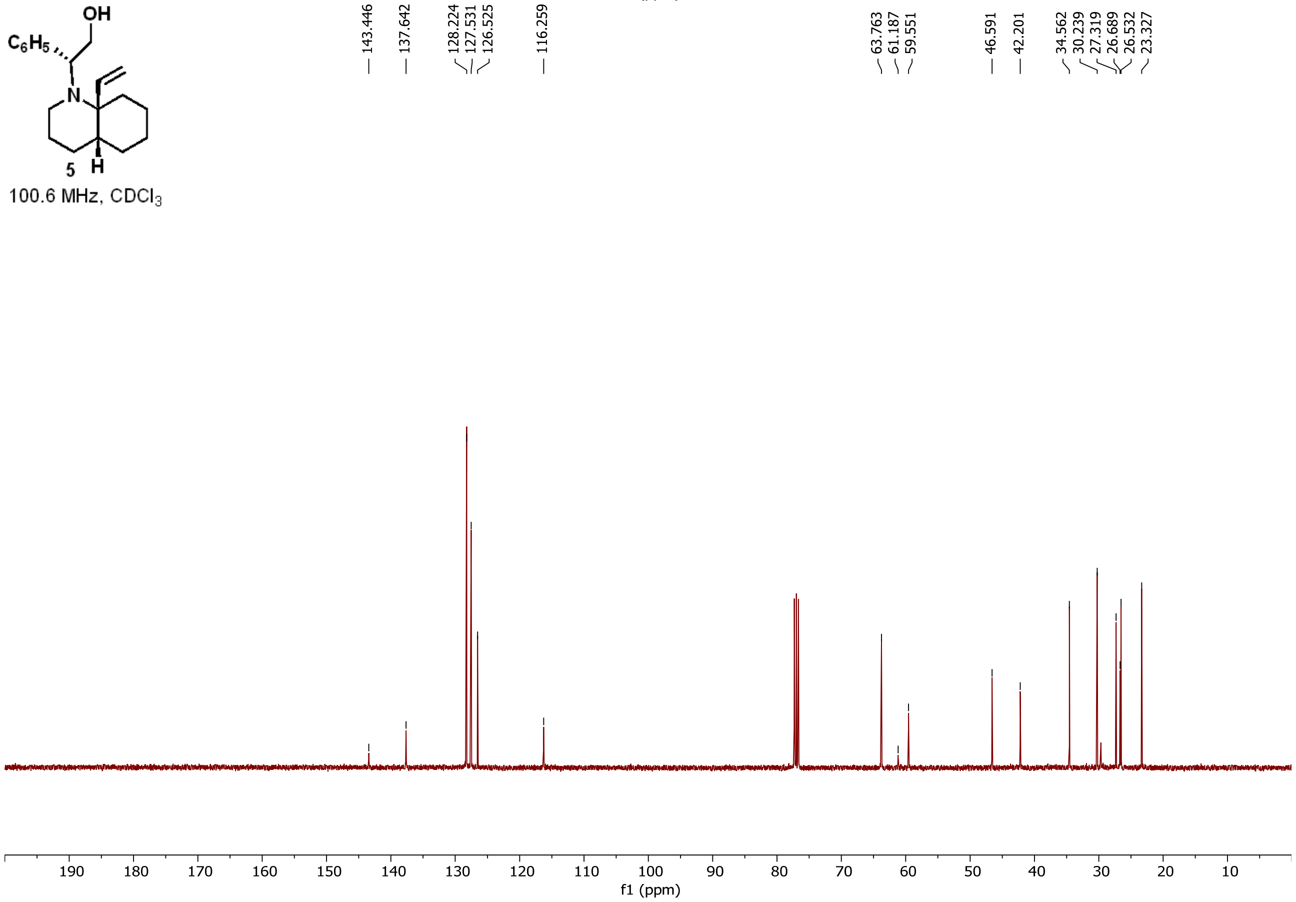
OH
C6H5
N
5
H
100.6 MHz, CDCl3
143.446 137.642 128.224 127.531 126.525 116.259 63.763 61.187 59.551 46.591 42.201 34.562 30.239 27.319 26.689 26.532 23.327
190 180 170 160 150 140 130 120 110 100 90 80 70 60 50 40 30 20 10
f1 (ppm)

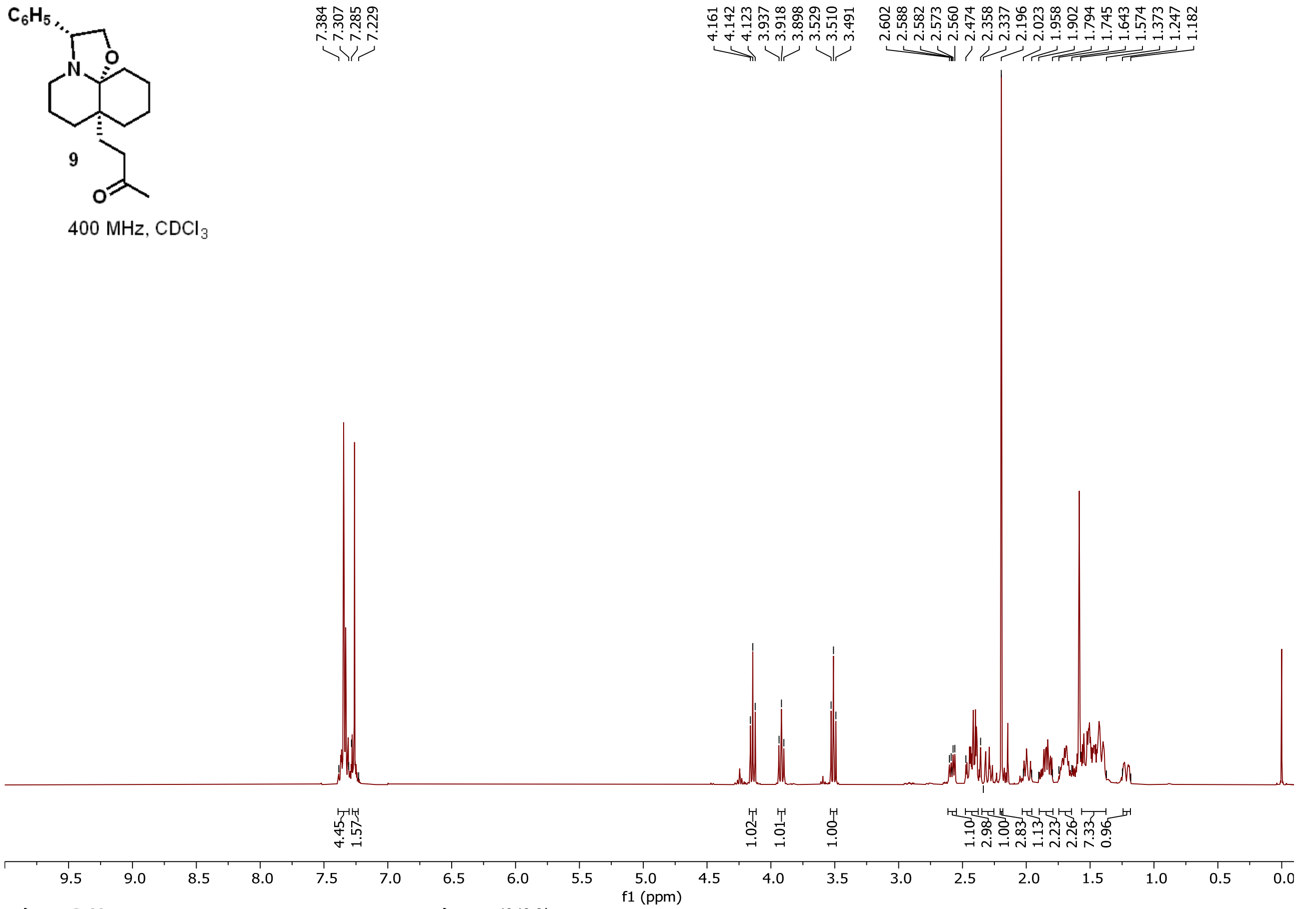
C6H5
N
O
9
400 MHz, CDCl3
7.384 7.307 7.285 7.229
4.161 4.142 4.123 3.937 3.918 3.898 3.529 3.510 3.491
2.602 2.588 2.582 2.573 2.560 2.474 2.358 2.337 2.196 2.023 1.958 1.902 1.794 1.745 1.643 1.574 1.373 1.247 1.182
4.45 1.57
1.02 1.01 1.00
1.10 2.98 1.00 2.83 1.13 2.23 2.26 7.33 0.96
9.5 9.0 8.5 8.0 7.5 7.0 6.5 6.0 5.5 5.0 4.5 4.0 3.5 3.0 2.5 2.0 1.5 1.0 0.5 0.0
f1 (ppm)

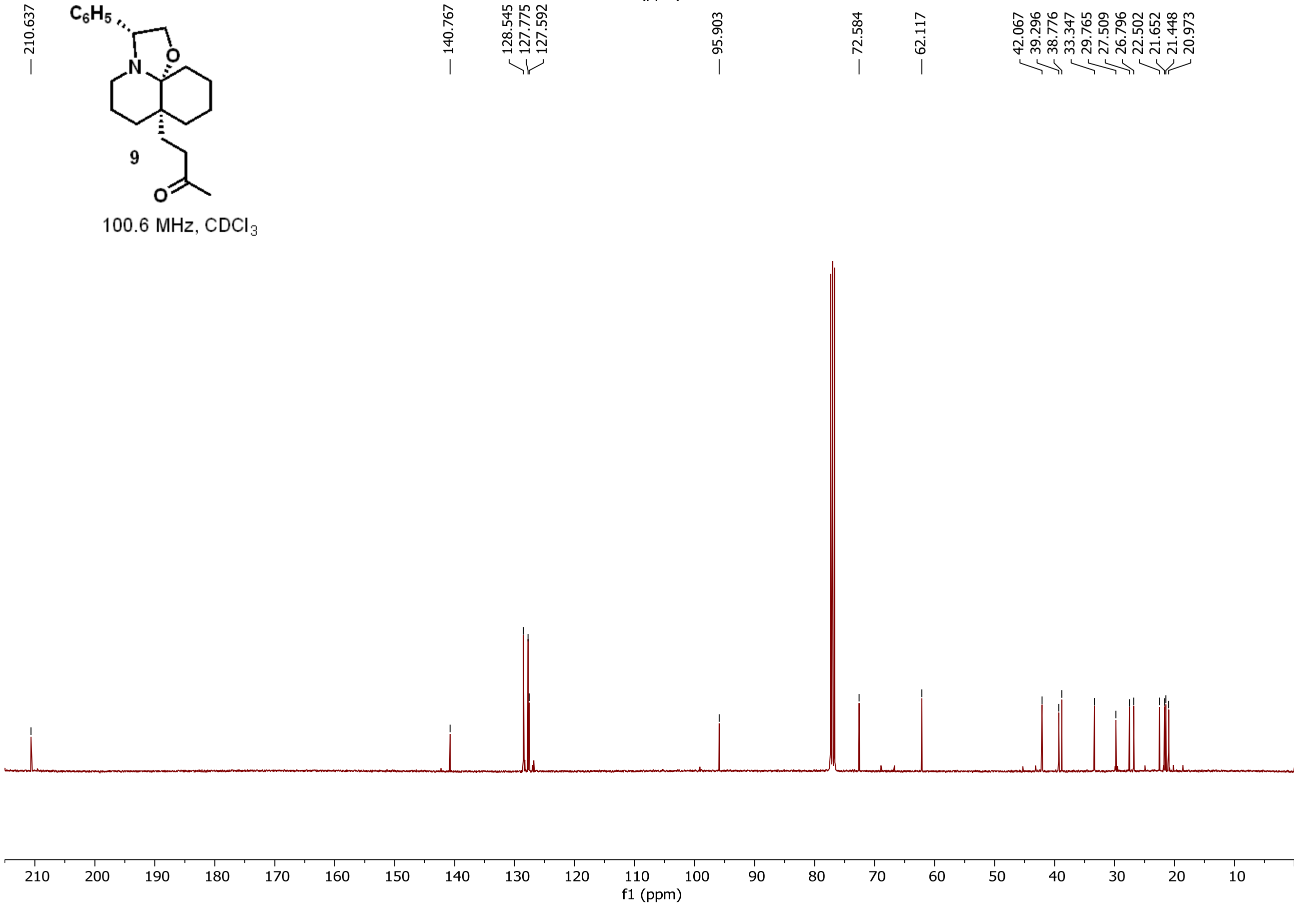
C6H5
N
O
9
100.6 MHz, CDCl3
210.637
140.767
128.545 127.775 127.592
95.903
72.584
62.117
42.067 39.296 38.776 33.347 29.765 27.509 26.796 22.502 21.652 21.448 20.973
210 200 190 180 170 160 150 140 130 120 110 100 90 80 70 60 50 40 30 20 10
f1 (ppm)

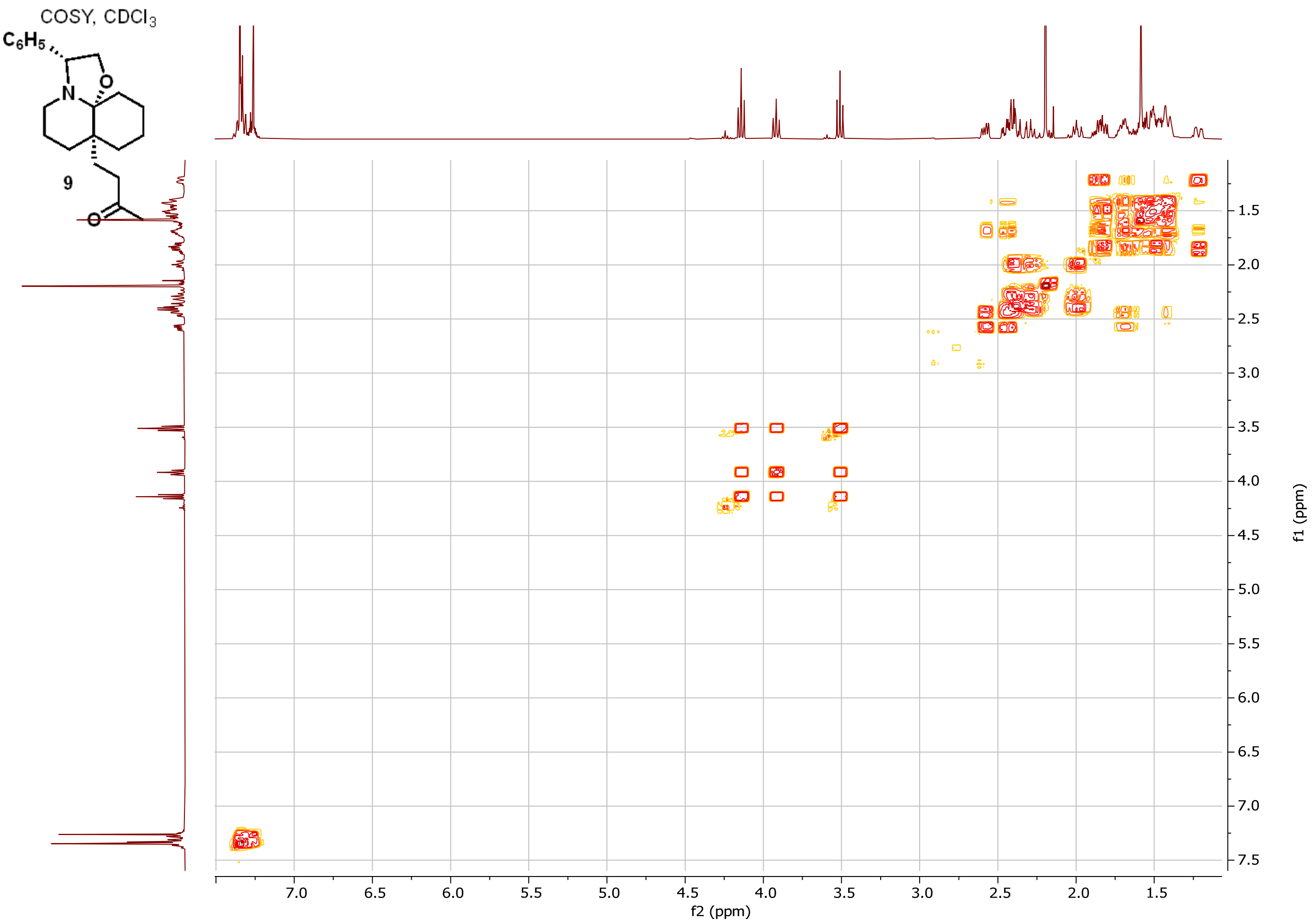
COSY, CDCl3
C6H5
N
O
9
O
f2 (ppm)
f1 (ppm)

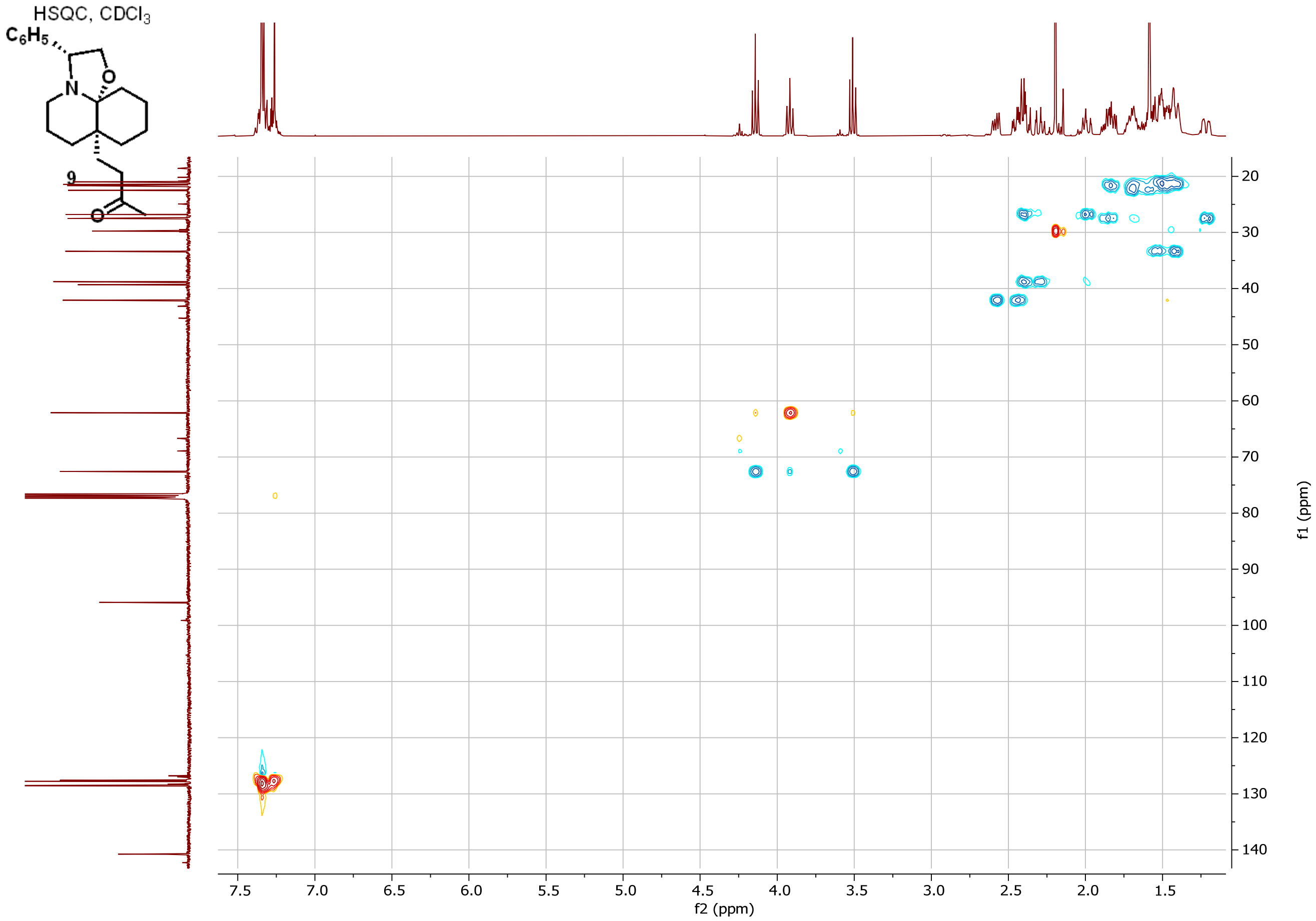
HSQC, CDCl3
C6H5
N
O
9
O
f2 (ppm)
f1 (ppm)

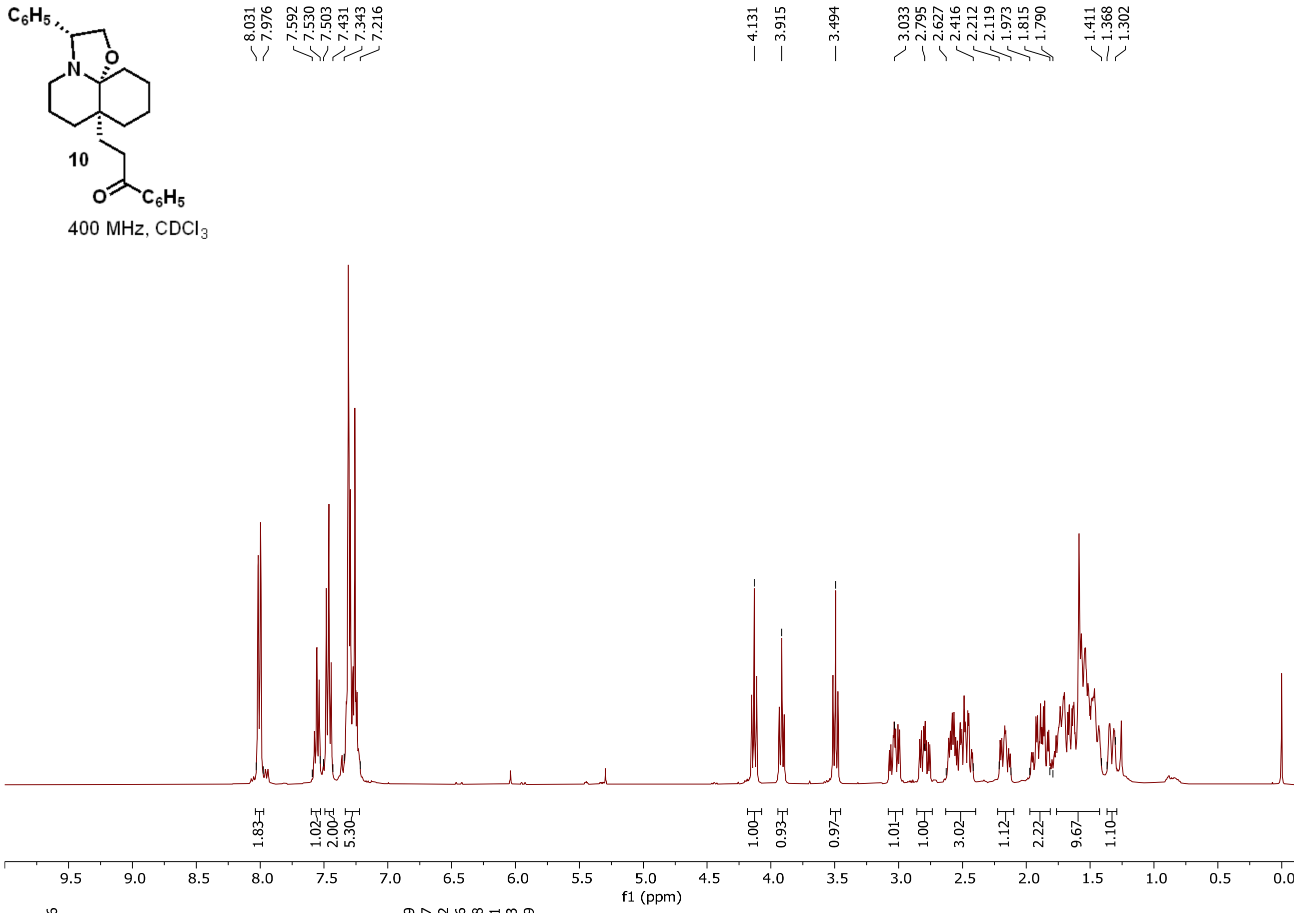
C6H5
O
N
10
O
C6H5
400 MHz, CDCl3
8.031
7.976
7.592
7.530
7.503
7.431
7.343
7.216
4.131
3.915
3.494
3.033
2.795
2.627
2.416
2.212
2.119
1.973
1.815
1.790
1.411
1.368
1.302
1.83
1.02
2.00
5.30
1.00
0.93
0.97
1.01
1.00
3.02
1.12
2.22
9.67
1.10
9.5 9.0 8.5 8.0 7.5 7.0 6.5 6.0 5.5 5.0 4.5 4.0 3.5 3.0 2.5 2.0 1.5 1.0 0.5 0.0
f1 (ppm)

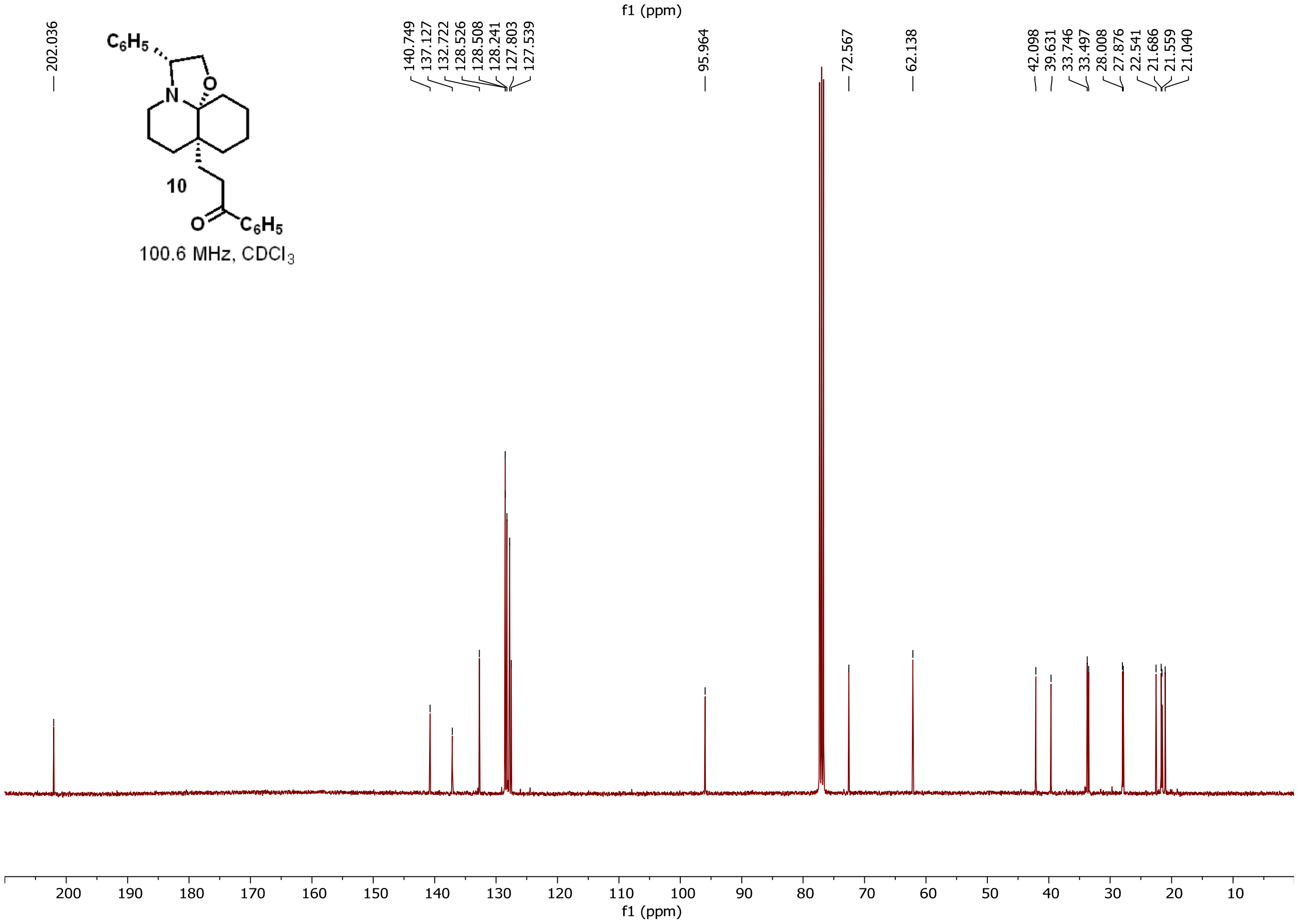
202.036
C6H5
O
N
10
O
C6H5
100.6 MHz, CDCl3
140.749
137.127
132.722
128.526
128.508
128.241
127.803
127.539
95.964
72.567
62.138
42.098
39.631
33.746
33.497
28.008
27.876
22.541
21.686
21.559
21.040
200 190 180 170 160 150 140 130 120 110 100 90 80 70 60 50 40 30 20 10
f1 (ppm)

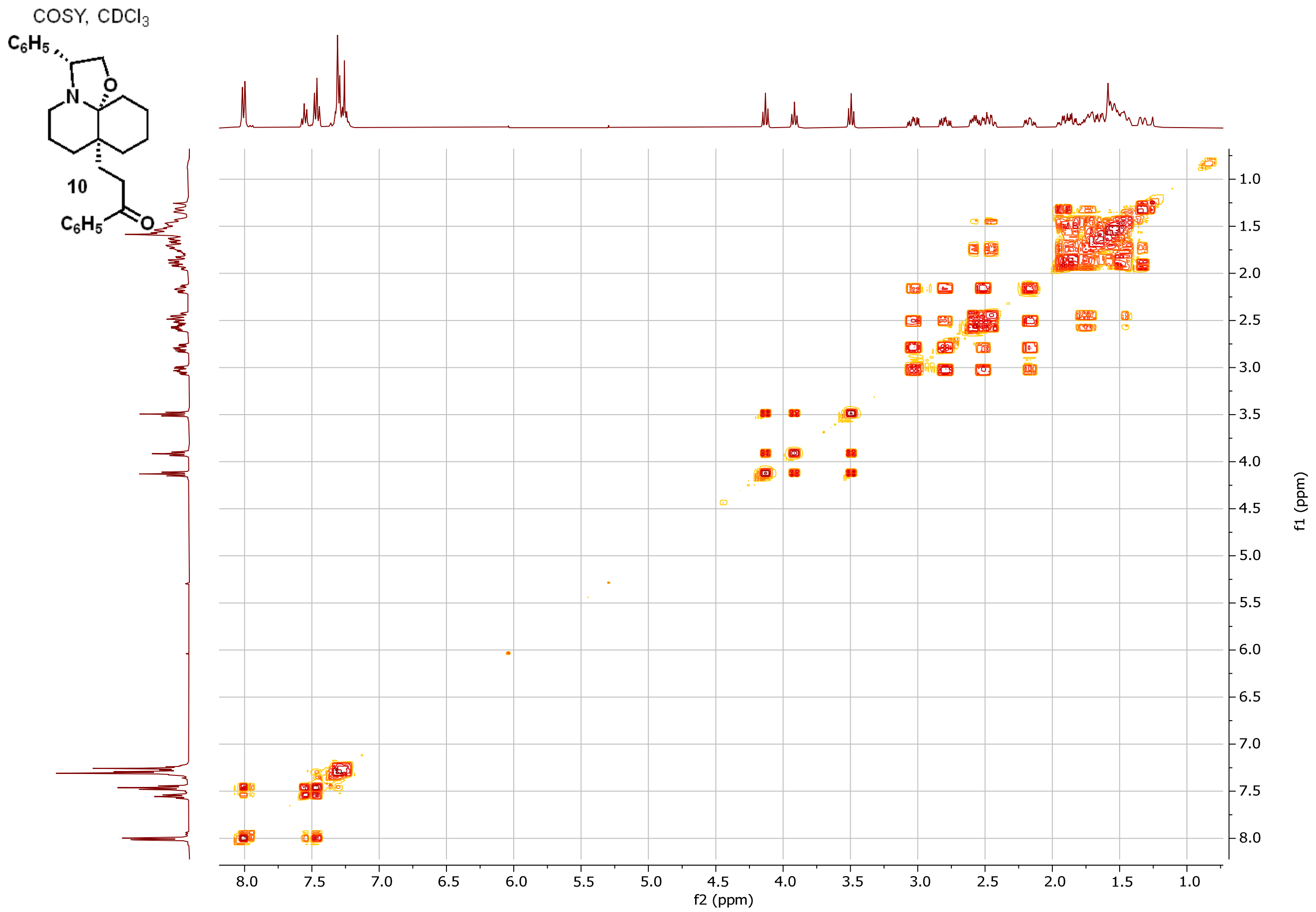
COSY, CDCl3
C6H5
N
O
10
C6H5
O
f2 (ppm)
f1 (ppm)

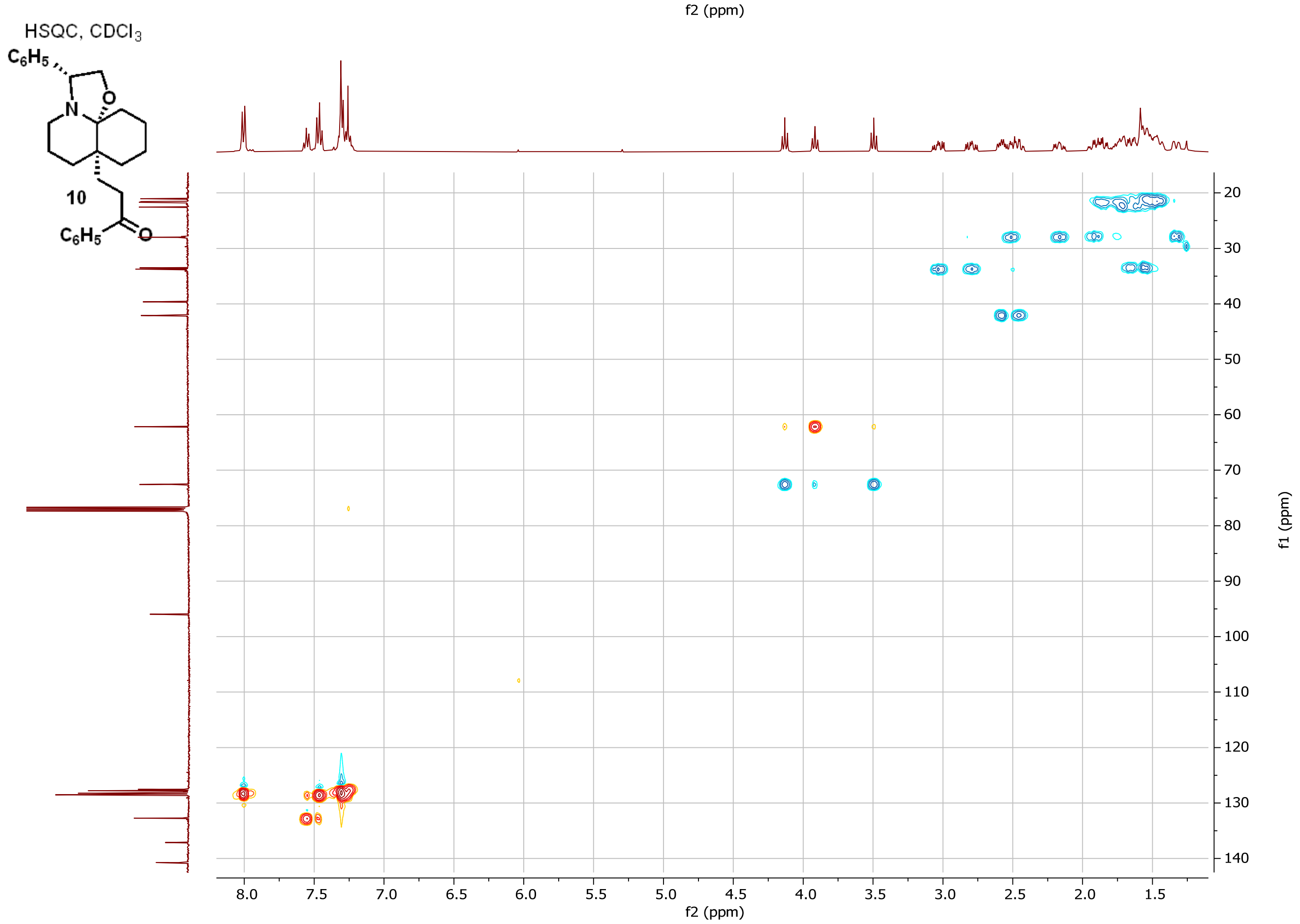
HSQC, CDCl3
C6H5
N
O
10
C6H5
O
f2 (ppm)
f1 (ppm)

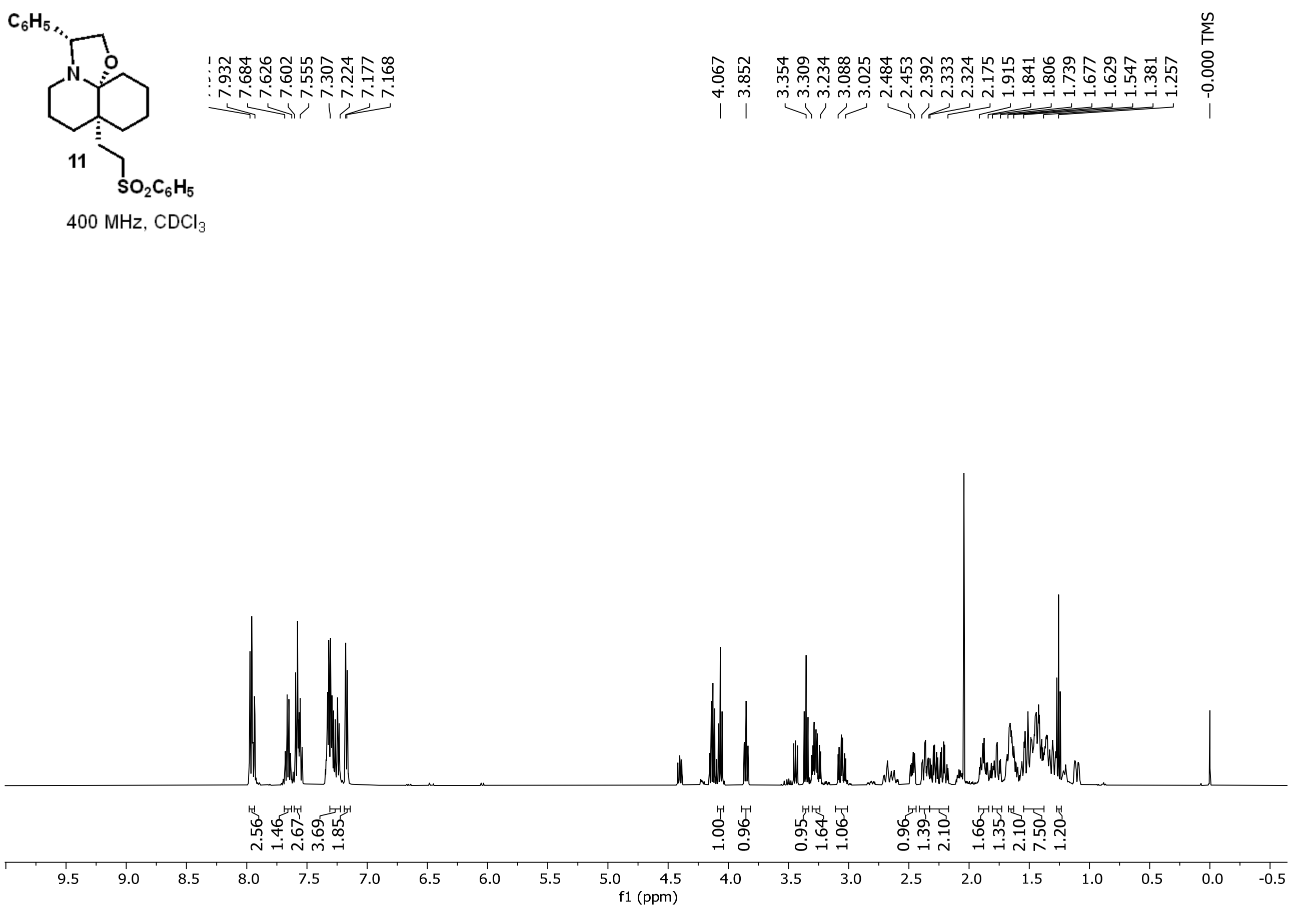

11

400 MHz, $CDCl_3$

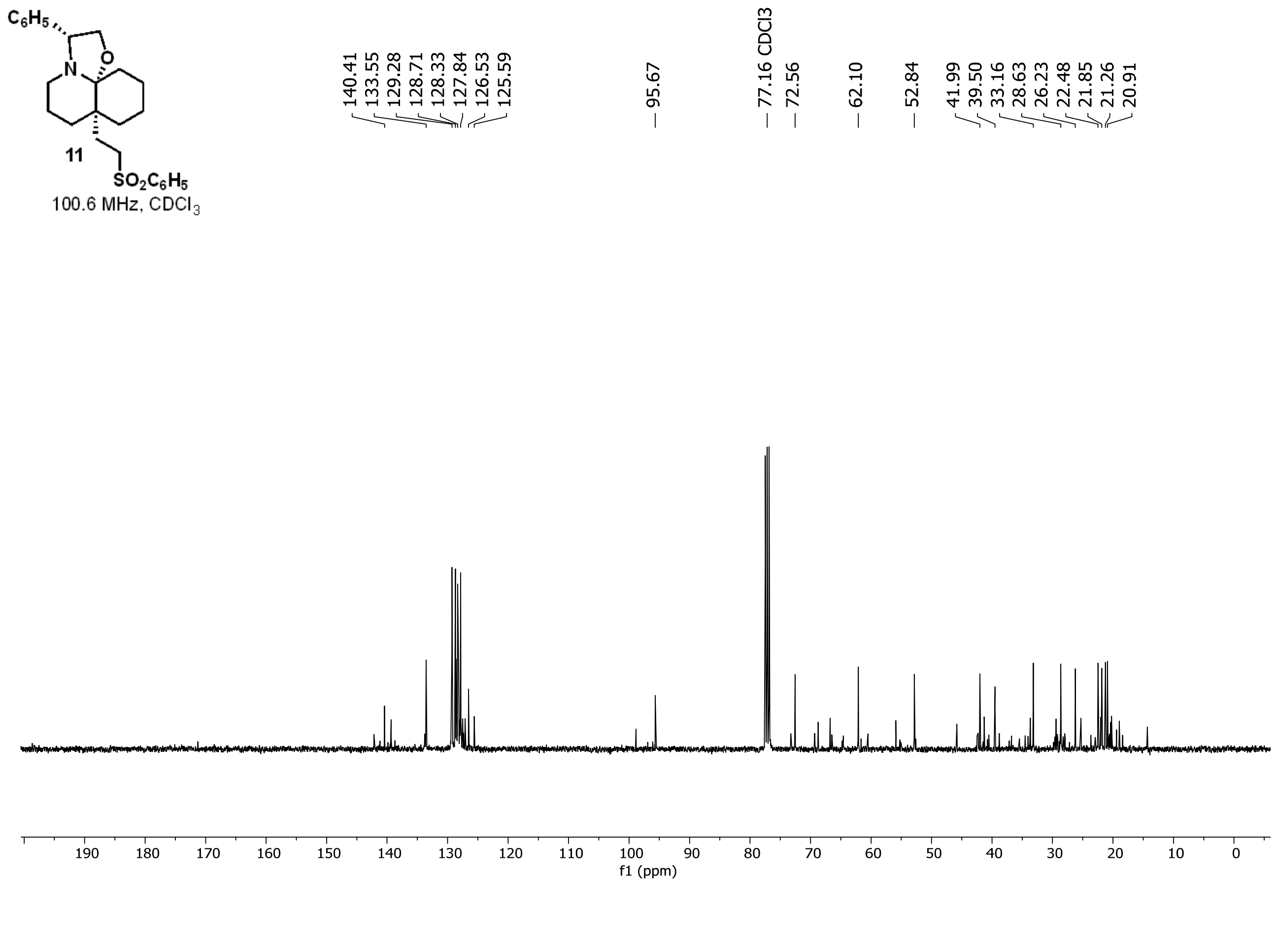

11

100.6 MHz, $CDCl_3$

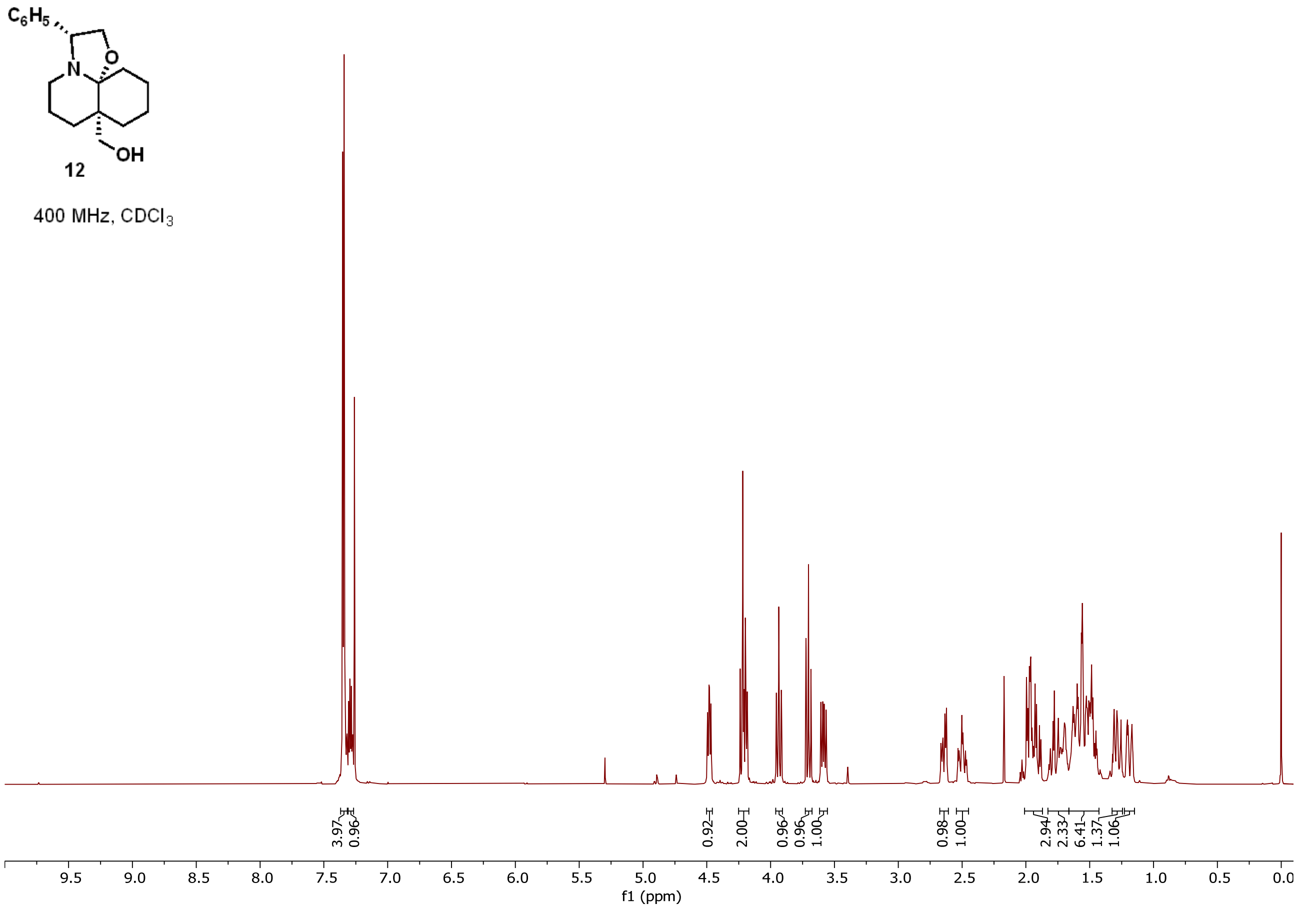

C_6H_5
N
O
OH
12
400 MHz, $CDCl_3$
3.97
0.96
0.92
2.00
0.96
0.96
1.00
0.98
1.00
2.94
2.33
6.41
1.37
1.06
9.5
9.0
8.5
8.0
7.5
7.0
6.5
6.0
5.5
5.0
4.5
4.0
3.5
3.0
2.5
2.0
1.5
1.0
0.5
0.0
f1 (ppm)

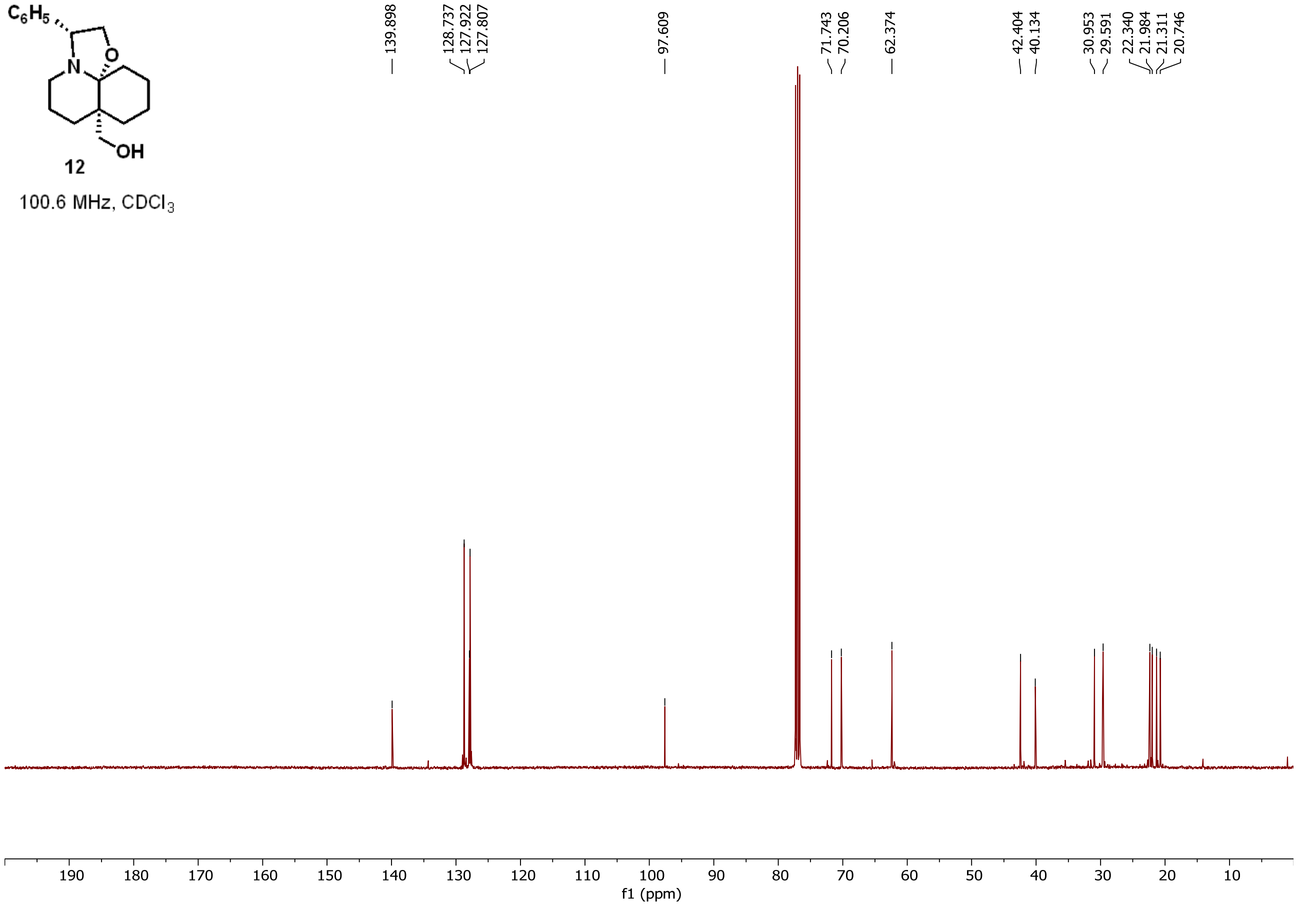

C_6H_5
N
O
OH
12
100.6 MHz, $CDCl_3$
139.898
128.737
127.922
127.807
97.609
71.743
70.206
62.374
42.404
40.134
30.953
29.591
22.340
21.984
21.311
20.746
190
180
170
160
150
140
130
120
110
100
90
80
70
60
50
40
30
20
10
f1 (ppm)

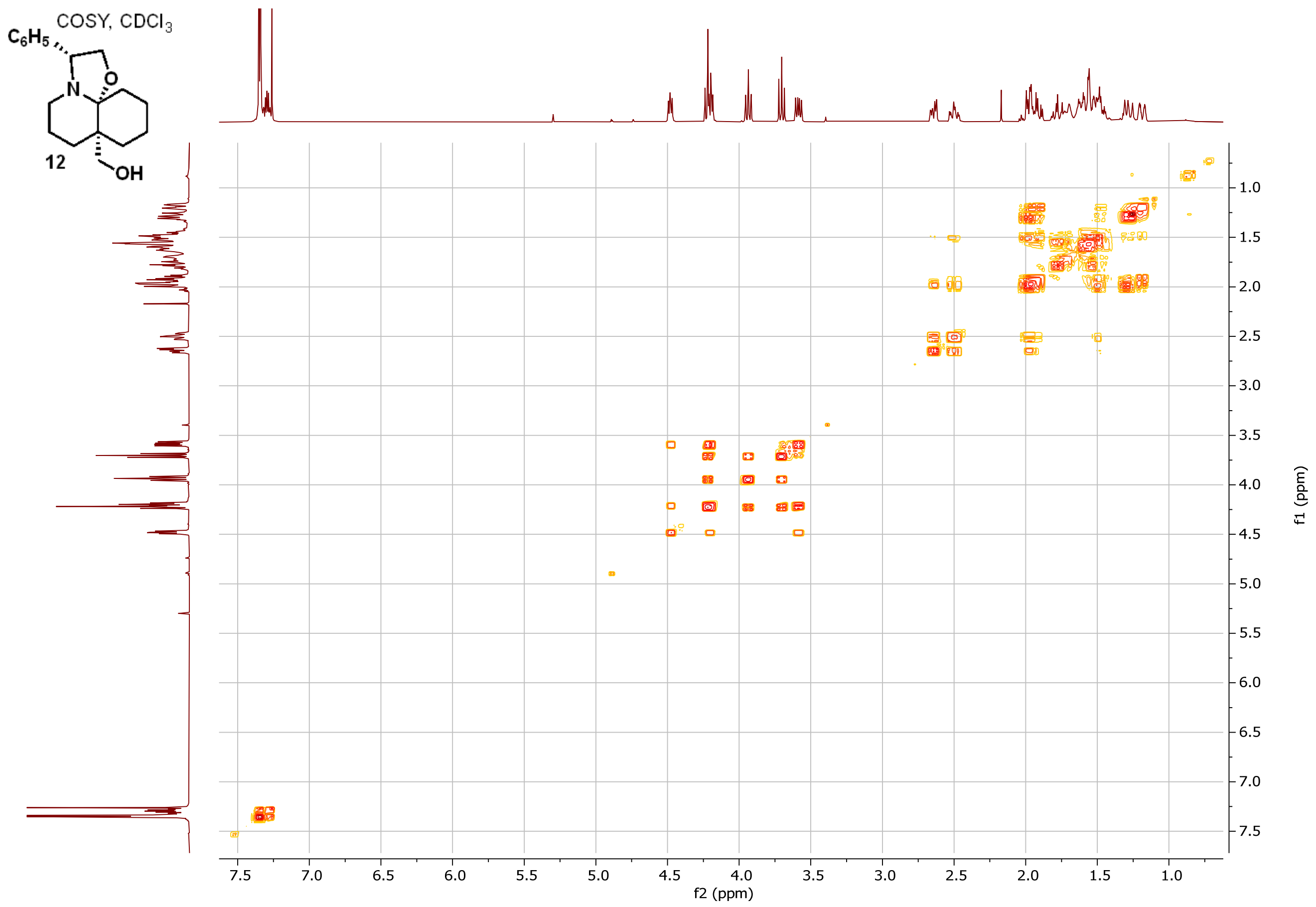
COSY, $CDCl_3$
C_6H_5
N
O
12
OH
f2 (ppm)
f1 (ppm)

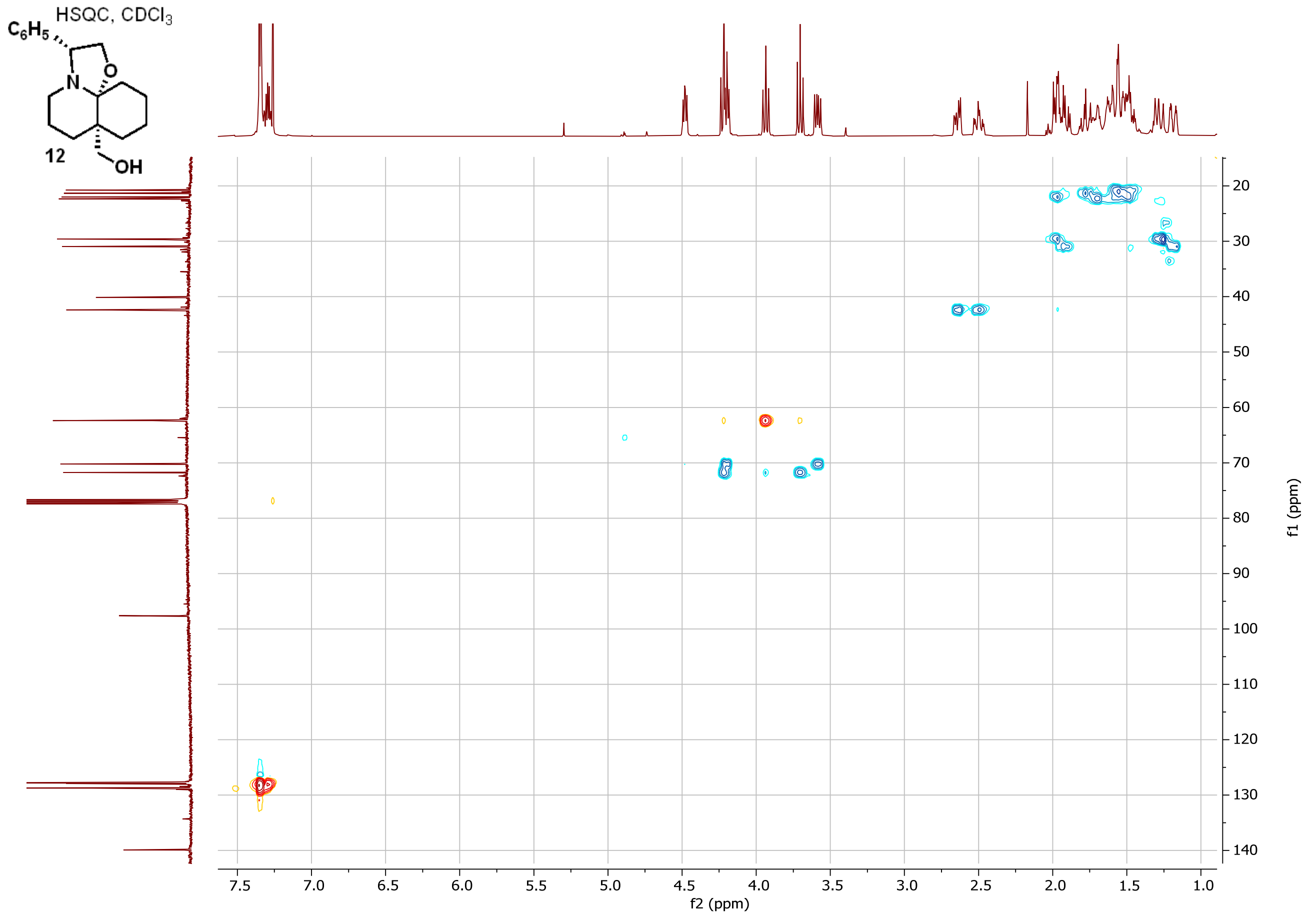
HSQC, $CDCl_3$
C_6H_5
N
O
12
OH
f2 (ppm)
f1 (ppm)

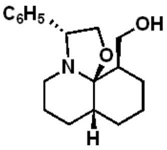

13

400 MHz, $CDCl_3$

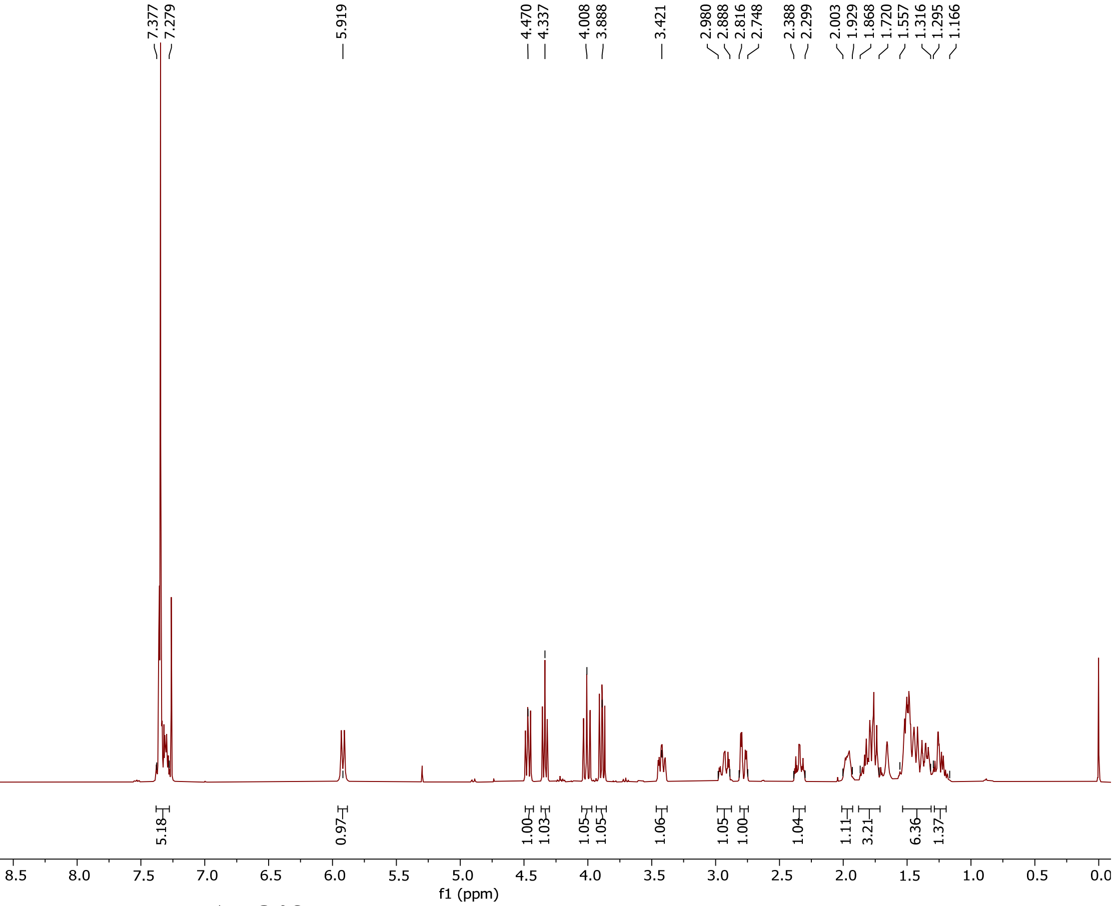

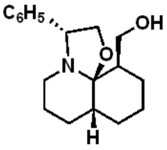

13

100.6 MHz, $CDCl_3$

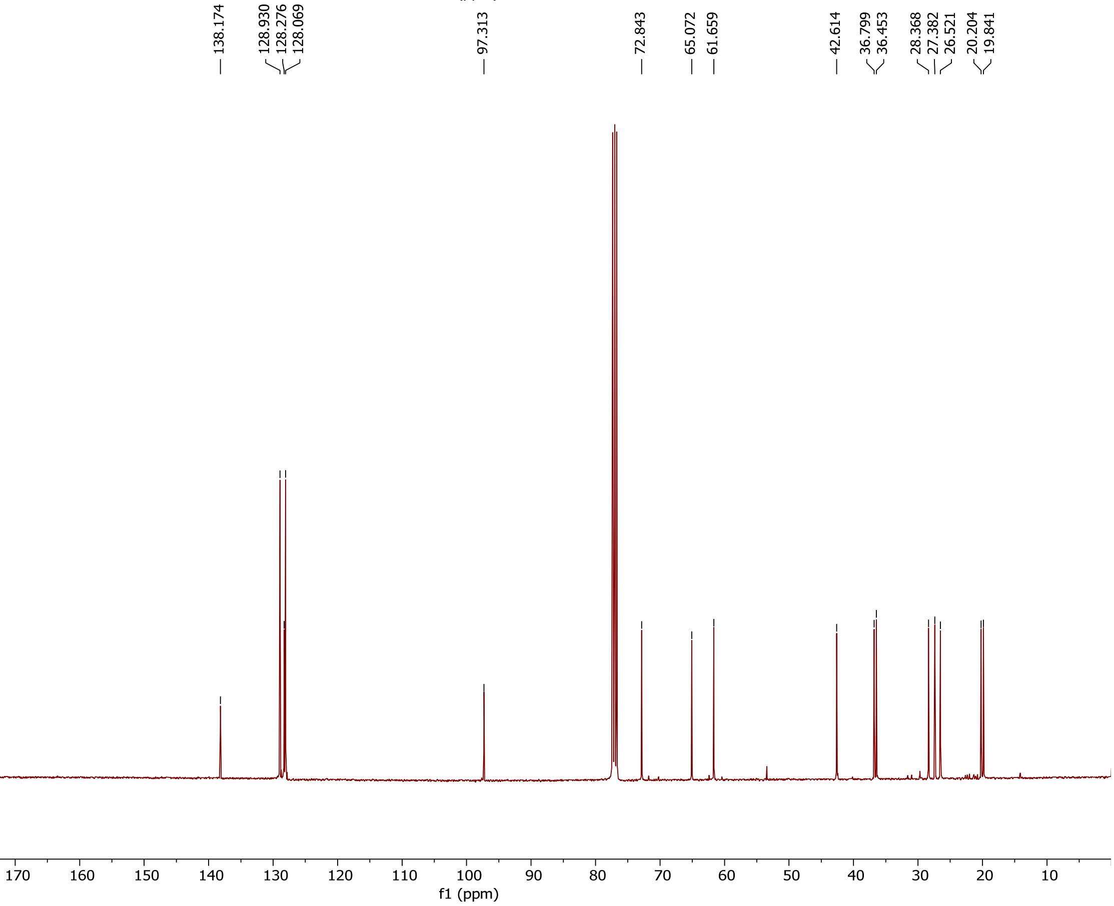

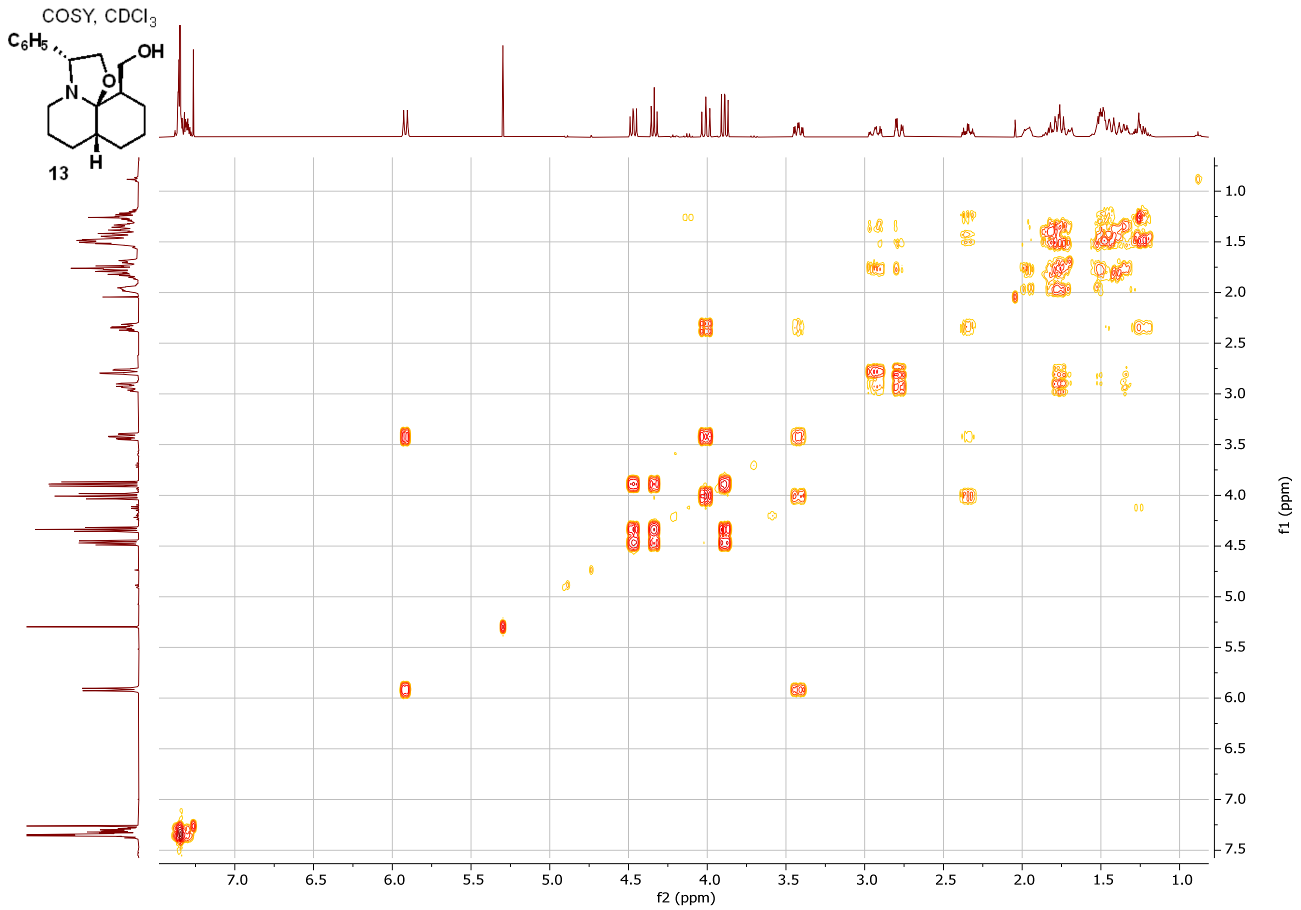
COSY, CDCl3
C6H5
OH
O
N
H
13
f2 (ppm)
f1 (ppm)

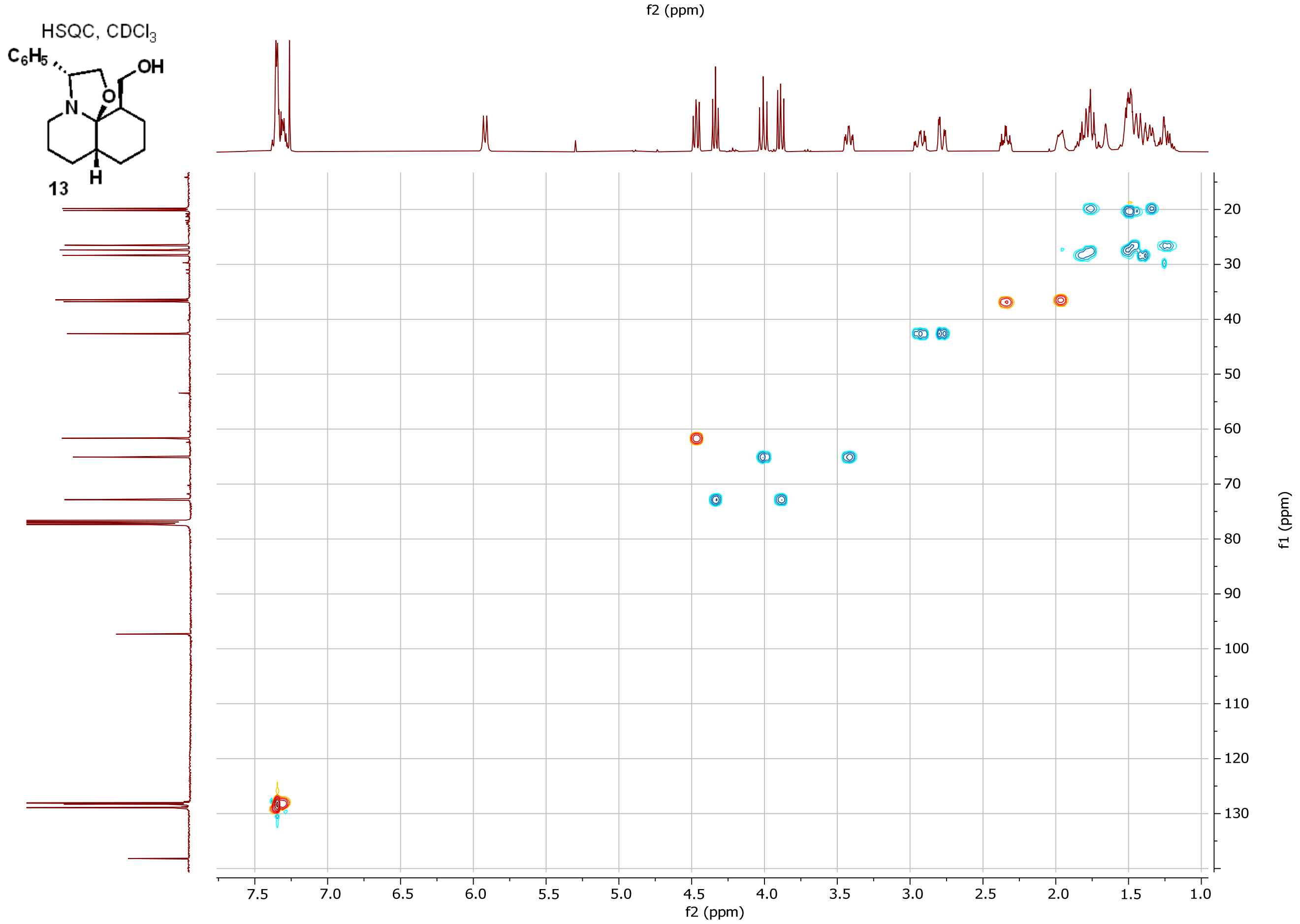
HSQC, CDCl3
C6H5
OH
O
N
H
13
f2 (ppm)
f1 (ppm)

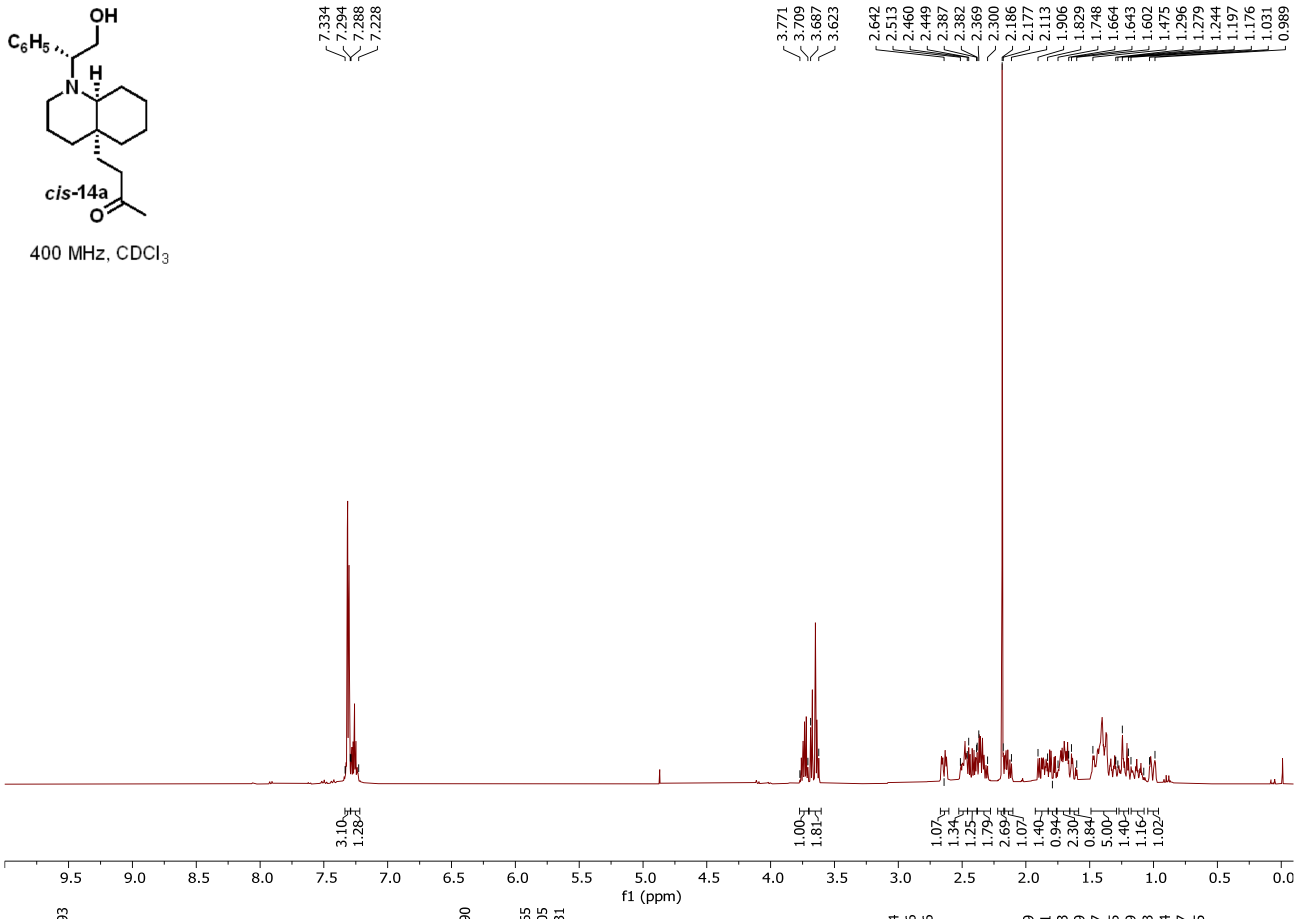

cis-14a
400 MHz, CDCl3
7.334
7.294
7.288
7.228
3.771
3.709
3.687
3.623
2.642
2.513
2.460
2.449
2.387
2.382
2.369
2.300
2.186
2.177
2.113
1.906
1.829
1.748
1.664
1.643
1.602
1.475
1.296
1.279
1.244
1.197
1.176
1.031
0.989
3.10
1.28
1.00
1.81
1.07
1.34
1.25
1.79
2.69
1.07
1.40
0.94
2.30
0.84
5.00
1.40
1.16
1.02
f1 (ppm)

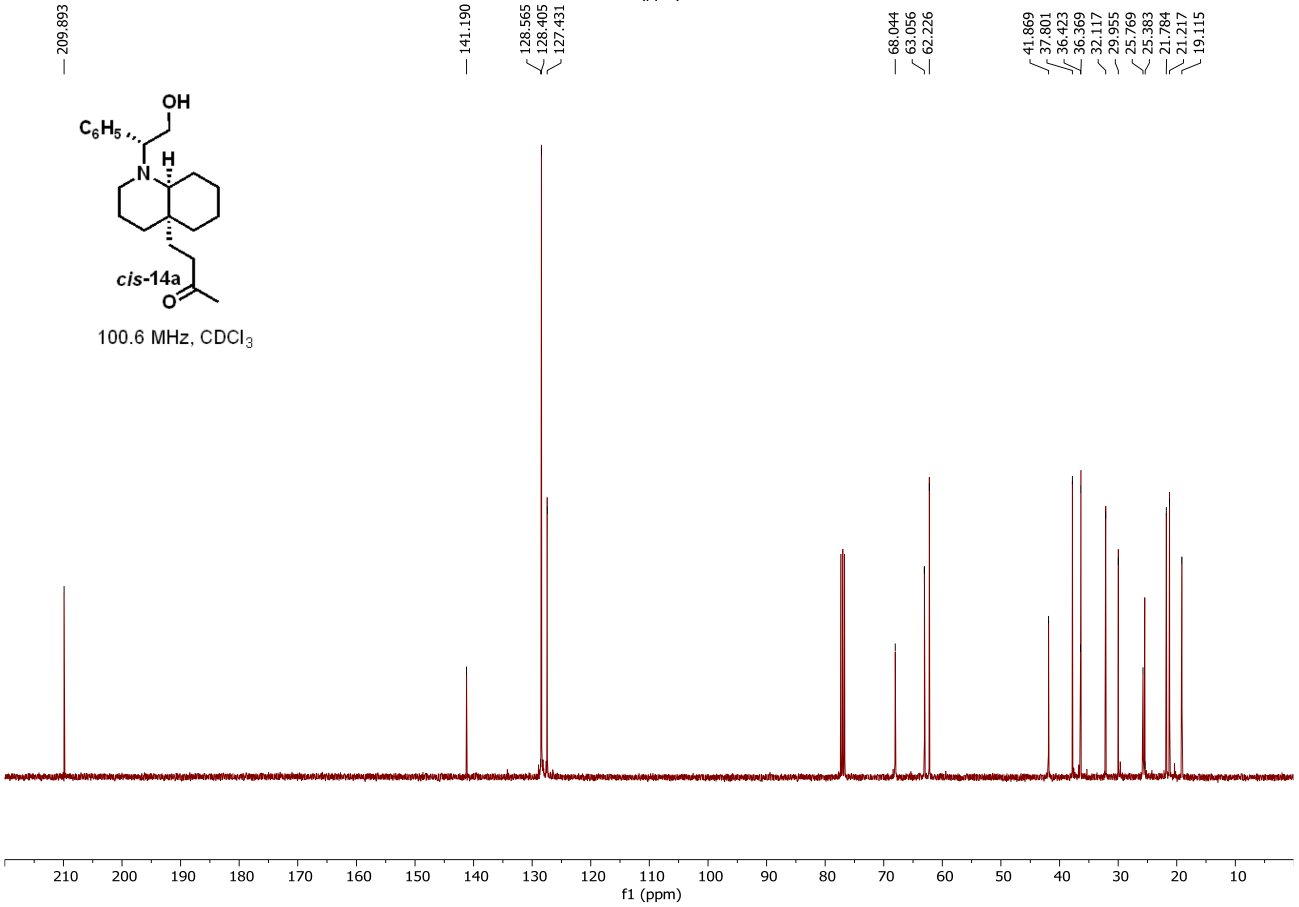

209.893
141.190
128.565
128.405
127.431
68.044
63.056
62.226
41.869
37.801
36.423
36.369
32.117
29.955
25.769
25.383
21.784
21.217
19.115
cis-14a
100.6 MHz, CDCl3
f1 (ppm)

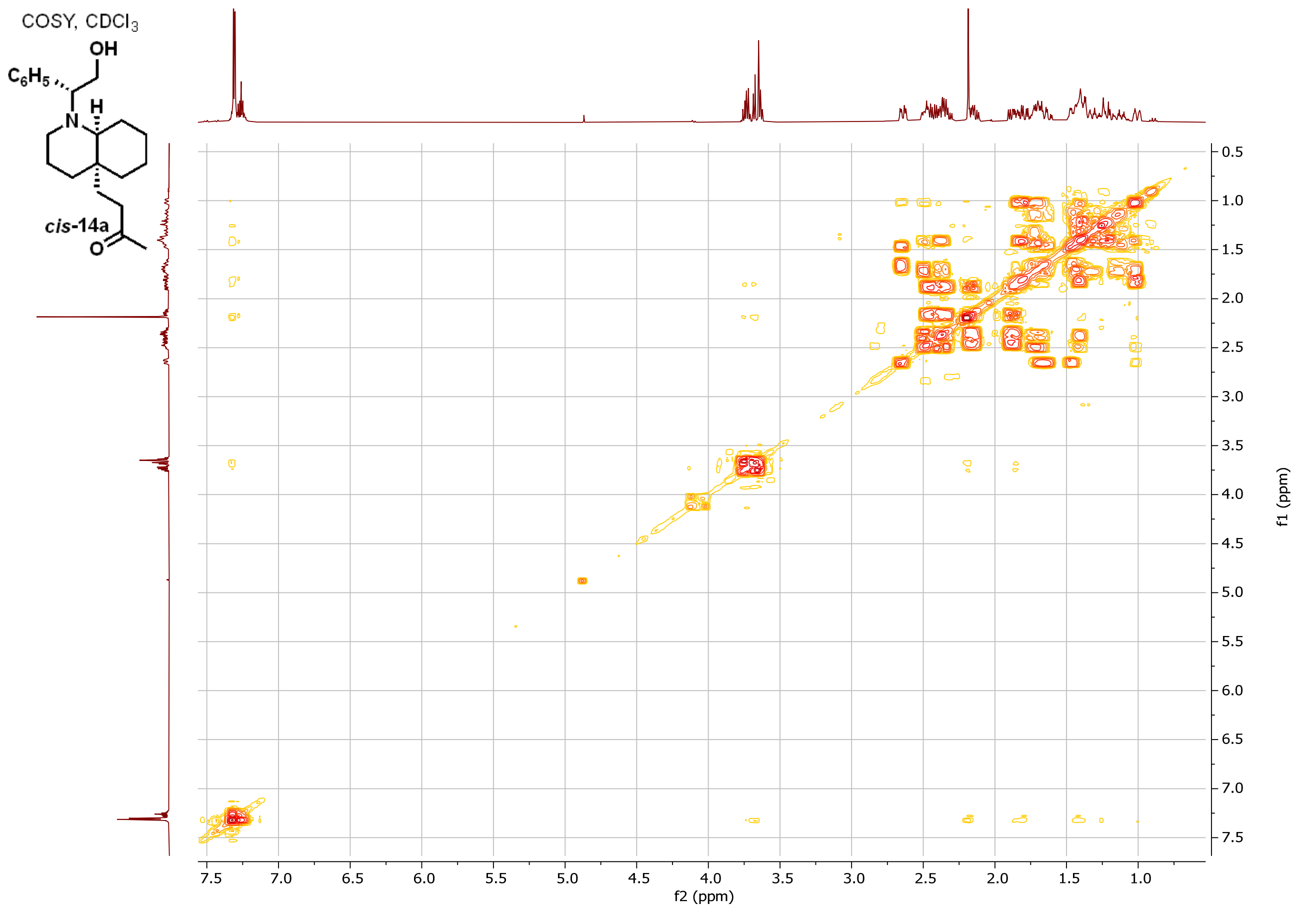
COSY, CDCl3
OH
C6H5
N
H
cis-14a
O
f2 (ppm)
f1 (ppm)

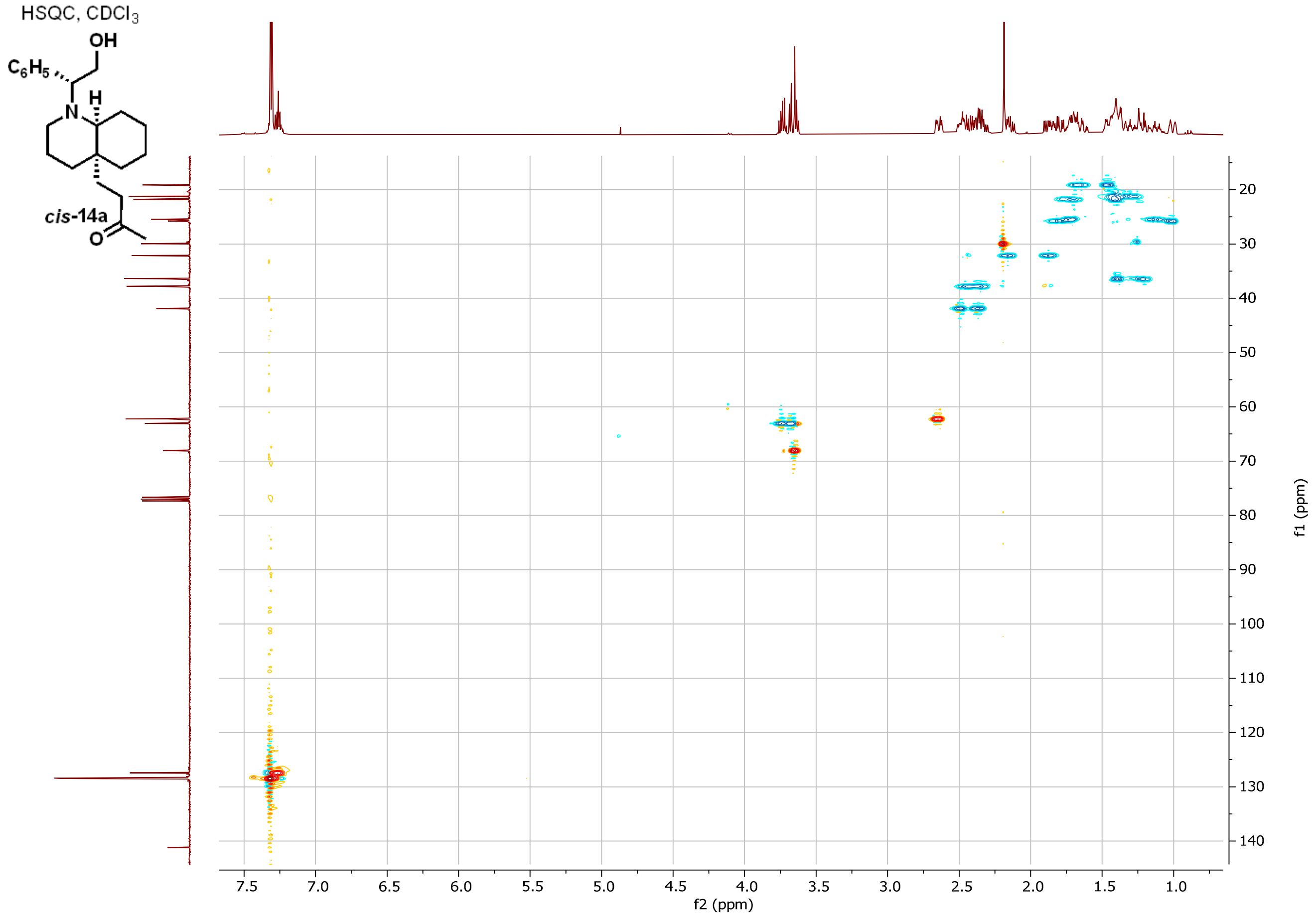
HSQC, CDCl3
OH
C6H5
N
H
cis-14a
O
f2 (ppm)
f1 (ppm)

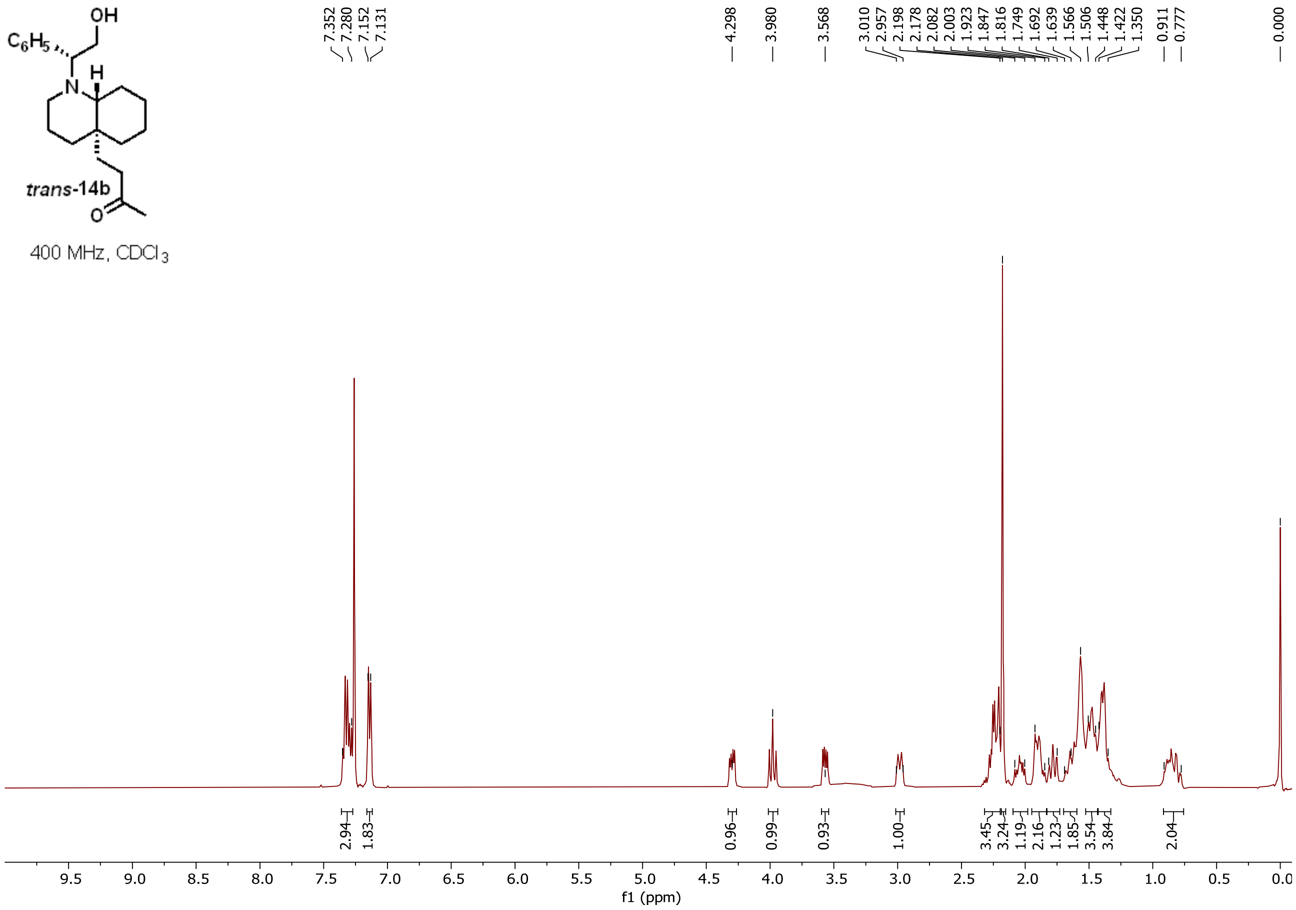

OH
C_6H_5
N
H
trans-14b
O
400 MHz, CDCl3
7.352
7.280
7.152
7.131
4.298
3.980
3.568
3.010
2.957
2.198
2.178
2.082
2.003
1.923
1.847
1.816
1.749
1.692
1.639
1.566
1.506
1.448
1.422
1.350
0.911
0.777
0.000
2.94
1.83
0.96
0.99
0.93
1.00
3.45
3.24
1.19
2.16
1.23
1.85
3.54
3.84
2.04
9.5 9.0 8.5 8.0 7.5 7.0 6.5 6.0 5.5 5.0 4.5 4.0 3.5 3.0 2.5 2.0 1.5 1.0 0.5 0.0
f1 (ppm)

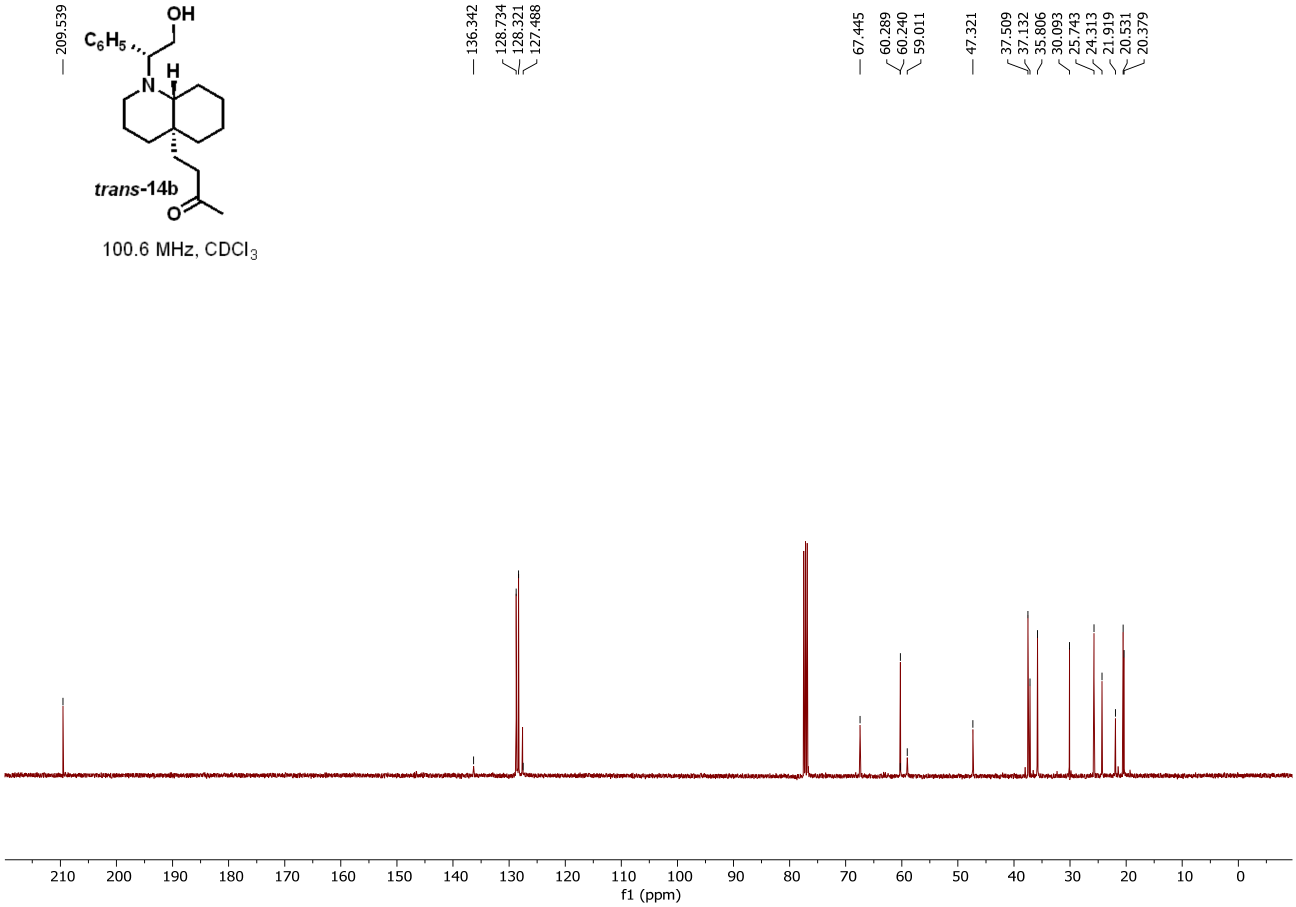

209.539
OH
C_6H_5
N
H
trans-14b
O
100.6 MHz, CDCl3
136.342
128.734
128.321
127.488
67.445
60.289
60.240
59.011
47.321
37.509
37.132
35.806
30.093
25.743
24.313
21.919
20.531
20.379
210 200 190 180 170 160 150 140 130 120 110 100 90 80 70 60 50 40 30 20 10 0
f1 (ppm)

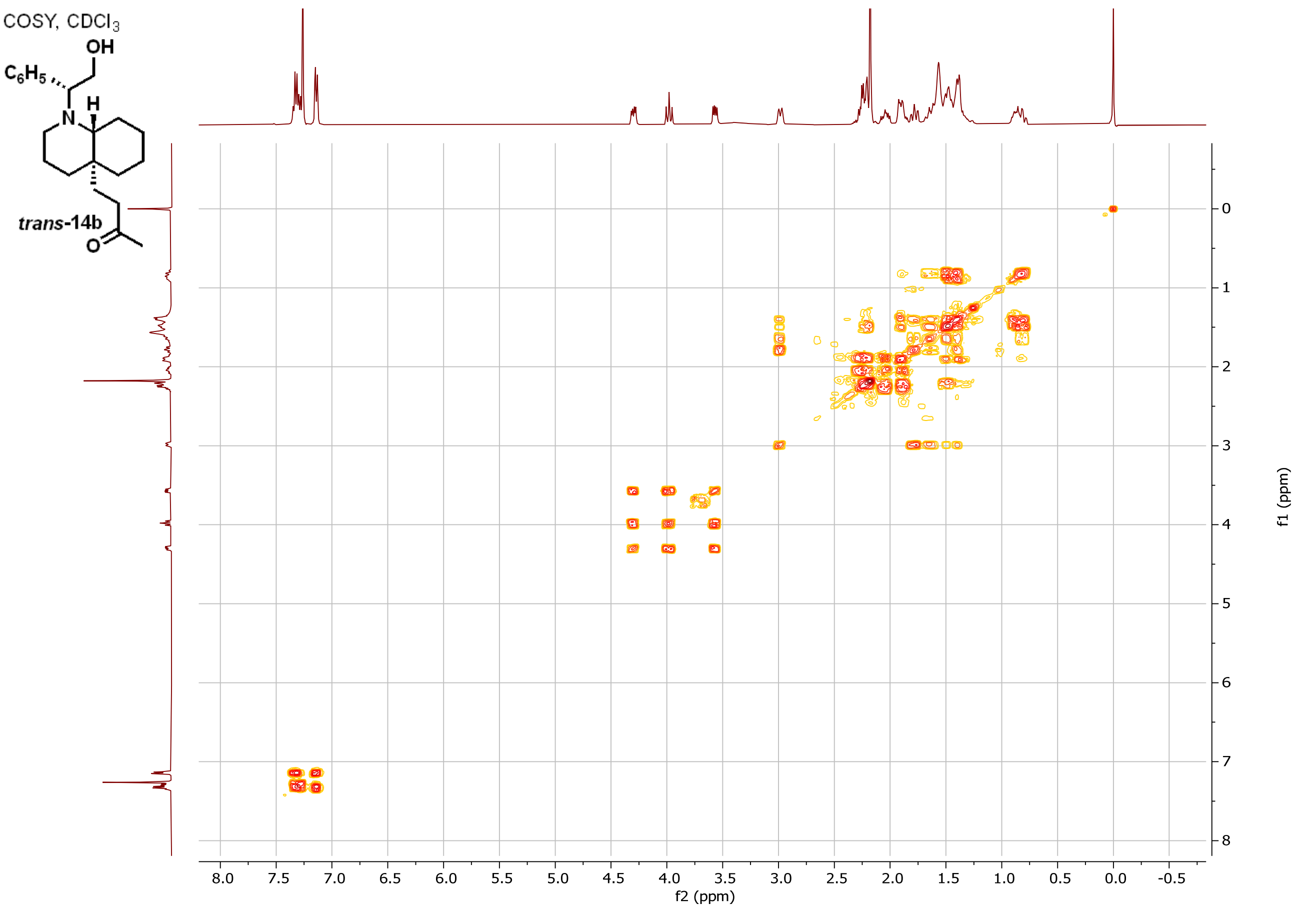
COSY, $CDCl_3$
OH
C_6H_5
N
H
trans-14b
O
f2 (ppm)
f1 (ppm)

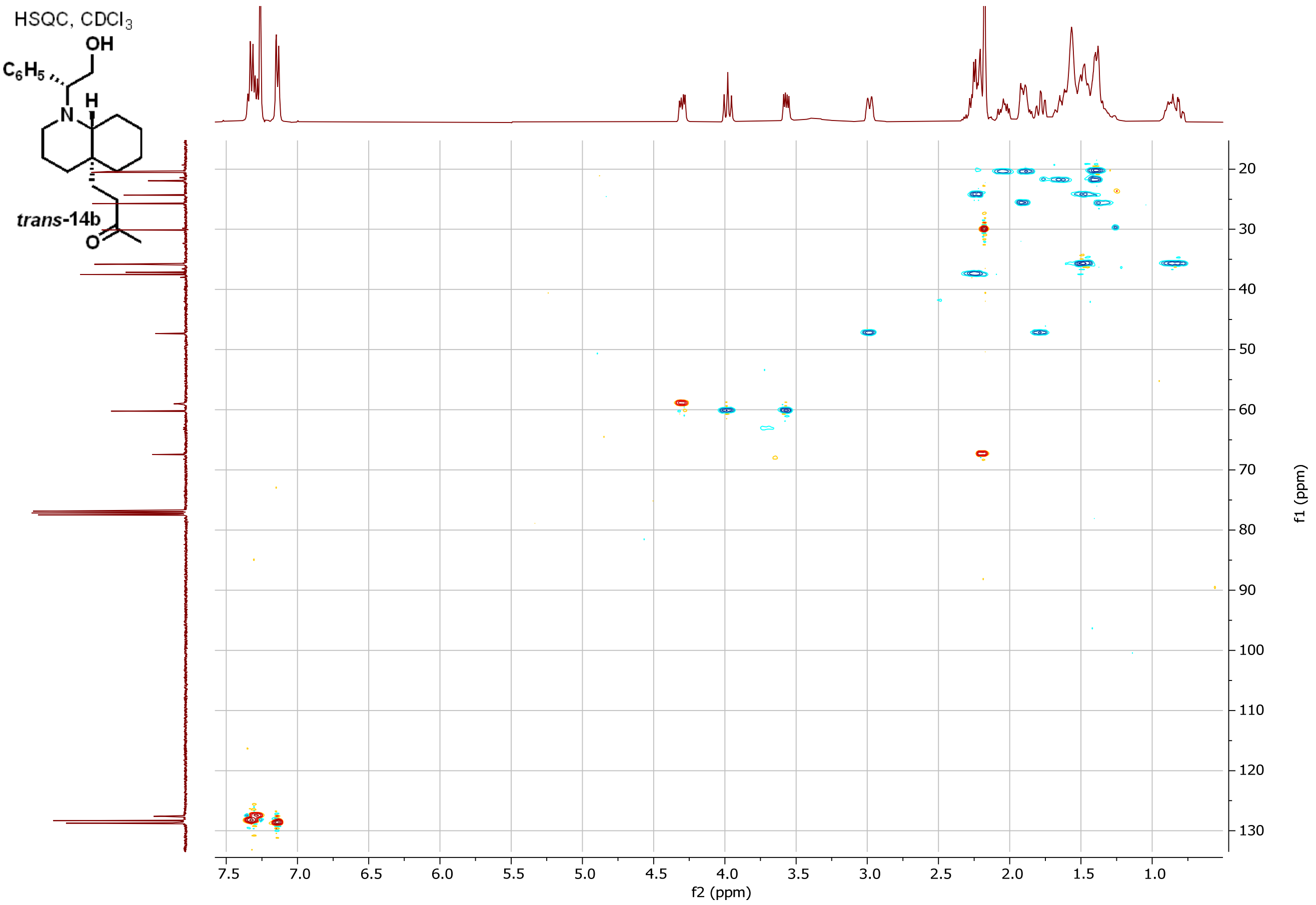
HSQC, $CDCl_3$
OH
C_6H_5
N
H
trans-14b
O
f2 (ppm)
f1 (ppm)

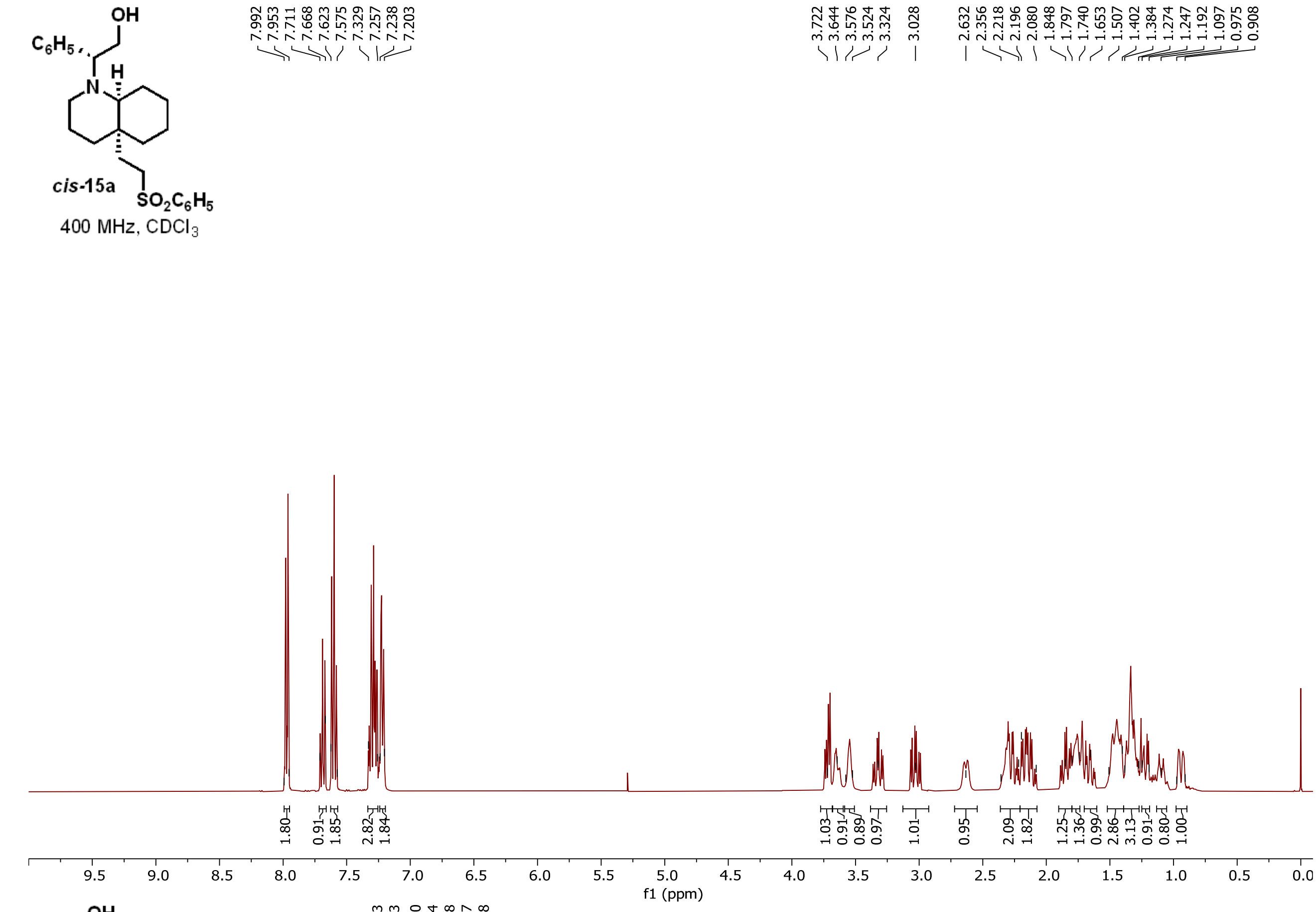
cis-15a
400 MHz, CDCl3
7.992
7.953
7.711
7.668
7.623
7.575
7.329
7.257
7.238
7.203
3.722
3.644
3.576
3.524
3.324
3.028
2.632
2.356
2.218
2.196
2.080
1.848
1.797
1.740
1.653
1.507
1.402
1.384
1.274
1.247
1.192
1.097
0.975
0.908
f1 (ppm)

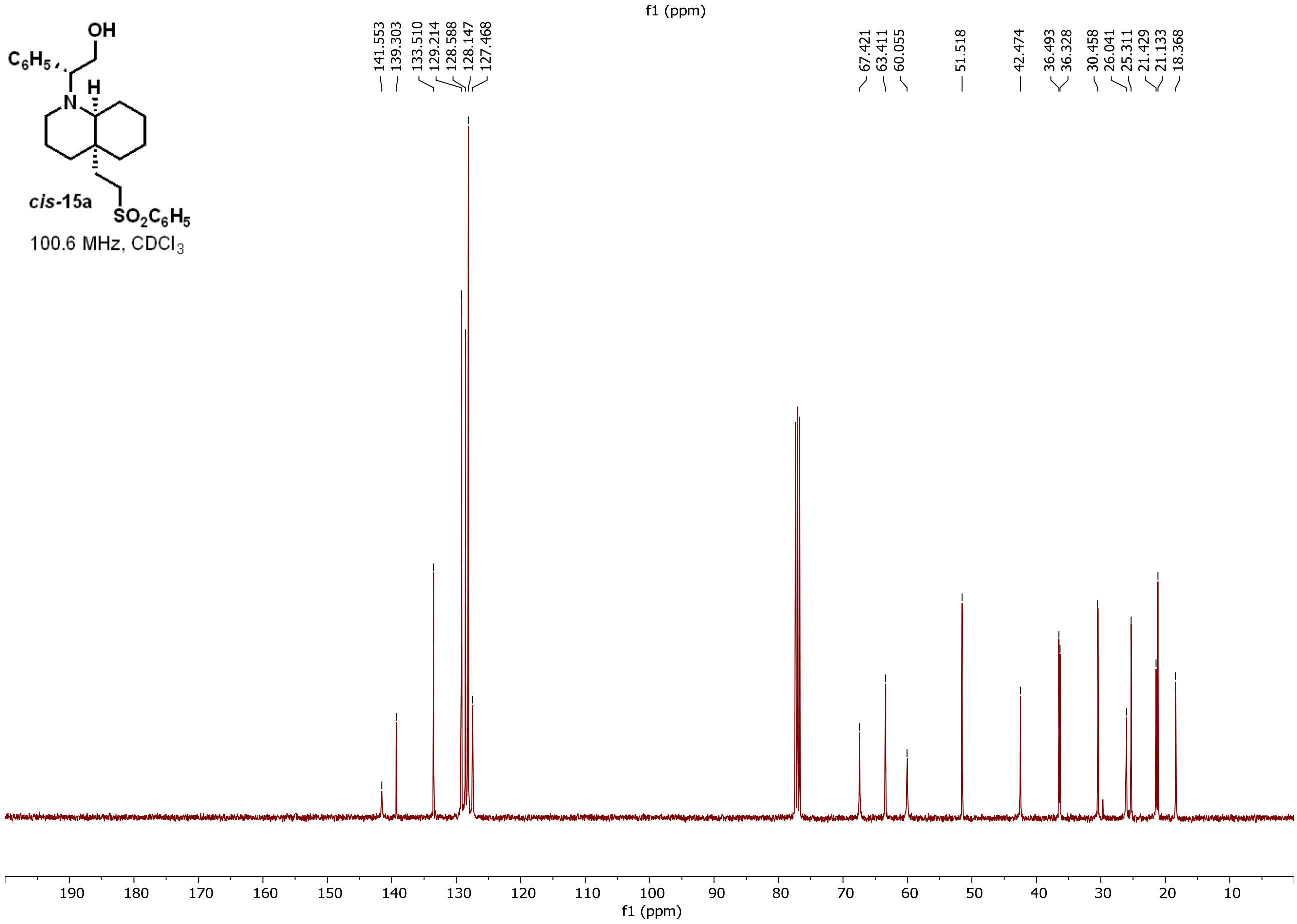
cis-15a
100.6 MHz, CDCl3
141.553
139.303
133.510
129.214
128.588
128.147
127.468
67.421
63.411
60.055
51.518
42.474
36.493
36.328
30.458
26.041
25.311
21.429
21.133
18.368
f1 (ppm)

COSY, $CDCl_3$

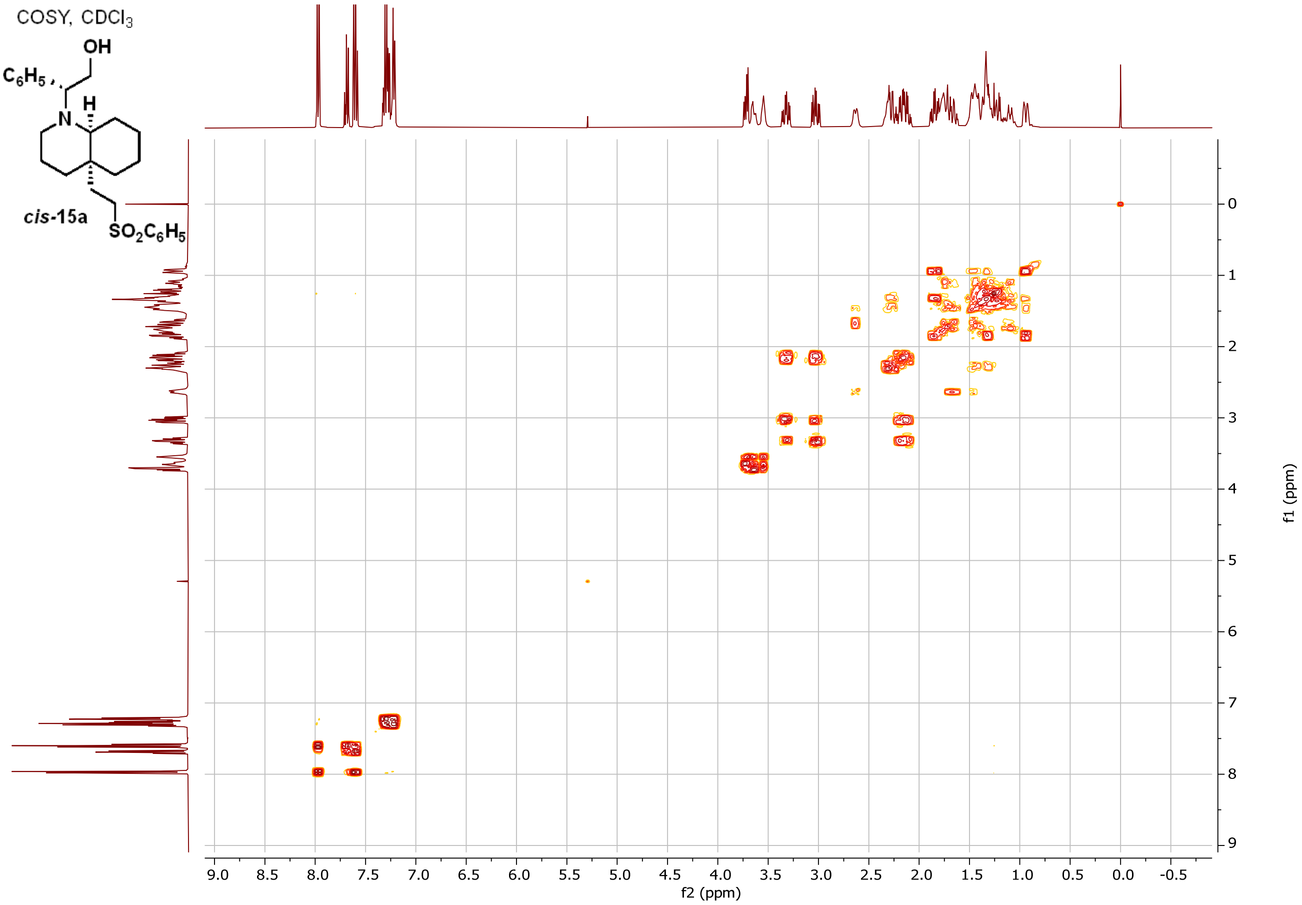

HSQC, $CDCl_3$

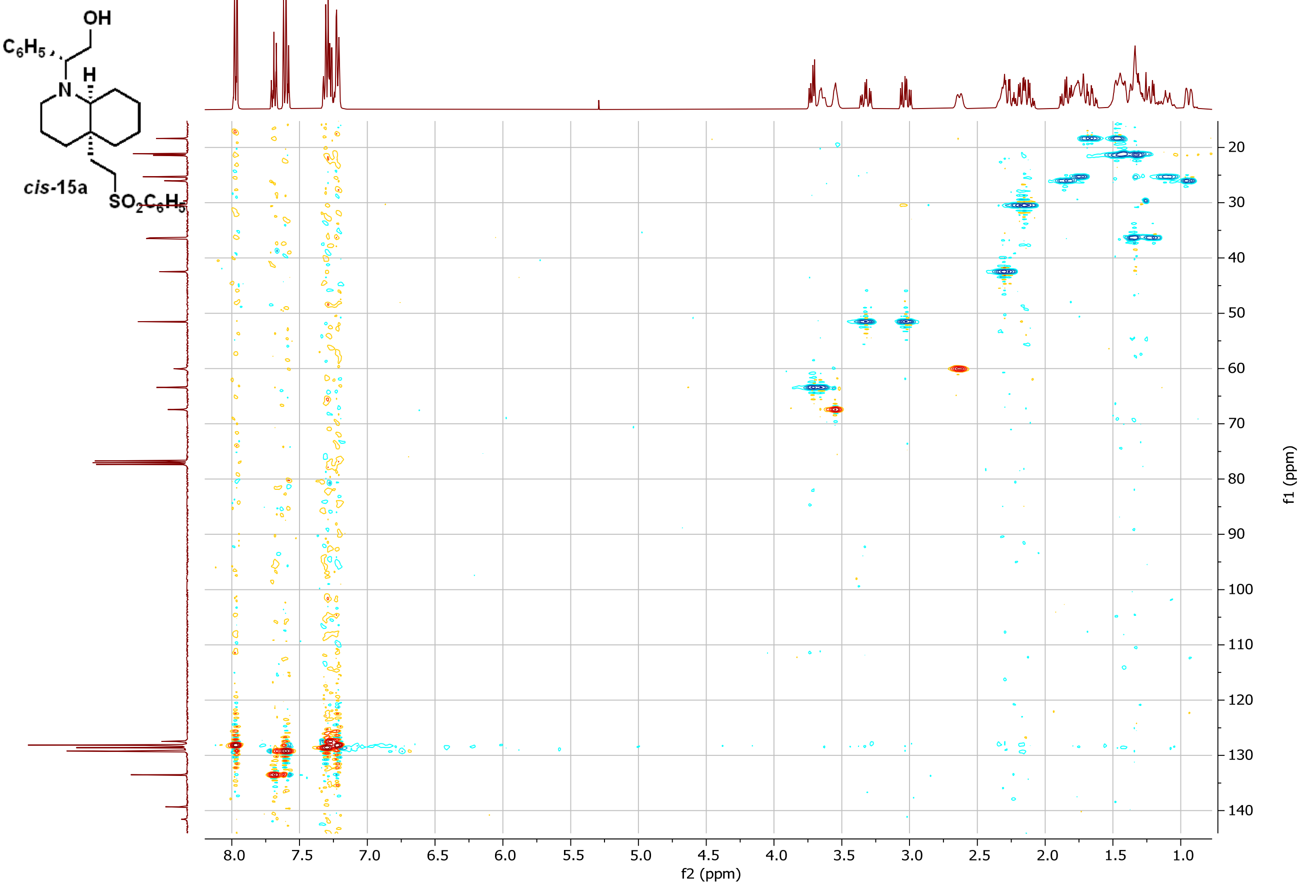

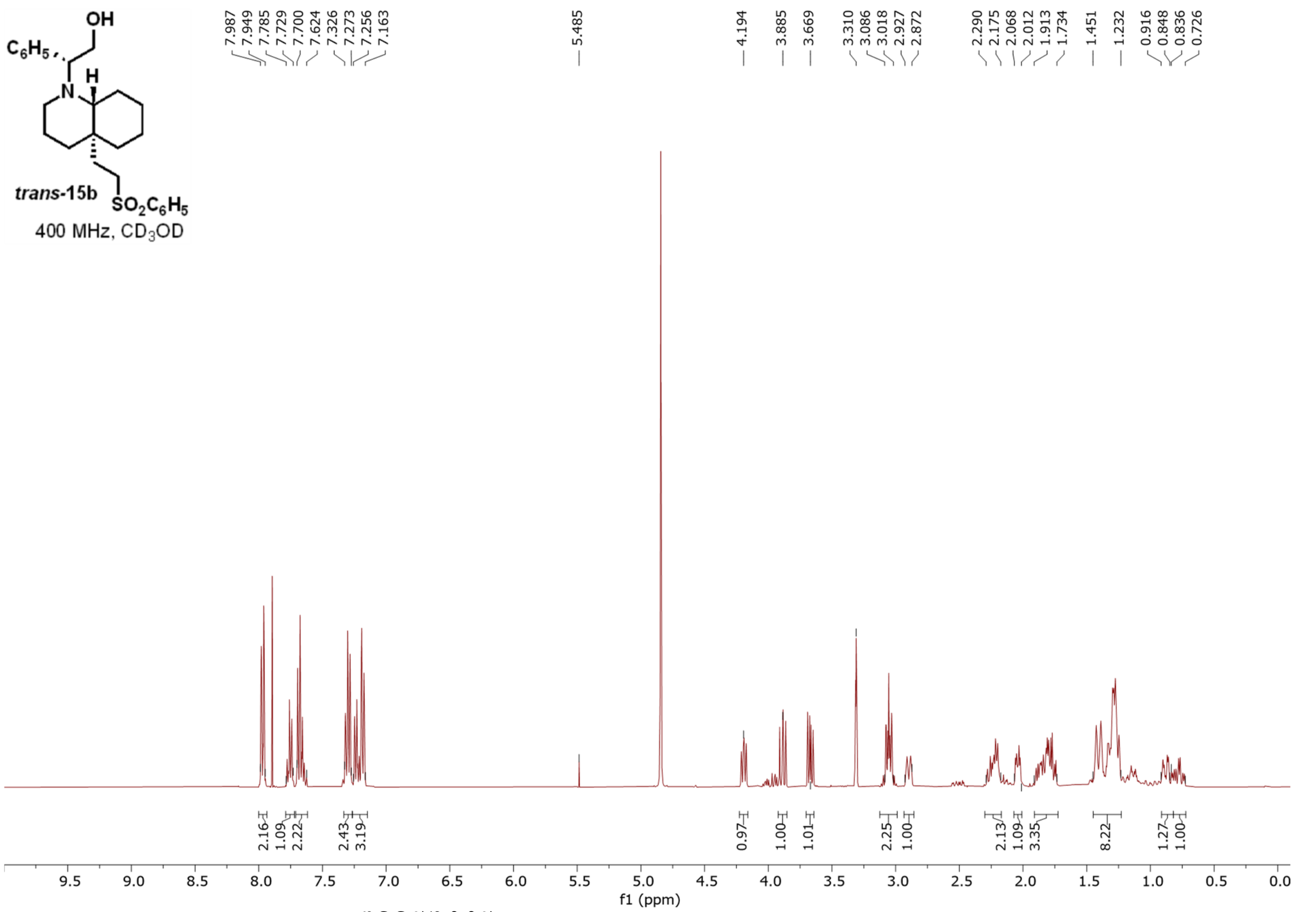

OH
C6H5
N
H
trans-15b
SO2C6H5
400 MHz, CD3OD
7.987
7.949
7.785
7.729
7.700
7.624
7.326
7.273
7.256
7.163
5.485
4.194
3.885
3.669
3.310
3.086
3.018
2.927
2.872
2.290
2.175
2.068
2.012
1.913
1.734
1.451
1.232
0.916
0.848
0.836
0.726
2.16
1.09
2.22
2.43
3.19
0.97
1.00
1.01
2.25
1.00
2.13
1.09
3.35
8.22
1.27
1.00
9.5 9.0 8.5 8.0 7.5 7.0 6.5 6.0 5.5 5.0 4.5 4.0 3.5 3.0 2.5 2.0 1.5 1.0 0.5 0.0
f1 (ppm)

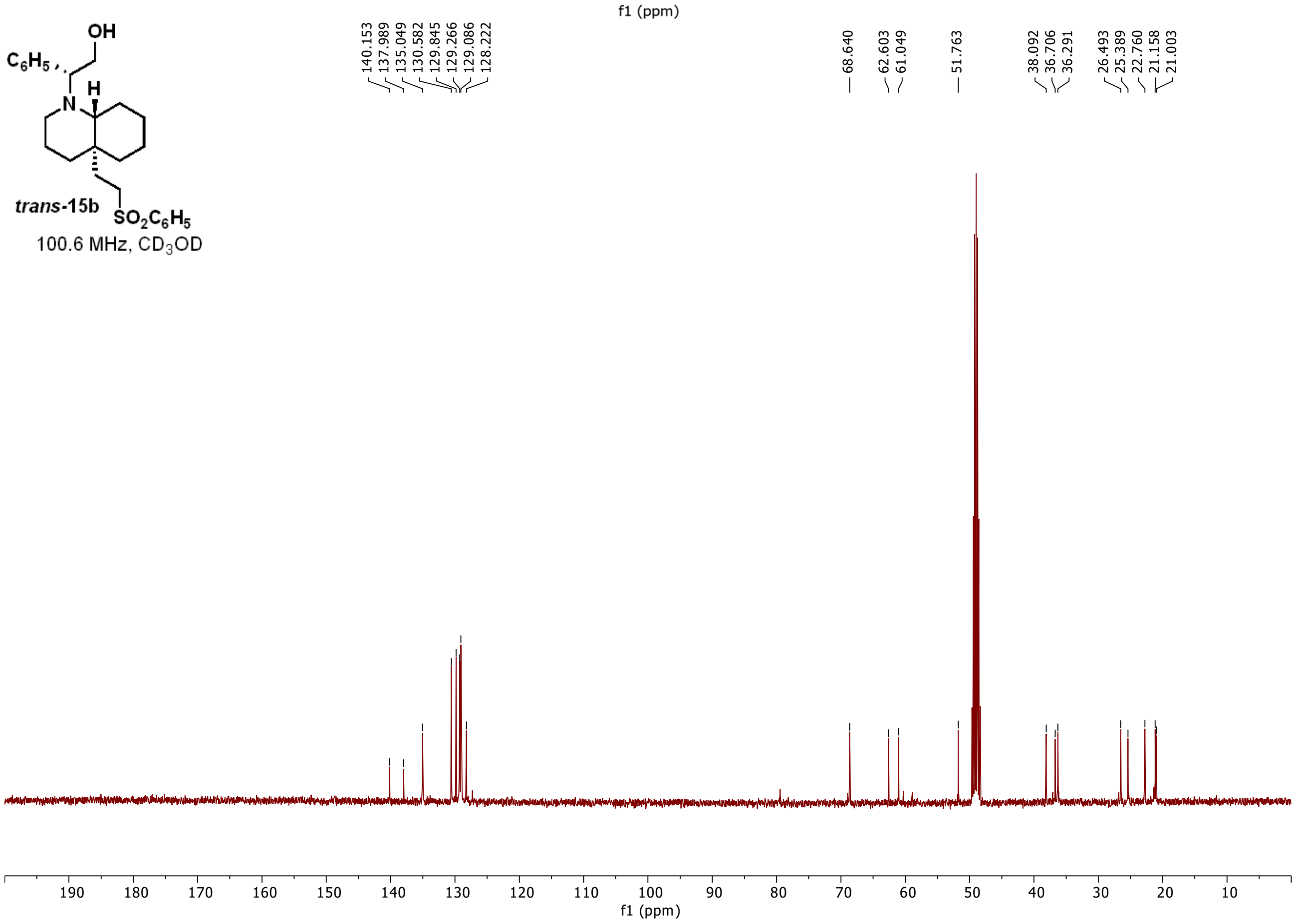

OH
C6H5
N
H
trans-15b
SO2C6H5
100.6 MHz, CD3OD
140.153
137.989
135.049
130.582
129.845
129.266
129.086
128.222
68.640
62.603
61.049
51.763
38.092
36.706
36.291
26.493
25.389
22.760
21.158
21.003
190 180 170 160 150 140 130 120 110 100 90 80 70 60 50 40 30 20 10
f1 (ppm)

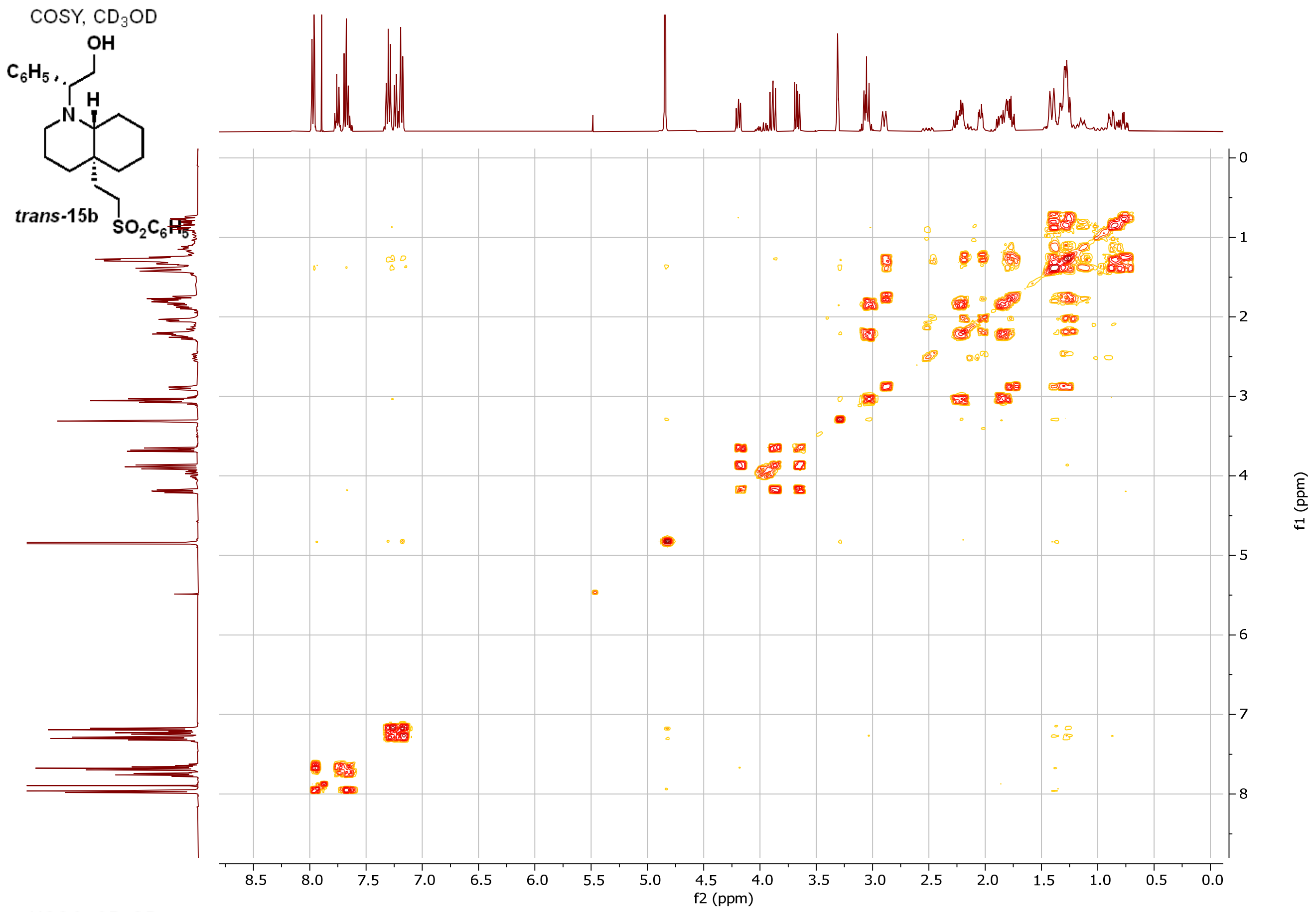
COSY, CD3OD
OH
C6H5
N
H
trans-15b
SO2C6H5
f2 (ppm)
f1 (ppm)

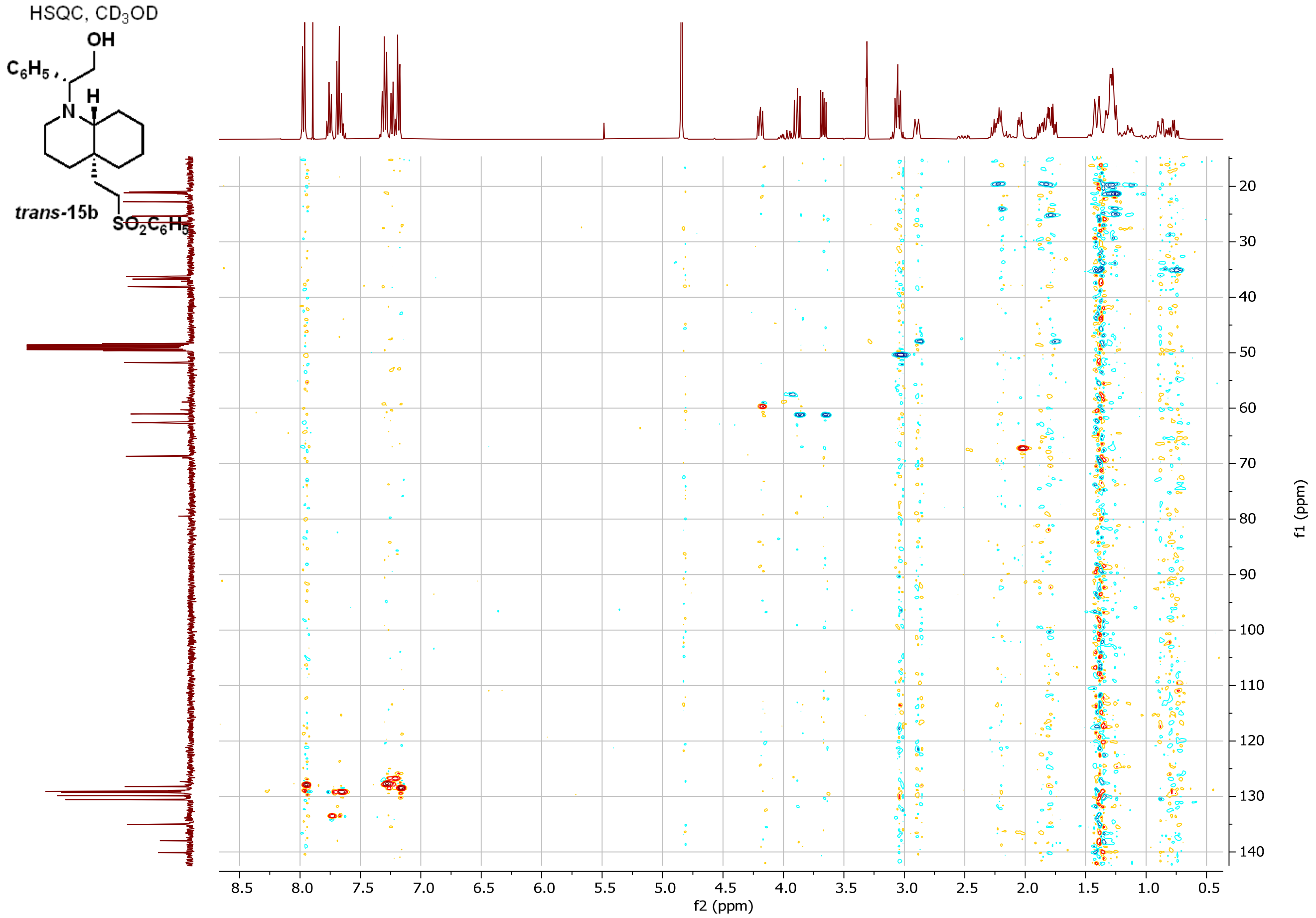
HSQC, CD3OD
OH
C6H5
N
H
trans-15b
SO2C6H5
f2 (ppm)
f1 (ppm)

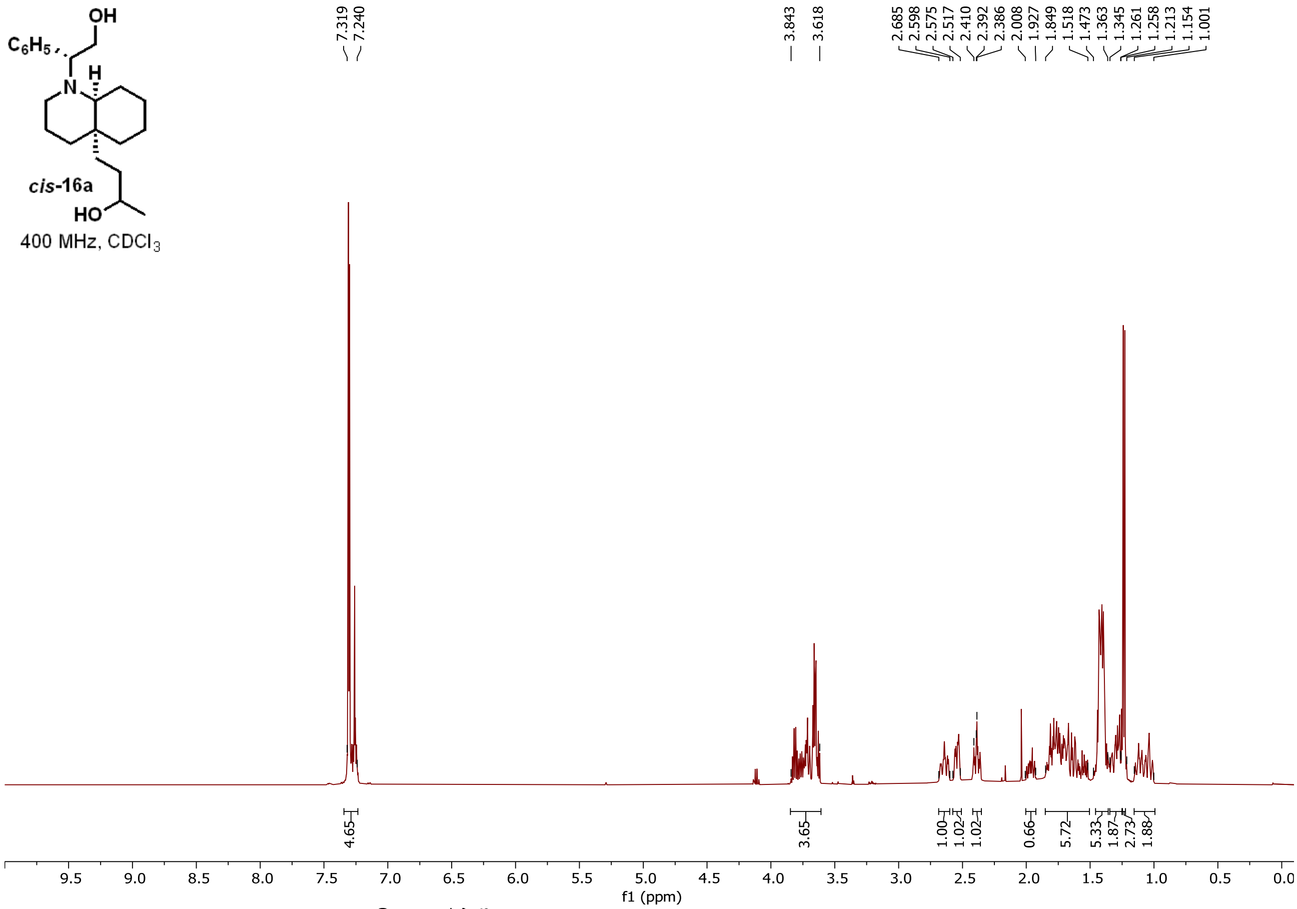
OH
C6H5
N
H
cis-16a
HO
400 MHz, CDCl3
7.319
7.240
3.843
3.618
2.685
2.598
2.575
2.517
2.410
2.392
2.386
2.008
1.927
1.849
1.518
1.473
1.363
1.345
1.261
1.258
1.213
1.154
1.001
4.65
3.65
1.00
1.02
1.02
0.66
5.72
5.33
1.87
2.73
1.88
9.5
9.0
8.5
8.0
7.5
7.0
6.5
6.0
5.5
5.0
4.5
4.0
3.5
3.0
2.5
2.0
1.5
1.0
0.5
0.0
f1 (ppm)

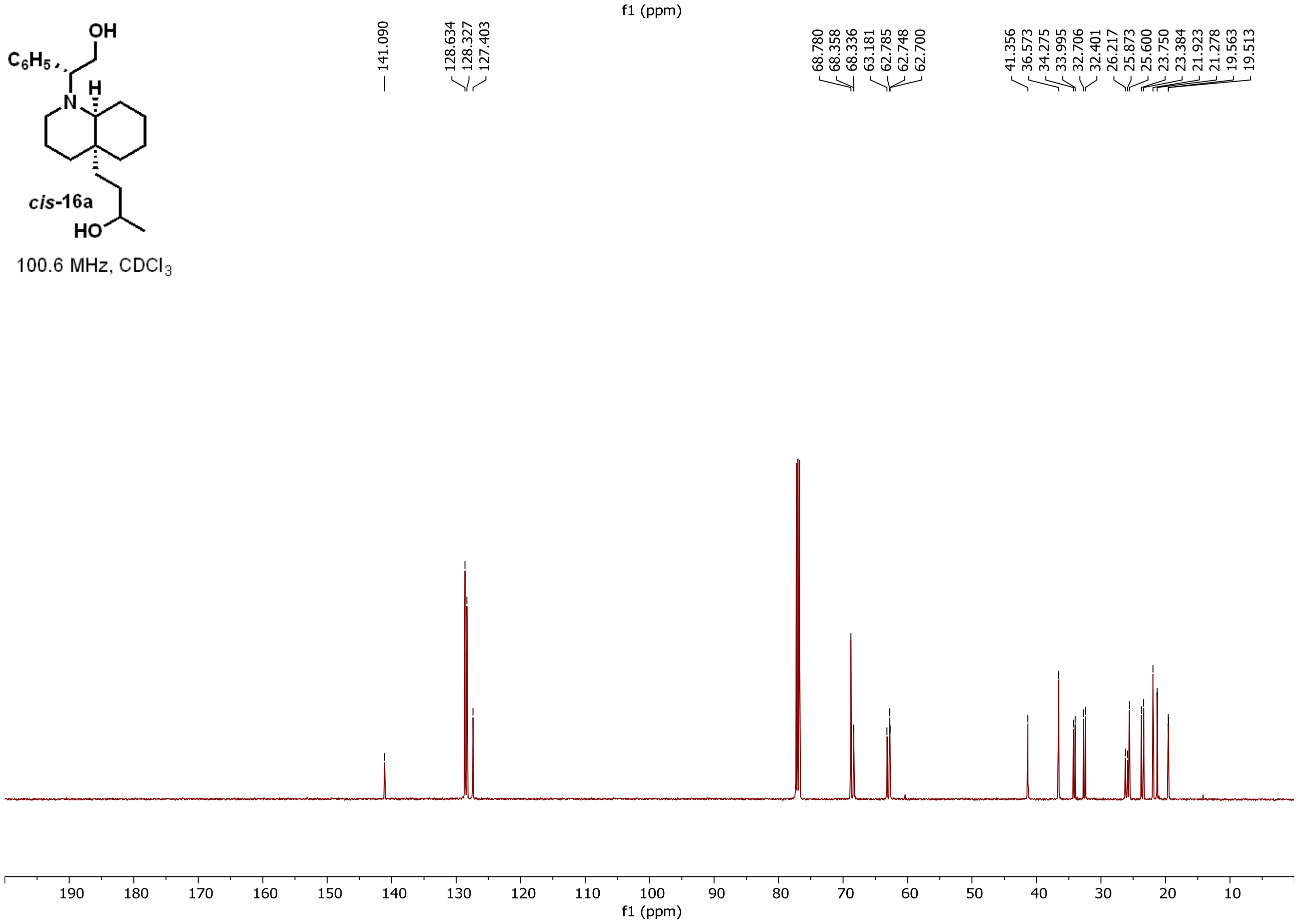
OH
C6H5
N
H
cis-16a
HO
100.6 MHz, CDCl3
141.090
128.634
128.327
127.403
68.780
68.358
68.336
63.181
62.785
62.748
62.700
41.356
36.573
34.275
33.995
32.706
32.401
26.217
25.873
25.600
23.750
23.384
21.923
21.278
19.563
19.513
190
180
170
160
150
140
130
120
110
100
90
80
70
60
50
40
30
20
10
f1 (ppm)

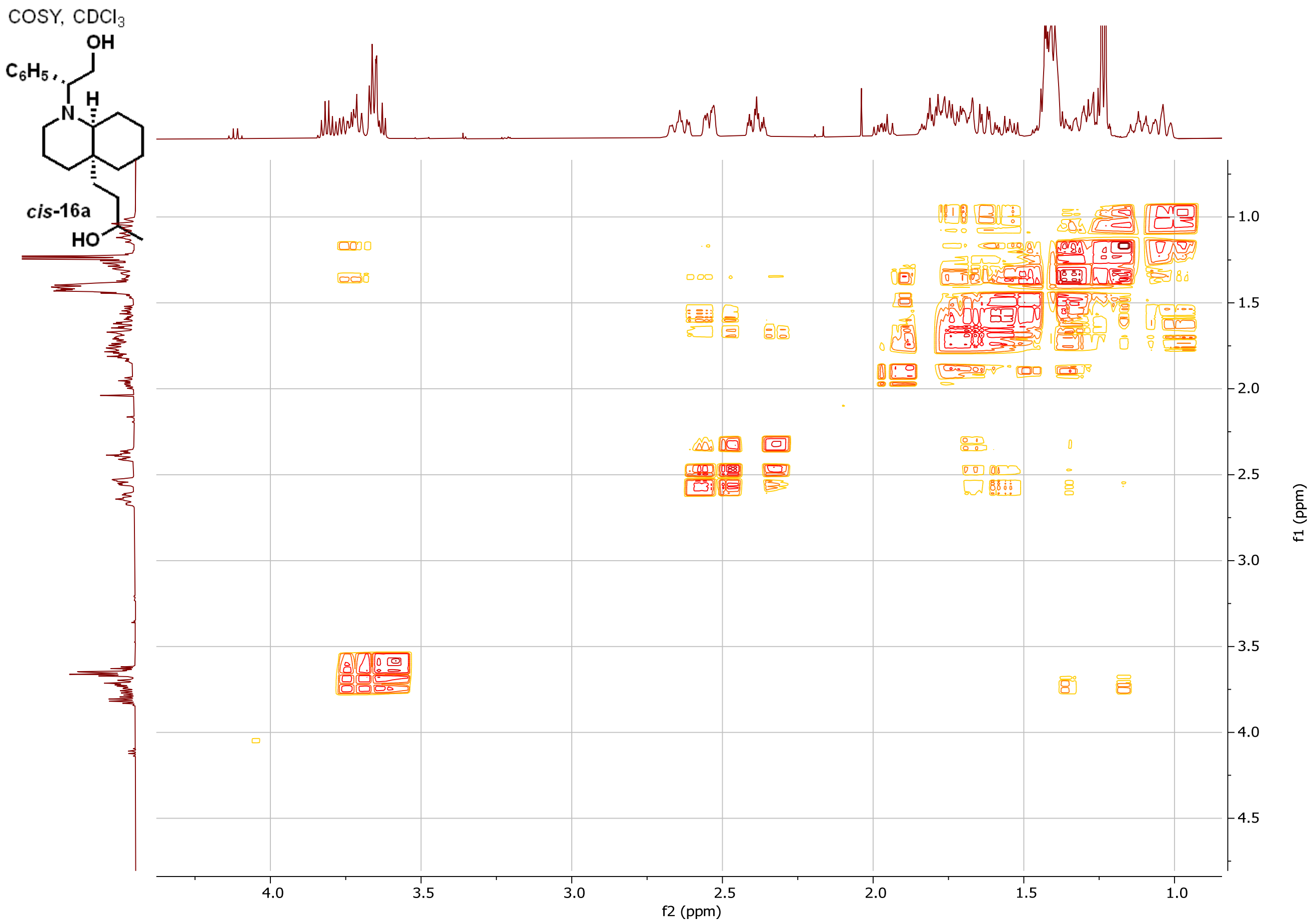

COSY, CDCl3
OH
C6H5
N
H
cis-16a
HO
f2 (ppm)
f1 (ppm)

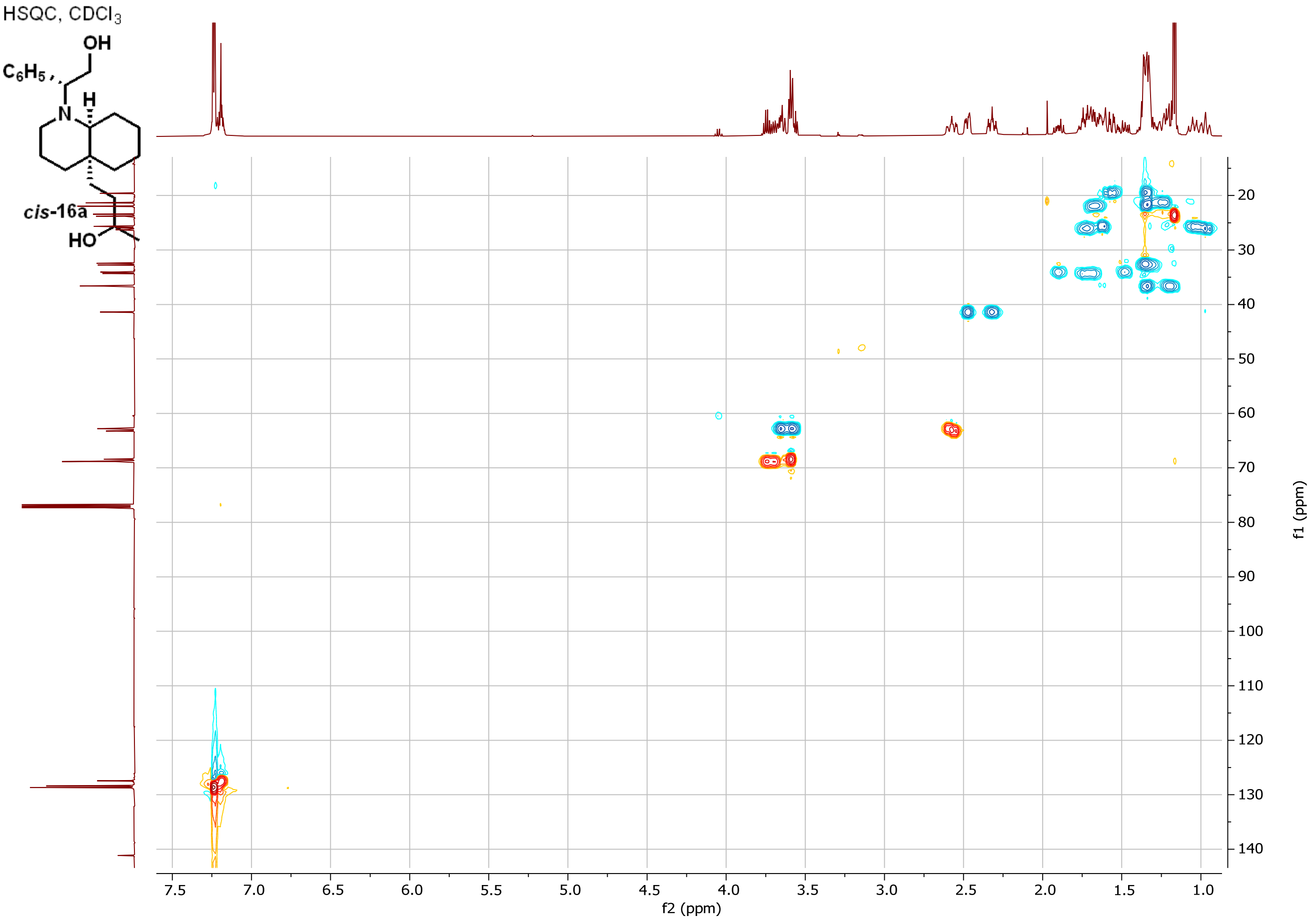

HSQC, CDCl3
OH
C6H5
N
H
cis-16a
HO
f2 (ppm)
f1 (ppm)

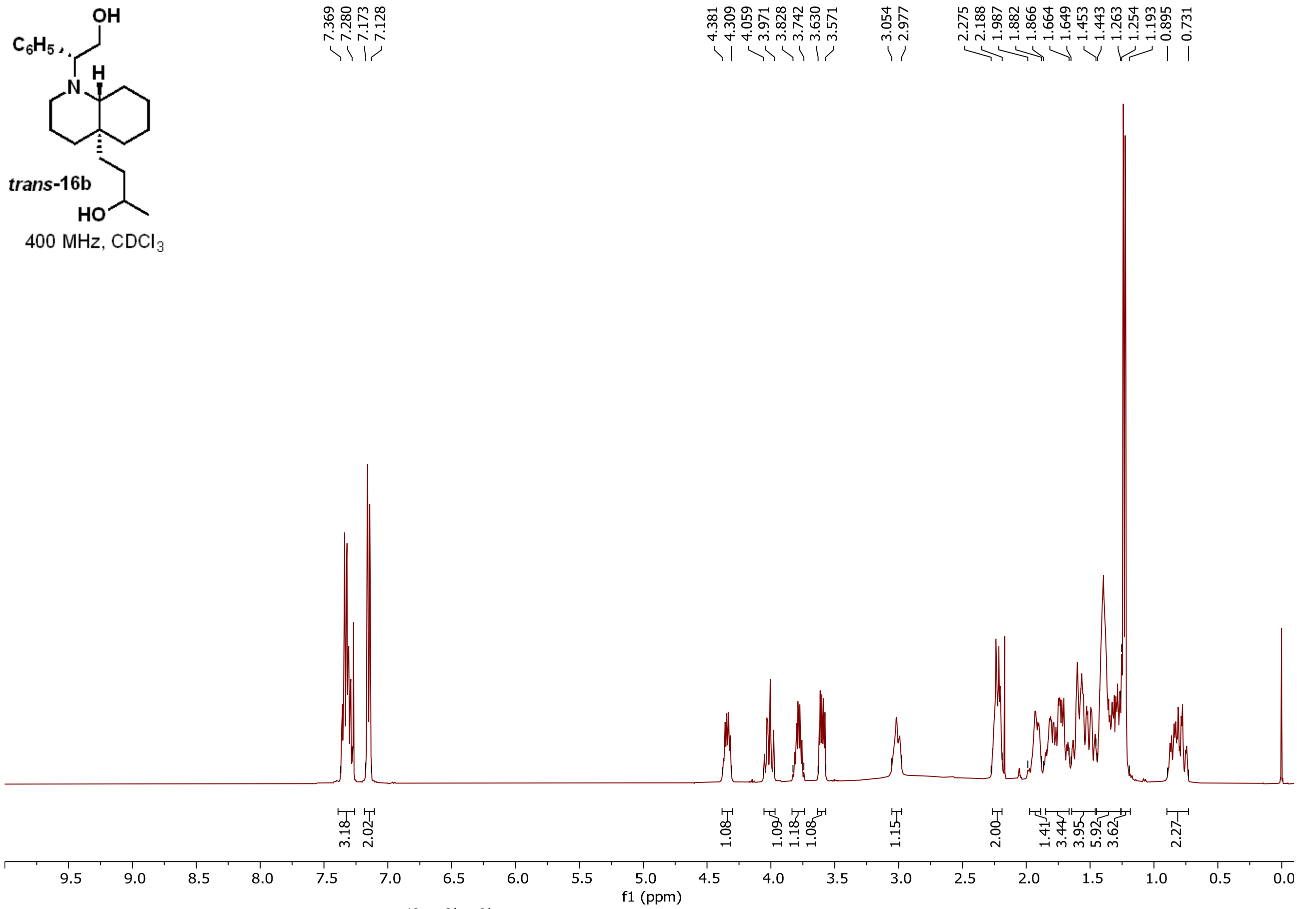
OH
C6H5
N
H
trans-16b
HO
400 MHz, CDCl3
7.369
7.280
7.173
7.128
4.381
4.309
4.059
3.971
3.828
3.742
3.630
3.571
3.054
2.977
2.275
2.188
1.987
1.882
1.866
1.664
1.649
1.453
1.443
1.263
1.254
1.193
0.895
0.731
3.18
2.02
1.08
1.09
1.18
1.08
1.15
2.00
1.41
3.44
3.95
5.92
3.62
2.27
9.5 9.0 8.5 8.0 7.5 7.0 6.5 6.0 5.5 5.0 4.5 4.0 3.5 3.0 2.5 2.0 1.5 1.0 0.5 0.0
f1 (ppm)

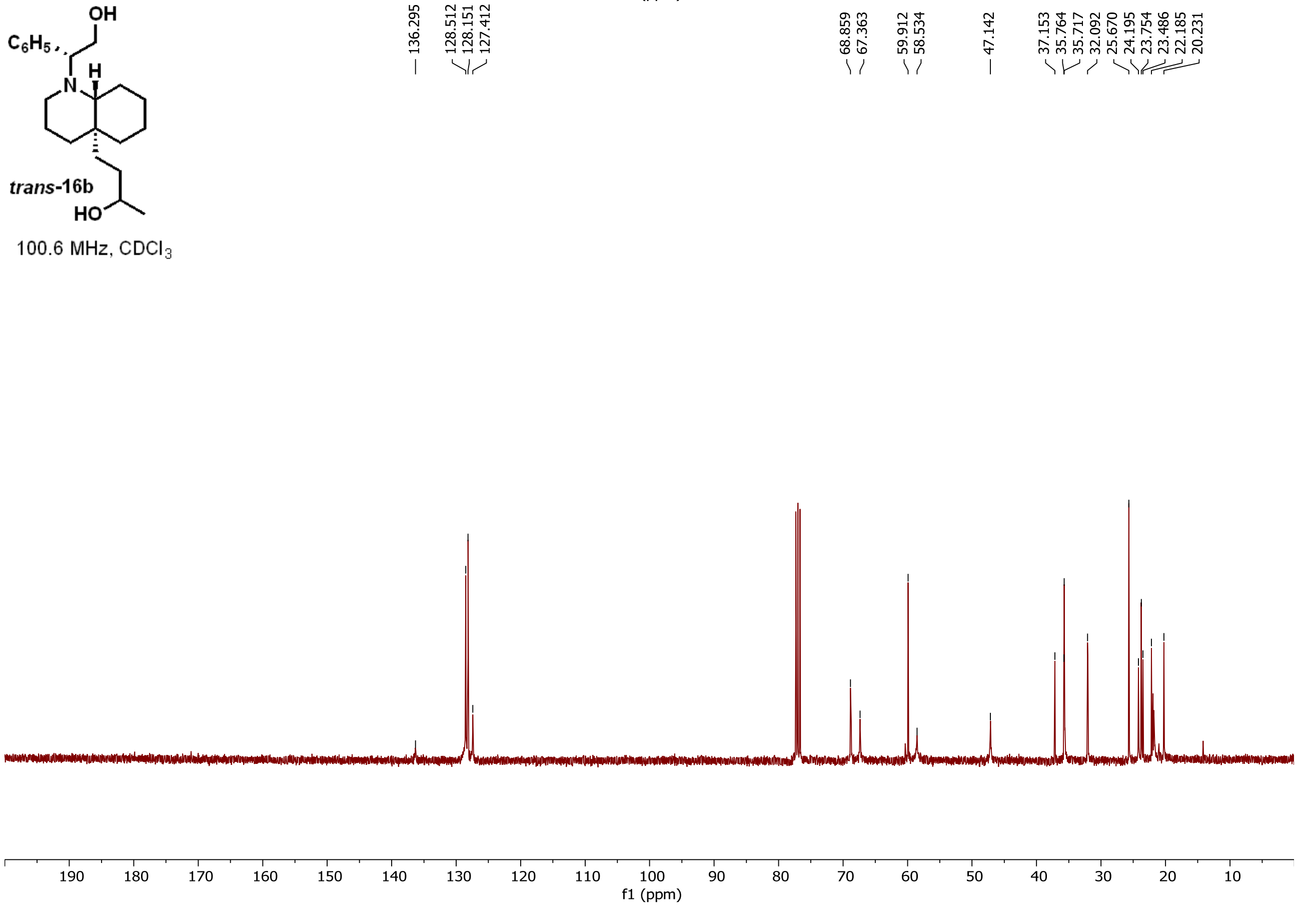
OH
C6H5
N
H
trans-16b
HO
100.6 MHz, CDCl3
136.295
128.512
128.151
127.412
68.859
67.363
59.912
58.534
47.142
37.153
35.764
35.717
32.092
25.670
24.195
23.754
23.486
22.185
20.231
190 180 170 160 150 140 130 120 110 100 90 80 70 60 50 40 30 20 10
f1 (ppm)

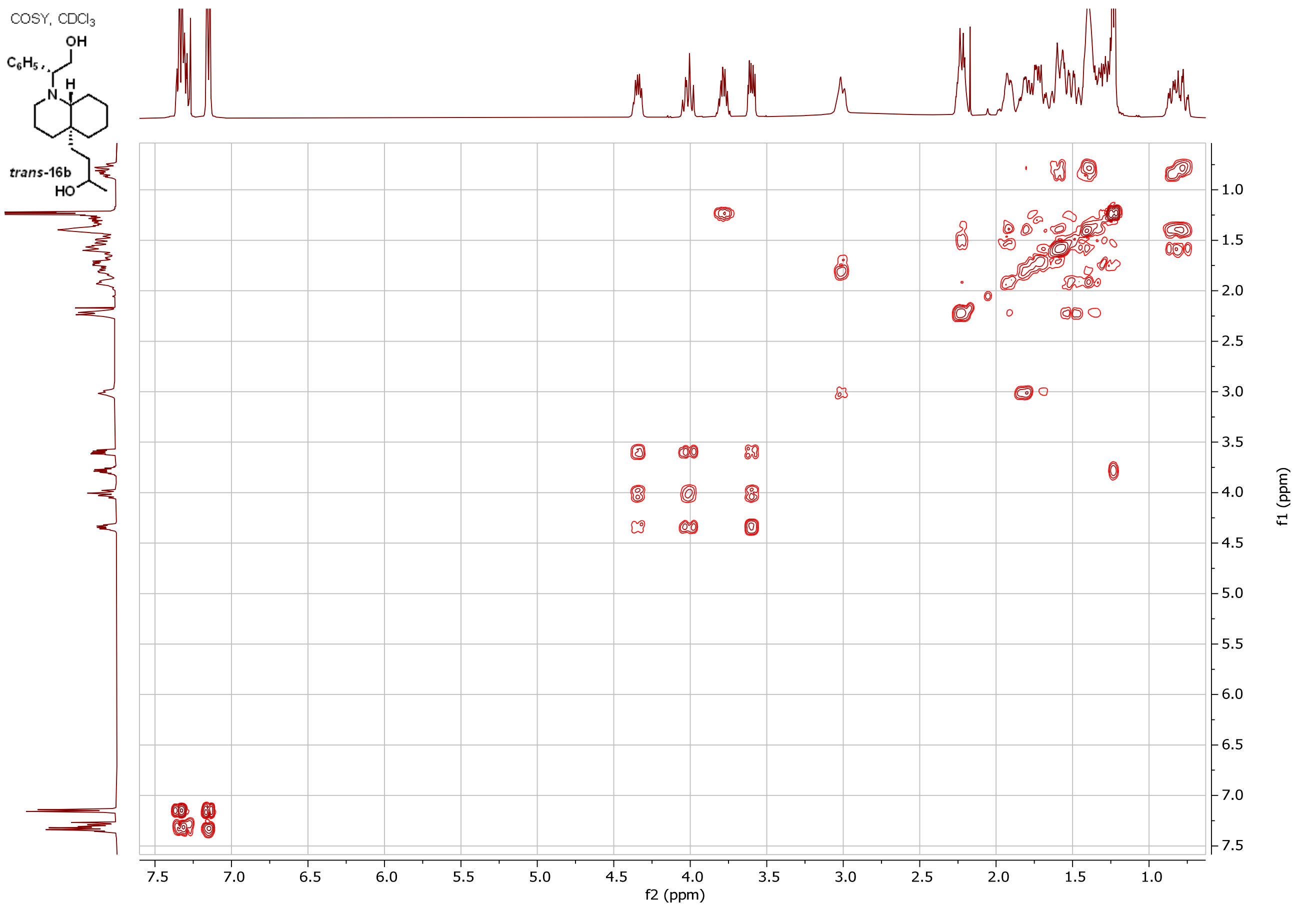
COSY, CDCl3
OH
C6H5
N
H
trans-16b
HO
f2 (ppm)
f1 (ppm)

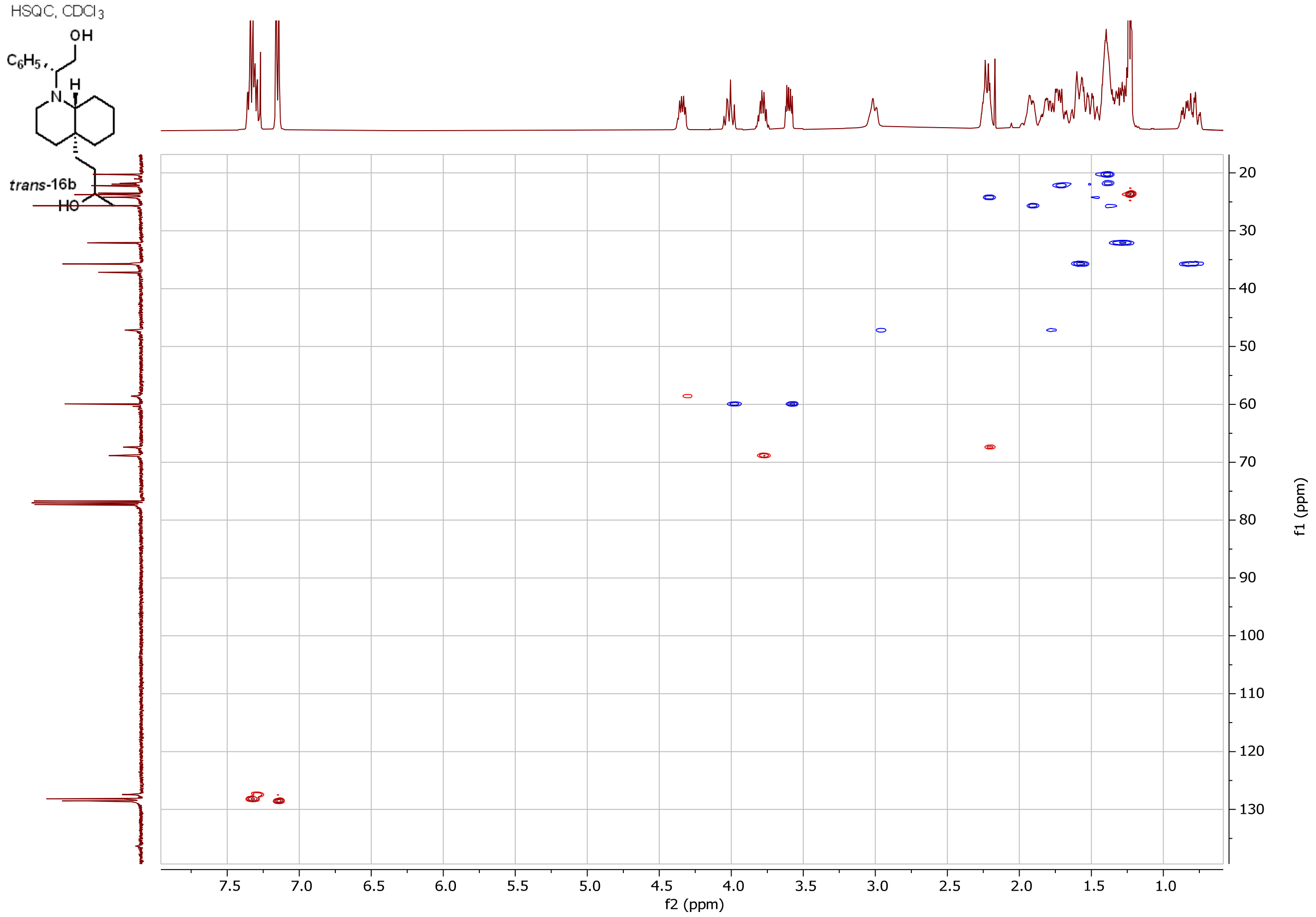
HSQC, CDCl3
OH
C6H5
N
H
trans-16b
HO
f2 (ppm)
f1 (ppm)

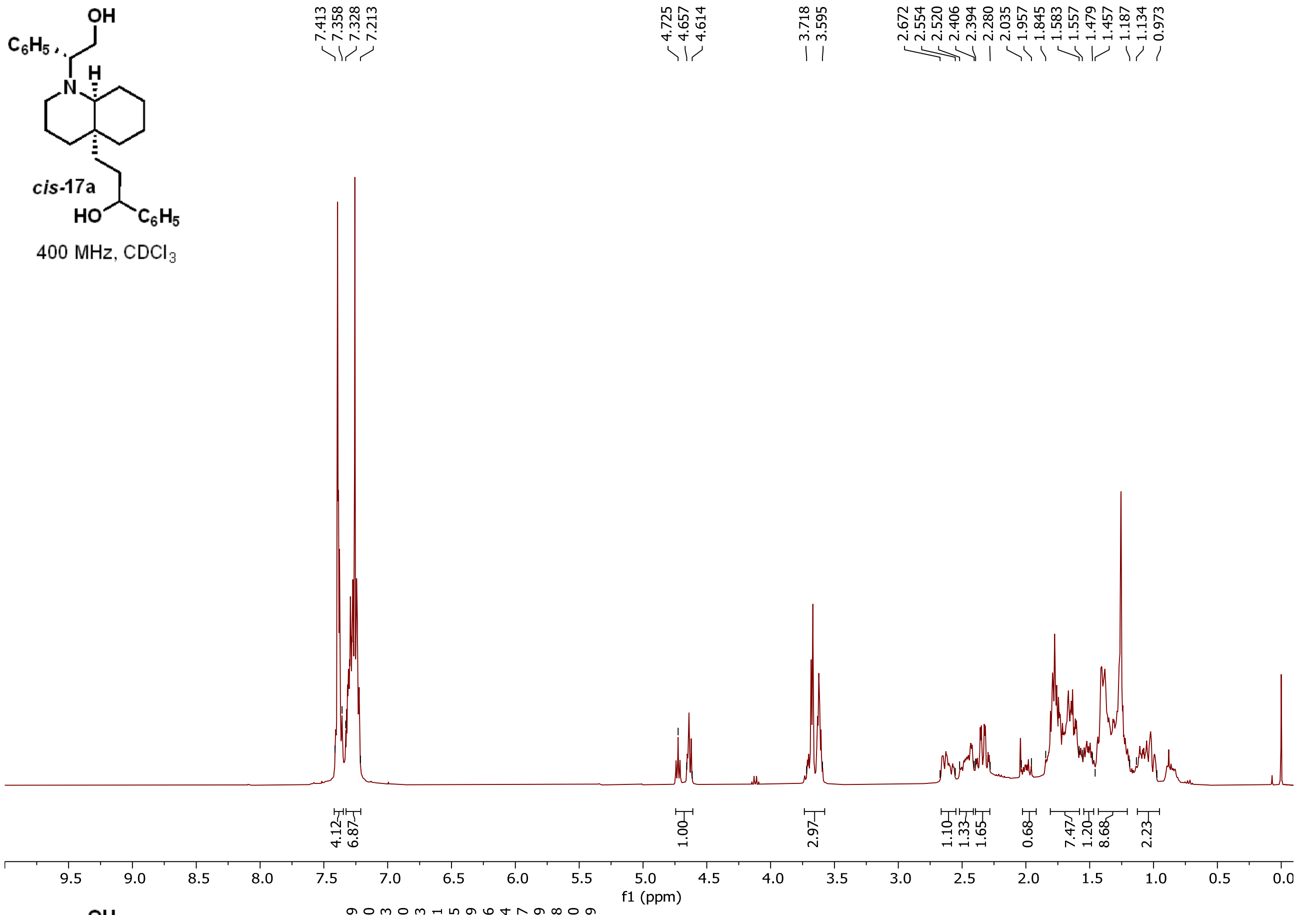

OH
C6H5
N
H
cis-17a
HO
C6H5
400 MHz, CDCl3
7.413
7.358
7.328
7.213
4.725
4.657
4.614
3.718
3.595
2.672
2.554
2.520
2.406
2.394
2.280
2.035
1.957
1.845
1.583
1.557
1.479
1.457
1.187
1.134
0.973
4.12
6.87
1.00
2.97
1.10
1.33
1.65
0.68
7.47
1.20
8.68
2.23
f1 (ppm)

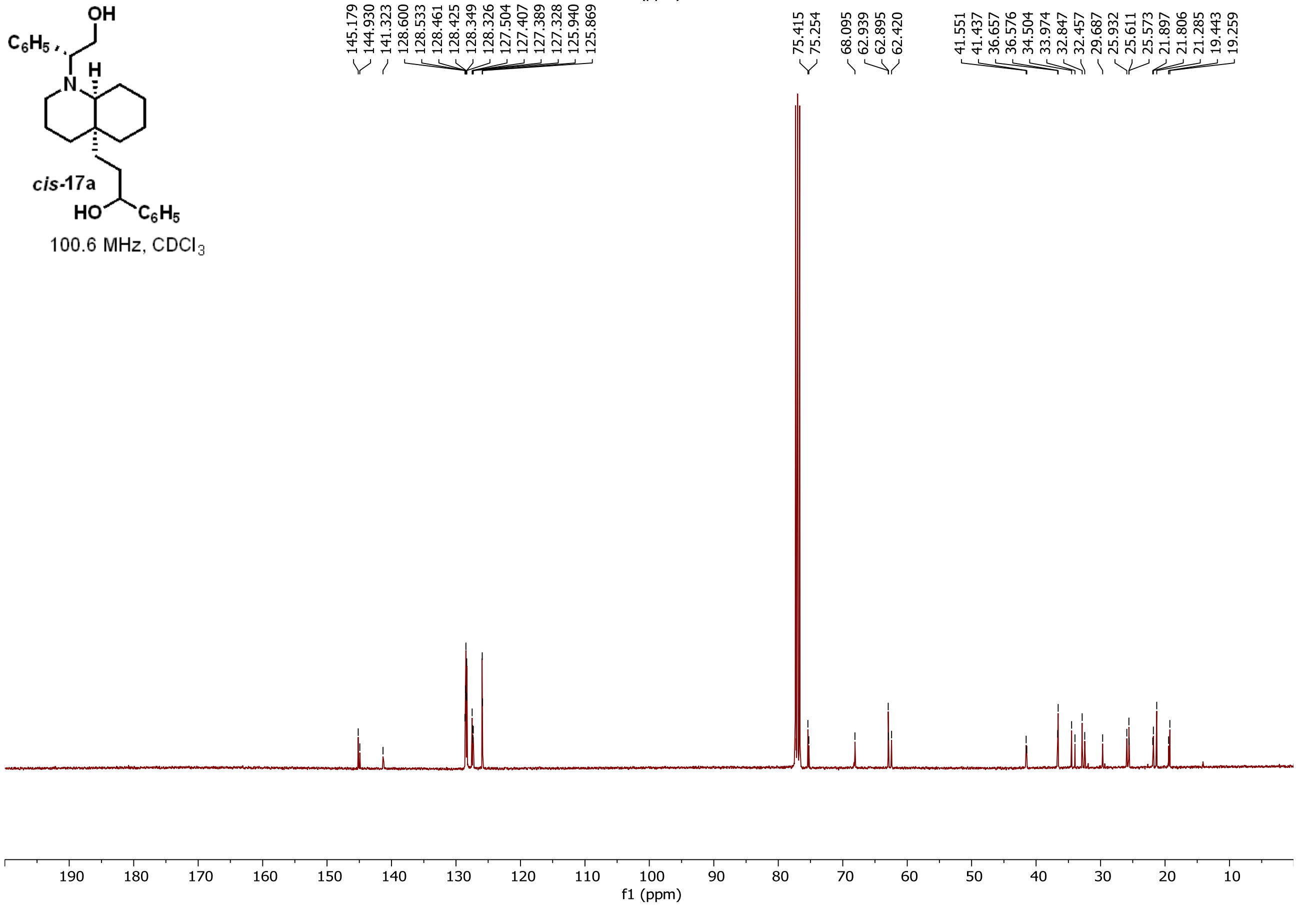

OH
C6H5
N
H
cis-17a
HO
C6H5
100.6 MHz, CDCl3
145.179
144.930
141.323
128.600
128.533
128.461
128.425
128.349
128.326
127.504
127.407
127.389
127.328
125.940
125.869
75.415
75.254
68.095
62.939
62.895
62.420
41.551
41.437
36.657
36.576
34.504
33.974
32.847
32.457
29.687
25.932
25.611
25.573
21.897
21.806
21.285
19.443
19.259
f1 (ppm)

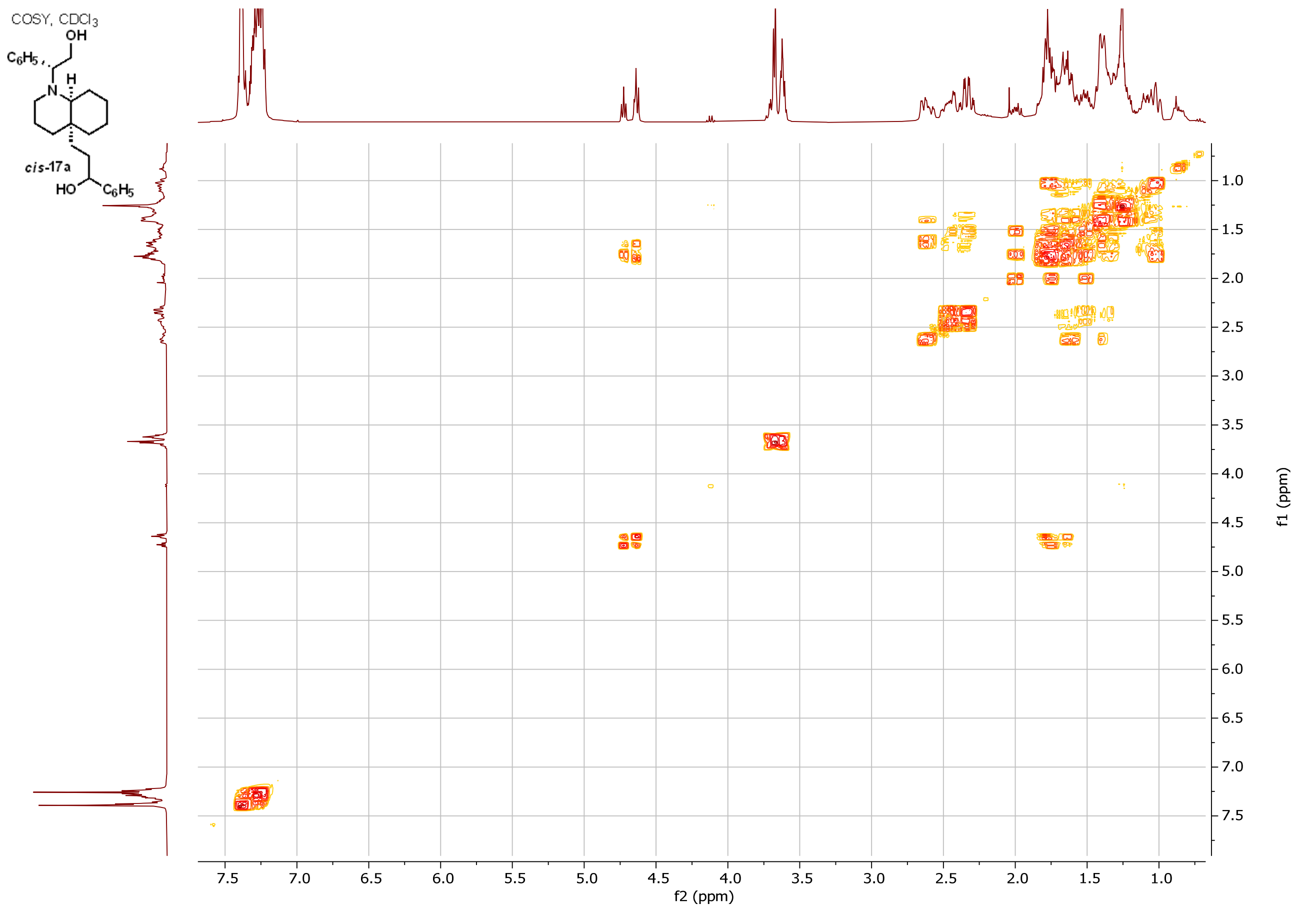
COSY, CDCl3
OH
C6H5
N
H
cis-17a
HO
C6H5
1.0
1.5
2.0
2.5
3.0
3.5
4.0
4.5
5.0
5.5
6.0
6.5
7.0
7.5
f1 (ppm)
7.5 7.0 6.5 6.0 5.5 5.0 4.5 4.0 3.5 3.0 2.5 2.0 1.5 1.0
f2 (ppm)

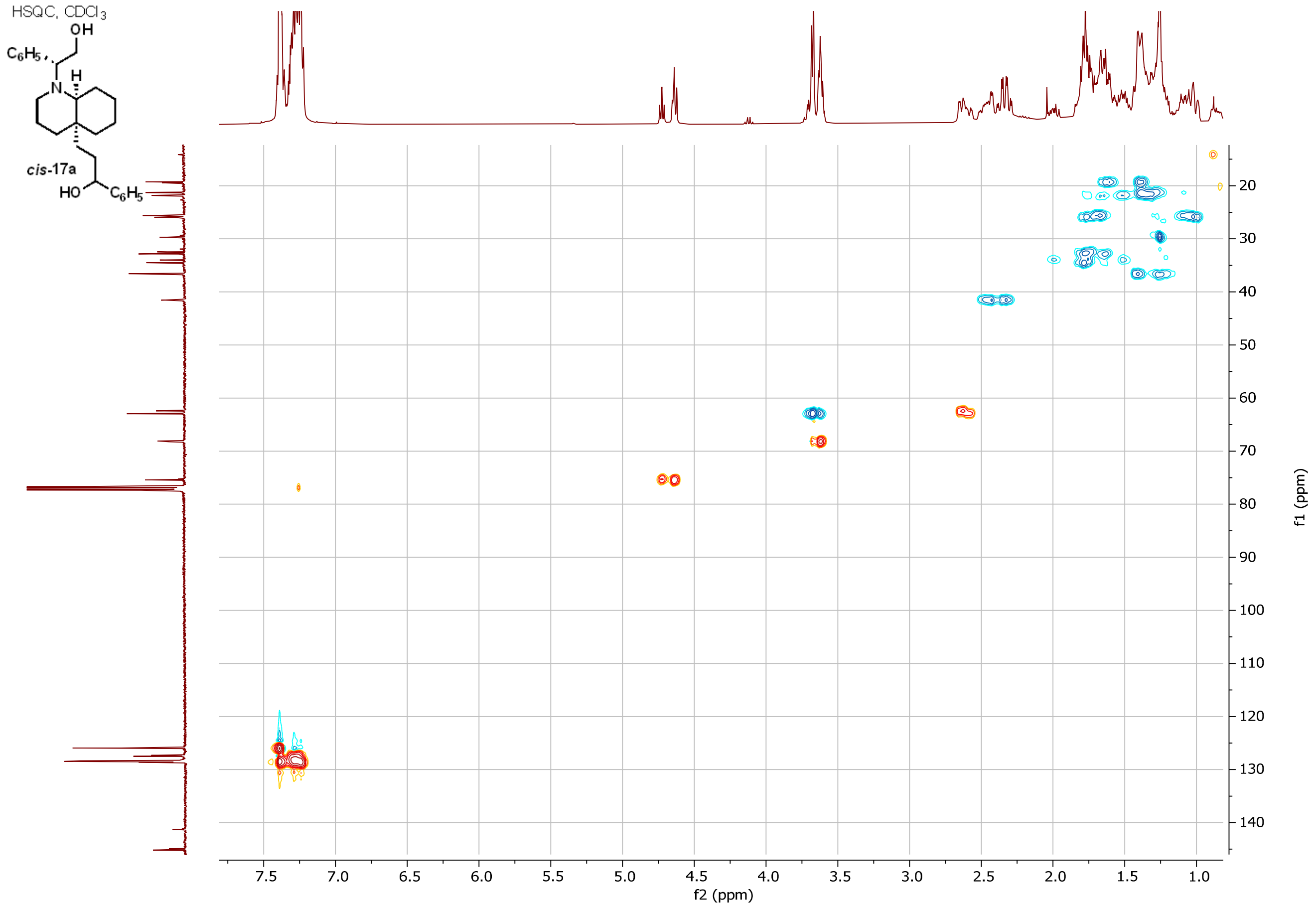
HSQC, CDCl3
OH
C6H5
N
H
cis-17a
HO
C6H5
20
30
40
50
60
70
80
90
100
110
120
130
140
f1 (ppm)
7.5 7.0 6.5 6.0 5.5 5.0 4.5 4.0 3.5 3.0 2.5 2.0 1.5 1.0
f2 (ppm)

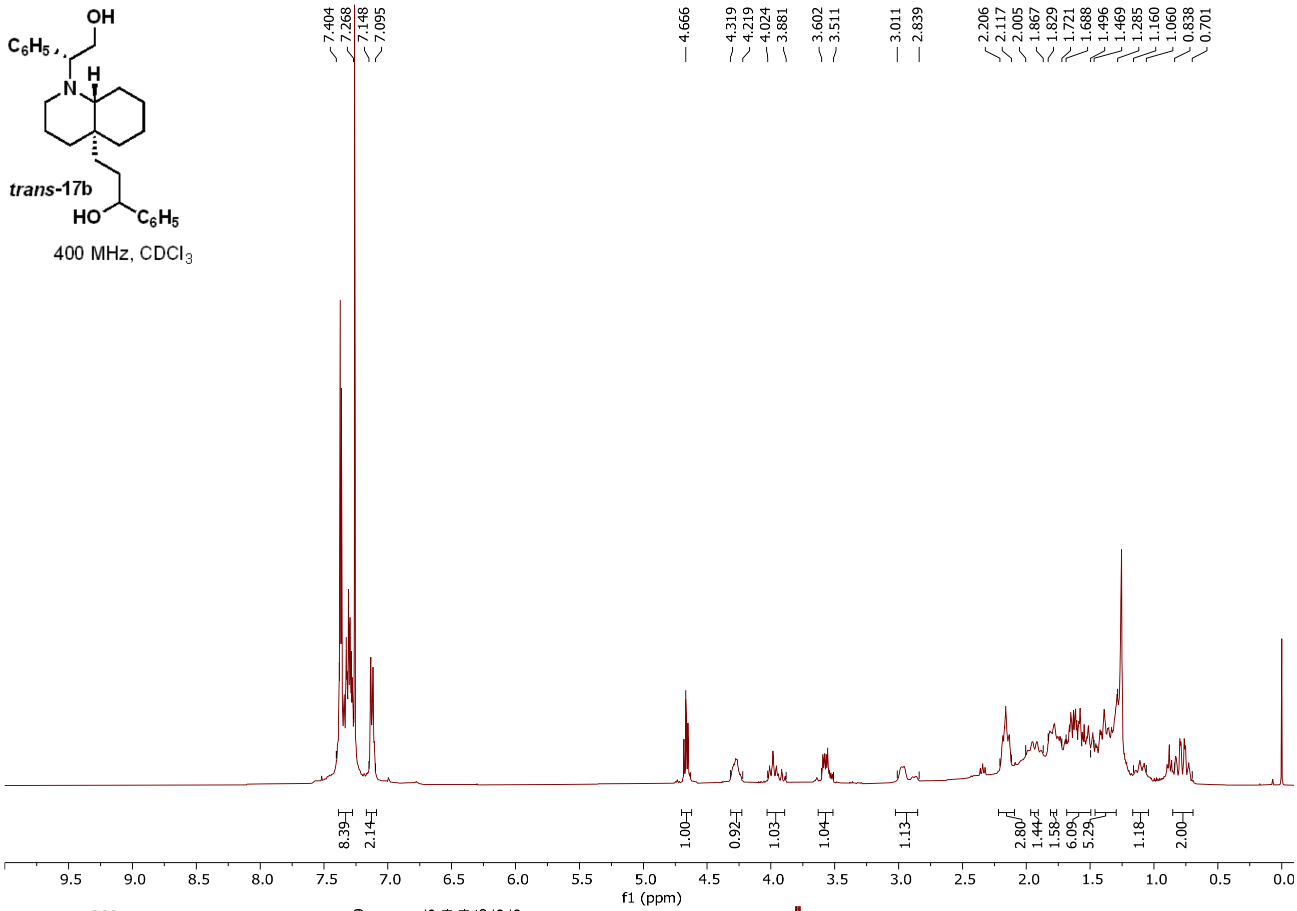

trans-**17b**

400 MHz, $CDCl_3$

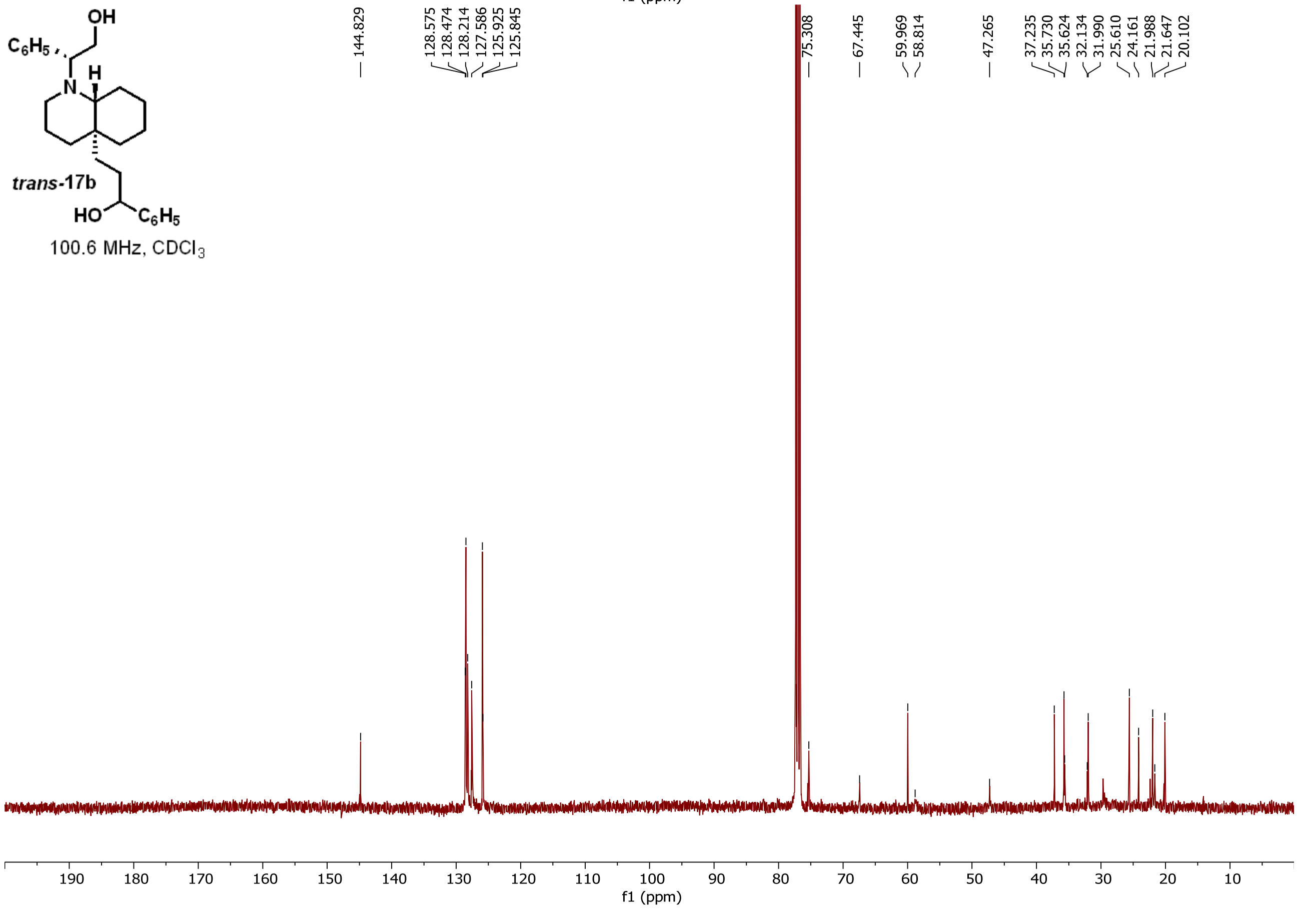

trans-**17b**

100.6 MHz, $CDCl_3$

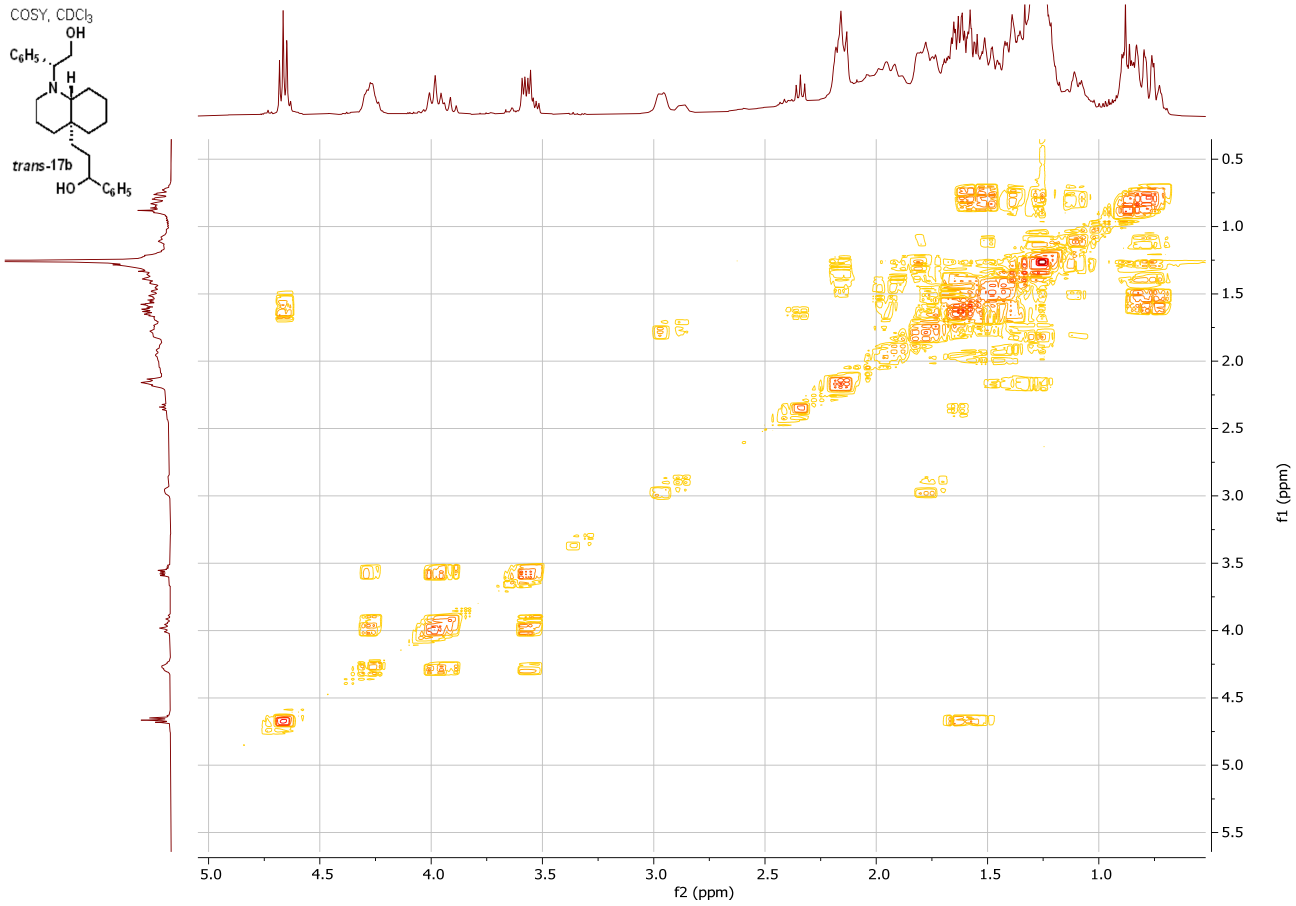
COSY, CDCl3
OH
C6H5
N
H
trans-17b
HO
C6H5
f2 (ppm)
f1 (ppm)

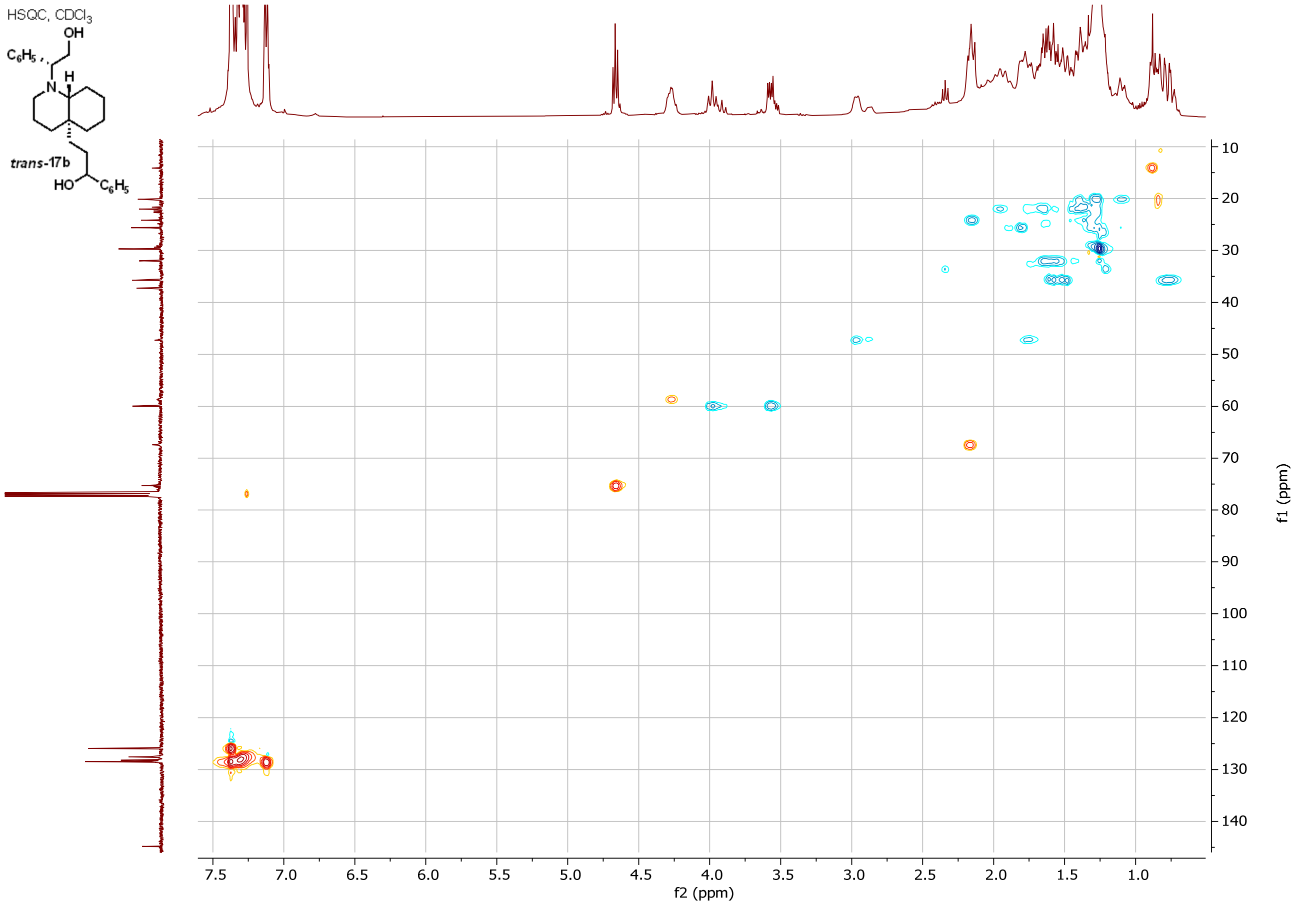
HSQC, CDCl3
OH
C6H5
N
H
trans-17b
HO
C6H5
f2 (ppm)
f1 (ppm)

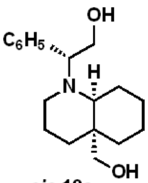

cis-**18a**

400 MHz, $CDCl_3$

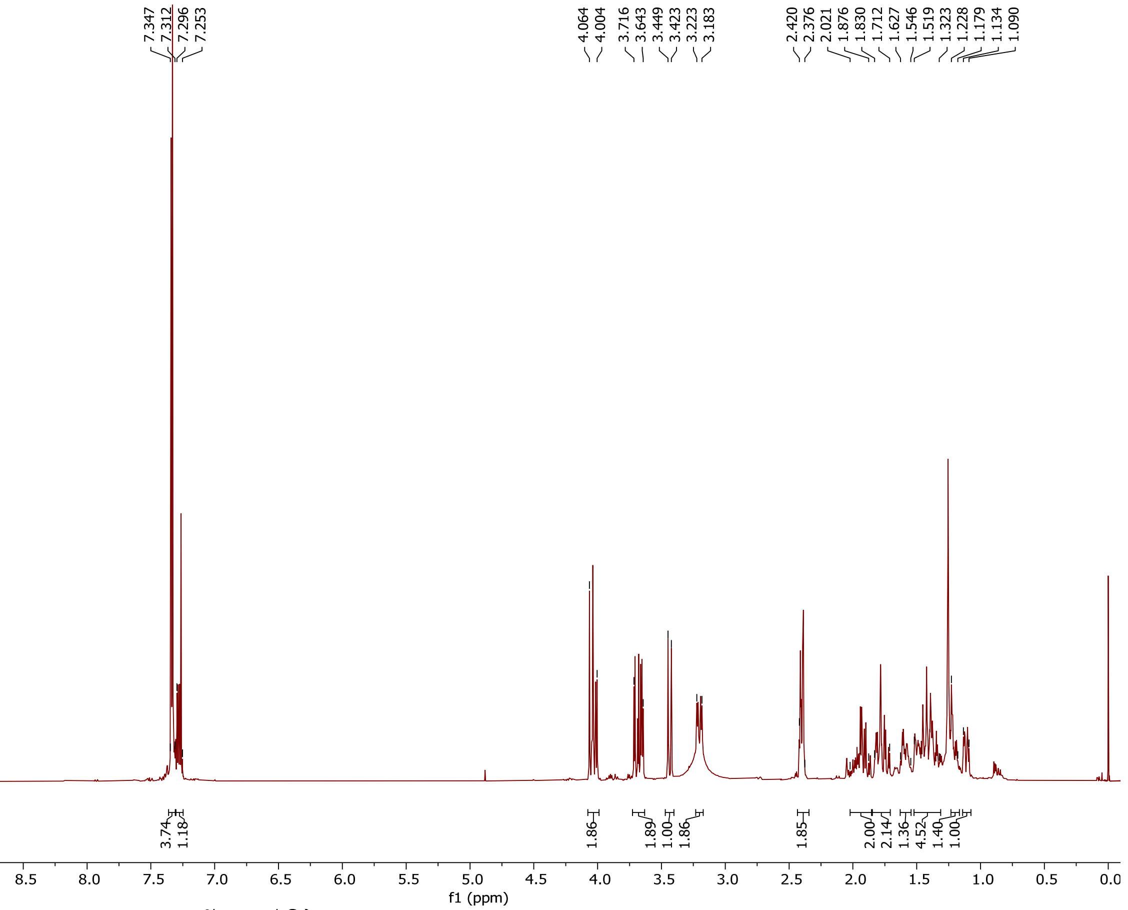

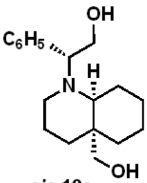

cis-**18a**

100.6 MHz, $CDCl_3$

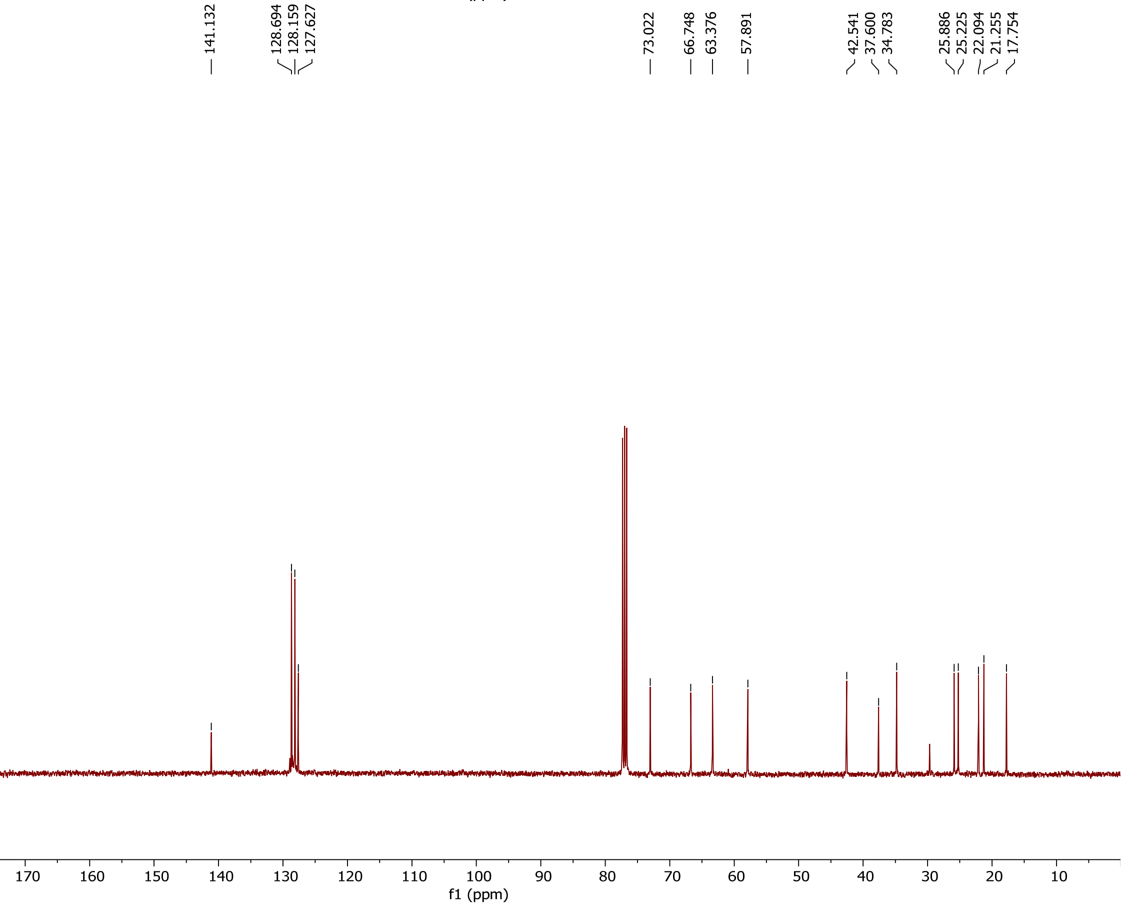

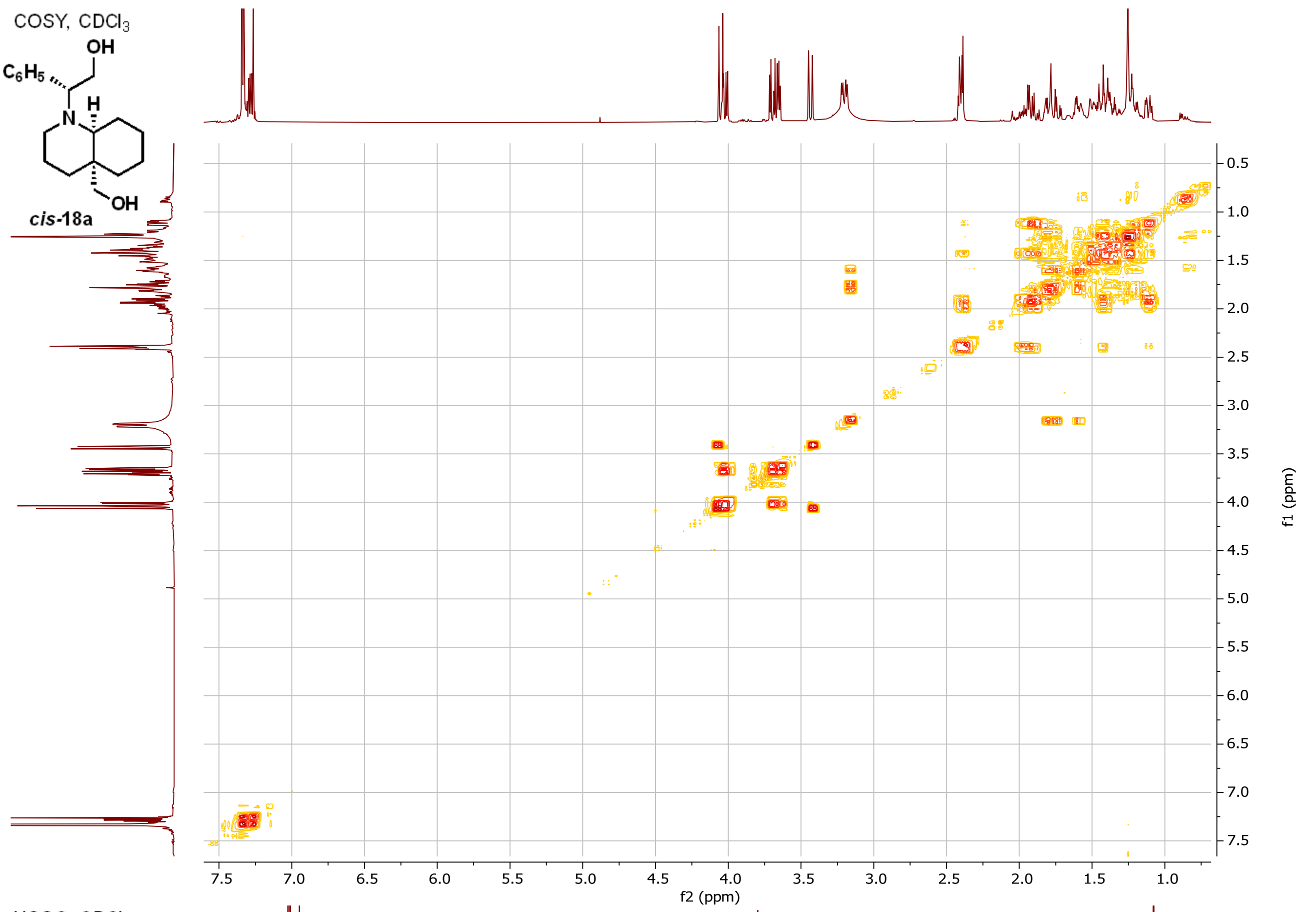
COSY, $CDCl_3$
OH
C_6H_5
H
N
OH
cis-18a
f2 (ppm)
f1 (ppm)

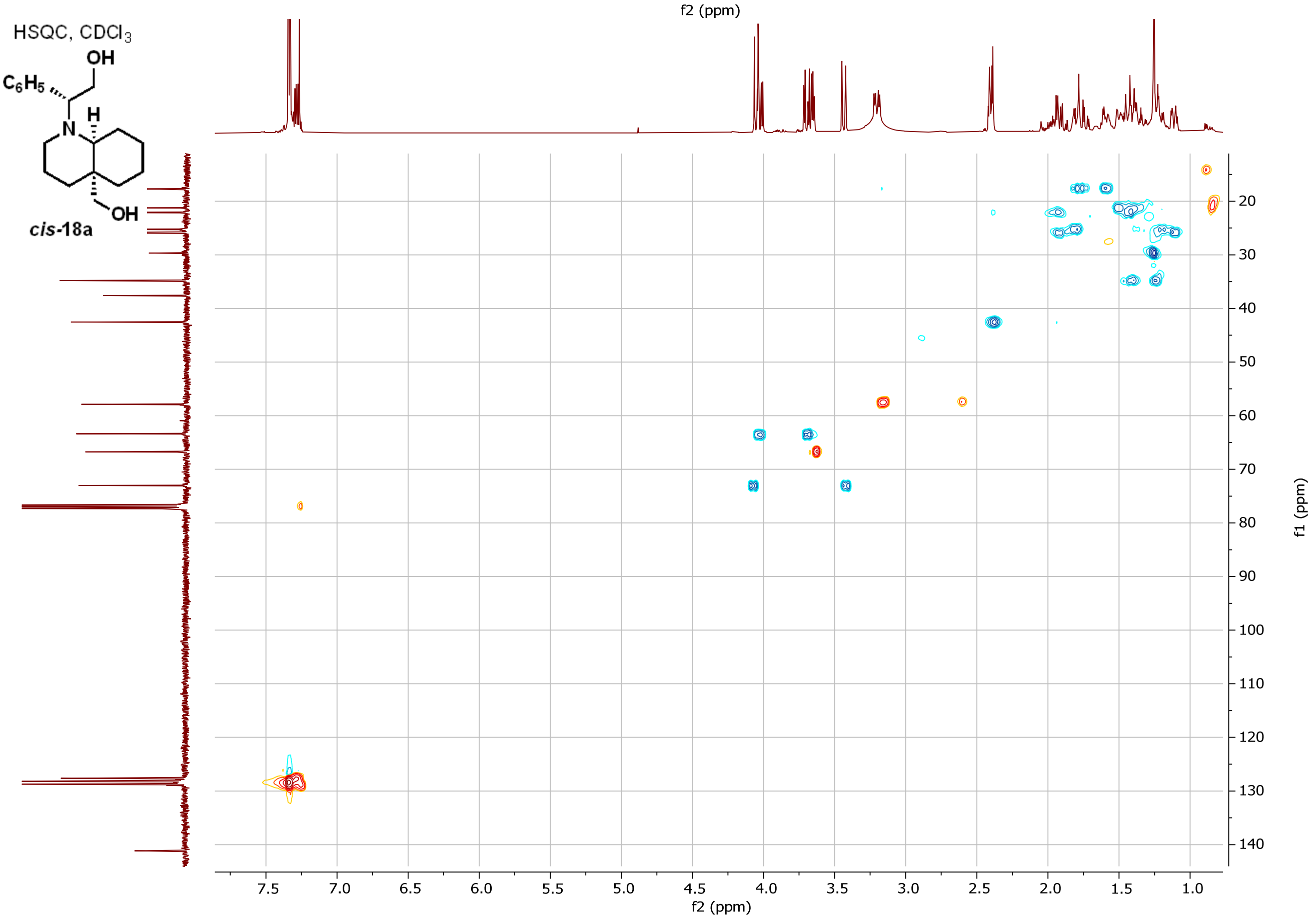
HSQC, $CDCl_3$
OH
C_6H_5
H
N
OH
cis-18a
f2 (ppm)
f1 (ppm)

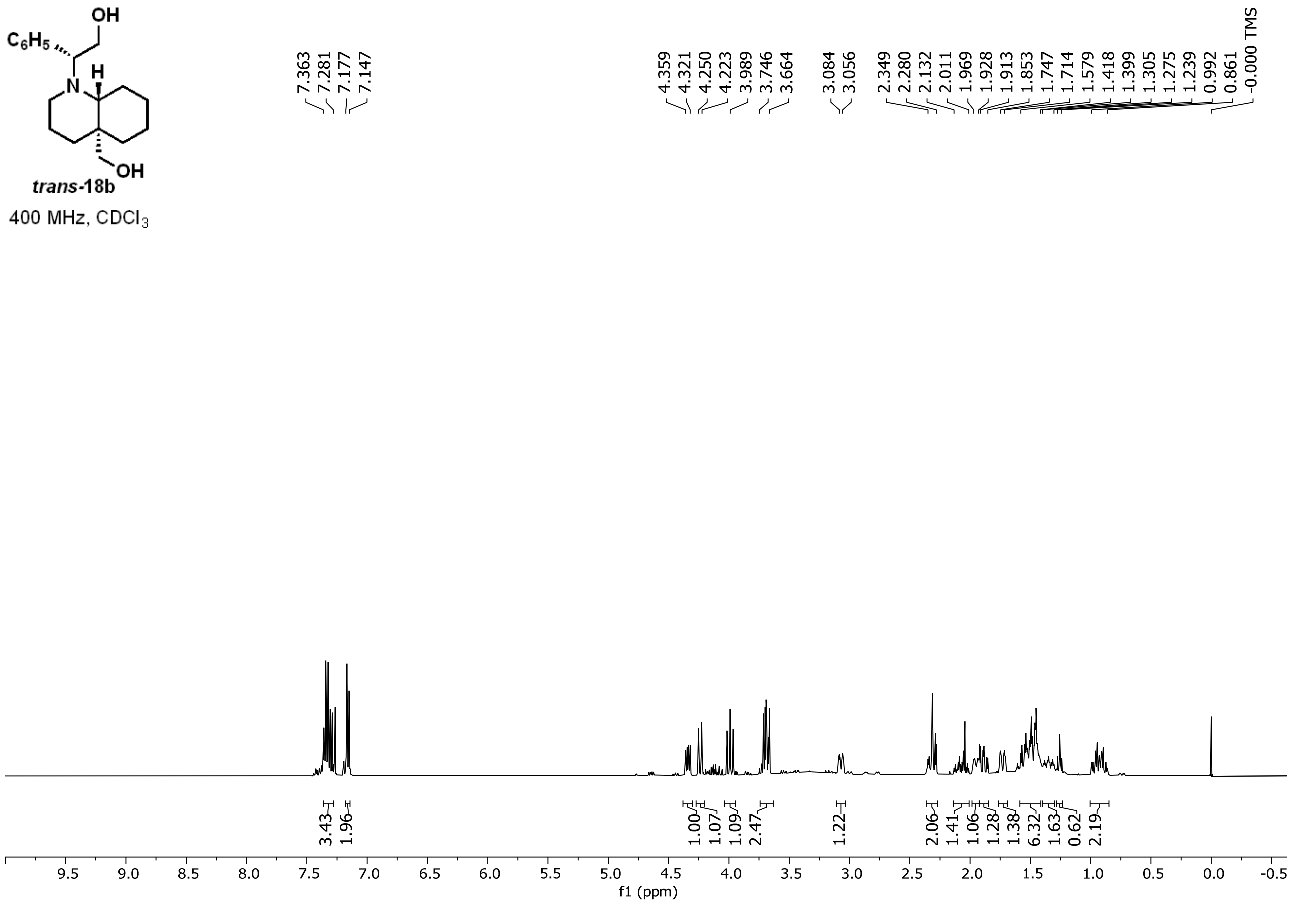
OH
C6H5
N
H
OH
trans-18b
400 MHz, CDCl3
7.363
7.281
7.177
7.147
4.359
4.321
4.250
4.223
3.989
3.746
3.664
3.084
3.056
2.349
2.280
2.132
2.011
1.969
1.928
1.913
1.853
1.747
1.714
1.579
1.418
1.399
1.305
1.275
1.239
0.992
0.861
-0.000 TMS
3.43
1.96
1.00
1.07
1.09
2.47
1.22
2.06
1.41
1.06
1.28
1.38
6.32
1.63
0.62
2.19
9.5 9.0 8.5 8.0 7.5 7.0 6.5 6.0 5.5 5.0 4.5 4.0 3.5 3.0 2.5 2.0 1.5 1.0 0.5 0.0 -0.5
f1 (ppm)

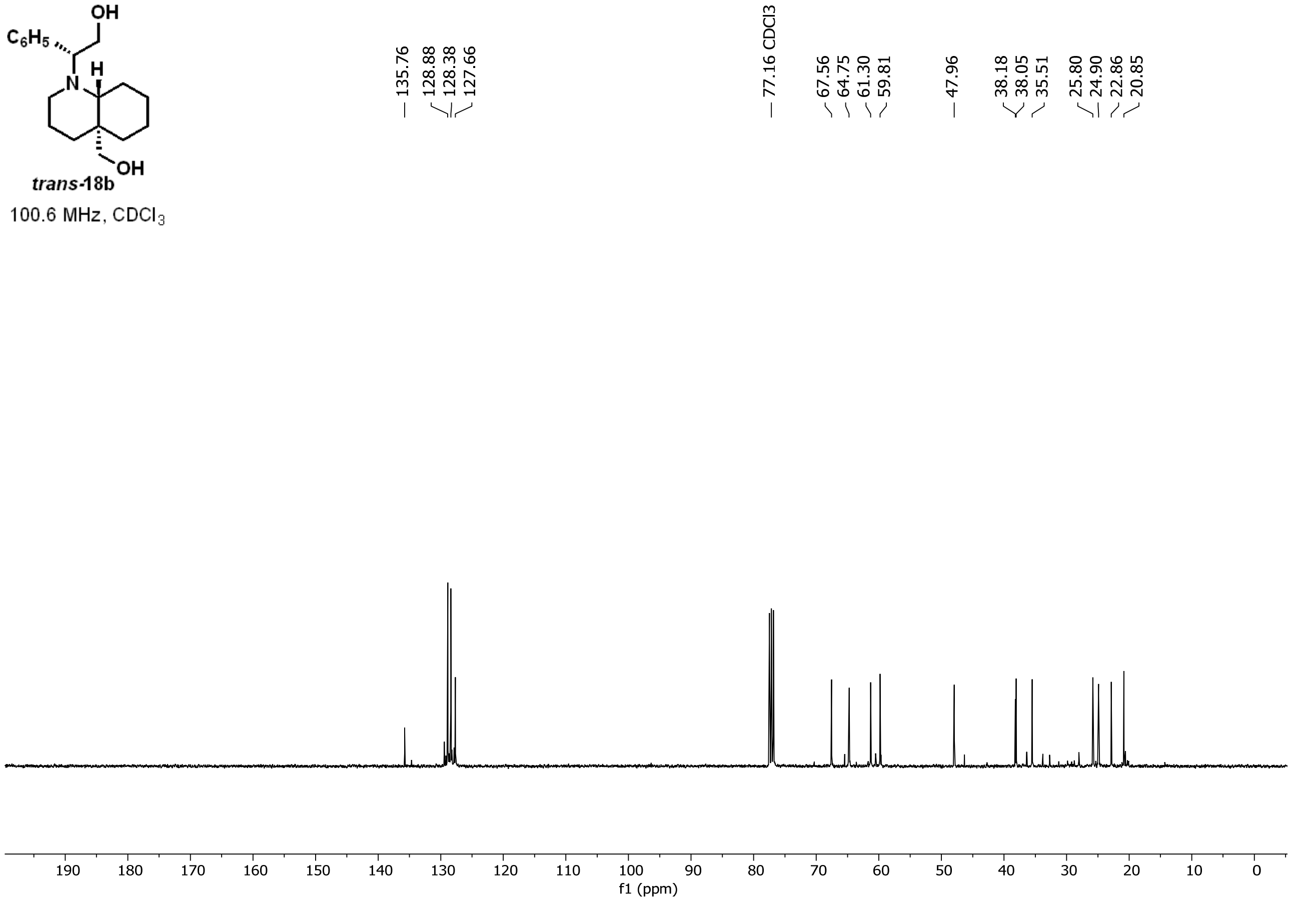
OH
C6H5
N
H
OH
trans-18b
100.6 MHz, CDCl3
135.76
128.88
128.38
127.66
77.16 CDCl3
67.56
64.75
61.30
59.81
47.96
38.18
38.05
35.51
25.80
24.90
22.86
20.85
190 180 170 160 150 140 130 120 110 100 90 80 70 60 50 40 30 20 10 0
f1 (ppm)

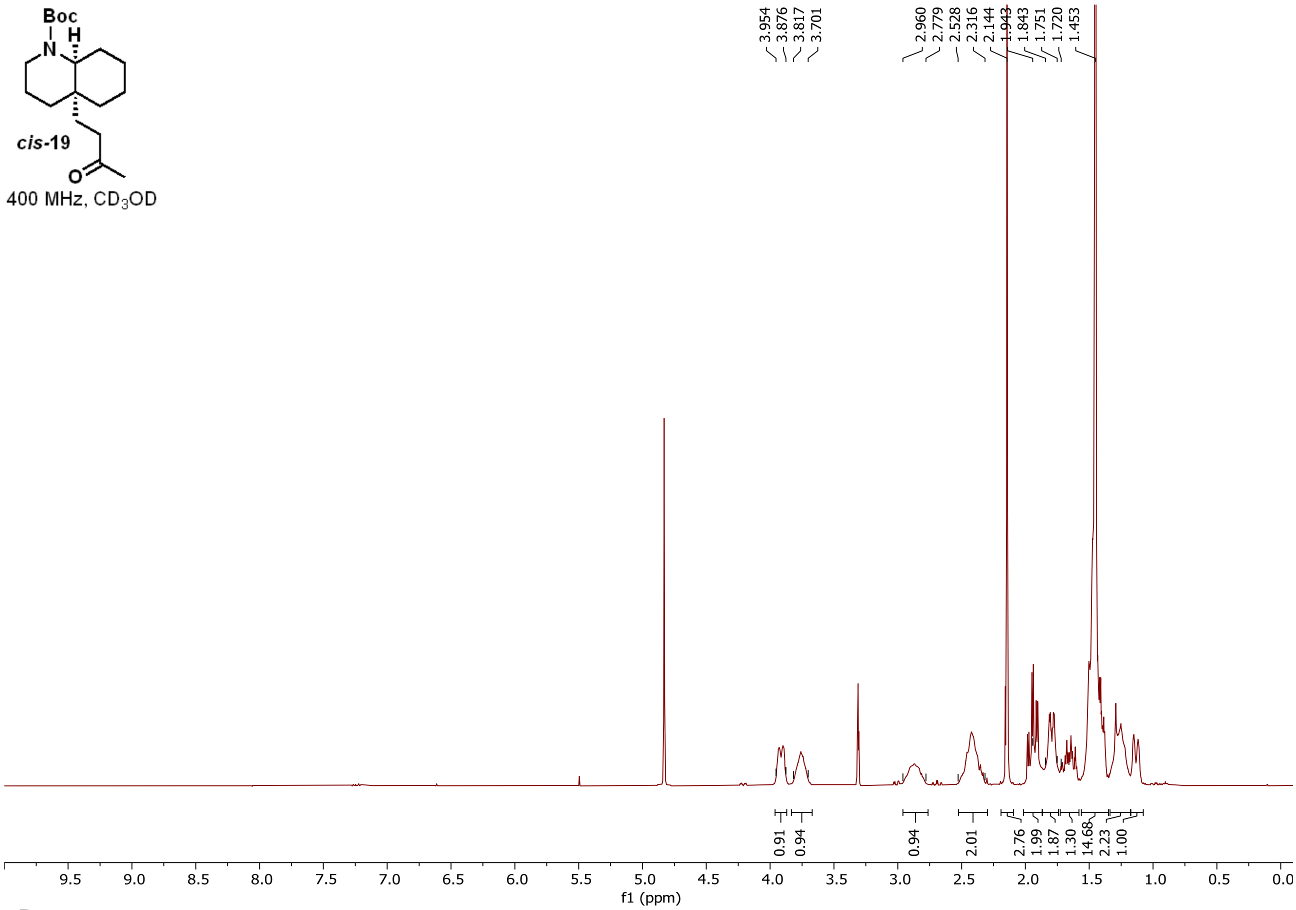
Boc
N
H
cis-19
O
400 MHz, CD3OD
3.954
3.876
3.817
3.701
2.960
2.779
2.528
2.316
2.144
1.943
1.843
1.751
1.720
1.453
0.91
0.94
0.94
2.01
2.76
1.99
1.87
1.30
14.68
2.23
1.00
9.5 9.0 8.5 8.0 7.5 7.0 6.5 6.0 5.5 5.0 4.5 4.0 3.5 3.0 2.5 2.0 1.5 1.0 0.5 0.0
f1 (ppm)

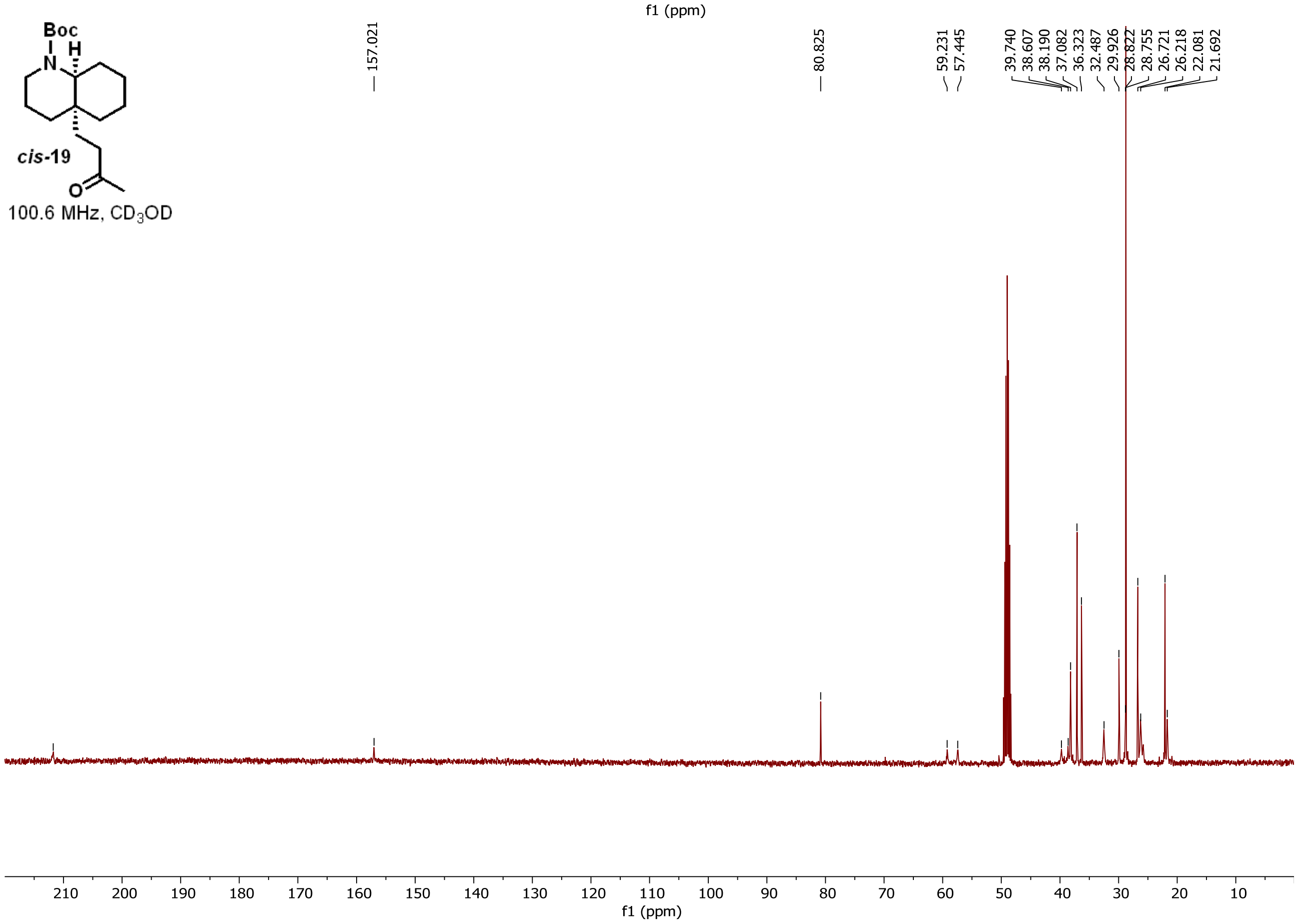
Boc
N
H
cis-19
O
100.6 MHz, CD3OD
157.021
80.825
59.231
57.445
39.740
38.607
38.190
37.082
36.323
32.487
29.926
28.822
28.755
26.721
26.218
22.081
21.692
210 200 190 180 170 160 150 140 130 120 110 100 90 80 70 60 50 40 30 20 10
f1 (ppm)

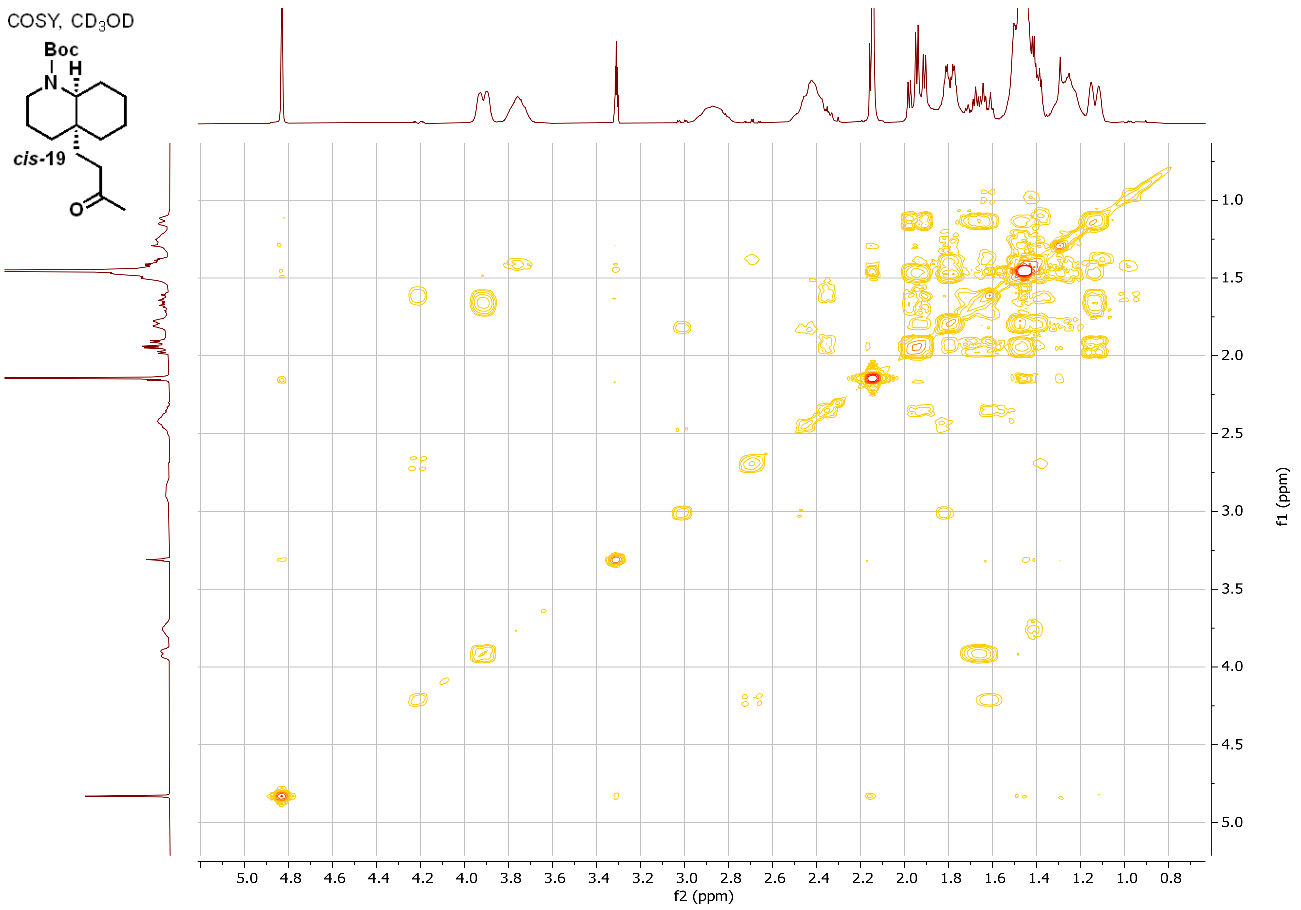
COSY, CD_3OD
Boc
N
H
cis-19
O
f2 (ppm)
f1 (ppm)

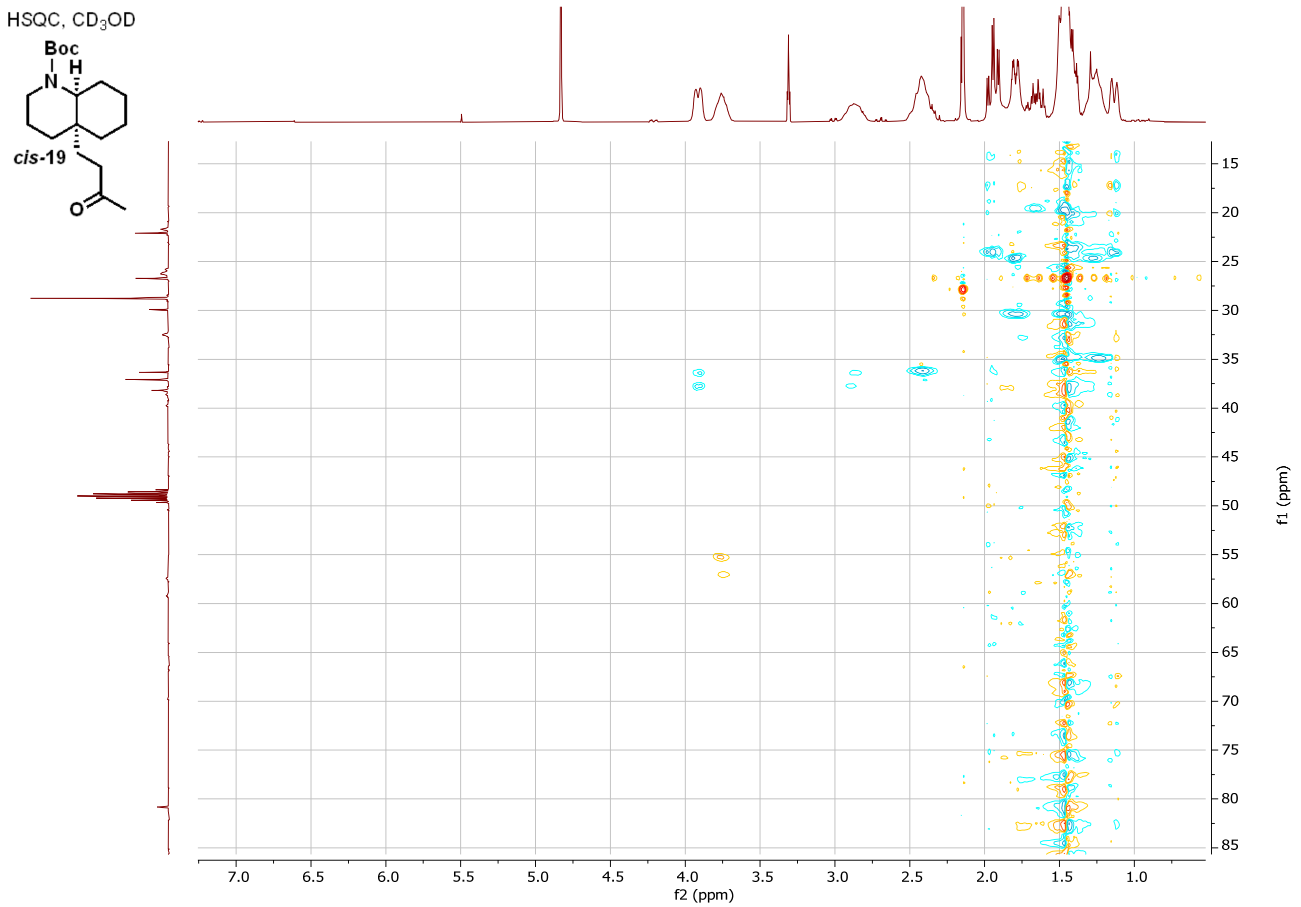
HSQC, CD_3OD
Boc
N
H
cis-19
O
f2 (ppm)
f1 (ppm)

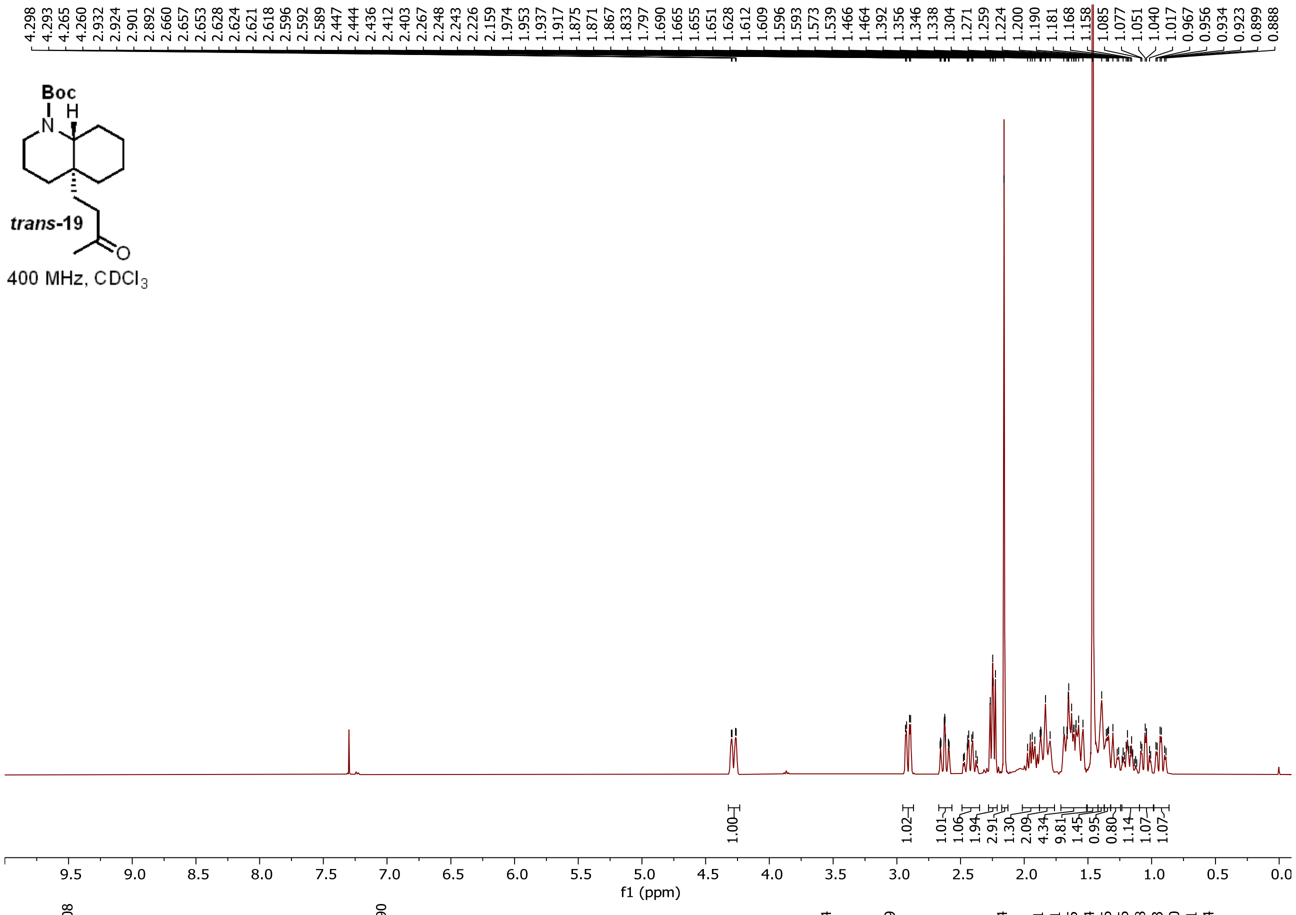

4.298 4.293 4.265 4.260 2.932 2.924 2.901 2.892 2.660 2.657 2.653 2.628 2.624 2.621 2.618 2.596 2.592 2.589 2.447 2.444 2.436 2.412 2.403 2.267 2.248 2.243 2.226 2.159 1.974 1.953 1.937 1.917 1.875 1.871 1.867 1.833 1.797 1.690 1.665 1.655 1.651 1.628 1.612 1.609 1.596 1.593 1.573 1.539 1.466 1.464 1.392 1.356 1.346 1.338 1.304 1.271 1.259 1.224 1.200 1.190 1.181 1.168 1.158 1.085 1.077 1.051 1.040 1.017 0.967 0.956 0.934 0.923 0.899 0.888
Boc
N
H
trans-19
O
400 MHz, CDCl3
1.00
1.02
1.01 1.06 1.94 2.91 1.30 2.09 4.34 9.81 1.45 0.95 0.80 1.14 1.07 1.07
9.5 9.0 8.5 8.0 7.5 7.0 6.5 6.0 5.5 5.0 4.5 4.0 3.5 3.0 2.5 2.0 1.5 1.0 0.5 0.0
f1 (ppm)

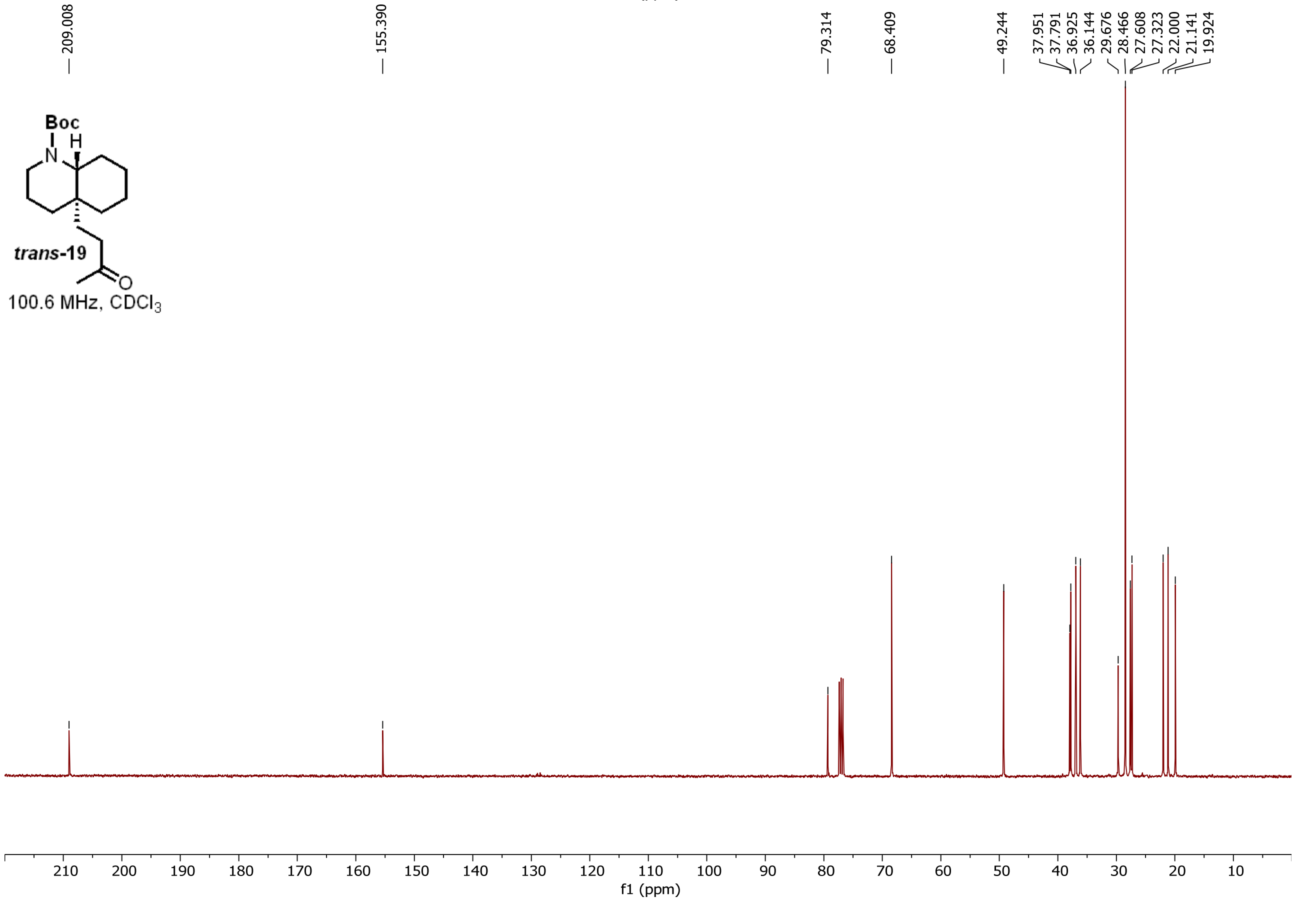

209.008
155.390
79.314
68.409
49.244
37.951 37.791 36.925 36.144 29.676 28.466 27.608 27.323 22.000 21.141 19.924
Boc
N
H
trans-19
O
100.6 MHz, CDCl3
210 200 190 180 170 160 150 140 130 120 110 100 90 80 70 60 50 40 30 20 10
f1 (ppm)

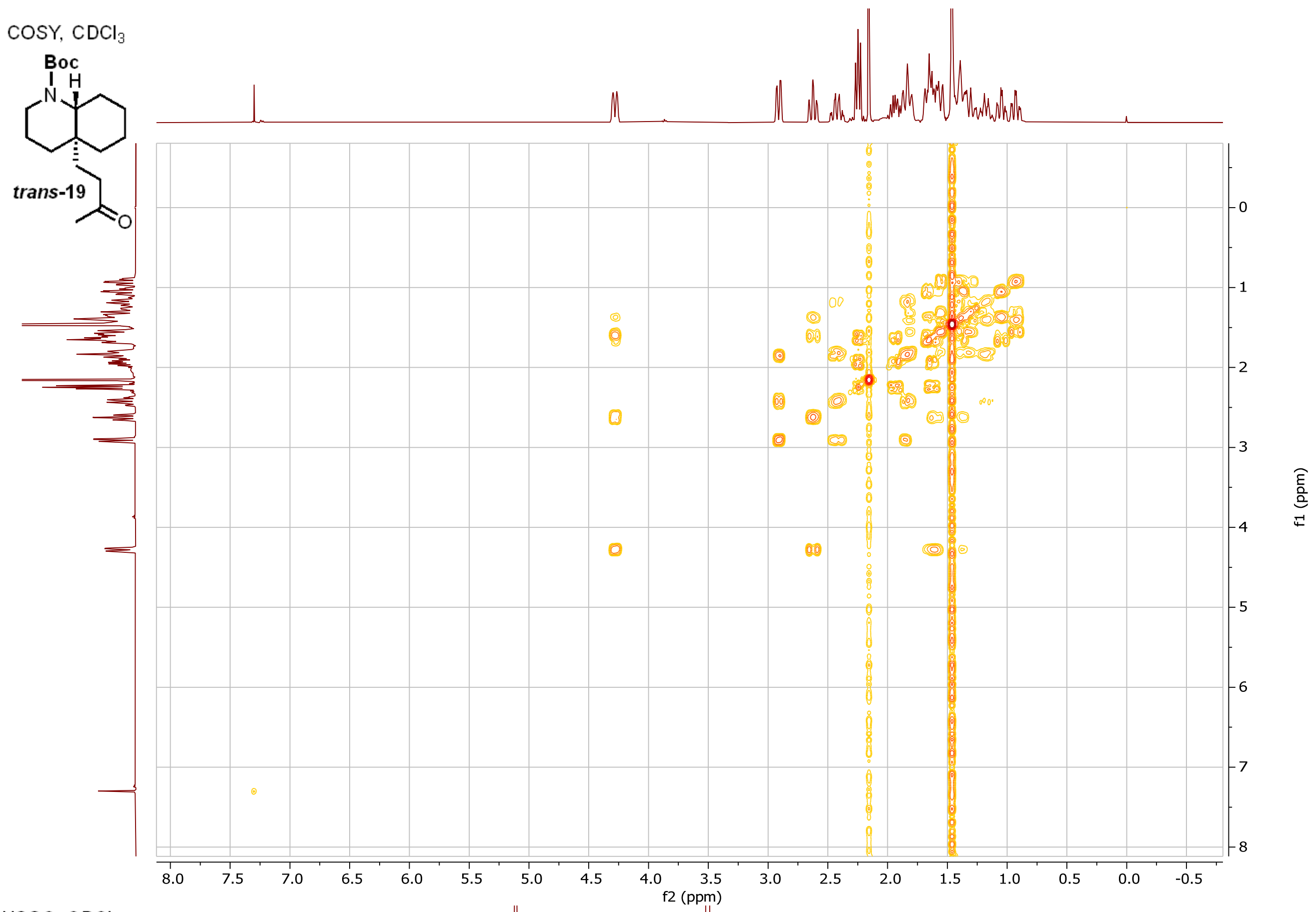
COSY, CDCl3
Boc
N
H
trans-19
O
f2 (ppm)
f1 (ppm)

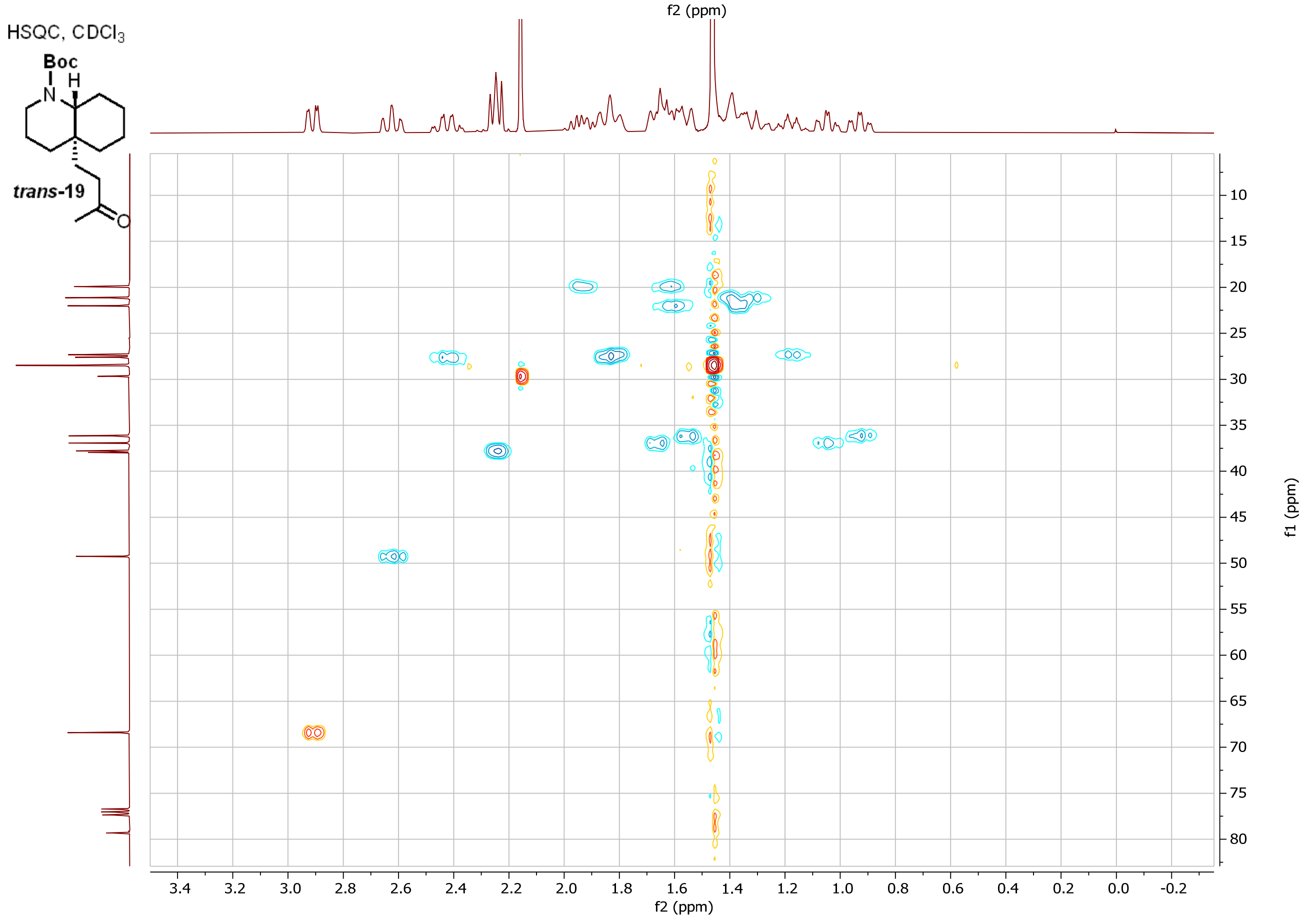
HSQC, CDCl3
Boc
N
H
trans-19
O
f2 (ppm)
f1 (ppm)

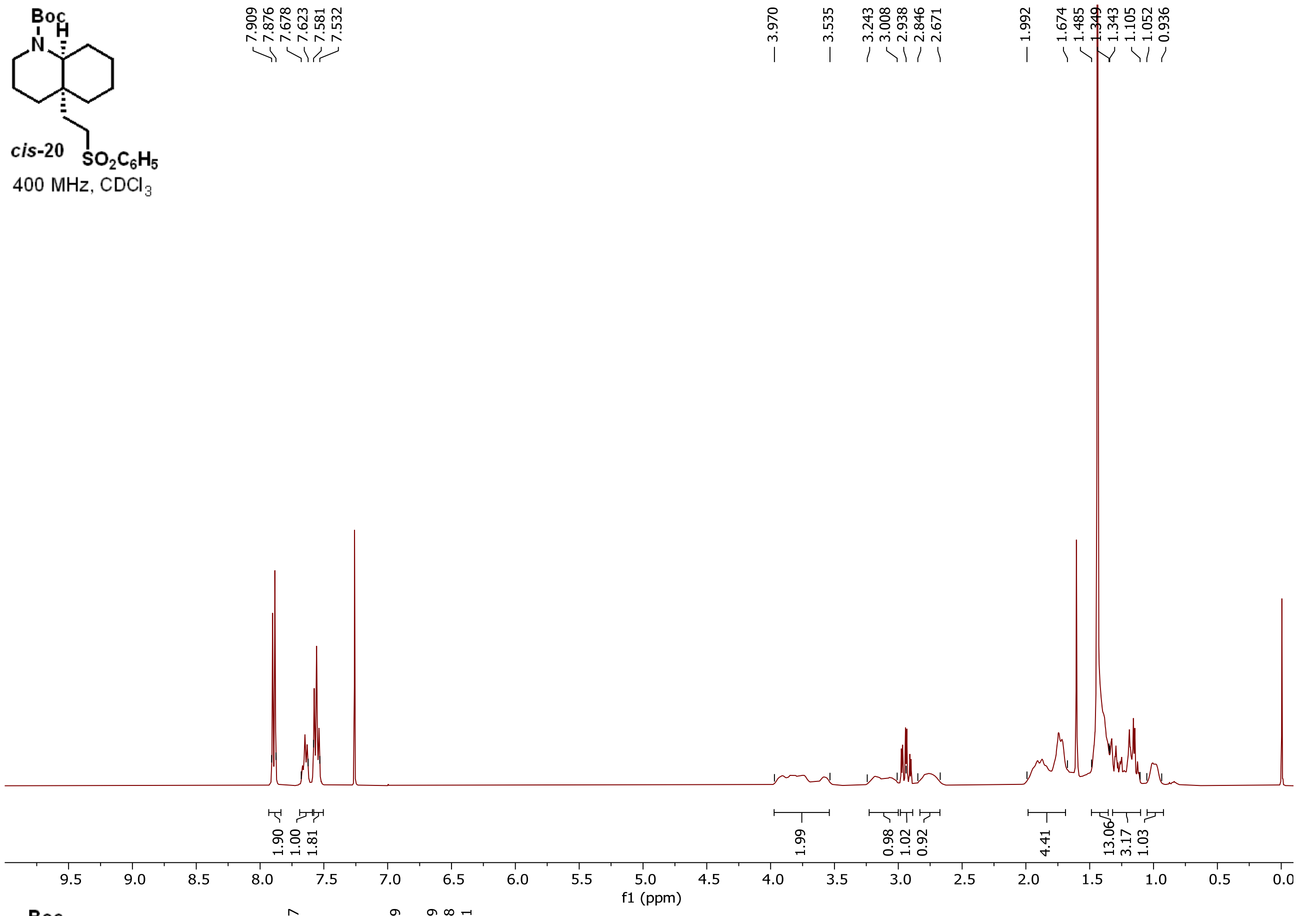
Boc
N
H
cis-20
SO2C6H5
400 MHz, CDCl3
7.909
7.876
7.678
7.623
7.581
7.532
3.970
3.535
3.243
3.008
2.938
2.846
2.671
1.992
1.674
1.485
1.349
1.343
1.105
1.052
0.936
1.90
1.00
1.81
1.99
0.98
1.02
0.92
4.41
13.06
3.17
1.03
f1 (ppm)

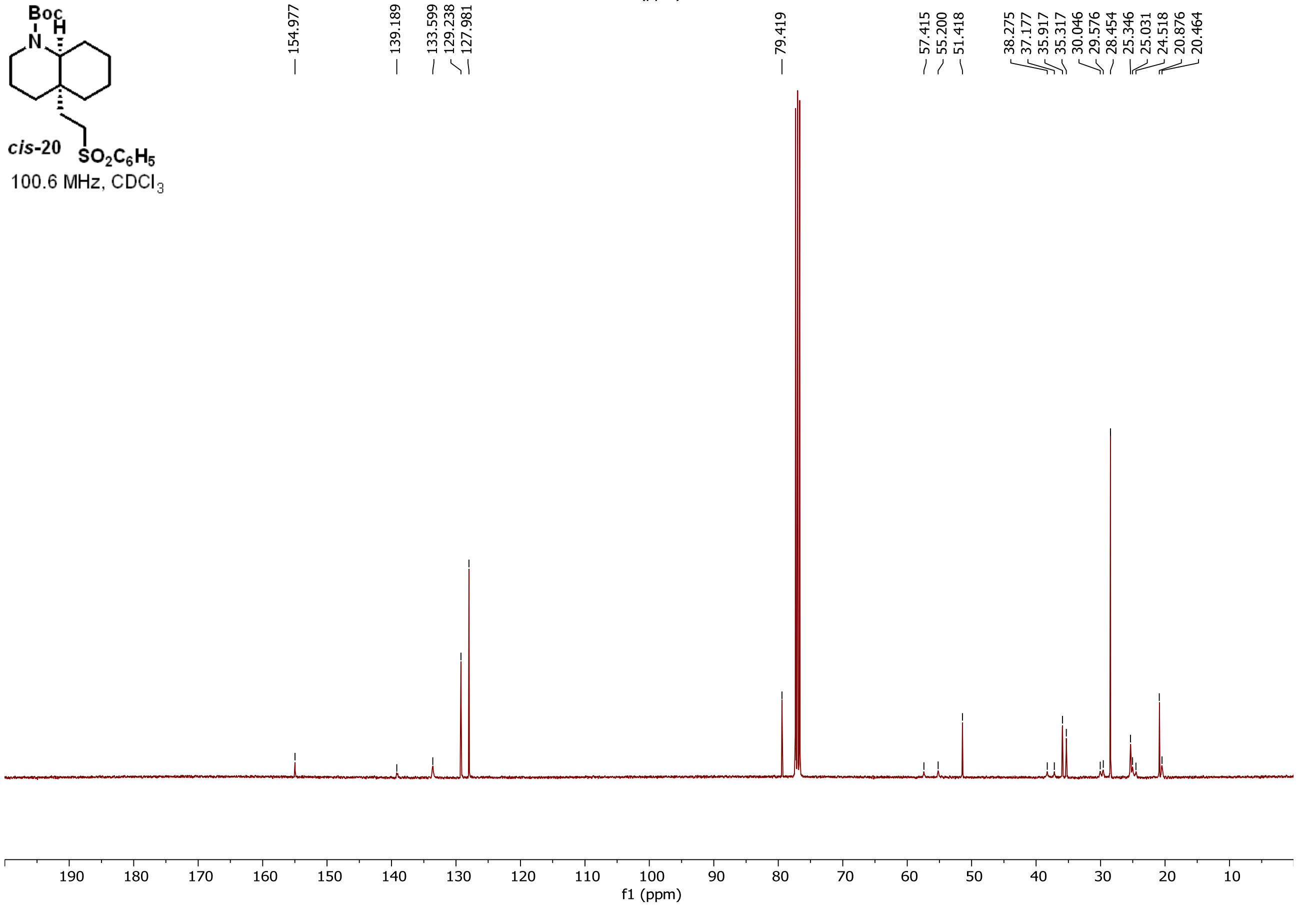
Boc
N
H
cis-20
SO2C6H5
100.6 MHz, CDCl3
154.977
139.189
133.599
129.238
127.981
79.419
57.415
55.200
51.418
38.275
37.177
35.917
35.317
30.046
29.576
28.454
25.346
25.031
24.518
20.876
20.464
f1 (ppm)

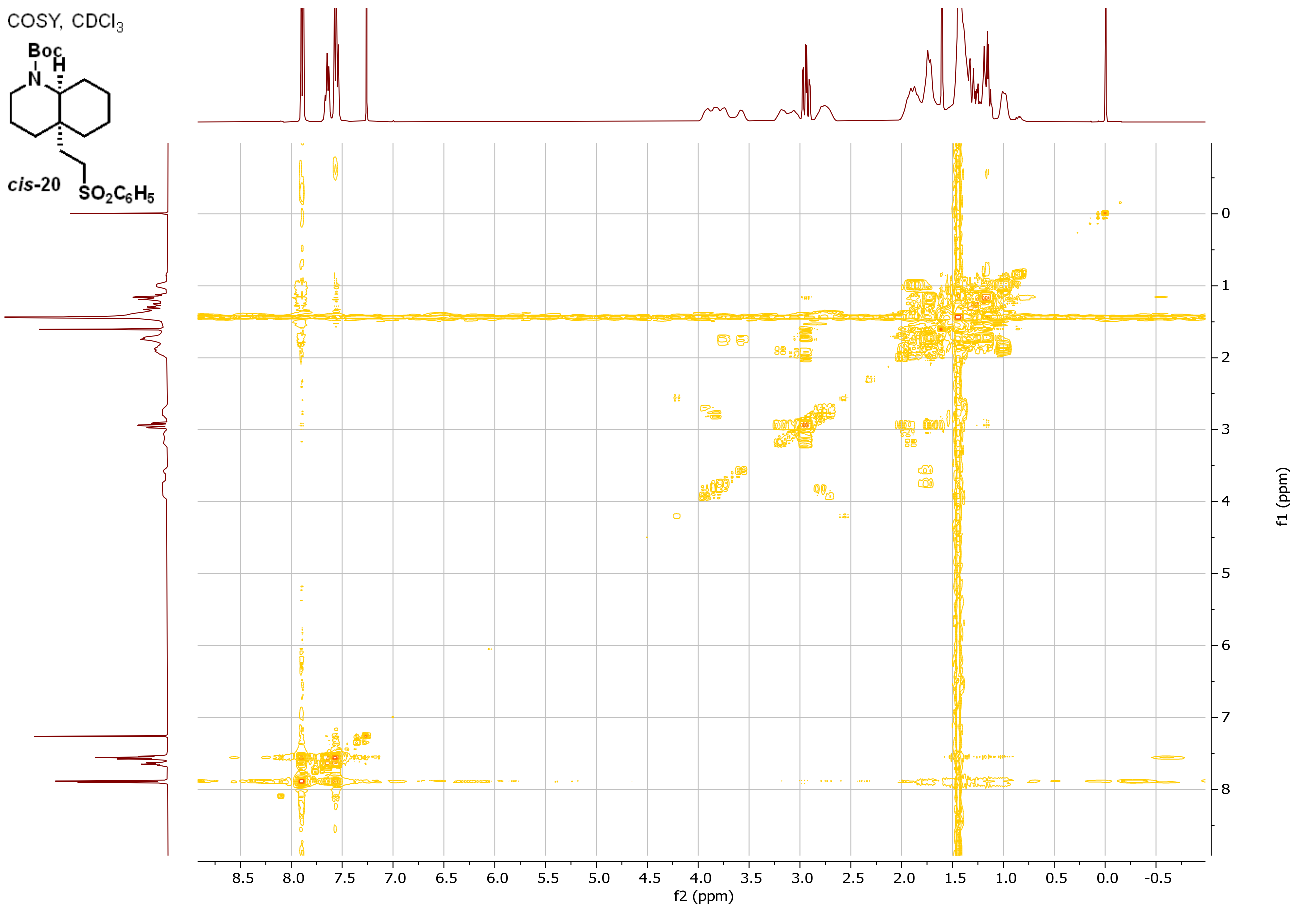
COSY, CDCl$_3$
Boc
N
H
cis-20
SO$_2$C$_6$H$_5$
f2 (ppm)
f1 (ppm)

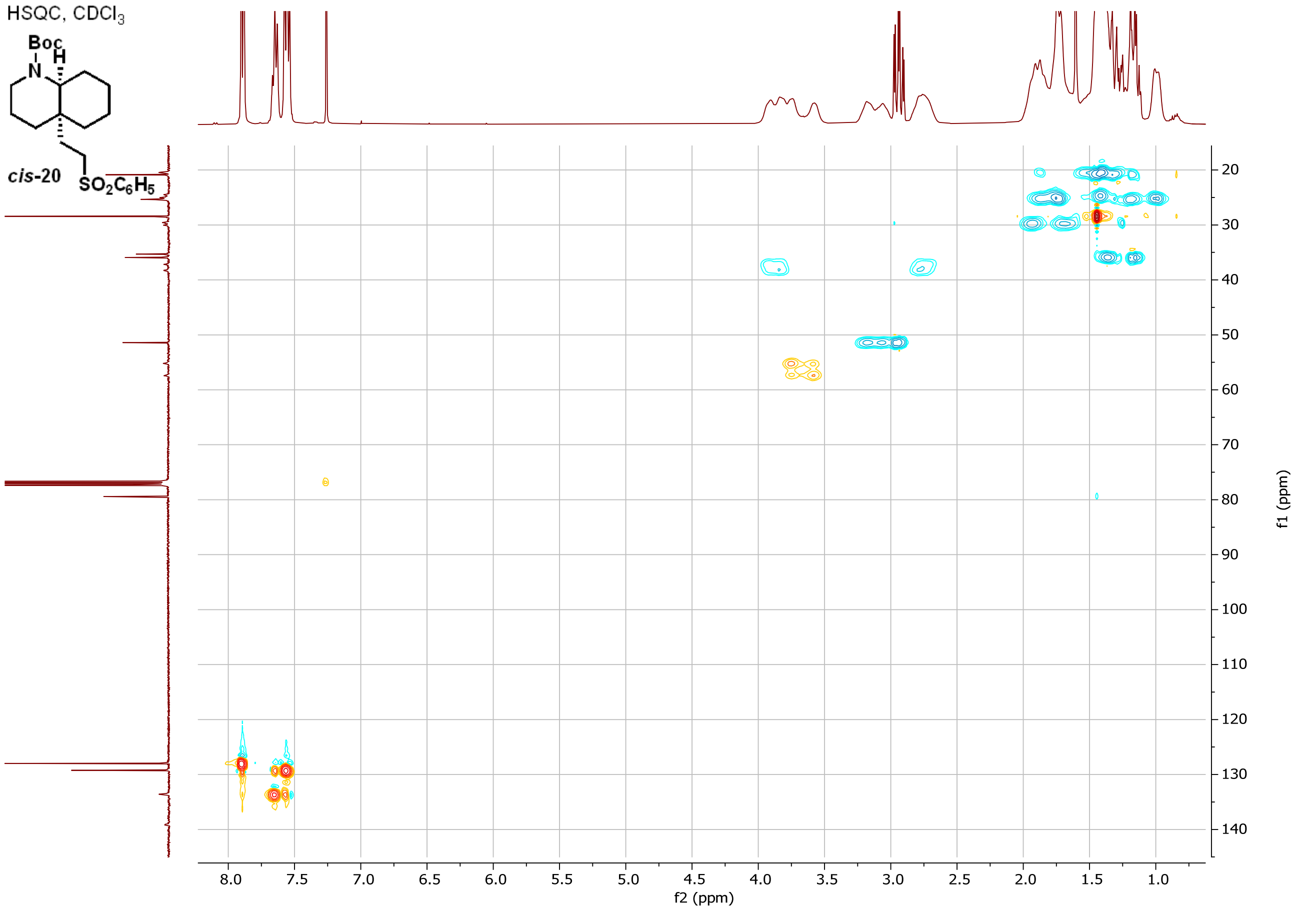
HSQC, CDCl$_3$
Boc
N
H
cis-20
SO$_2$C$_6$H$_5$
f2 (ppm)
f1 (ppm)

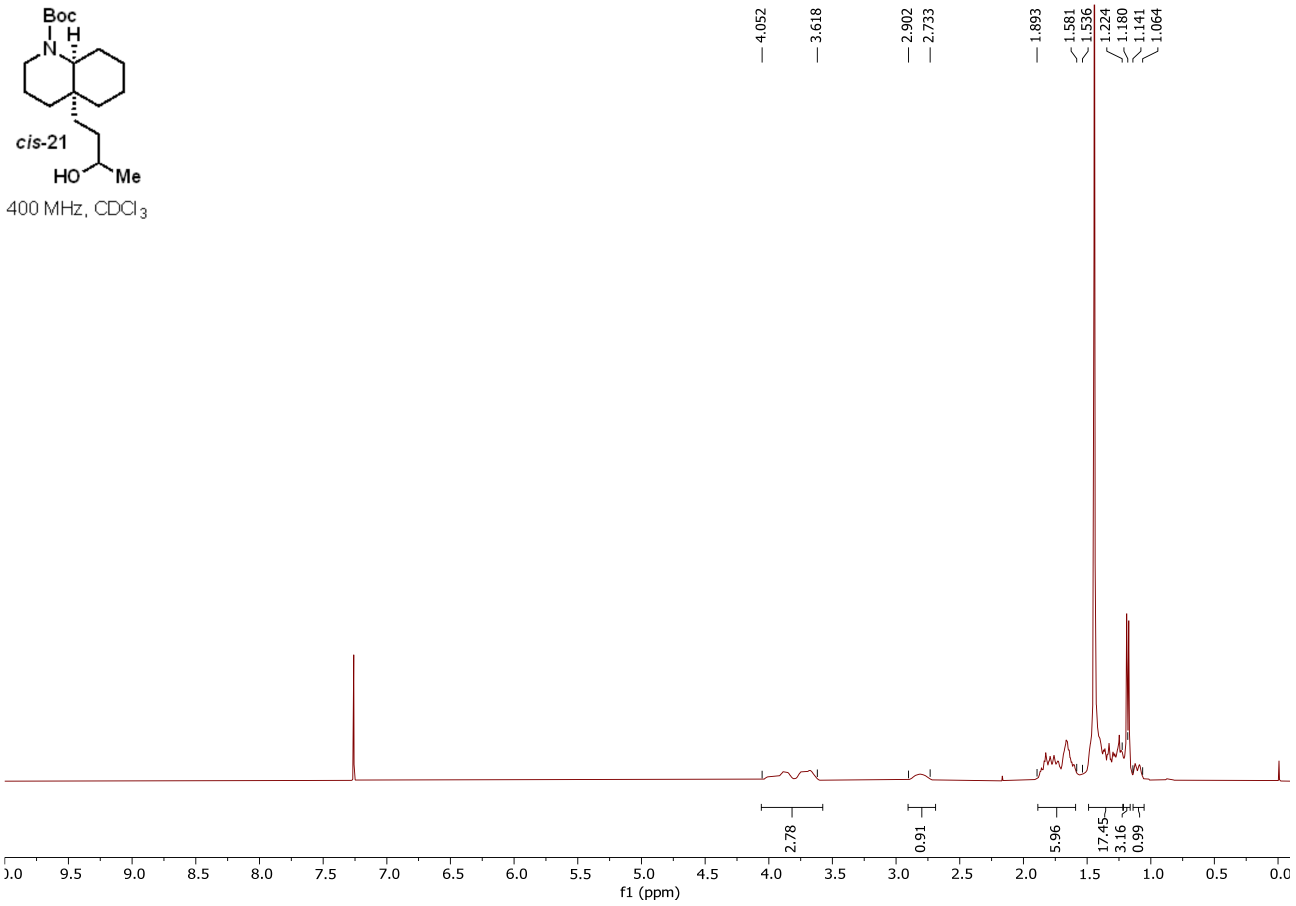
Boc
N
H
cis-21
HO
Me
400 MHz, CDCl3
4.052
3.618
2.902
2.733
1.893
1.581
1.536
1.224
1.180
1.141
1.064
2.78
0.91
5.96
17.45
3.16
0.99
f1 (ppm)

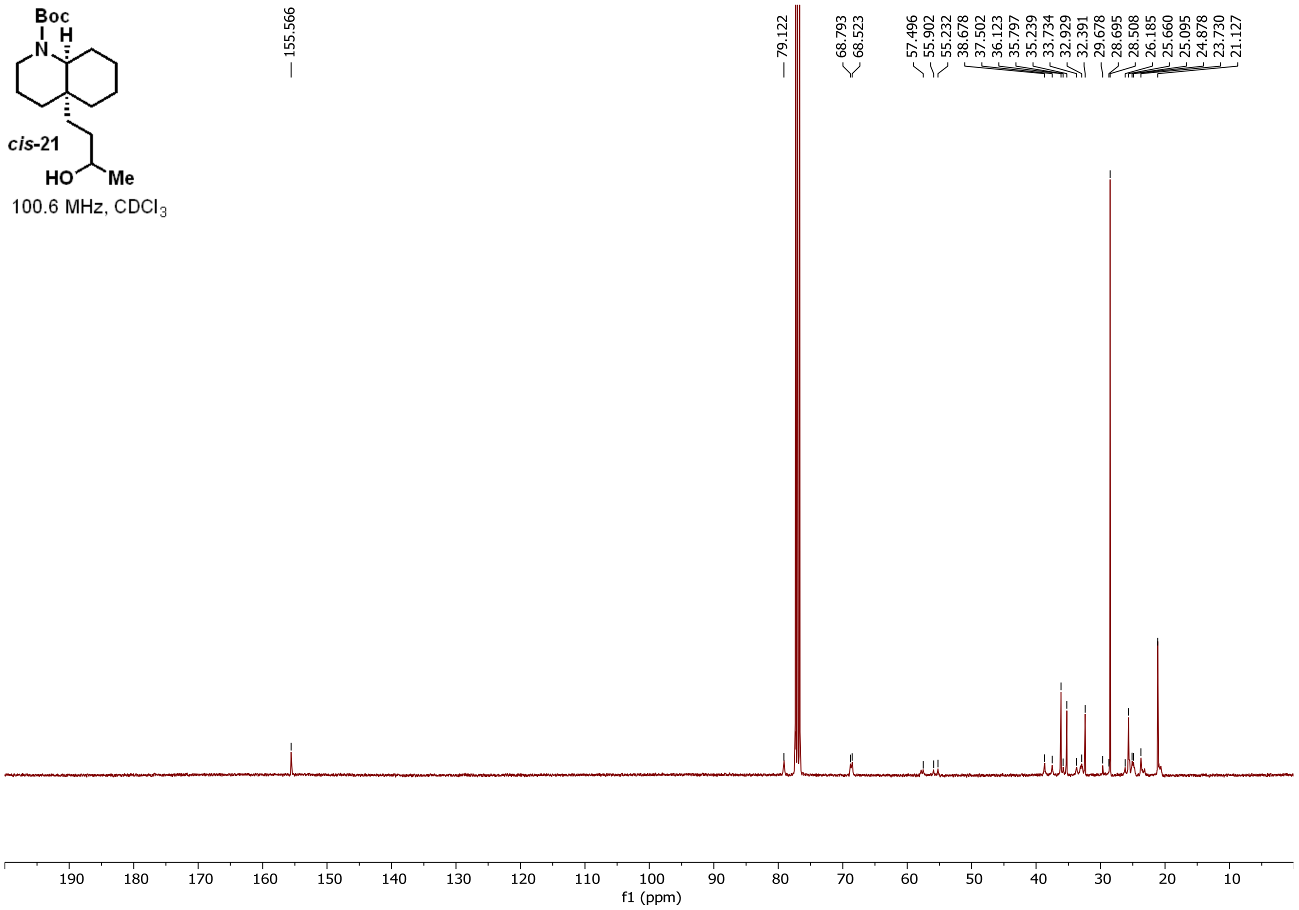
Boc
N
H
cis-21
HO
Me
100.6 MHz, CDCl3
155.566
79.122
68.793
68.523
57.496
55.902
55.232
38.678
37.502
36.123
35.797
35.239
33.734
32.929
32.391
29.678
28.695
28.508
26.185
25.660
25.095
24.878
23.730
21.127
f1 (ppm)

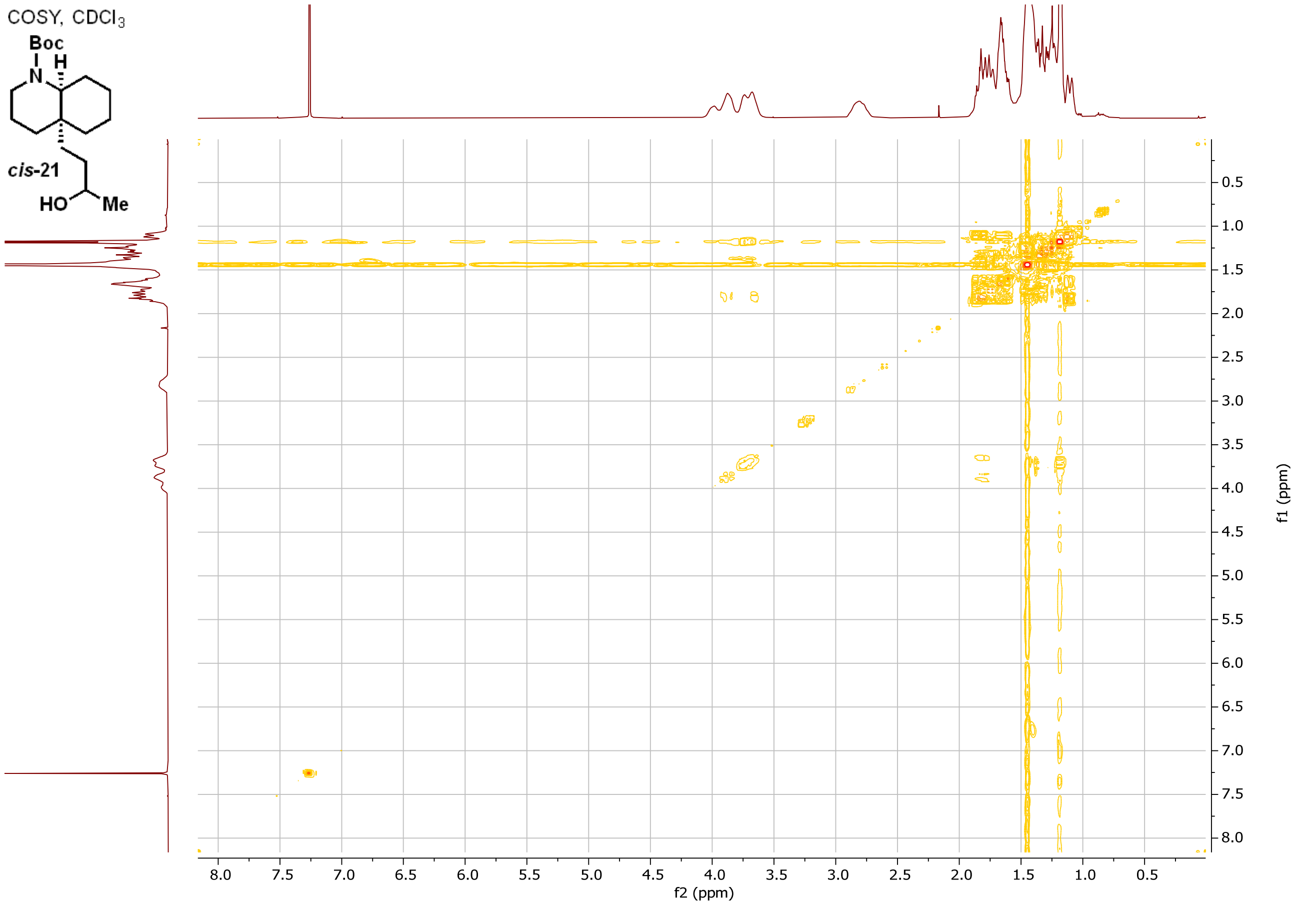
COSY, CDCl3
Boc
N
H
cis-21
HO
Me
f2 (ppm)
f1 (ppm)

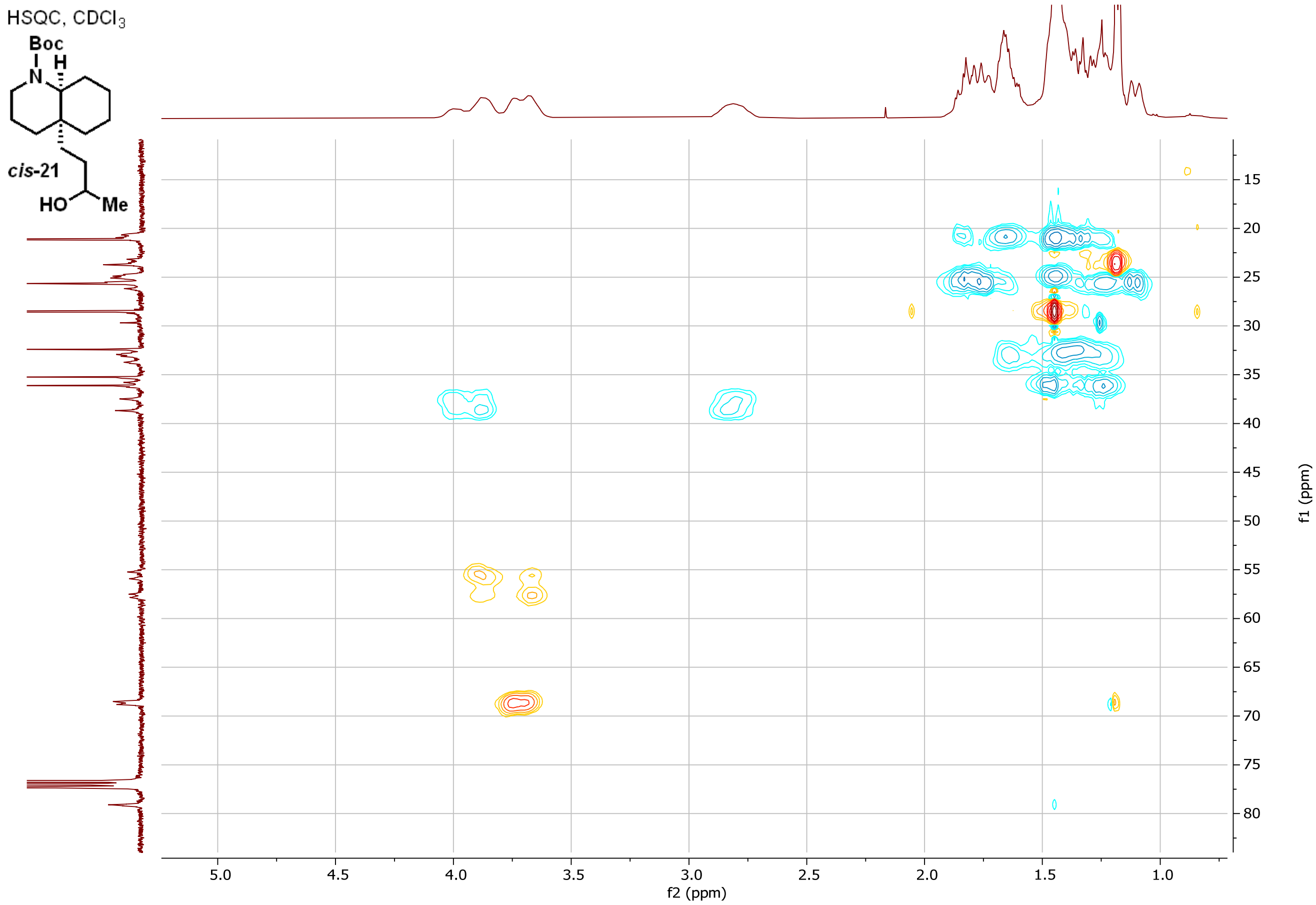
HSQC, CDCl3
Boc
N
H
cis-21
HO
Me
f2 (ppm)
f1 (ppm)

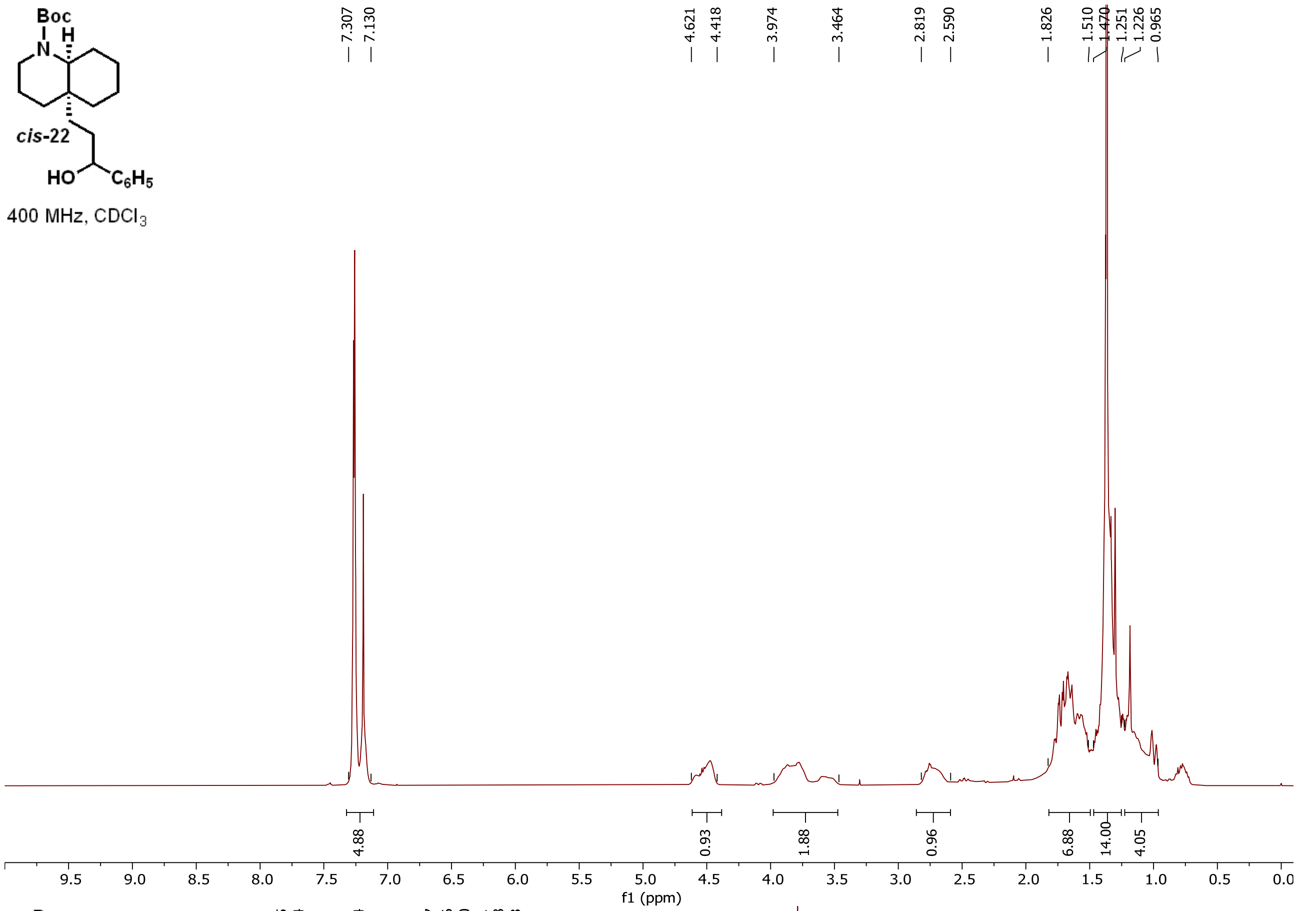
Boc
N
H
cis-22
HO
C6H5
400 MHz, CDCl3
7.307
7.130
4.621
4.418
3.974
3.464
2.819
2.590
1.826
1.510
1.470
1.251
1.226
0.965
4.88
0.93
1.88
0.96
6.88
14.00
4.05
f1 (ppm)

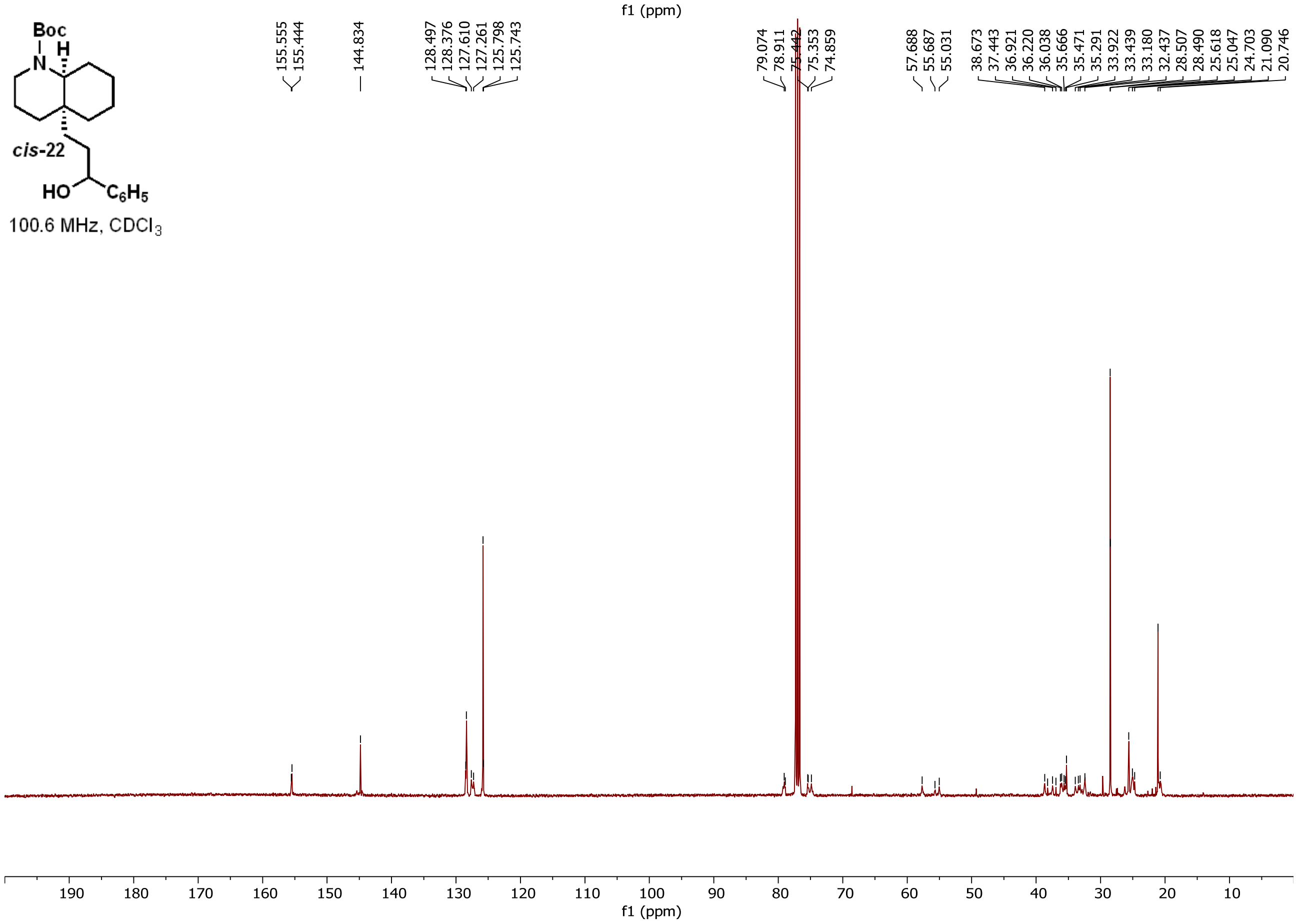
Boc
N
H
cis-22
HO
C6H5
100.6 MHz, CDCl3
155.555
155.444
144.834
128.497
128.376
127.610
127.261
125.798
125.743
79.074
78.911
75.442
75.353
74.859
57.688
55.687
55.031
38.673
37.443
36.921
36.220
36.038
35.666
35.471
35.291
33.922
33.439
33.180
32.437
28.507
28.490
25.618
25.047
24.703
21.090
20.746
f1 (ppm)

COSY, $CDCl_3$

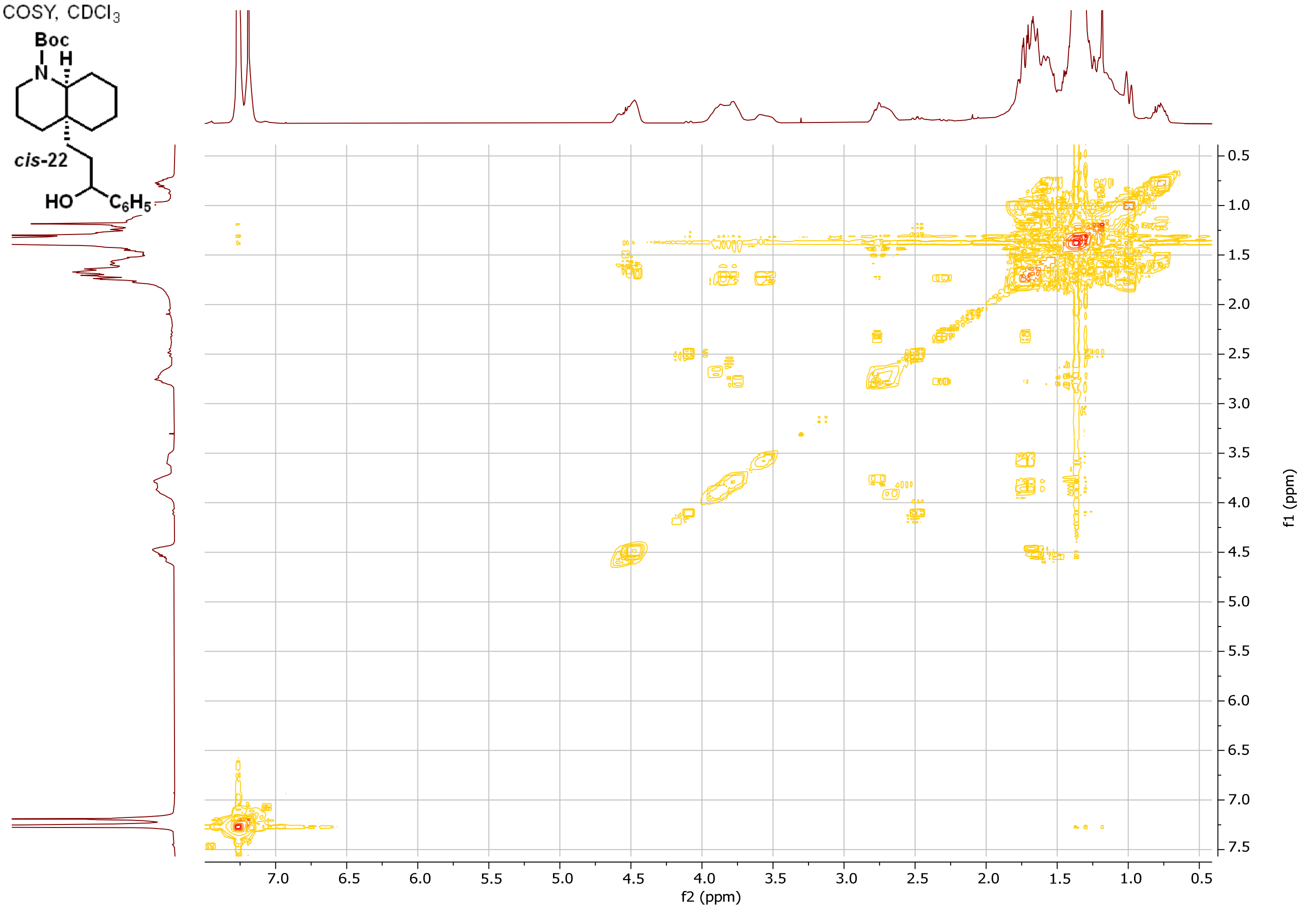

HSQC, $CDCl_3$

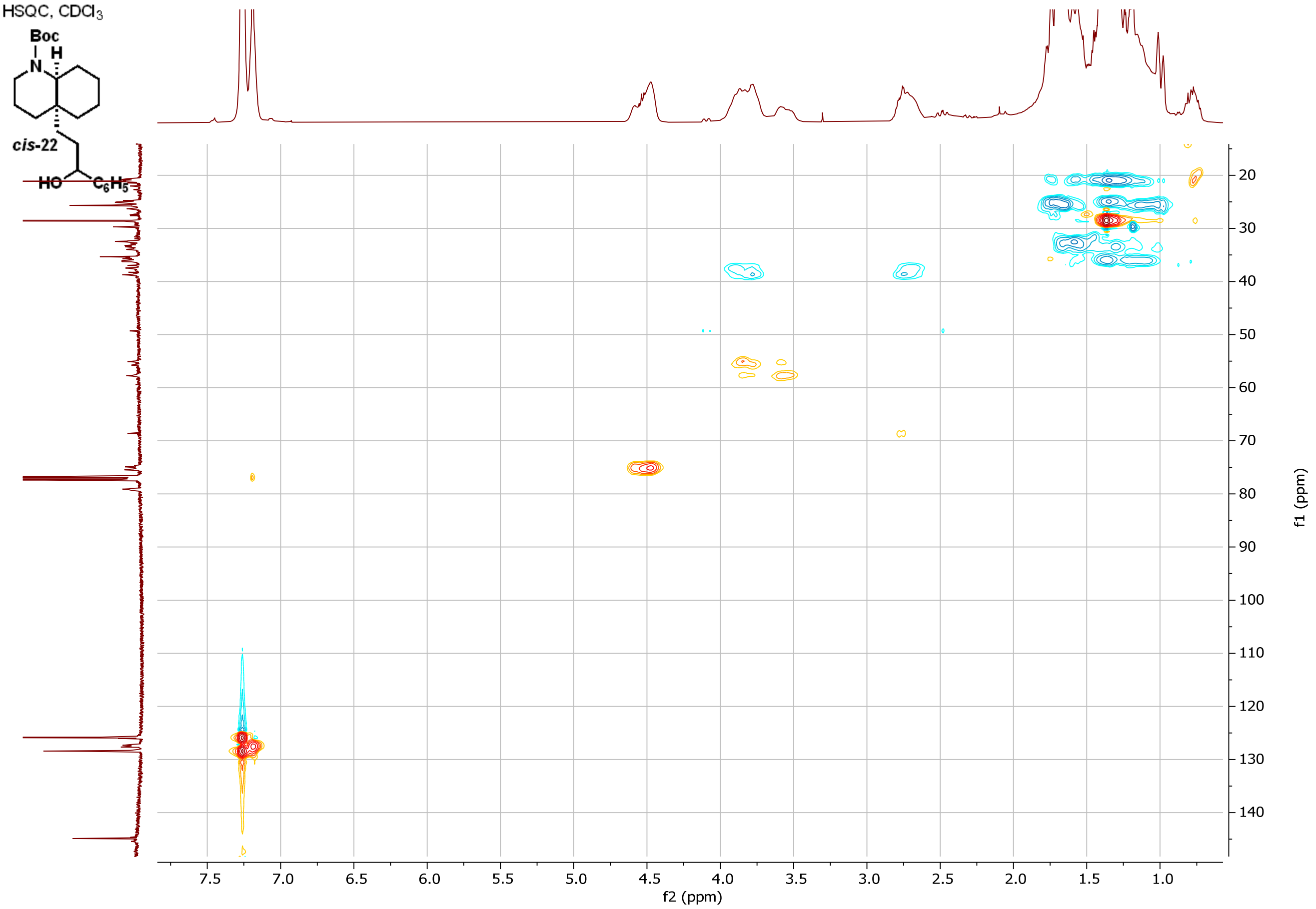

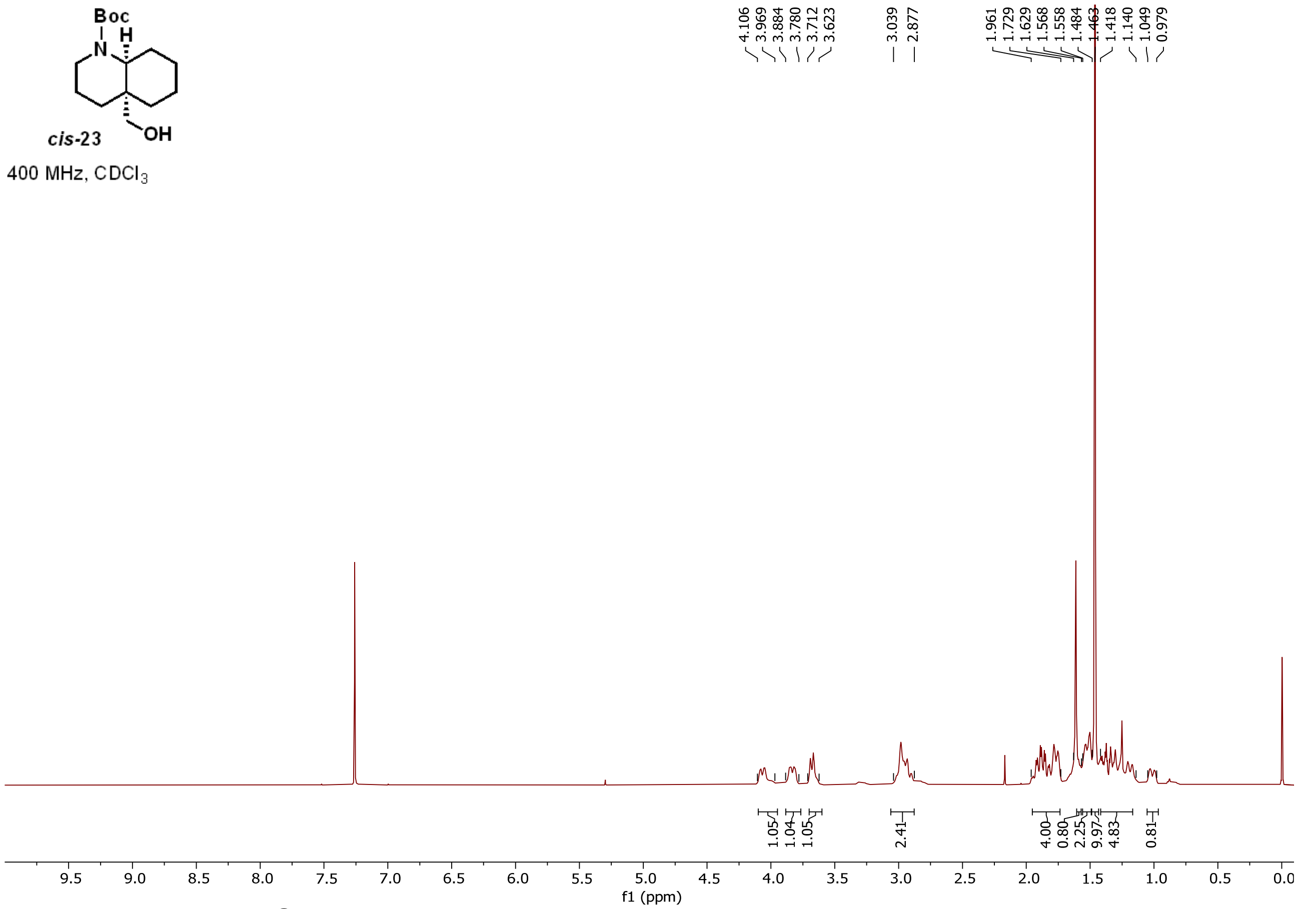
Boc
N
H
OH
cis-23
400 MHz, CDCl3
4.106
3.969
3.884
3.780
3.712
3.623
3.039
2.877
1.961
1.729
1.629
1.568
1.558
1.484
1.463
1.418
1.140
1.049
0.979
1.05
1.04
1.05
2.41
4.00
0.80
2.25
9.97
4.83
0.81
9.5 9.0 8.5 8.0 7.5 7.0 6.5 6.0 5.5 5.0 4.5 4.0 3.5 3.0 2.5 2.0 1.5 1.0 0.5 0.0
f1 (ppm)

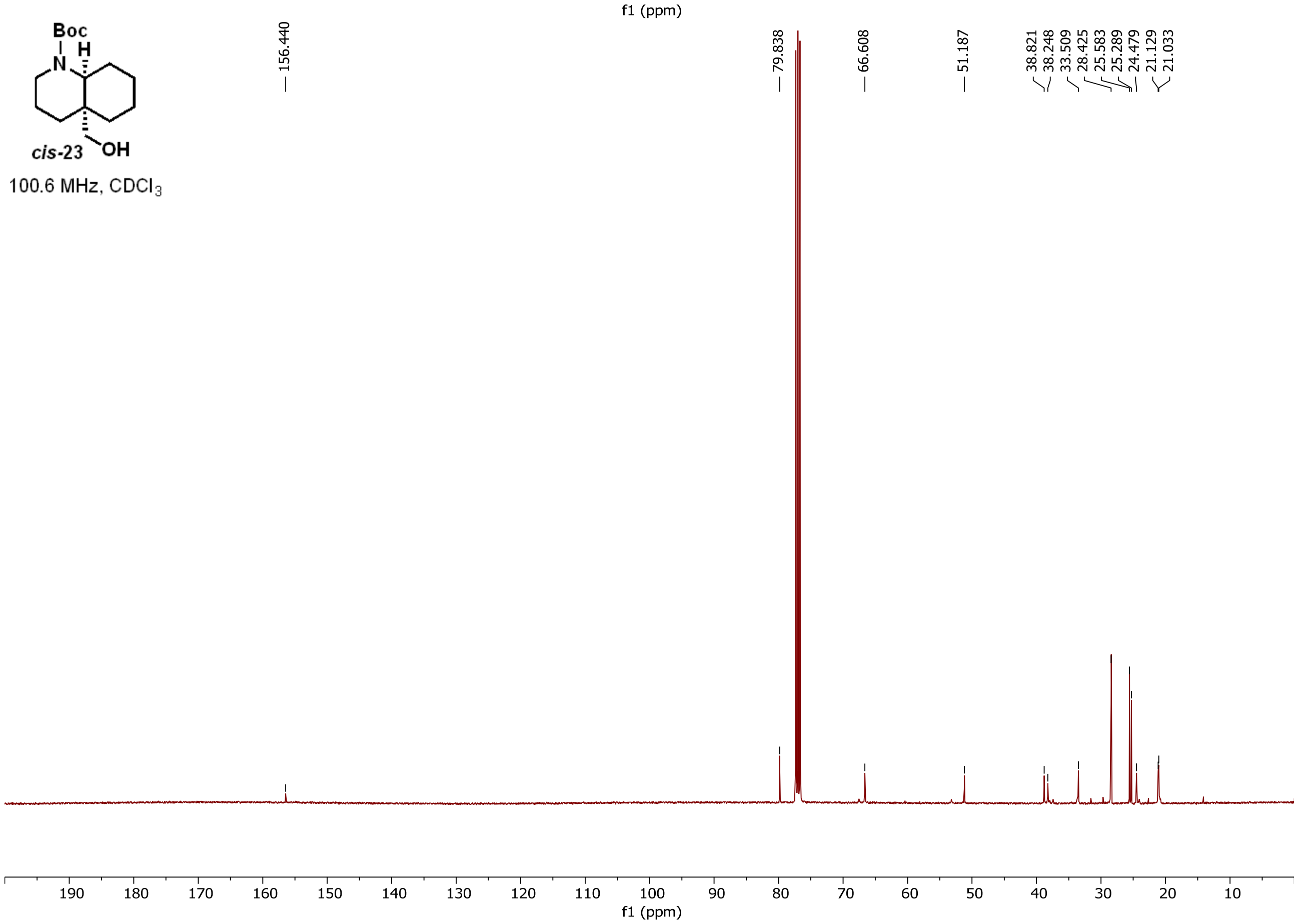
Boc
N
H
OH
cis-23
100.6 MHz, CDCl3
156.440
79.838
66.608
51.187
38.821
38.248
33.509
28.425
25.583
25.289
24.479
21.129
21.033
190 180 170 160 150 140 130 120 110 100 90 80 70 60 50 40 30 20 10
f1 (ppm)

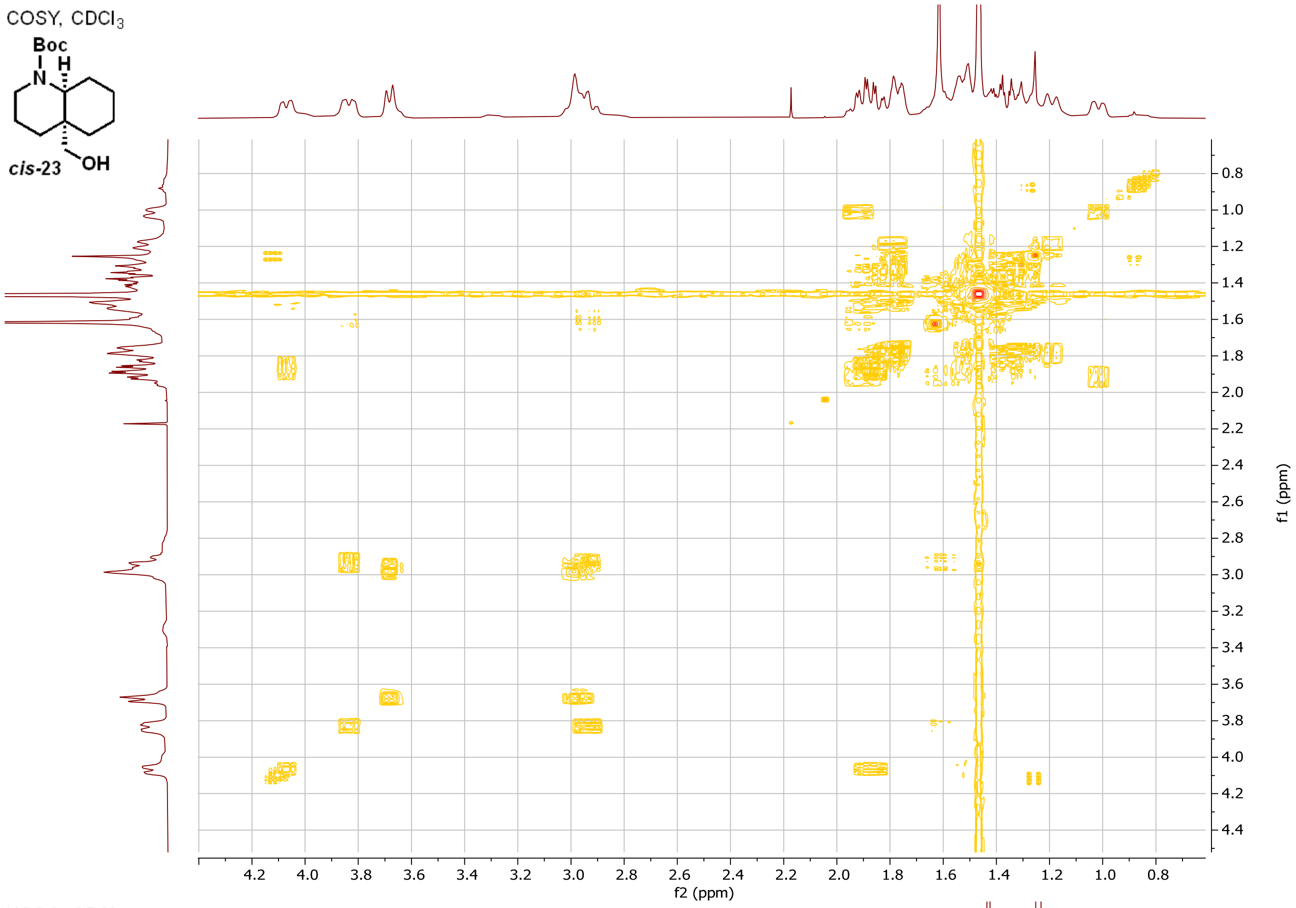
COSY, CDCl3
Boc
N
H
cis-23
OH
f2 (ppm)
f1 (ppm)

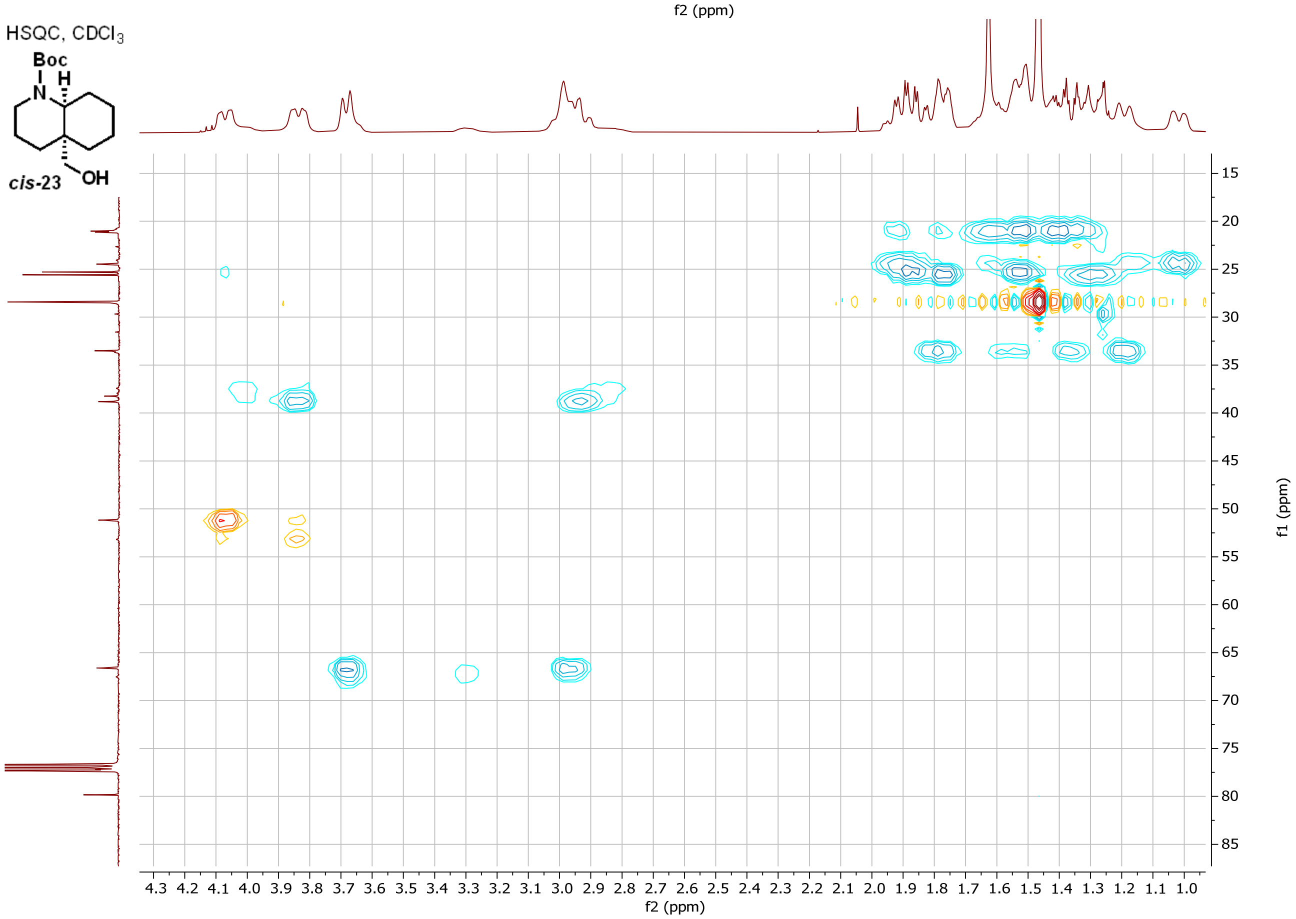
HSQC, CDCl3
Boc
N
H
cis-23
OH
f2 (ppm)
f1 (ppm)

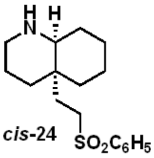

cis-24

400 MHz, $CDCl_3$

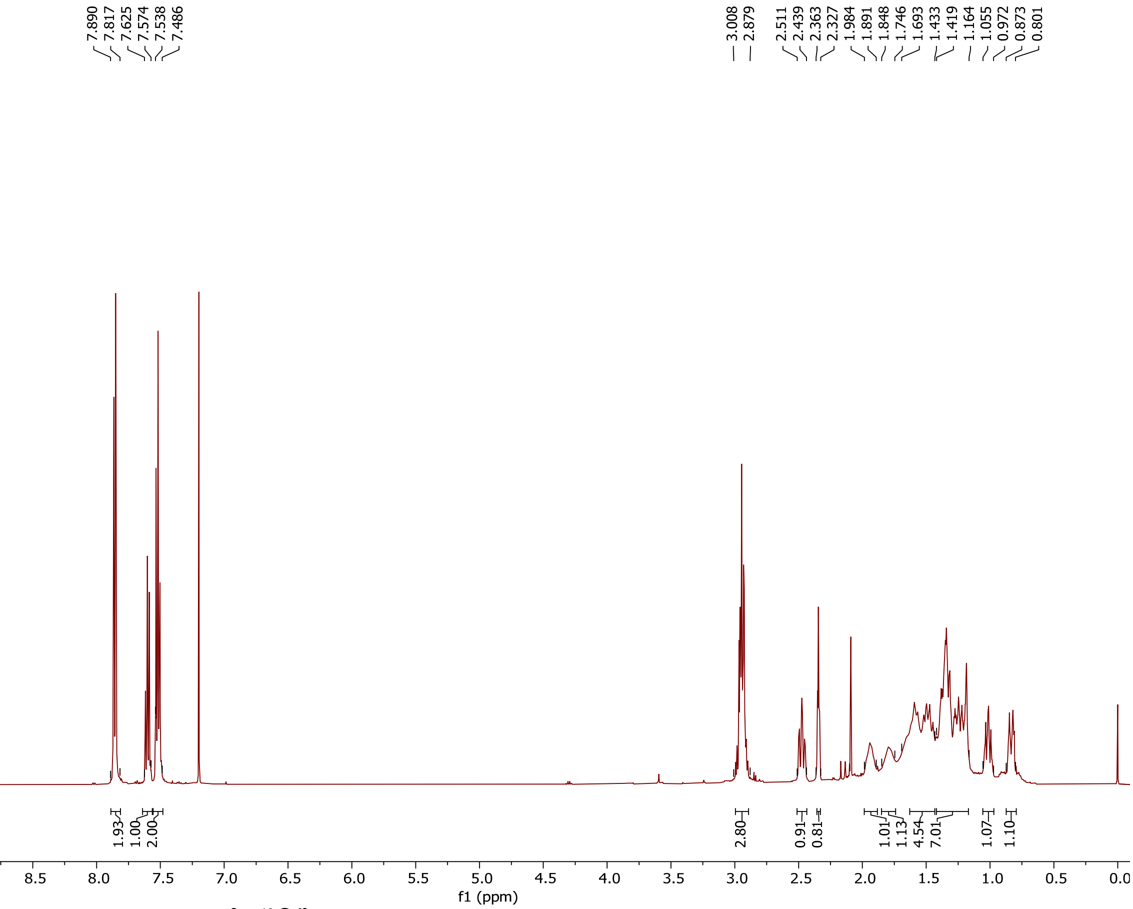

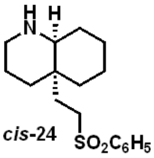

cis-24

100.6 MHz, $CDCl_3$

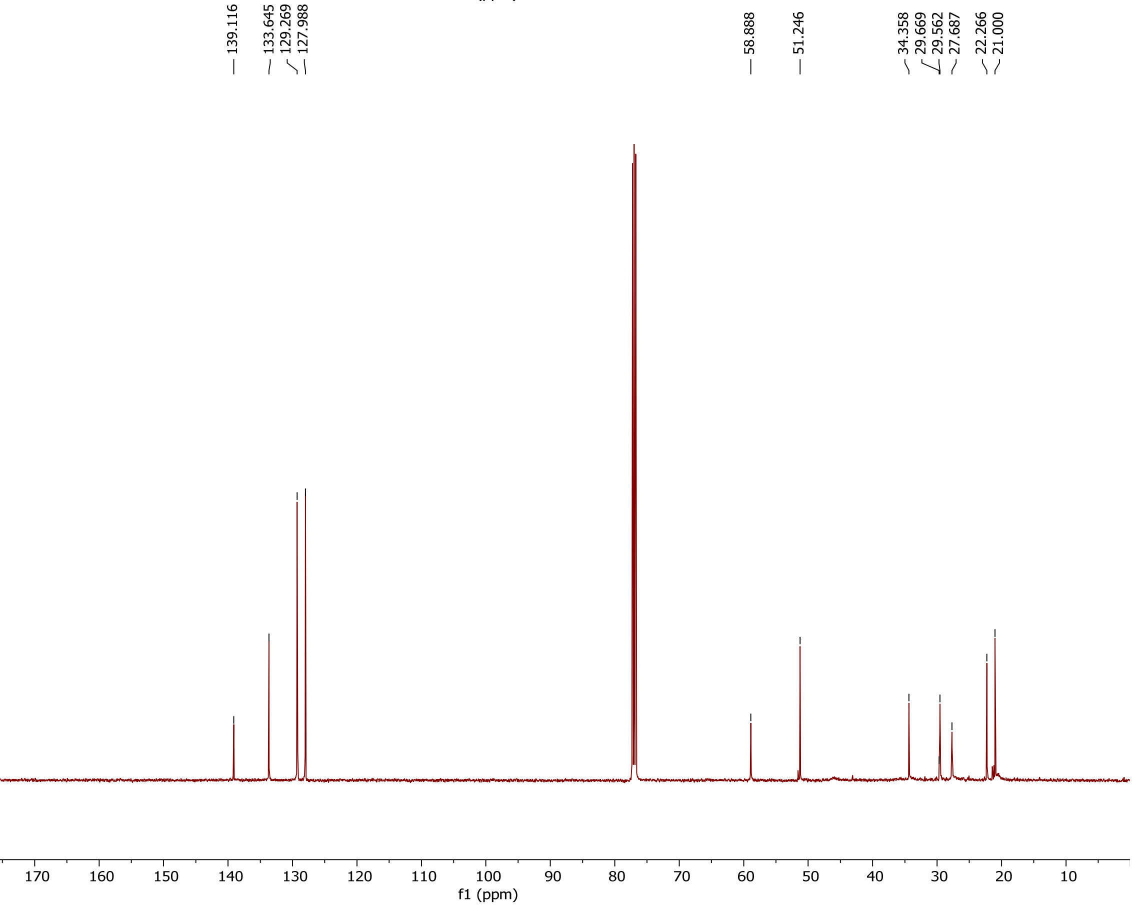

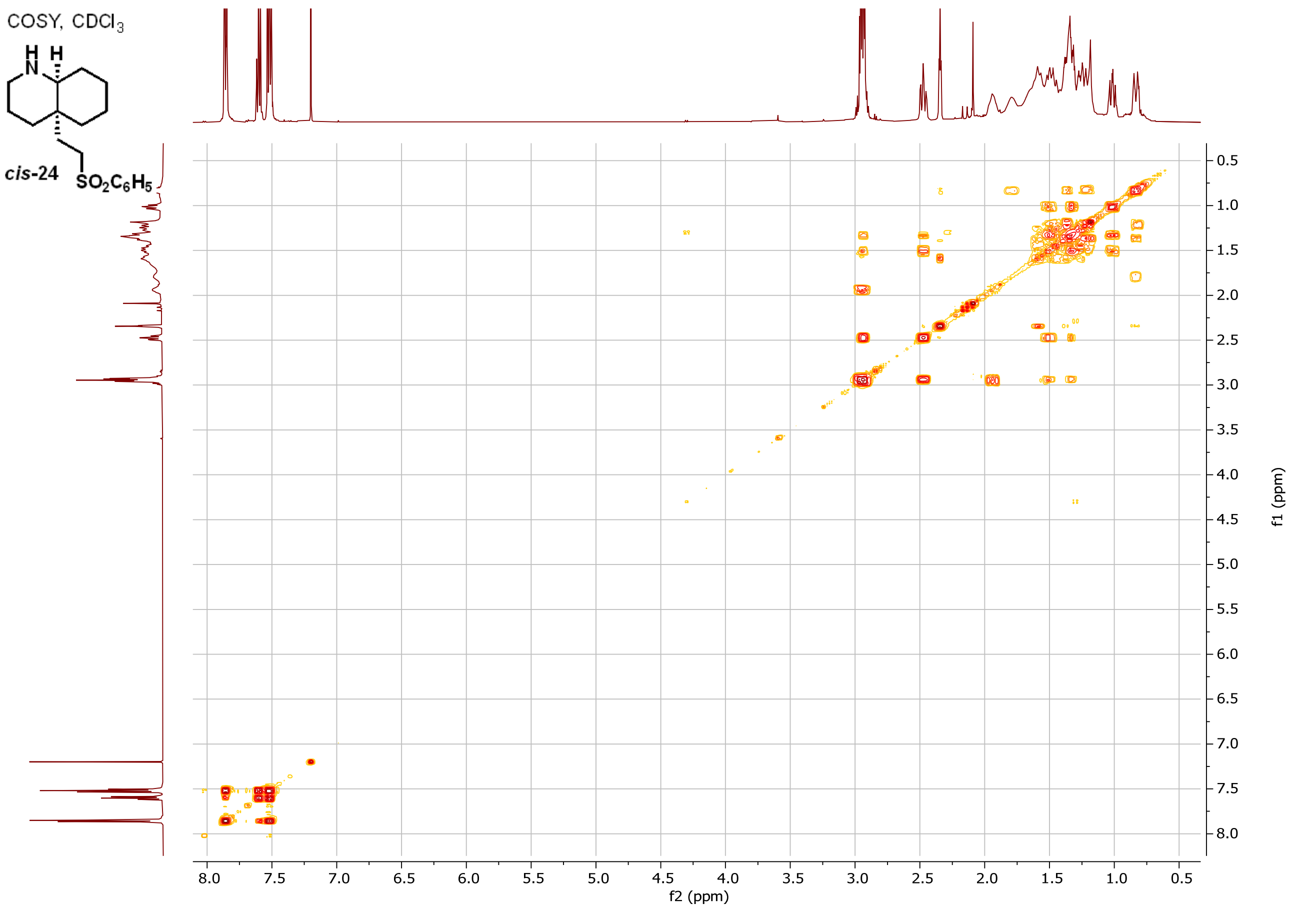
COSY, CDCl3
cis-24
SO2C6H5
f2 (ppm)
f1 (ppm)

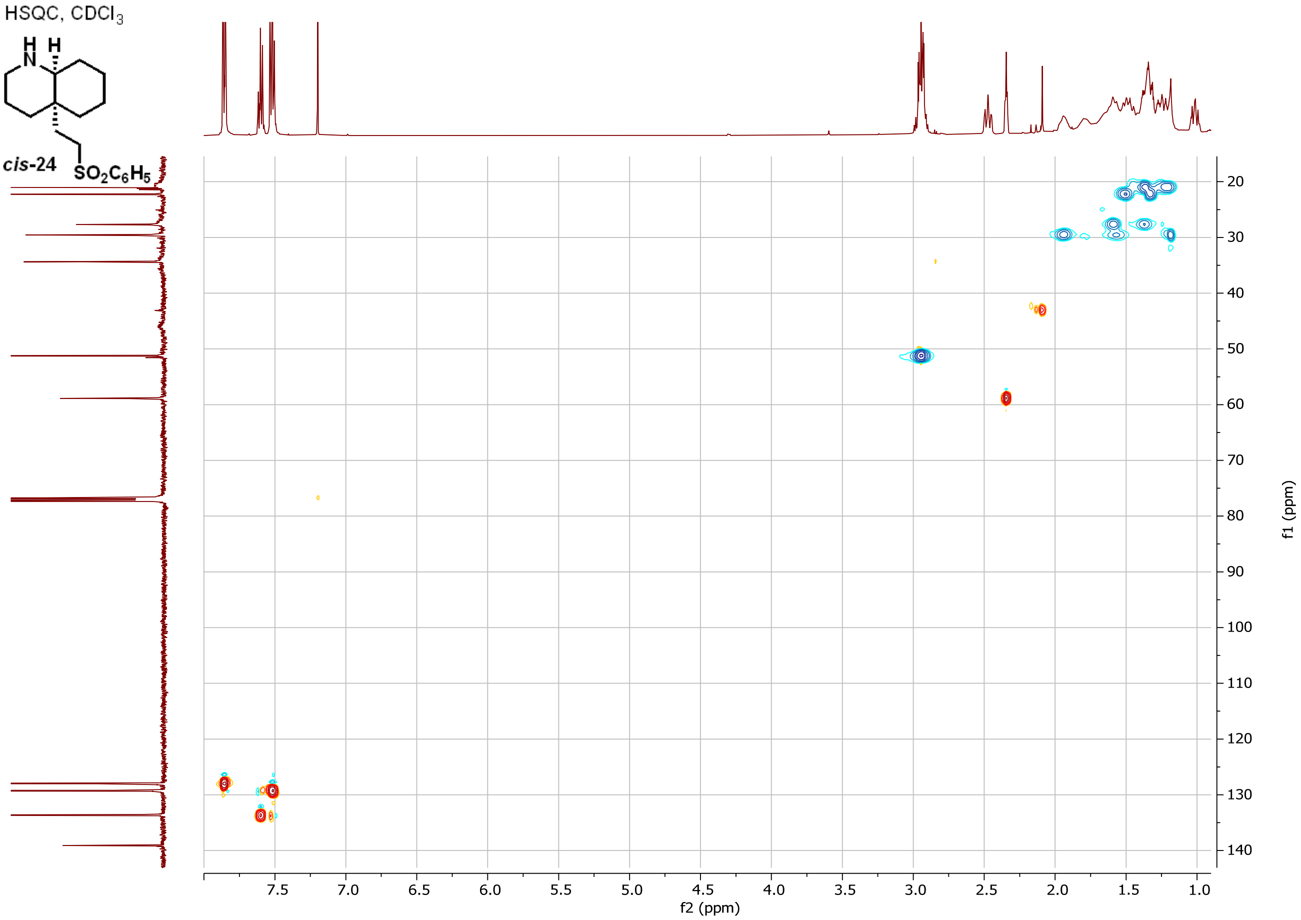
HSQC, CDCl3
cis-24
SO2C6H5
f2 (ppm)
f1 (ppm)

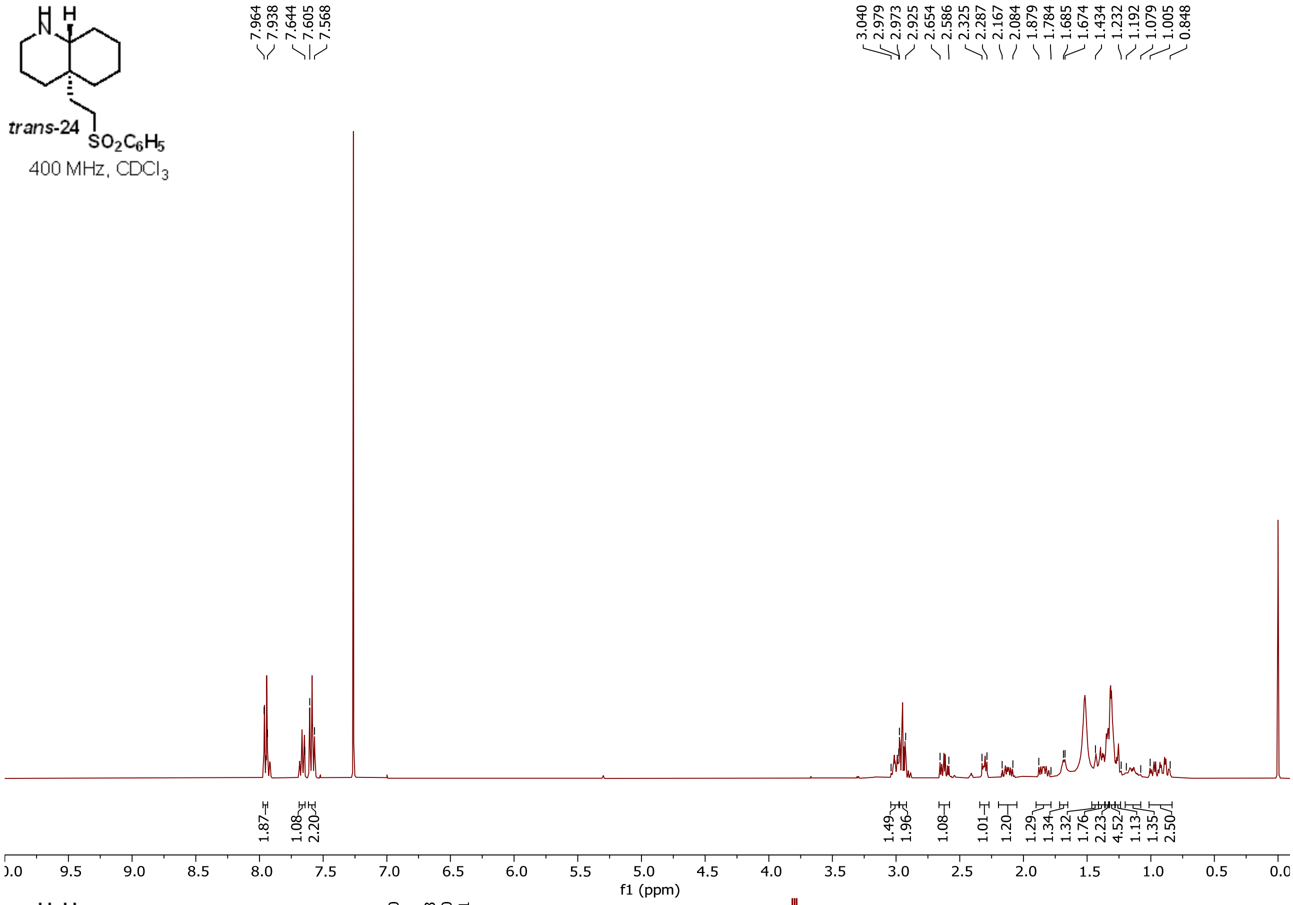
trans-24
SO2C6H5
400 MHz, CDCl3
7.964
7.938
7.644
7.605
7.568
3.040
2.979
2.973
2.925
2.654
2.586
2.325
2.287
2.167
2.084
1.879
1.784
1.685
1.674
1.434
1.232
1.192
1.079
1.005
0.848
1.87
1.08
2.20
1.49
1.96
1.08
1.01
1.20
1.29
1.34
1.32
1.76
2.23
4.52
1.13
1.35
2.50
f1 (ppm)

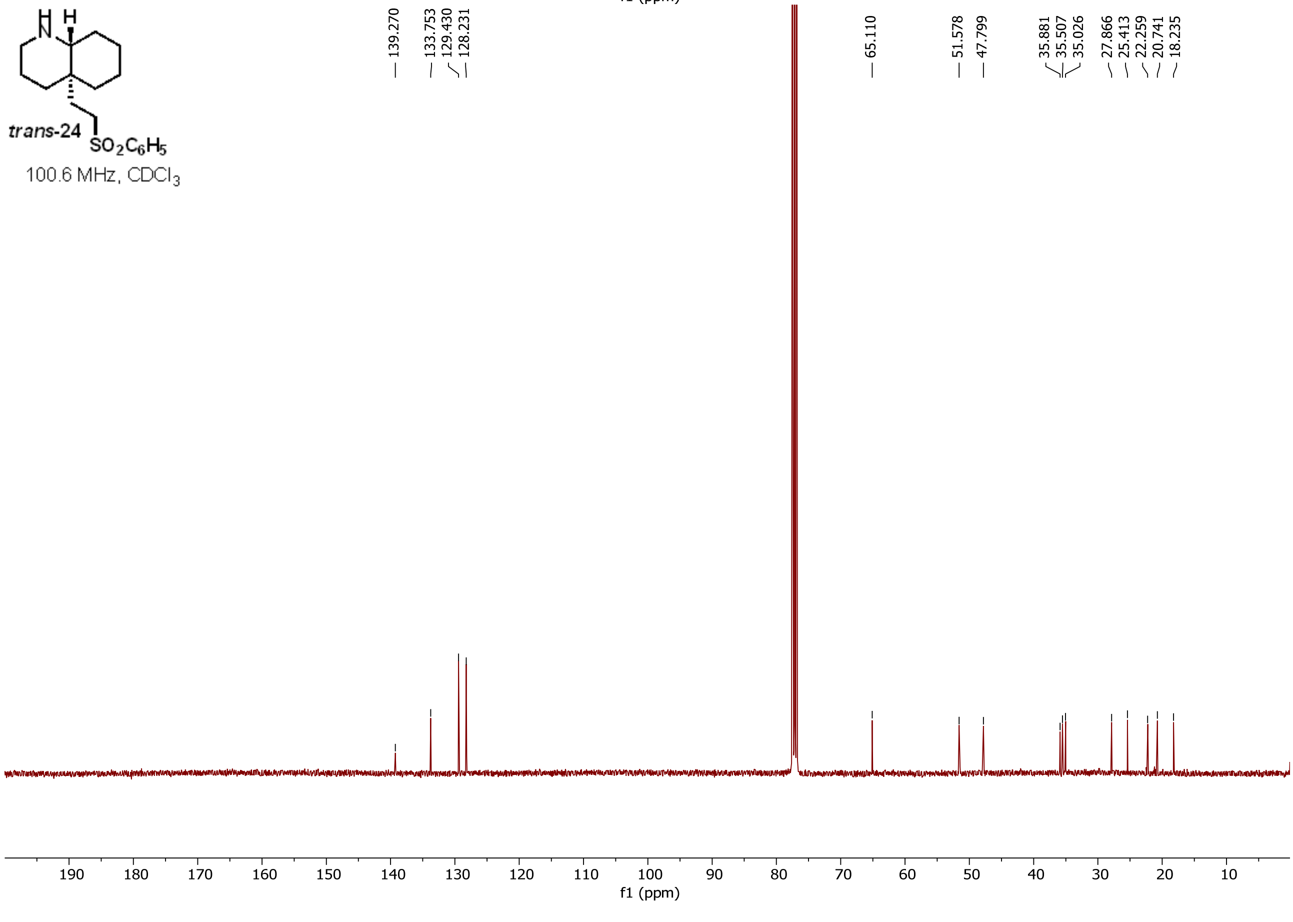
trans-24
SO2C6H5
100.6 MHz, CDCl3
139.270
133.753
129.430
128.231
65.110
51.578
47.799
35.881
35.507
35.026
27.866
25.413
22.259
20.741
18.235
f1 (ppm)

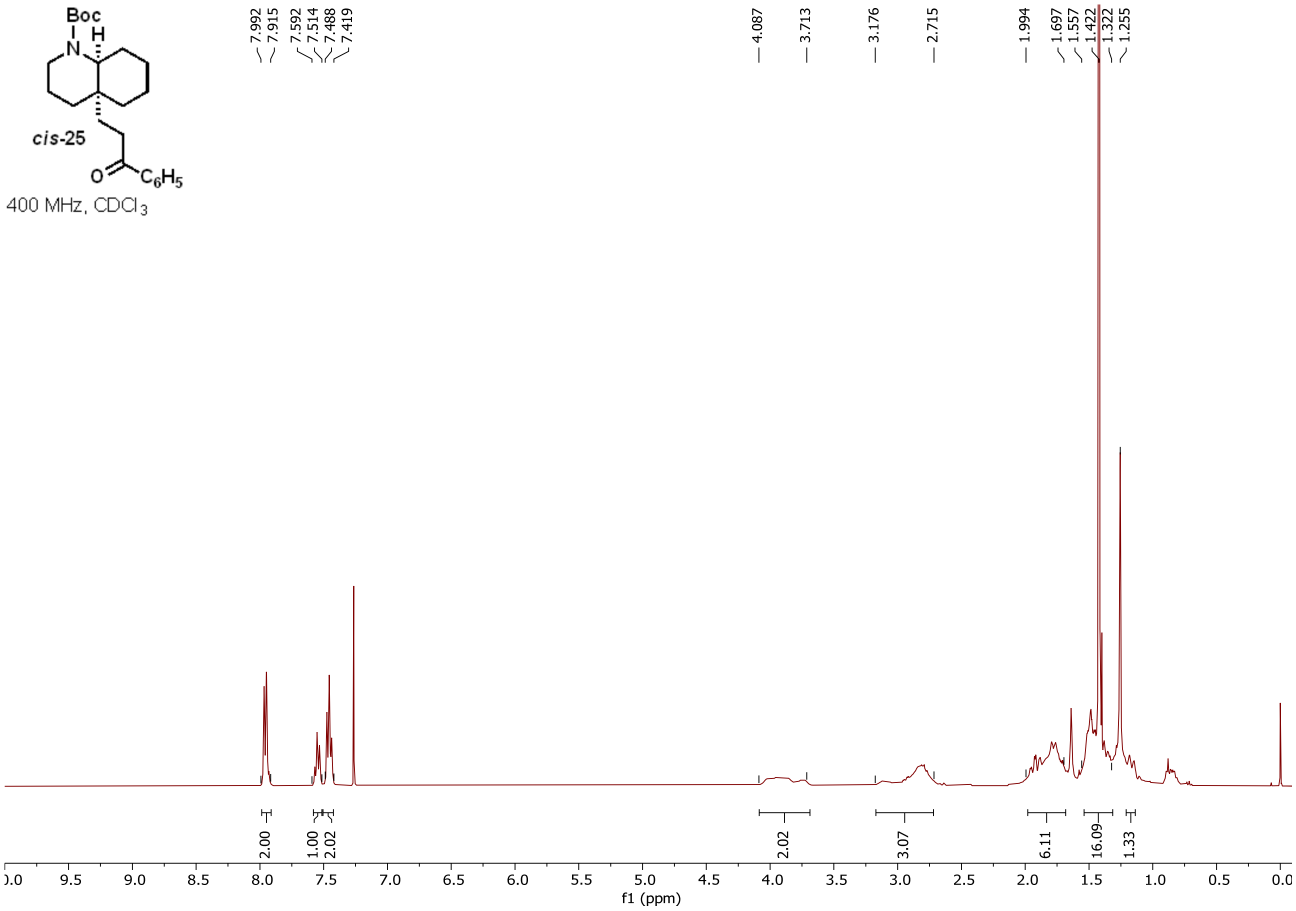
Boc
H
N
cis-25
O
C6H5
400 MHz, CDCl3
7.992
7.915
7.592
7.514
7.488
7.419
4.087
3.713
3.176
2.715
1.994
1.697
1.557
1.422
1.322
1.255
2.00
1.00
2.02
2.02
3.07
6.11
16.09
1.33
f1 (ppm)

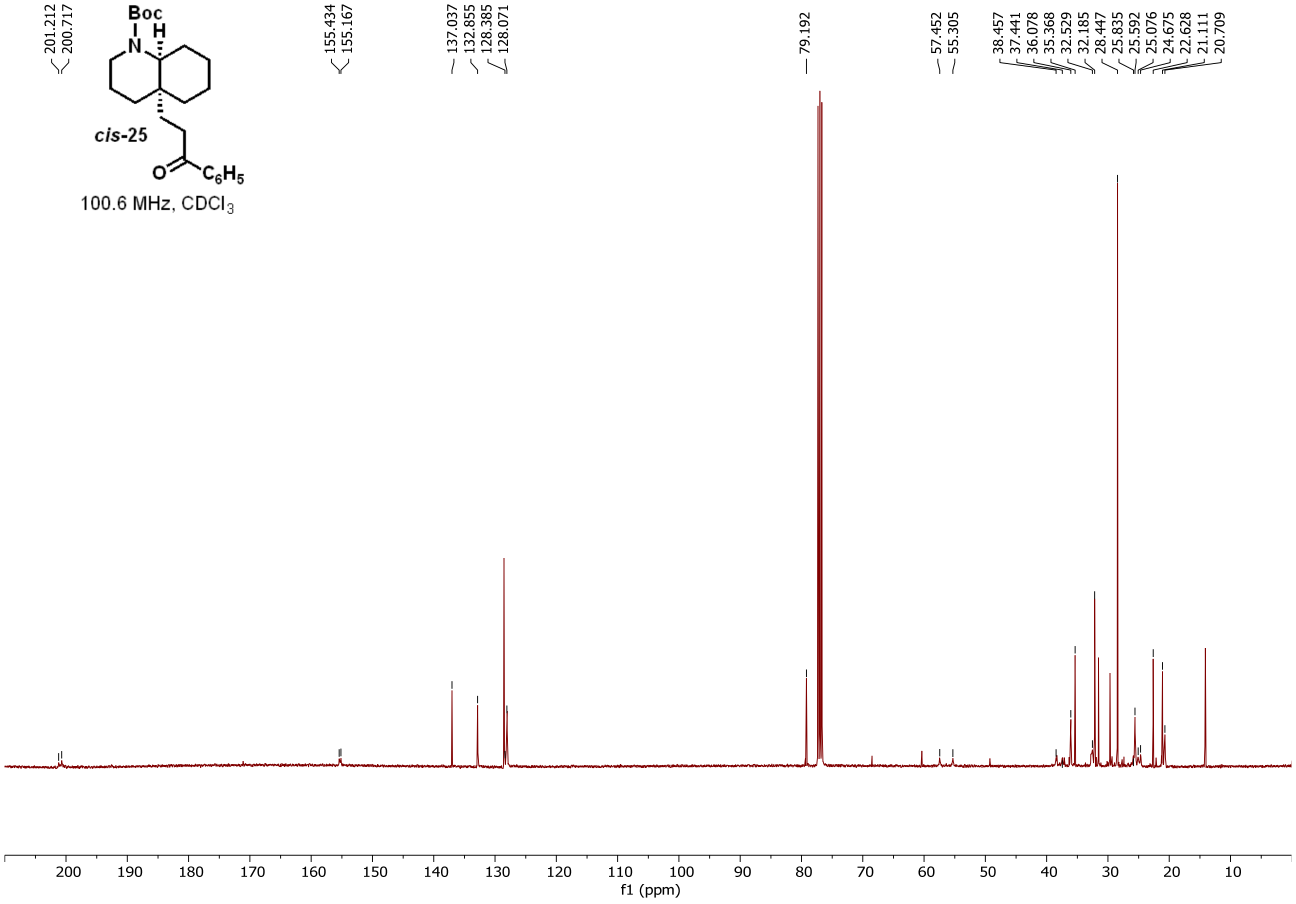
201.212
200.717
Boc
H
N
cis-25
O
C6H5
100.6 MHz, CDCl3
155.434
155.167
137.037
132.855
128.385
128.071
79.192
57.452
55.305
38.457
37.441
36.078
35.368
32.529
32.185
28.447
25.835
25.592
25.076
24.675
22.628
21.111
20.709
f1 (ppm)

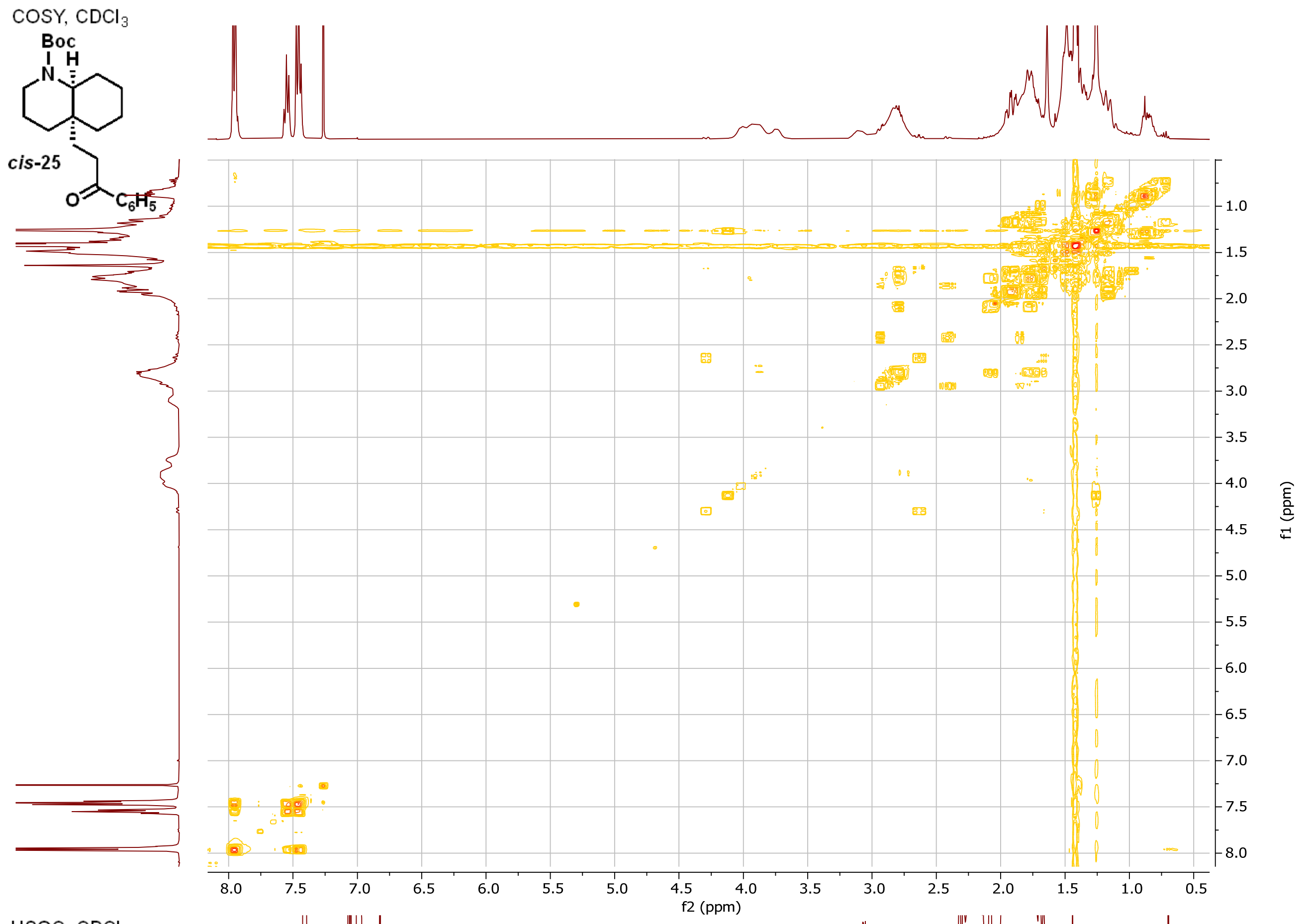
COSY, $CDCl_3$
Boc
N
H
cis-25
O
C_6H_5
f2 (ppm)
f1 (ppm)

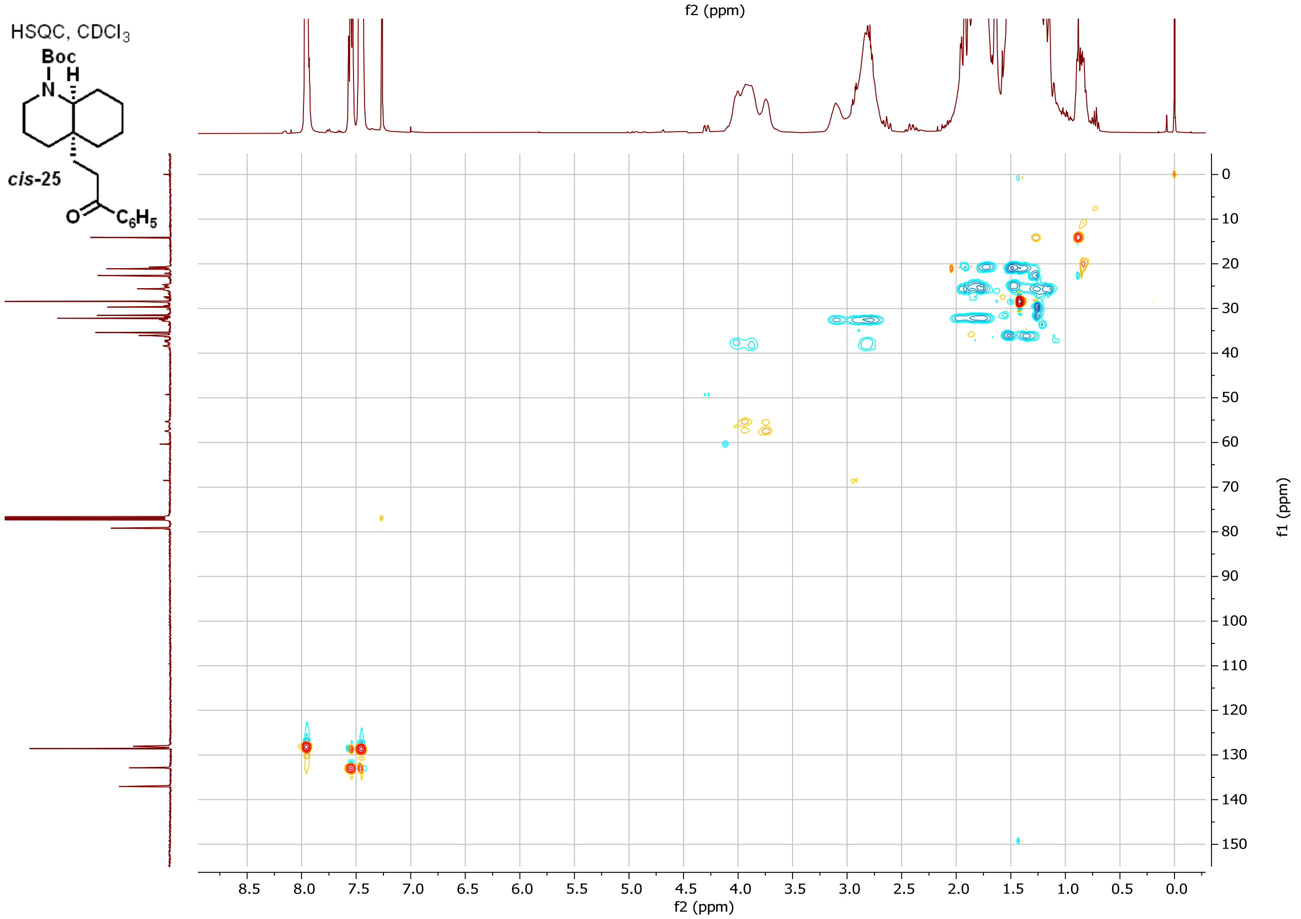
HSQC, $CDCl_3$
Boc
N
H
cis-25
O
C_6H_5
f2 (ppm)
f1 (ppm)

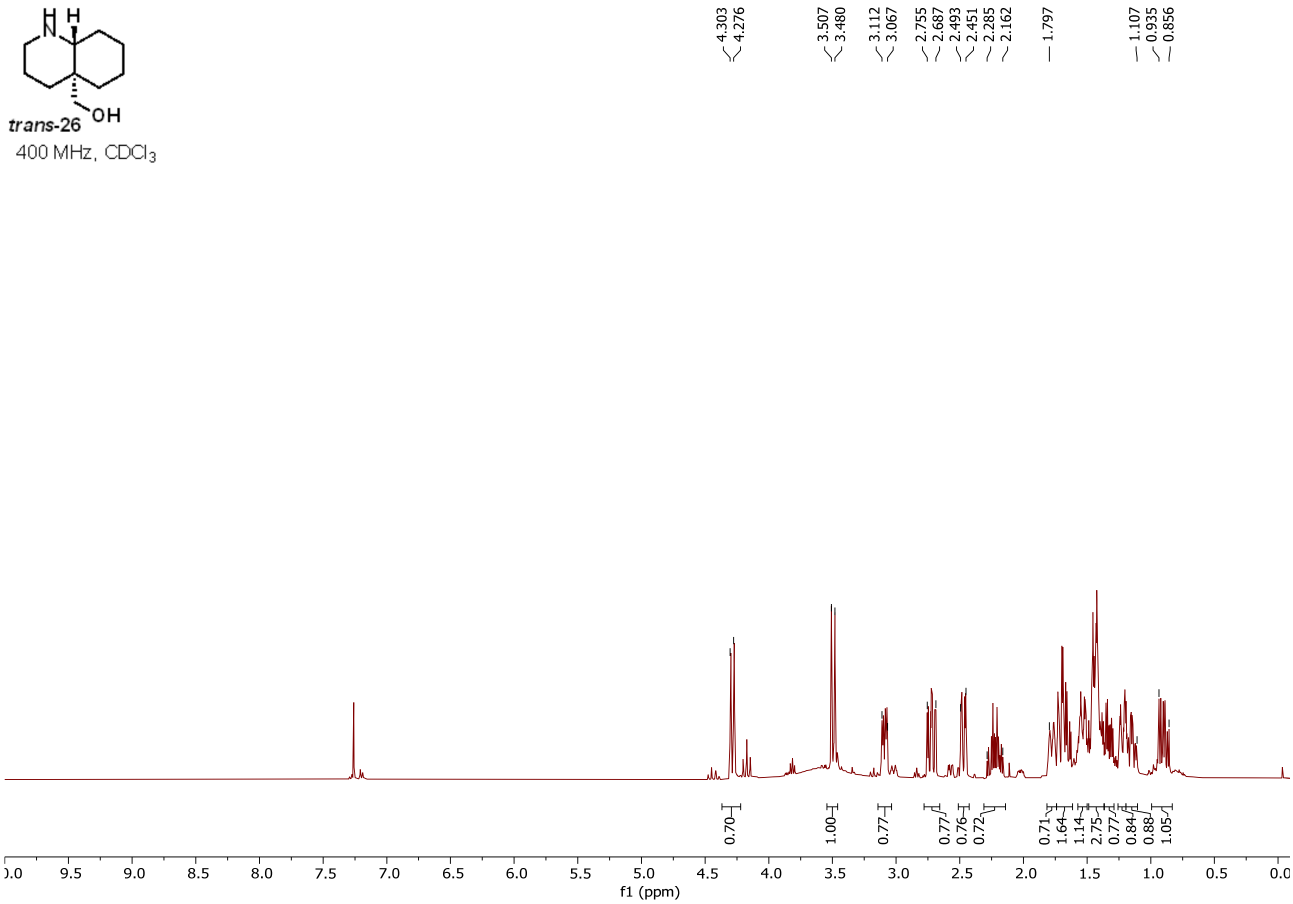
trans-26
400 MHz, CDCl3
4.303
4.276
3.507
3.480
3.112
3.067
2.755
2.687
2.493
2.451
2.285
2.162
1.797
1.107
0.935
0.856
0.70
1.00
0.77
0.77
0.76
0.72
0.71
1.64
1.14
2.75
0.77
0.84
0.88
1.05
f1 (ppm)

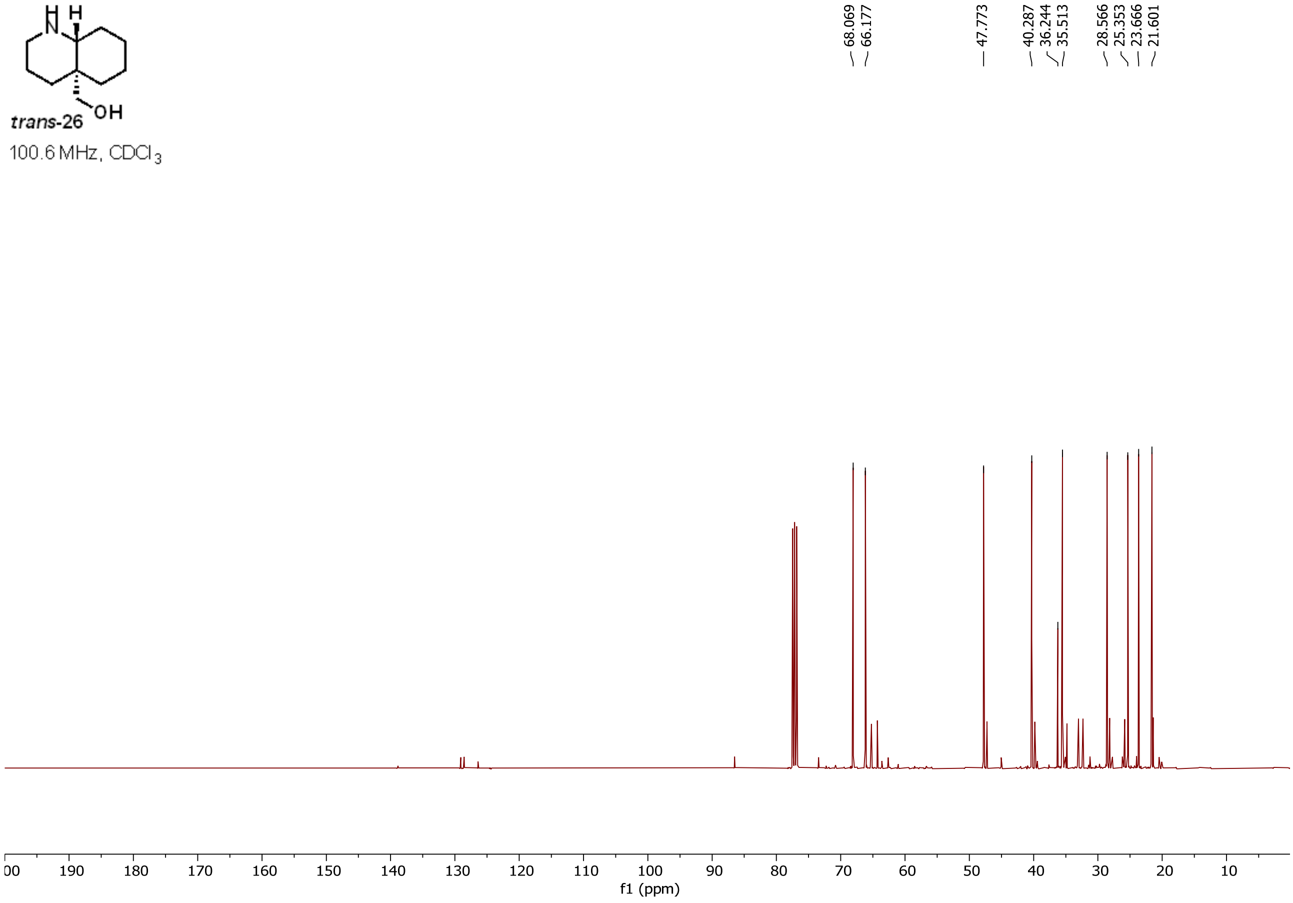
trans-26
100.6 MHz, CDCl3
68.069
66.177
47.773
40.287
36.244
35.513
28.566
25.353
23.666
21.601
f1 (ppm)

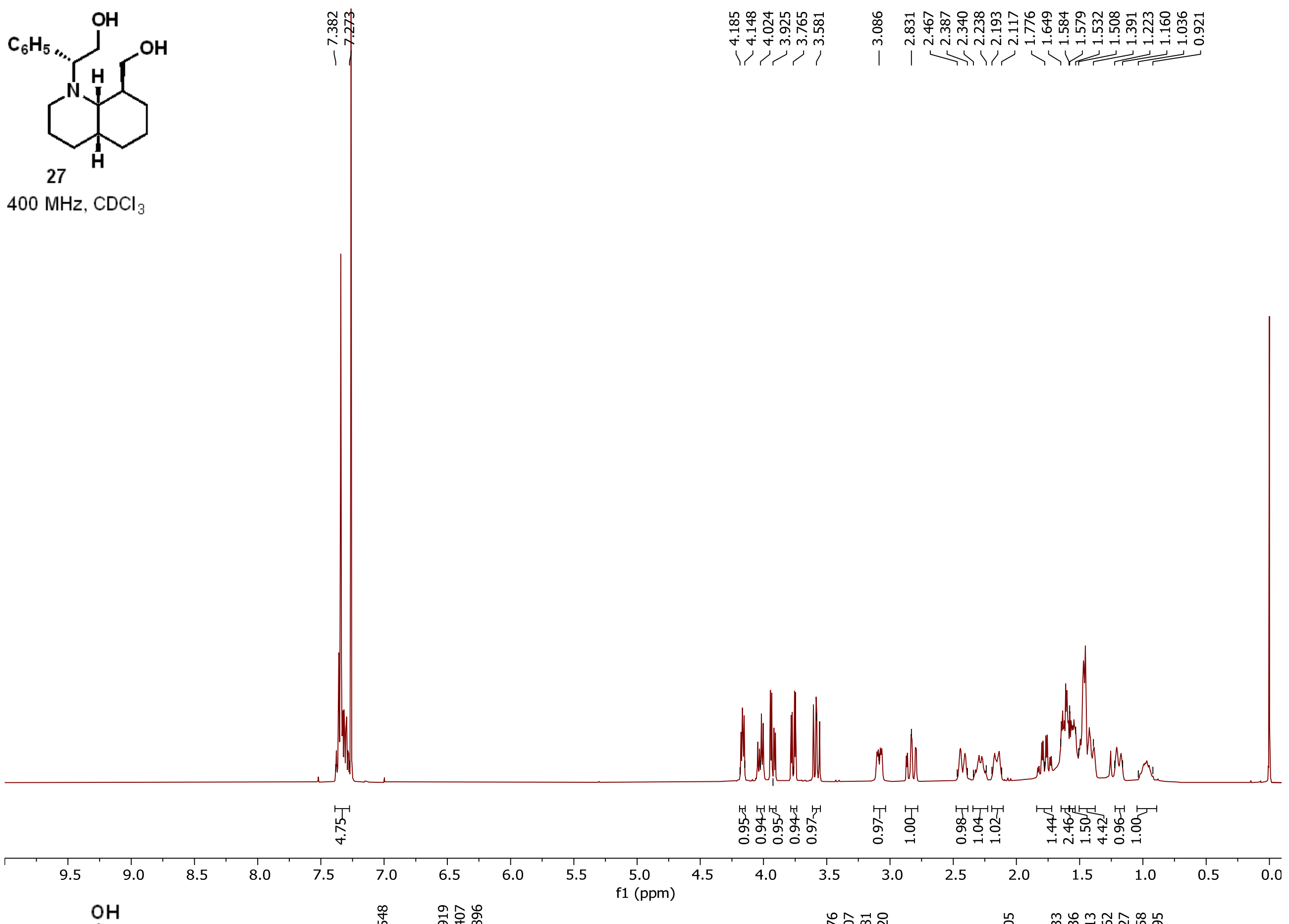

OH
C6H5
OH
N
H
H
27
400 MHz, CDCl3
7.382
7.273
4.185
4.148
4.024
3.925
3.765
3.581
3.086
2.831
2.467
2.387
2.340
2.238
2.193
2.117
1.776
1.649
1.584
1.579
1.532
1.508
1.391
1.223
1.160
1.036
0.921
4.75
0.95
0.94
0.95
0.94
0.97
0.97
1.00
0.98
1.04
1.02
1.44
2.46
1.50
4.42
0.96
1.00
f1 (ppm)

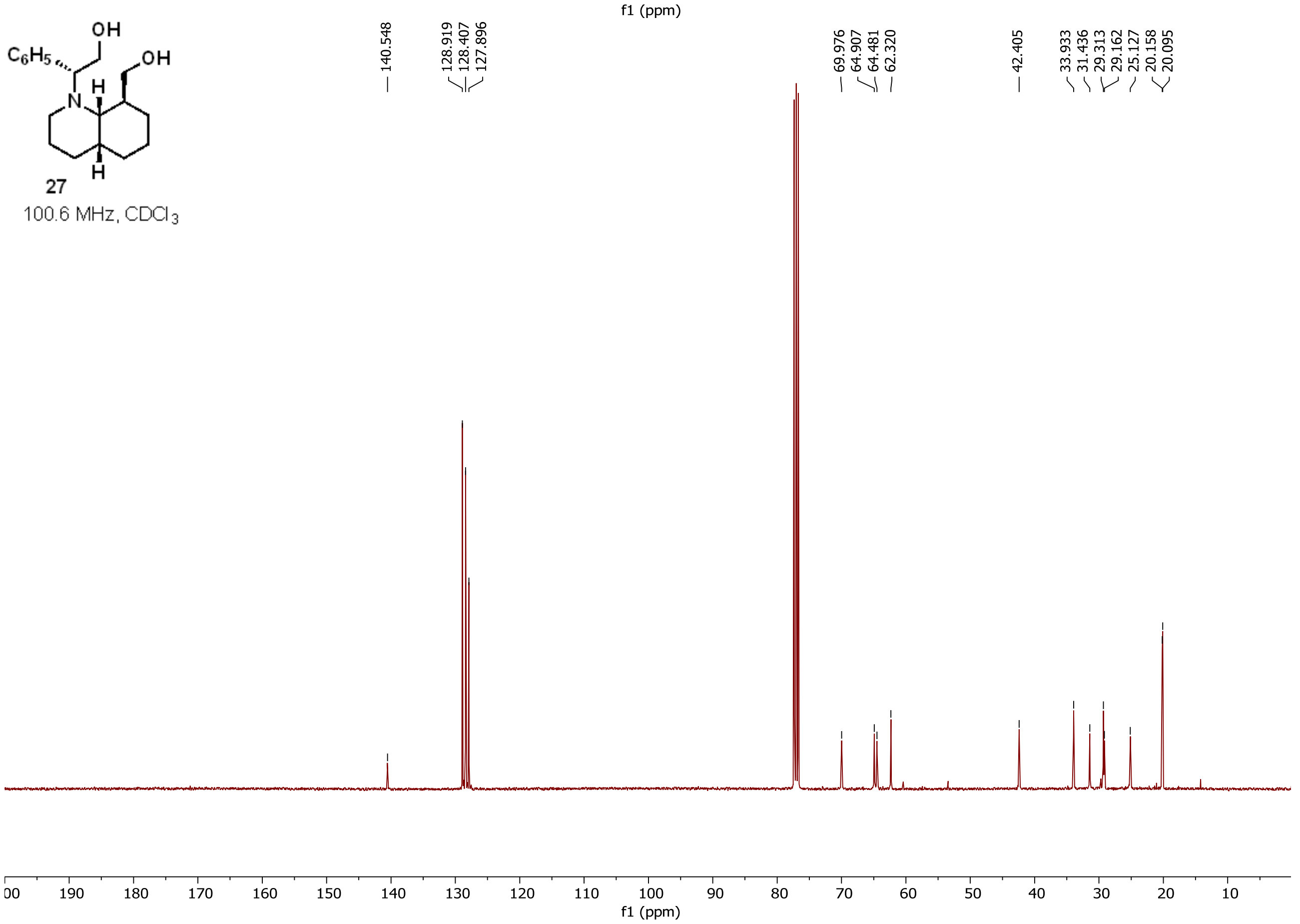

OH
C6H5
OH
N
H
H
27
100.6 MHz, CDCl3
140.548
128.919
128.407
127.896
69.976
64.907
64.481
62.320
42.405
33.933
31.436
29.313
29.162
25.127
20.158
20.095
f1 (ppm)

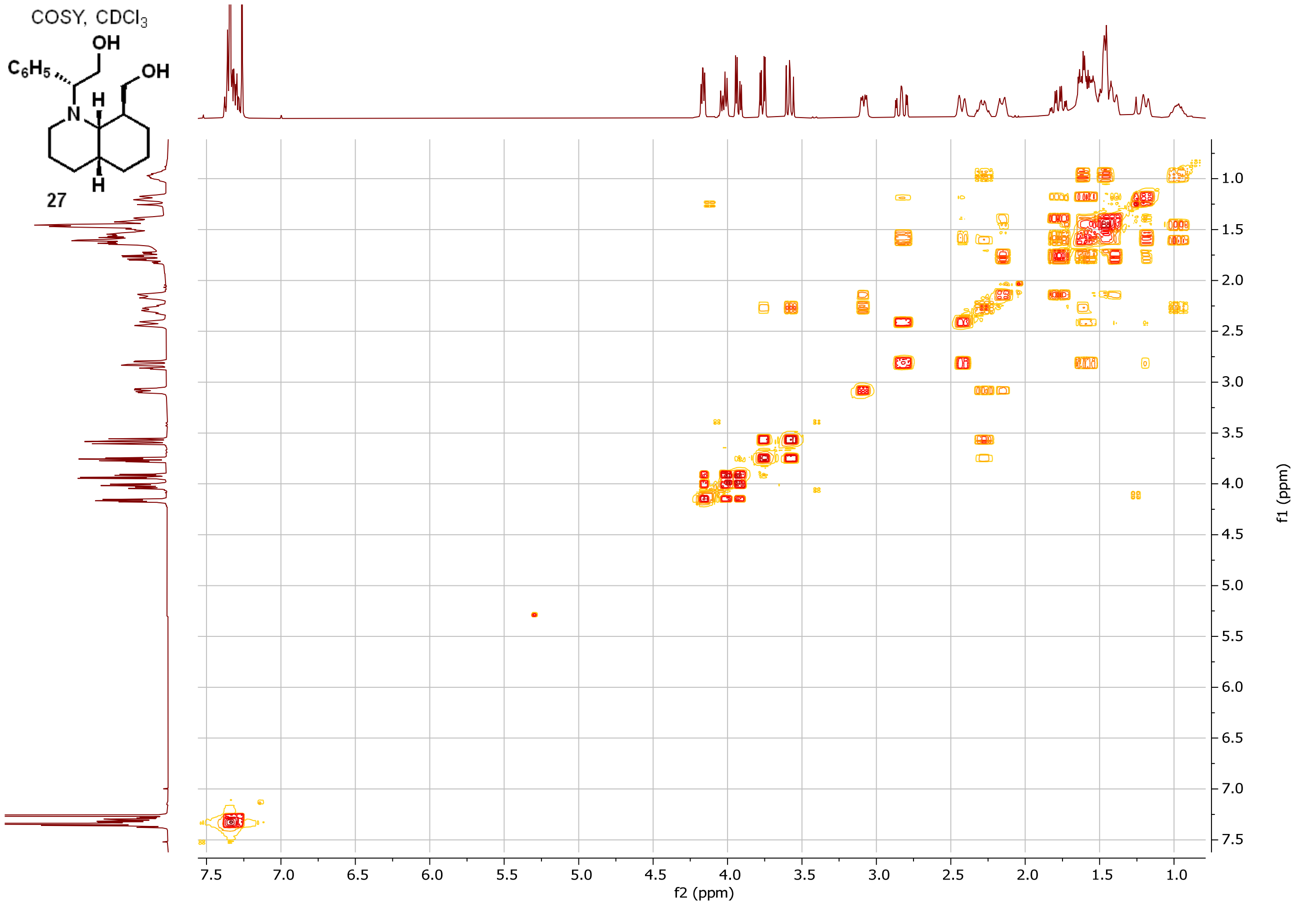
COSY, CDCl3
OH
C6H5
OH
N
H
H
27
f1 (ppm)
f2 (ppm)

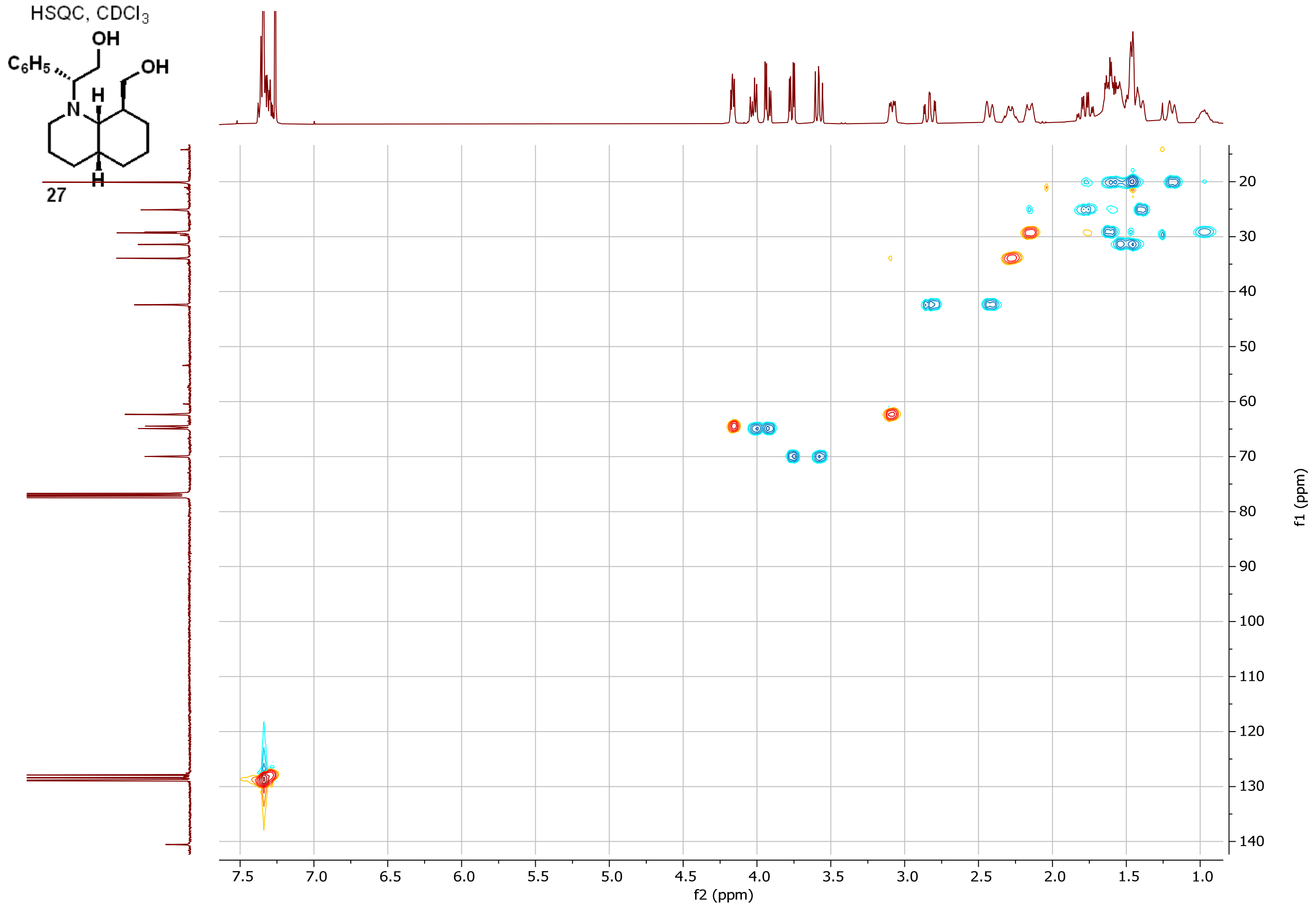
HSQC, CDCl3
OH
C6H5
OH
N
H
H
27
f1 (ppm)
f2 (ppm)

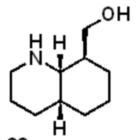

28

400 MHz, $CDCl_3$

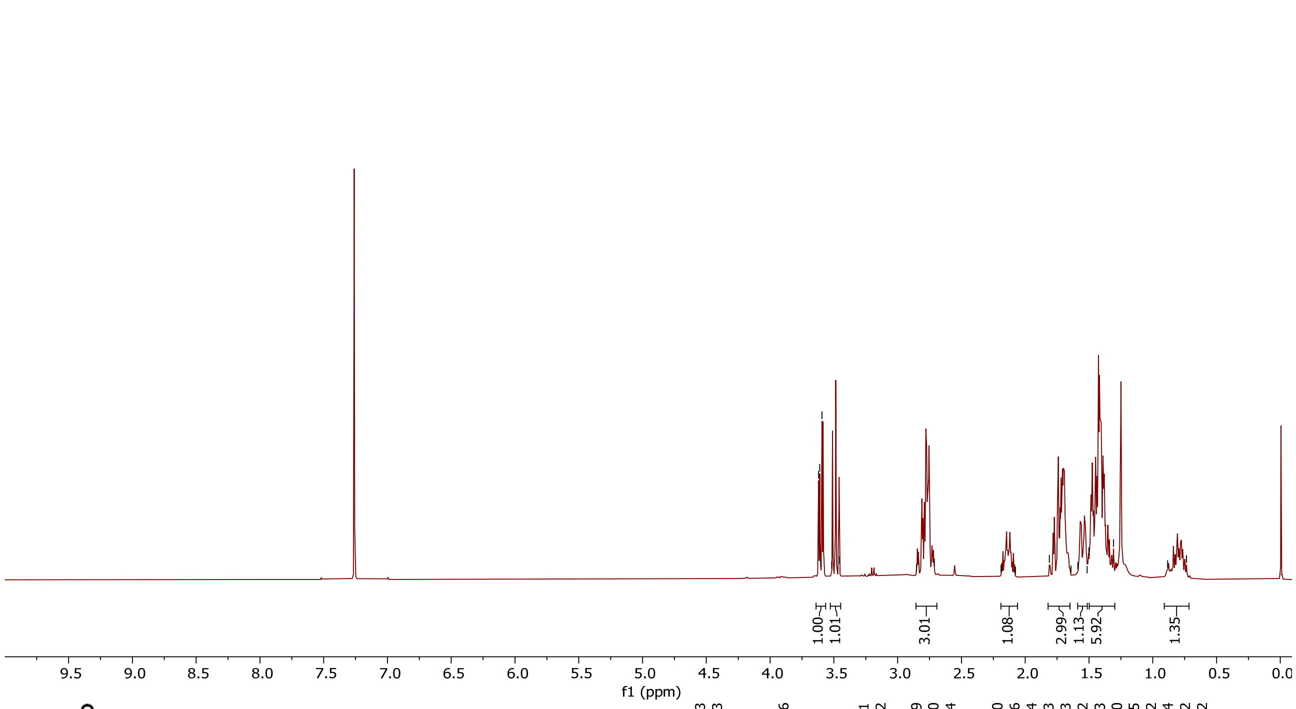

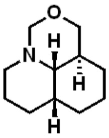

(−)-Myrioxazine A

400 MHz, $CDCl_3$

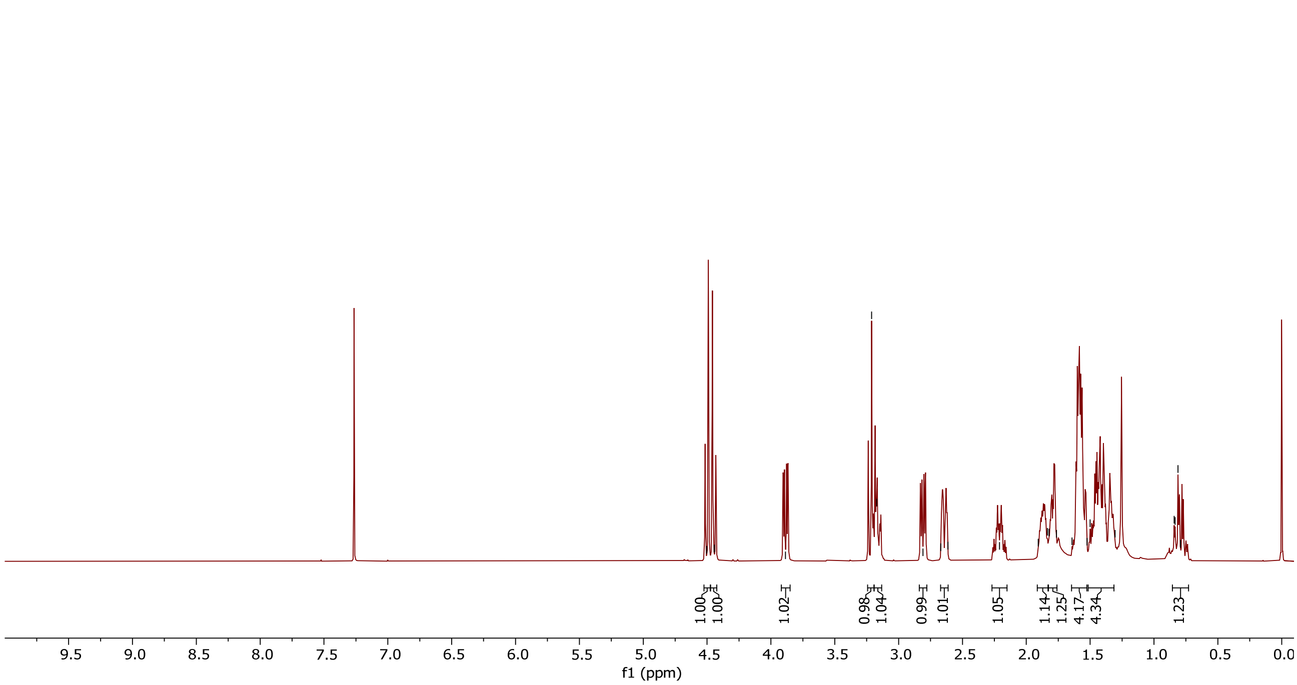

III) X- ray crystallographic data for compounds 13 and *cis*-15a

Compound 13

Sample preparation:

A colorless prism-like specimen with approximate dimensions 0.148 mm x 0.101 mm x 0.081 mm, was used for the X-ray crystallographic analysis.

Instrument and experimental conditions:

The X-ray intensity data were measured at 100K on a D8 Venture system equipped with a multilayer monochromator and a Mo microfocus (λ = 0.71073 Å). The frames were integrated with the Bruker SAINT software package using a narrow-frame algorithm. The integration of the data using a monoclinic unit cell yielded a total of 6813 reflections to a maximum θ angle of 26.38° (0.80 Å resolution), of which 3132 were independent (average redundancy 2.175, completeness = 99.9%, R_{int} = 5.23%, R_{sig} = 7.22%) and 2484 (79.31%) were greater than $2\sigma(F^2)$. The final cell constants of a = 8.5387(5)Å, b = 11.7935(7) Å, c = 8.7650(5) Å, β = 118.814(2)°, volume = 773.36(8) Å^3, are based upon the refinement of the XYZ-centroids of reflections above 20 σ(I). Data were corrected for absorption effects using the multi-scan method (SADABS). The calculated minimum and maximum transmission coefficients (based on crystal size) are 0.5877 and 0.7454.

The structure was solved and refined using the Bruker SHELXTL Software Package, using the space group $P2_1$, with Z = 2 for the formula unit, $C_{18}H_{25}NO_2$. The final anisotropic full-matrix least-squares refinement on F^2 with 194 variables converged at R1 = 5.19%, for the observed data and wR2 = 18.76% for all data. The goodness-of-fit was 0.717. The largest peak in the final difference electron density synthesis was 0.184 e^-/Å^3 and the largest hole was -0.259 e^-/Å^3 with an RMS deviation of 0.046e^-/Å^3. On the basis of the final model, the calculated density was 1.234 g/cm^3 and F(000), 312 e^-.

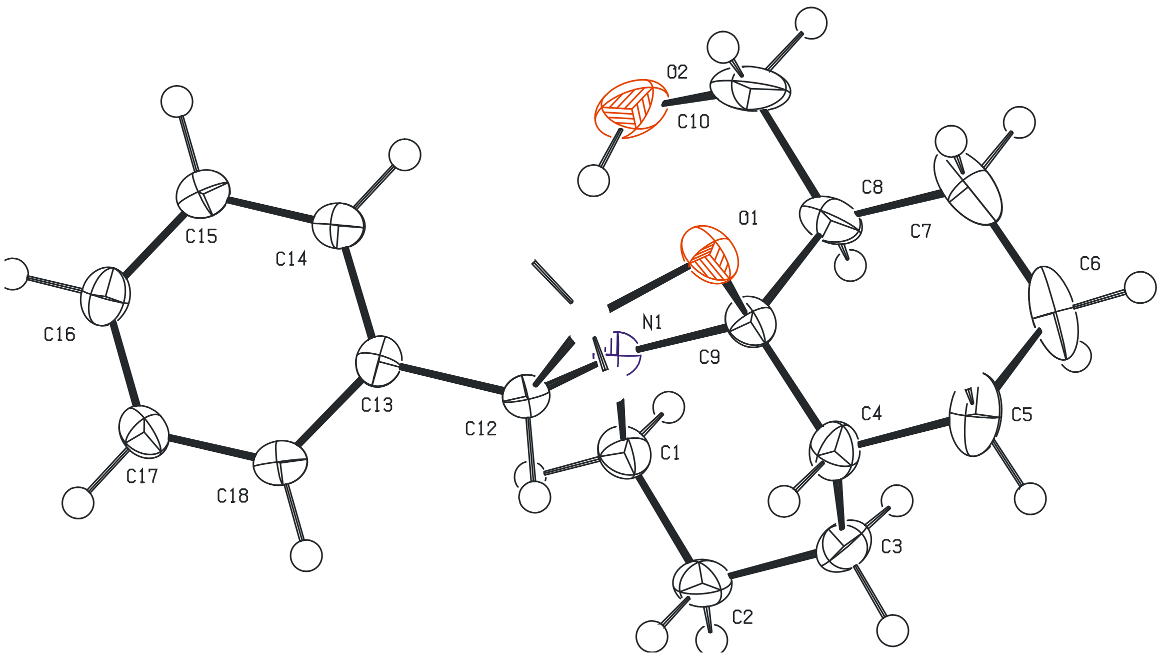

Figure S1. ORTEP diagram and atomic numbering of **13**. Ellipsoids are drawn at 50% probability level.

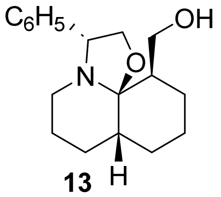

13

Table S1. Sample and crystal data for **13**

Identification code	D66ABB43A	
Chemical formula	$C_{18}H_{25}NO_2$	
Formula weight	287.39 g/mol	
Temperature	100(2) K	
Wavelength	0.71073 Å	
Crystal system	monoclinic	
Space group	P 1 21 1	
Unit cell dimensions	a = 8.5387(5) Å	α = 90°
	b = 11.7935(7) Å	β = 118.814(2)°
	c = 8.7650(5) Å	γ = 90°
Volume	773.36(8) $Å^3$	
Z	2	
Density (calculated)	1.234 g/cm^3	
Absorption coefficient	0.079 mm^{-1}	
F(000)	312	

Table S2. Data collection and structure refinement for **13**

Theta range for data collection	2.65 to 26.38°	
Index ranges	-9<=h<=10, -14<=k<=14, -10<=l<=10	
Reflections collected	6813	
Independent reflections	3132 [R(int) = 0.0523]	
Refinement method	Full-matrix least-squares on F^2	
Refinement program	SHELXL-2019/1 (Sheldrick, 2019)	
Function minimized	$\Sigma\ w(F_o^2 - F_c^2)^2$	
Data / restraints / parameters	3132 / 2 / 194	
Goodness-of-fit on F^2	0.716	
Final R indices	2484 data; I>2σ(I)	R1 = 0.0519, wR2 = 0.1547
	all data	R1 = 0.0722, wR2 = 0.1876
Weighting scheme	$w=1/[\sigma^2(F_o^2)+(0.2000P)^2]$ where $P=(F_o^2+2F_c^2)/3$	
Absolute structure parameter	-1.9(10)	
Largest diff. peak and hole	0.184 and -0.259 eÅ^{-3}	
R.M.S. deviation from mean	0.046 eÅ^{-3}	

ANNEX

Table S3. Atomic coordinates and equivalent isotropic atomic displacement parameters (Å^2) for **13** **(**D66ABB43A).

U(eq) is defined as one third of the trace of the orthogonalized U_{ij} tensor.

	x/a	y/b	z/c	U(eq)
C1	0.1394(6)	0.4052(3)	0.2155(5)	0.0276(9)
C2	0.2265(7)	0.2956(3)	0.1999(6)	0.0337(10)
C3	0.3771(6)	0.3246(3)	0.1598(6)	0.0332(10)
C4	0.5143(6)	0.4042(4)	0.2966(6)	0.0304(9)
C5	0.6693(6)	0.4328(5)	0.2614(7)	0.0432(12)
C6	0.6146(7)	0.5153(6)	0.1101(7)	0.0519(15)
C7	0.5349(8)	0.6215(5)	0.1416(8)	0.0517(14)
C8	0.3717(6)	0.5950(3)	0.1644(6)	0.0325(10)
C9	0.4230(5)	0.5120(3)	0.3156(5)	0.0250(9)
C10	0.2890(8)	0.7051(4)	0.1866(7)	0.0455(13)
C11	0.5203(5)	0.5171(4)	0.6125(5)	0.0281(9)
C12	0.3397(5)	0.4539(3)	0.5245(5)	0.0223(8)
C13	0.2139(5)	0.4849(3)	0.5941(5)	0.0232(8)
C14	0.1662(6)	0.5979(3)	0.5970(6)	0.0290(9)
C15	0.0540(6)	0.6265(3)	0.6644(6)	0.0295(9)
C16	0.9854(6)	0.5425(4)	0.7267(5)	0.0282(9)
C17	0.0282(6)	0.4292(3)	0.7205(5)	0.0269(9)
C18	0.1434(5)	0.4021(3)	0.6554(5)	0.0239(8)
N1	0.2663(4)	0.4866(3)	0.3405(4)	0.0223(7)

	x/a	y/b	z/c	U(eq)
O1	0.5395(4)	0.5693(2)	0.4745(4)	0.0310(7)
O2	0.1225(6)	0.6896(3)	0.1825(5)	0.0522(10)

Table S4. Bond lengths (Å) for **13** (D66ABB43A).

C1-N1	1.467(5)	C1-C2	1.529(6)
C1-H1A	0.990000	C1-H1B	0.990000
C2-C3	1.526(6)	C2-H2A	0.990000
C2-H2B	0.990000	C3-C4	1.529(6)
C3-H3A	0.990000	C3-H3B	0.990000
C4-C5	1.534(6)	C4-C9	1.542(6)
C4-H4	1.000000	C5-C6	1.525(8)
C5-H5A	0.990000	C5-H5B	0.990000
C6-C7	1.513(9)	C6-H6A	0.990000
C6-H6B	0.990000	C7-C8	1.533(7)
C7-H7A	0.990000	C7-H7B	0.990000
C8-C9	1.532(5)	C8-C10	1.534(7)
C8-H8	1.000000	C9-O1	1.432(5)
C9-N1	1.486(5)	C10-O2	1.416(7)
C10-H10A	0.990000	C10-H10B	0.990000
C11-O1	1.435(5)	C11-C12	1.543(5)
C11-H11A	0.990000	C11-H11B	0.990000
C12-N1	1.473(5)	C12-C13	1.513(5)
C12-H12	1.000000	C13-C18	1.384(5)
C13-C14	1.398(5)	C14-C15	1.388(6)
C14-H14	0.950000	C15-C16	1.390(6)
C15-H15	0.950000	C16-C17	1.394(6)

C16-H16	0.950000	C17-C18	1.392(6)
C17-H17	0.950000	C18-H18	0.950000
O2-H2	0.859(14)		

Table S5. Bond angles (°) for **13** (D66ABB43A).

N1-C1-C2	113.8(3)	N1-C1-H1A	108.800000
C2-C1-H1A	108.800000	N1-C1-H1B	108.800000
C2-C1-H1B	108.800000	H1A-C1-H1B	107.700000
C3-C2-C1	109.4(3)	C3-C2-H2A	109.800000
C1-C2-H2A	109.800000	C3-C2-H2B	109.800000
C1-C2-H2B	109.800000	H2A-C2-H2B	108.300000
C2-C3-C4	111.7(3)	C2-C3-H3A	109.300000
C4-C3-H3A	109.300000	C2-C3-H3B	109.300000
C4-C3-H3B	109.300000	H3A-C3-H3B	107.900000
C3-C4-C5	112.7(4)	C3-C4-C9	110.8(3)
C5-C4-C9	111.7(4)	C3-C4-H4	107.100000
C5-C4-H4	107.100000	C9-C4-H4	107.100000
C6-C5-C4	112.5(4)	C6-C5-H5A	109.100000
C4-C5-H5A	109.100000	C6-C5-H5B	109.100000
C4-C5-H5B	109.100000	H5A-C5-H5B	107.800000
C7-C6-C5	110.2(4)	C7-C6-H6A	109.600000
C5-C6-H6A	109.600000	C7-C6-H6B	109.600000
C5-C6-H6B	109.600000	H6A-C6-H6B	108.100000
C6-C7-C8	111.9(4)	C6-C7-H7A	109.200000
C8-C7-H7A	109.200000	C6-C7-H7B	109.200000
C8-C7-H7B	109.200000	H7A-C7-H7B	107.900000
C9-C8-C7	110.1(4)	C9-C8-C10	112.9(4)
C7-C8-C10	110.3(4)	C9-C8-H8	107.800000

C7-C8-H8	107.800000	C10-C8-H8	107.800000
O1-C9-N1	101.8(3)	O1-C9-C8	108.3(3)
N1-C9-C8	110.7(3)	O1-C9-C4	110.1(3)
N1-C9-C4	112.8(3)	C8-C9-C4	112.5(3)
O2-C10-C8	113.9(4)	O2-C10-H10A	108.800000
C8-C10-H10A	108.800000	O2-C10-H10B	108.800000
C8-C10-H10B	108.800000	H10A-C10-H10B	107.700000
O1-C11-C12	106.2(3)	O1-C11-H11A	110.500000
C12-C11-H11A	110.500000	O1-C11-H11B	110.500000
C12-C11-H11B	110.500000	H11A-C11-H11B	108.700000
N1-C12-C13	111.6(3)	N1-C12-C11	102.5(3)
C13-C12-C11	114.4(3)	N1-C12-H12	109.400000
C13-C12-H12	109.400000	C11-C12-H12	109.400000
C18-C13-C14	118.7(3)	C18-C13-C12	120.8(3)
C14-C13-C12	120.5(3)	C15-C14-C13	120.5(4)
C15-C14-H14	119.800000	C13-C14-H14	119.800000
C14-C15-C16	120.1(4)	C14-C15-H15	119.900000
C16-C15-H15	119.900000	C15-C16-C17	120.0(4)
C15-C16-H16	120.000000	C17-C16-H16	120.000000
C16-C17-C18	119.1(4)	C16-C17-H17	120.500000
C18-C17-H17	120.500000	C13-C18-C17	121.5(3)
C13-C18-H18	119.200000	C17-C18-H18	119.200000
C1-N1-C12	114.6(3)	C1-N1-C9	114.6(3)
C12-N1-C9	106.0(3)	C9-O1-C11	108.0(3)

C10-O2-H2	105.(5)

Table S6. Anisotropic atomic displacement parameters (Å^2) for **13** (D66ABB43A).

The anisotropic atomic displacement factor exponent takes the form: $-2\pi^2[h^2 a^{*2} U_{11} + ... + 2 h k a^* b^* U_{12}]$

	U_{11}	U_{22}	U_{33}	U_{23}	U_{13}	U_{12}
C1	0.025(2)	0.0290(19)	0.028(2)	-0.0056(16)	0.0119(18)	-0.0082(17)
C2	0.048(3)	0.0223(18)	0.033(2)	-0.0046(16)	0.022(2)	-0.0050(18)
C3	0.046(3)	0.027(2)	0.033(2)	0.0004(17)	0.024(2)	0.0067(19)
C4	0.031(2)	0.037(2)	0.024(2)	0.0043(17)	0.0138(18)	0.0067(18)
C5	0.030(2)	0.071(4)	0.032(2)	0.002(2)	0.017(2)	0.006(2)
C6	0.039(3)	0.090(4)	0.033(3)	0.010(3)	0.023(2)	-0.008(3)
C7	0.051(3)	0.063(3)	0.040(3)	0.007(2)	0.021(3)	-0.022(3)
C8	0.041(3)	0.0254(19)	0.027(2)	0.0052(16)	0.013(2)	-0.0050(18)
C9	0.023(2)	0.0264(18)	0.022(2)	-0.0022(14)	0.0076(16)	-0.0063(16)
C10	0.070(4)	0.020(2)	0.039(3)	0.0052(17)	0.021(3)	0.000(2)
C11	0.025(2)	0.036(2)	0.025(2)	-0.0034(16)	0.0140(18)	-0.0054(17)
C12	0.0248(19)	0.0195(16)	0.026(2)	-0.0022(14)	0.0144(17)	-0.0002(15)
C13	0.0194(18)	0.0275(19)	0.0222(18)	-0.0029(15)	0.0097(16)	-0.0014(15)
C14	0.033(2)	0.0221(19)	0.037(2)	0.0015(16)	0.021(2)	0.0012(16)
C15	0.033(2)	0.0229(18)	0.036(2)	-0.0001(16)	0.019(2)	0.0024(16)
C16	0.024(2)	0.034(2)	0.025(2)	-0.0013(16)	0.0104(17)	0.0014(17)
C17	0.029(2)	0.029(2)	0.023(2)	0.0010(14)	0.0127(18)	-0.0026(16)
C18	0.0238(19)	0.0217(17)	0.0238(19)	-0.0002(15)	0.0094(17)	0.0003(15)
N1	0.0226(16)	0.0211(15)	0.0232(16)	0.0003(12)	0.0109(14)	0.0005(12)
O1	0.0295(16)	0.0338(15)	0.0263(16)	-0.0029(12)	0.0106(13)	-0.0123(12)
O2	0.074(3)	0.0345(18)	0.051(2)	0.0123(16)	0.032(2)	0.0272(18)

Table S7. Hydrogen atomic coordinates and isotropic atomic displacement parameters (Å^2) for **13** (D66ABB43A).

	x/a	y/b	z/c	U(eq)
H1A	0.0764	0.4416	0.0996	0.033000
H1B	0.0490	0.3859	0.2513	0.033000
H2A	0.2750	0.2525	0.3102	0.040000
H2B	0.1363	0.2479	0.1056	0.040000
H3A	0.3260	0.3611	0.0439	0.040000
H3B	0.4374	0.2539	0.1556	0.040000
H4	0.5666	0.3635	0.4107	0.036000
H5A	0.7683	0.4664	0.3677	0.052000
H5B	0.7140	0.3620	0.2352	0.052000
H6A	0.7205	0.5355	0.0977	0.062000
H6B	0.5259	0.4788	0.0006	0.062000
H7A	0.4992	0.6739	0.0420	0.062000
H7B	0.6266	0.6601	0.2474	0.062000
H8	0.2805	0.5571	0.0556	0.039000
H10A	0.3736	0.7403	0.2990	0.055000
H10B	0.2725	0.7584	0.0927	0.055000
H11A	0.5207	0.5752	0.6942	0.034000
H11B	0.6195	0.4633	0.6780	0.034000
H12	0.3618	0.3702	0.5363	0.027000
H14	0.2109	0.6555	0.5526	0.035000
H15	0.0241	0.7037	0.6679	0.035000

	x/a	y/b	z/c	U(eq)
H16	-0.0907	0.5624	0.7735	0.034000
H17	-0.0206	0.3712	0.7603	0.032000
H18	0.1744	0.3250	0.6529	0.029000
H2	0.133(9)	0.626(3)	0.235(8)	0.058(18)

Table S8. Hydrogen Bonds (Angstrom, Deg) for **13** (D66ABB43A)

Donor – H..Aceptor [ARU]	D – H	H…A	D…A	D-H…A	
O2 -- H2 .. N1	0.86(4)	1.98(4)	2.741(4)	147(6)	.
C2 -- H2B .. O2 [-x,-1/2+y,-z]	0.9900	2.5200	3.470(6)	161.00	
C10 -- H10A .. O1	0.9900	2.5200	2.884(5)	101.00	.
C17 -- H17 .. O2 [-x,-1/2+y,1-z]	0.9500	2.4600	3.382(5)	165.00	

Compound *cis*-15a

Sample preparation:

A yellow prism-like specimen of $C_{25}H_{32}NO_3S$, approximate dimensions 0.051 mm x 0.213 mm x 0.416 mm, was used for the X-ray crystallographic analysis.

Instrument and experimental conditions:

The X-ray intensity data were measured on a D8 Venture system equipped with a multilayer monochromator and a Mo microfocus (λ = 0.71073 Å). The frames were integrated with the Bruker SAINT software package using a narrow-frame algorithm. The integration of the data using an orthorhombic unit cell yielded a total of 24306 reflections to a maximum θ angle of 26.42° (0.80 Å resolution), of which 4663 were independent (average redundancy 5.213, completeness = 99.2%, R_{int} = 17.48%, R_{sig} = 11.76%) and 3123 (66.97%) were greater than $2\sigma(F^2)$. The final cell constants of a = 8.369(4) Å, b = 14.428(8) Å, c = 18.957(10) Å, volume = 2289.(2) $Å^3$, are based upon the refinement of the XYZ-centroids of reflections above 20 σ(I). Data were corrected for absorption effects using the Multi-Scan method (SADABS). The calculated minimum and maximum transmission coefficients (based on crystal size) are 0.4283 and 0.7454.

The structure was solved and refined using the Bruker SHELXTL Software Package, using the space group P 21 21 21, with Z = 4 for the formula unit, $C_{25}H_{32}NO_3S$. The final anisotropic full-matrix least-squares refinement on F^2 with 272 variables converged at R1 = 5.08%, for the observed data and wR2 = 14.12% for all data. The goodness-of-fit was 1.003. The largest peak in the final difference electron density synthesis was 0.282 $e^-/Å^3$ and the largest hole was -0.278 $e^-/Å^3$ with an RMS deviation of 0.067 $e^-/Å^3$. On the basis of the final model, the calculated density was 1.238 g/cm^3 and F(000), 916 e^-.

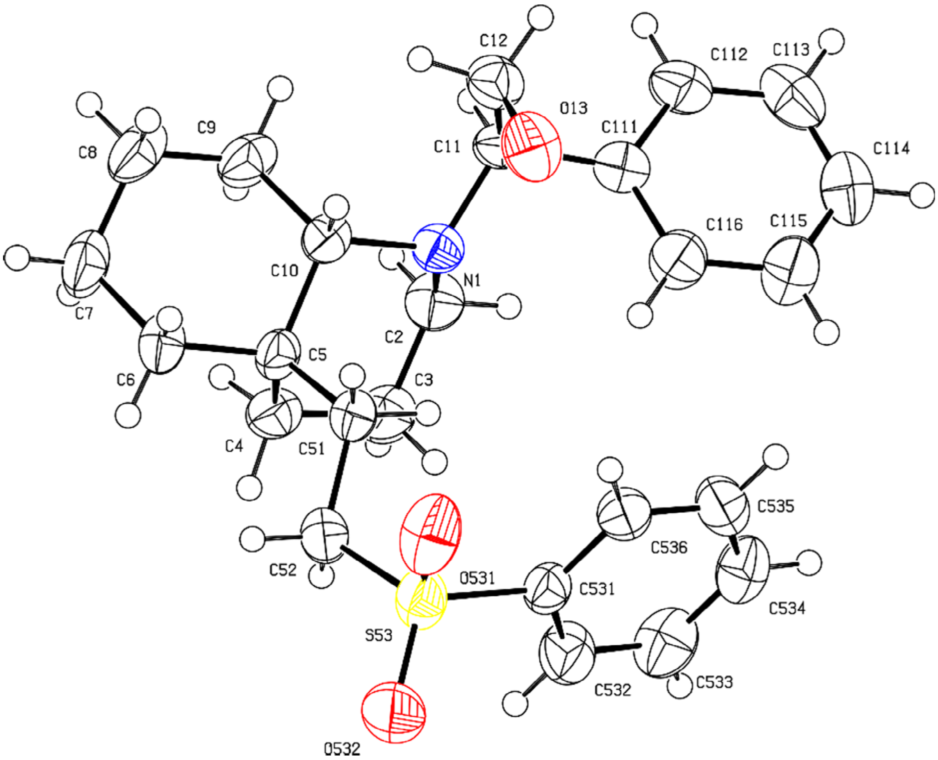

Figure S2. ORTEP diagram and atomic numbering of *cis*-**15a**. Ellipsoids are drawn at 50% probability level.

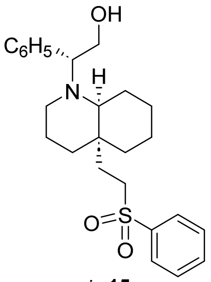

cis-**15a**

Table S9. Crystal data and structure refinement for *cis*-**15a** (D57YB72_0m_a)

Identification code	D57YB72_0m_a	
Empirical formula	C25 H32 N O3 S	
Formula weight	426.57	
Temperature	287(2) K	
Wavelength	0.71073 Å	
Crystal system	Orthorhombic	
Space group	P 21 21 21	
Unit cell dimensions	a = 8.369(4) Å	α= 90°.
	b = 14.428(8) Å	β= 90°.
	c = 18.957(10) Å	γ = 90°.
Volume	2289(2) Å^3	
Z	4	
Density (calculated)	1.238 Mg/m^3	
Absorption coefficient	0.167 mm^{-1}	
F(000)	916	
Crystal size	0.416 x 0.213 x 0.051 mm^3	
Theta range for data collection	2.571 to 26.418°.	
Index ranges	-10<=h<=10, -18<=k<=17, -23<=l<=23	
Reflections collected	24306	
Independent reflections	4663 [R(int) = 0.1748]	
Completeness to theta = 25.242°	99.9 %	
Absorption correction	Semi-empirical from equivalents	
Max. and min. transmission	0.7454 and 0.4283	
Refinement method	Full-matrix least-squares on F^2	
Data / restraints / parameters	4663 / 0 / 272	
Goodness-of-fit on F^2	1.003	
Final R indices [I>2sigma(I)]	R1 = 0.0508, wR2 = 0.1173	
R indices (all data)	R1 = 0.0948, wR2 = 0.1412	
Absolute structure parameter	-0.16(10)	
Extinction coefficient	0.020(3)	
Largest diff. peak and hole	0.282 and -0.278 e.Å^{-3}	

Table S10. Atomic coordinates (x 10^4) and equivalent isotropic displacement parameters ($Å^2$x 10^3) for *cis*-**15a** (D57YB72_0m_a). U(eq) is defined as one third of the trace of the orthogonalized U^{ij} tensor.

	x	y	z	U(eq)
S(53)	6994(1)	3781(1)	1065(1)	52(1)
O(13)	5095(3)	6178(2)	3331(2)	59(1)
O(531)	7817(3)	4651(3)	1102(2)	69(1)
O(532)	7421(4)	3150(3)	509(2)	80(1)
N(1)	4286(4)	4248(2)	3581(2)	39(1)
C(2)	3303(5)	3410(3)	3617(2)	50(1)
C(3)	3922(5)	2663(3)	3131(3)	54(1)
C(4)	5677(5)	2461(3)	3262(2)	46(1)
C(5)	6686(4)	3348(3)	3247(2)	39(1)
C(6)	8447(5)	3153(4)	3451(2)	50(1)
C(7)	8691(6)	2890(4)	4229(2)	59(1)
C(8)	7999(6)	3618(4)	4715(2)	60(1)
C(9)	6237(5)	3762(4)	4558(2)	51(1)
C(10)	5970(4)	4050(3)	3778(2)	37(1)
C(11)	3534(4)	5012(3)	3970(2)	42(1)
C(12)	4612(5)	5861(3)	4015(2)	50(1)
C(51)	6651(4)	3808(3)	2506(2)	41(1)
C(52)	7211(5)	3207(3)	1886(2)	50(1)
C(111)	1963(5)	5309(3)	3630(2)	42(1)
C(112)	791(5)	5691(3)	4049(2)	55(1)
C(113)	-586(6)	6060(4)	3760(3)	68(2)
C(114)	-826(5)	6035(4)	3044(3)	66(1)
C(115)	312(5)	5649(4)	2613(3)	64(1)
C(116)	1696(5)	5288(4)	2905(2)	55(1)
C(531)	4926(4)	4023(3)	1003(2)	40(1)
C(532)	3865(5)	3326(4)	867(3)	58(1)
C(533)	2251(6)	3528(4)	829(3)	64(1)
C(534)	1731(5)	4417(4)	919(2)	56(1)
C(535)	2801(5)	5112(3)	1049(3)	55(1)
C(536)	4409(4)	4920(3)	1098(2)	45(1)

Table S11. Bond lengths [Å] and angles [°] for *cis*-**15a** (D57YB72_0m_a).

S(53)-O(531)	1.434(4)
S(53)-O(532)	1.438(4)
S(53)-C(531)	1.770(4)
S(53)-C(52)	1.772(4)
O(13)-C(12)	1.433(5)
N(1)-C(2)	1.464(5)
N(1)-C(11)	1.469(5)
N(1)-C(10)	1.486(5)
C(2)-C(3)	1.509(7)
C(2)-H(2A)	0.9700
C(2)-H(2B)	0.9700
C(3)-C(4)	1.518(6)
C(3)-H(3A)	0.9700
C(3)-H(3B)	0.9700
C(4)-C(5)	1.534(6)
C(4)-H(4A)	0.9700
C(4)-H(4B)	0.9700
C(5)-C(10)	1.549(6)
C(5)-C(6)	1.549(5)
C(5)-C(51)	1.553(5)
C(6)-C(7)	1.537(6)
C(6)-H(6A)	0.9700
C(6)-H(6B)	0.9700
C(7)-C(8)	1.513(7)
C(7)-H(7A)	0.9700
C(7)-H(7B)	0.9700
C(8)-C(9)	1.518(6)
C(8)-H(8A)	0.9700
C(8)-H(8B)	0.9700
C(9)-C(10)	1.552(5)
C(9)-H(9A)	0.9700
C(9)-H(9B)	0.9700
C(10)-H(10)	0.9800
C(11)-C(12)	1.523(6)
C(11)-C(111)	1.526(6)
C(11)-H(11)	0.9800
C(12)-H(12A)	0.9700

C(12)-H(12B)	0.9700
C(51)-C(52)	1.535(6)
C(51)-H(51A)	0.9700
C(51)-H(51B)	0.9700
C(52)-H(52A)	0.9700
C(52)-H(52B)	0.9700
C(111)-C(112)	1.377(6)
C(111)-C(116)	1.393(6)
C(112)-C(113)	1.383(7)
C(112)-H(112)	0.9300
C(113)-C(114)	1.373(7)
C(113)-H(113)	0.9300
C(114)-C(115)	1.373(7)
C(114)-H(114)	0.9300
C(115)-C(116)	1.385(6)
C(115)-H(115)	0.9300
C(116)-H(116)	0.9300
C(531)-C(532)	1.367(6)
C(531)-C(536)	1.376(6)
C(532)-C(533)	1.383(7)
C(532)-H(532)	0.9300
C(533)-C(534)	1.365(7)
C(533)-H(533)	0.9300
C(534)-C(535)	1.366(7)
C(534)-H(534)	0.9300
C(535)-C(536)	1.377(6)
C(535)-H(535)	0.9300
C(536)-H(536)	0.9300

O(531)-S(53)-O(532)	118.1(2)
O(531)-S(53)-C(531)	107.5(2)
O(532)-S(53)-C(531)	108.6(2)
O(531)-S(53)-C(52)	108.5(2)
O(532)-S(53)-C(52)	108.8(2)
C(531)-S(53)-C(52)	104.5(2)
C(2)-N(1)-C(11)	110.8(3)
C(2)-N(1)-C(10)	111.3(3)
C(11)-N(1)-C(10)	115.1(3)
N(1)-C(2)-C(3)	111.6(3)

N(1)-C(2)-H(2A)	109.3
C(3)-C(2)-H(2A)	109.3
N(1)-C(2)-H(2B)	109.3
C(3)-C(2)-H(2B)	109.3
H(2A)-C(2)-H(2B)	108.0
C(2)-C(3)-C(4)	111.7(4)
C(2)-C(3)-H(3A)	109.3
C(4)-C(3)-H(3A)	109.3
C(2)-C(3)-H(3B)	109.3
C(4)-C(3)-H(3B)	109.3
H(3A)-C(3)-H(3B)	107.9
C(3)-C(4)-C(5)	111.7(4)
C(3)-C(4)-H(4A)	109.3
C(5)-C(4)-H(4A)	109.3
C(3)-C(4)-H(4B)	109.3
C(5)-C(4)-H(4B)	109.3
H(4A)-C(4)-H(4B)	107.9
C(4)-C(5)-C(10)	108.7(3)
C(4)-C(5)-C(6)	111.5(3)
C(10)-C(5)-C(6)	109.0(3)
C(4)-C(5)-C(51)	111.3(3)
C(10)-C(5)-C(51)	107.5(3)
C(6)-C(5)-C(51)	108.7(3)
C(7)-C(6)-C(5)	114.3(4)
C(7)-C(6)-H(6A)	108.7
C(5)-C(6)-H(6A)	108.7
C(7)-C(6)-H(6B)	108.7
C(5)-C(6)-H(6B)	108.7
H(6A)-C(6)-H(6B)	107.6
C(8)-C(7)-C(6)	111.2(4)
C(8)-C(7)-H(7A)	109.4
C(6)-C(7)-H(7A)	109.4
C(8)-C(7)-H(7B)	109.4
C(6)-C(7)-H(7B)	109.4
H(7A)-C(7)-H(7B)	108.0
C(7)-C(8)-C(9)	110.3(4)
C(7)-C(8)-H(8A)	109.6
C(9)-C(8)-H(8A)	109.6
C(7)-C(8)-H(8B)	109.6

C(9)-C(8)-H(8B)	109.6
H(8A)-C(8)-H(8B)	108.1
C(8)-C(9)-C(10)	111.3(3)
C(8)-C(9)-H(9A)	109.4
C(10)-C(9)-H(9A)	109.4
C(8)-C(9)-H(9B)	109.4
C(10)-C(9)-H(9B)	109.4
H(9A)-C(9)-H(9B)	108.0
N(1)-C(10)-C(5)	109.2(3)
N(1)-C(10)-C(9)	115.3(3)
C(5)-C(10)-C(9)	112.8(3)
N(1)-C(10)-H(10)	106.3
C(5)-C(10)-H(10)	106.3
C(9)-C(10)-H(10)	106.3
N(1)-C(11)-C(12)	112.2(3)
N(1)-C(11)-C(111)	111.6(3)
C(12)-C(11)-C(111)	108.0(3)
N(1)-C(11)-H(11)	108.3
C(12)-C(11)-H(11)	108.3
C(111)-C(11)-H(11)	108.3
O(13)-C(12)-C(11)	111.9(3)
O(13)-C(12)-H(12A)	109.2
C(11)-C(12)-H(12A)	109.2
O(13)-C(12)-H(12B)	109.2
C(11)-C(12)-H(12B)	109.2
H(12A)-C(12)-H(12B)	107.9
C(52)-C(51)-C(5)	116.5(4)
C(52)-C(51)-H(51A)	108.2
C(5)-C(51)-H(51A)	108.2
C(52)-C(51)-H(51B)	108.2
C(5)-C(51)-H(51B)	108.2
H(51A)-C(51)-H(51B)	107.3
C(51)-C(52)-S(53)	112.2(3)
C(51)-C(52)-H(52A)	109.2
S(53)-C(52)-H(52A)	109.2
C(51)-C(52)-H(52B)	109.2
S(53)-C(52)-H(52B)	109.2
H(52A)-C(52)-H(52B)	107.9
C(112)-C(111)-C(116)	117.7(4)

C(112)-C(111)-C(11)	118.8(4)
C(116)-C(111)-C(11)	123.3(4)
C(111)-C(112)-C(113)	121.2(4)
C(111)-C(112)-H(112)	119.4
C(113)-C(112)-H(112)	119.4
C(114)-C(113)-C(112)	120.2(5)
C(114)-C(113)-H(113)	119.9
C(112)-C(113)-H(113)	119.9
C(115)-C(114)-C(113)	119.9(4)
C(115)-C(114)-H(114)	120.1
C(113)-C(114)-H(114)	120.1
C(114)-C(115)-C(116)	119.7(5)
C(114)-C(115)-H(115)	120.2
C(116)-C(115)-H(115)	120.2
C(115)-C(116)-C(111)	121.3(4)
C(115)-C(116)-H(116)	119.3
C(111)-C(116)-H(116)	119.3
C(532)-C(531)-C(536)	120.9(4)
C(532)-C(531)-S(53)	120.2(4)
C(536)-C(531)-S(53)	119.0(3)
C(531)-C(532)-C(533)	119.2(5)
C(531)-C(532)-H(532)	120.4
C(533)-C(532)-H(532)	120.4
C(534)-C(533)-C(532)	120.2(5)
C(534)-C(533)-H(533)	119.9
C(532)-C(533)-H(533)	119.9
C(533)-C(534)-C(535)	120.1(4)
C(533)-C(534)-H(534)	119.9
C(535)-C(534)-H(534)	119.9
C(534)-C(535)-C(536)	120.4(5)
C(534)-C(535)-H(535)	119.8
C(536)-C(535)-H(535)	119.8
C(531)-C(536)-C(535)	119.1(4)
C(531)-C(536)-H(536)	120.4
C(535)-C(536)-H(536)	120.4

Symmetry transformations used to generate equivalent atoms:

Table S12. Anisotropic displacement parameters ($Å^2$x 10^3) for *cis*-**15a** (D57YB72_0m_a). The anisotropic displacement factor exponent takes the form: $-2\pi^2[h^2 a^{*2}U^{11} + ... + 2 h k a^* b^* U^{12}]$

	U^{11}	U^{22}	U^{33}	U^{23}	U^{13}	U^{12}
S(53)	42(1)	73(1)	42(1)	7(1)	5(1)	13(1)
O(13)	60(2)	48(2)	69(2)	16(2)	7(2)	1(2)
O(531)	44(1)	93(3)	71(2)	26(2)	2(2)	-9(2)
O(532)	83(2)	116(4)	41(2)	-9(2)	12(2)	42(2)
N(1)	40(2)	35(2)	42(2)	-1(2)	0(1)	-3(2)
C(2)	48(2)	44(3)	58(3)	1(2)	-2(2)	-10(2)
C(3)	59(3)	44(3)	60(3)	-1(2)	-10(2)	-11(2)
C(4)	61(2)	36(2)	41(2)	1(2)	-5(2)	-3(2)
C(5)	45(2)	36(2)	35(2)	6(2)	-8(2)	3(2)
C(6)	47(2)	51(3)	51(3)	8(2)	-9(2)	8(2)
C(7)	61(3)	58(3)	59(3)	14(2)	-22(2)	8(2)
C(8)	75(3)	58(3)	46(2)	10(2)	-24(2)	-8(3)
C(9)	68(3)	47(3)	37(2)	6(2)	-9(2)	-7(3)
C(10)	44(2)	32(2)	37(2)	3(2)	-5(2)	-5(2)
C(11)	51(2)	41(2)	33(2)	-3(2)	4(2)	0(2)
C(12)	53(2)	40(3)	58(3)	-6(2)	-2(2)	0(2)
C(51)	44(2)	42(2)	38(2)	4(2)	-5(2)	5(2)
C(52)	49(2)	55(3)	45(2)	1(2)	-3(2)	15(2)
C(111)	45(2)	39(2)	41(2)	1(2)	6(2)	-5(2)
C(112)	59(2)	56(3)	49(3)	-7(2)	11(2)	4(2)
C(113)	57(3)	59(4)	87(4)	-10(3)	15(3)	11(3)
C(114)	49(2)	66(4)	82(4)	10(3)	-4(3)	10(3)
C(115)	54(3)	78(4)	60(3)	10(3)	-5(2)	3(3)
C(116)	46(2)	71(4)	48(3)	0(2)	4(2)	4(2)
C(531)	42(2)	45(3)	35(2)	4(2)	-3(2)	3(2)
C(532)	63(3)	42(3)	68(3)	4(2)	-12(2)	0(2)
C(533)	57(3)	61(4)	73(3)	8(3)	-12(2)	-19(3)
C(534)	42(2)	72(4)	55(3)	13(3)	-3(2)	0(2)
C(535)	53(2)	49(3)	61(3)	0(2)	1(2)	11(2)
C(536)	43(2)	42(3)	50(2)	-3(2)	-3(2)	-3(2)

Table S13. Hydrogen coordinates (x 10^4) and isotropic displacement parameters (Å^{2}x 10 ^{3}) for *cis*-**15a** (D57YB72_0m_a).

	x	y	z	U(eq)
H(2A)	2212	3561	3488	60
H(2B)	3296	3179	4098	60
H(3A)	3779	2857	2646	65
H(3B)	3307	2101	3202	65
H(4A)	6065	2036	2904	56
H(4B)	5797	2163	3718	56
H(6A)	8847	2653	3157	60
H(6B)	9079	3700	3348	60
H(7A)	8181	2299	4321	71
H(7B)	9825	2822	4323	71
H(8A)	8569	4198	4651	71
H(8B)	8130	3424	5201	71
H(9A)	5816	4239	4866	61
H(9B)	5660	3192	4653	61
H(10)	6558	4631	3711	45
H(11)	3308	4799	4451	50
H(12A)	4049	6355	4256	60
H(12B)	5554	5708	4290	60
H(51A)	5565	4007	2411	50
H(51B)	7313	4359	2521	50
H(52A)	6595	2637	1878	60
H(52B)	8325	3044	1954	60
H(112)	928	5700	4536	66
H(113)	-1351	6327	4052	81
H(114)	-1757	6279	2851	79
H(115)	155	5630	2128	77
H(116)	2462	5026	2611	66
H(532)	4223	2722	800	70
H(533)	1519	3057	742	76
H(534)	645	4550	893	68
H(535)	2441	5717	1104	65
H(536)	5136	5391	1194	54

C(5)	- C(4)	C(6)	C(10)	C(51)	sp3	S
C(10)	- N(1)	C(5)	C(9)	H(10)	sp3	S
C(11)	- N(1)	C(12)	C(111)	H(11)	sp3	R

IV) Computational data

Computational Studies

Density functional theory calculations were carried out to locate the stationary points involved in the reaction and determine the free energy profiles in the gas phase and in solution. To this end, calculations were performed using the M062X[4] method and the 6-31G(d,p)[5,6] basis set in conjunction with the Gaussian16 program.[7] Full geometry optimizations were performed to locate the energy minima and transitions states, and the nature of the stationary points was verified upon inspection of the harmonic vibrational frequencies (with zero and one imaginary frequency for energy minimum and transition state structures, respectively). The connection between transition states and the corresponding 'reactant' and 'product' states was verified by using the Intrinsic Reaction Coordinate calculations.

The relative stabilities in the gas phase were estimated by adding the zero-point energy, thermal and entropy corrections to the relative energies estimated at 1 atm and 298 K. Finally, the relative stability in dioxane and methanol was estimated by adding the corresponding solvation free energy determined by using the SMD[8] continuum solvation model.

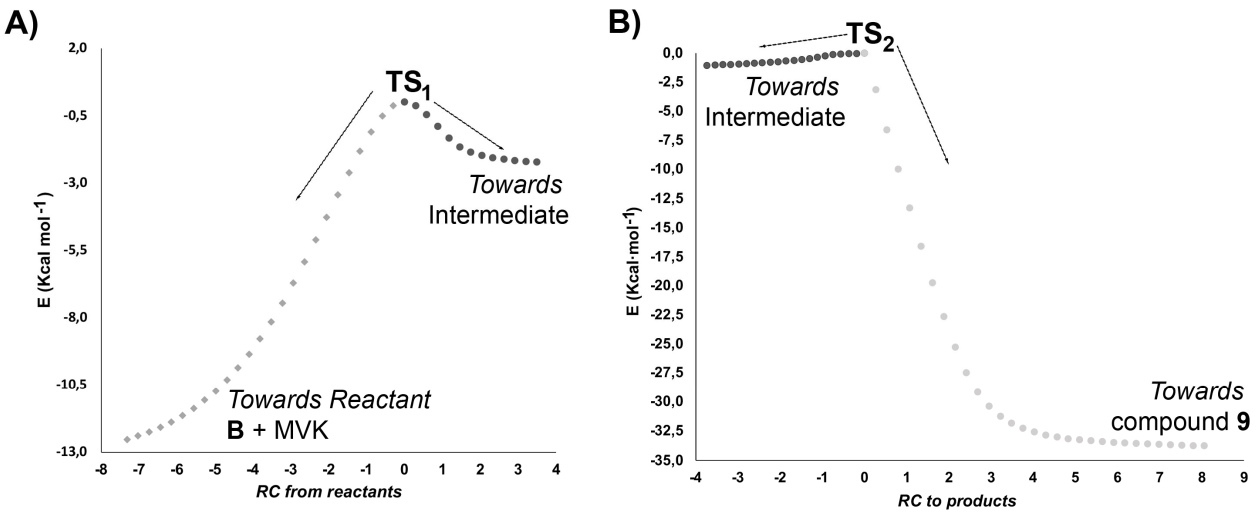

Figure S3. The intrinsic reaction coordinate (IRC) energy profile calculated from transition states A) TS_1 and B) TS_2 have been calculated to confirm that from both TS connect with the corresponding reactants and products of the two main steps.

Coordinates (Å) of stationary points along the reaction mechanism

Reactant

C	0.758141085135	-1.970143753002	1.538666404199
C	1.170768935252	-0.601738807979	0.986219670385
C	-0.236194998298	1.267313246741	0.138232830051
H	0.699520755941	-2.722931945534	0.739214842328
H	1.522529827821	-2.304640723563	2.249444201670
H	1.176079538912	0.049634723087	1.861467180317
O	-0.459855940455	-1.874056510080	2.233036206133
C	2.569600623244	-0.551763910326	0.381946528615
C	3.067305576930	0.683039661822	-0.053459313195
C	3.370463743931	-1.683215052547	0.223681613441
C	4.333157093816	0.785384174997	-0.616572346805
H	2.440669499620	1.568619197235	0.035328277317
C	4.643709450040	-1.583449766589	-0.337987076783
H	3.009036554759	-2.657441391646	0.538629147098
C	5.129847069598	-0.350892778877	-0.757471144031
H	4.701090305972	1.752313919890	-0.946899457352
H	5.252916717662	-2.475587112246	-0.448888108165
H	6.120358575298	-0.273024594395	-1.194915121108
N	0.142744736979	-0.091243352272	0.067364153112
C	-0.210436983028	1.881411812575	1.526088338169
H	0.823025488732	2.120775836985	1.818025983537
H	-0.563757175109	1.131317112318	2.245639200320
C	-0.703737342473	1.953633171050	-0.926635979742
C	-1.144236076937	3.394760288284	-0.844191508967
H	-0.729874314803	3.939412031542	-1.704483197377
H	-2.238722582973	3.448624360549	-0.963611579636
C	-0.886126099457	1.302084254998	-2.275019462212
H	-1.807188412302	1.684670745414	-2.735590686733
H	-0.069467350578	1.582606738403	-2.956637561969
C	-3.186730575845	-0.126840474762	0.913964920464
H	-3.642692968431	0.787276200708	1.281557930867
C	-3.258606077803	-2.296514815809	-0.232807149707
O	-2.116427993366	-2.609176196174	0.060439024150
C	-3.906348302805	-1.063449983933	0.293117437529
C	-4.076761556752	-3.138320088911	-1.181233306930
H	-4.970781881603	-0.941432895184	0.109373060584
H	-5.013404490289	-3.442098457056	-0.701647401981
H	-3.509076328819	-4.018329599231	-1.483268263849
H	-4.342878509220	-2.545326300599	-2.063616876200
H	-2.115515618245	-0.256961071986	1.057190328759
C	-1.055507684059	3.148895520249	1.624907025570
H	-0.873316030927	3.638660392001	2.586958976243
H	-2.121306345106	2.884817181963	1.593981371844
C	-0.739877180598	4.077429214258	0.457886817035
H	-1.254548787422	5.038062359882	0.563764550471
H	0.338277799499	4.285551277827	0.447141331642
C	0.196789439645	-0.690654790197	-1.263141334068
H	0.160587258072	-1.778923135504	-1.147912167943
H	1.145207228020	-0.439476075625	-1.765287583031
C	-0.959406072594	-0.213132922234	-2.126934810156
H	-1.904916422256	-0.484180675759	-1.646055126038
H	-0.919356712304	-0.714755545106	-3.099192611259
H	-1.157820490018	-2.017183695650	1.569997823381

E(RM062X) = -1022.41023590 a.u.

Transition State 1

C	-0.899374329	-1.4251977693	-1.554747151
C	-1.4588863435	-0.1494471701	-0.90674387
C	0.483906526	1.1542645223	-0.1039780577
H	-0.78030781	-2.2156696162	-0.7980454757
H	-1.6290370879	-1.7768318793	-2.2973658586

H	-1.4725056805	0.6079924354	-1.6916087001
O	0.3170133292	-1.1389298904	-2.1777805391
C	-2.8666116999	-0.244592591	-0.3457273707
C	-3.4565780131	0.9088366749	0.1812176644
C	-3.5933798634	-1.4348703202	-0.3369822072
C	-4.7445412331	0.877342878	0.7008937875
H	-2.8891138084	1.837679176	0.18580128
C	-4.8880340651	-1.4688986211	0.1810089494
H	-3.1562539006	-2.3466345585	-0.7322661823
C	-5.4660376104	-0.3160286727	0.6999330228
H	-5.1879164997	1.7821687543	1.1050382047
H	-5.4417011334	-2.4026079688	0.1795632427
H	-6.4732032246	-0.3443540359	1.1036868278
N	-0.5166117176	0.3044594242	0.1365385425
C	0.4338698337	1.9729515984	-1.3770659522
H	-0.5447372274	2.4707630205	-1.4136927615
H	0.475928776	1.2816358835	-2.2265664008
C	1.6318035363	1.254175708	0.74175564
C	2.270297012	2.6410864198	0.8897736722
H	2.0781736108	3.0018229031	1.9078999403
H	3.3609475817	2.5470798161	0.8093592313
C	1.6031504687	0.4956005699	2.0542129988
H	2.6305167481	0.2356022058	2.332947409
H	1.2077508805	1.1553317763	2.8397320672
C	2.8519662604	0.260386386	-0.4393795083
H	3.4867507754	1.1196064058	-0.6442320123
C	3.0369904407	-2.1607588064	-0.0290727055
O	1.9805859097	-2.4804217564	-0.6409124651
C	3.4920214407	-0.8447962072	0.174582695
C	3.8550010134	-3.3071590154	0.5380344987
H	4.3811395716	-0.6857607163	0.7758357457
H	4.7642608882	-2.9738051789	1.0429501983
H	4.1207947484	-3.9891324313	-0.2750072037
H	3.2375370574	-3.8741651674	1.242348896
H	2.218166235	0.0036895832	-1.2857183491
C	1.5364372914	3.0301338409	-1.4763864341
H	1.2412032545	3.77360324	-2.2231210135
H	2.4660721099	2.578422068	-1.8361854237
C	1.759937627	3.6773240805	-0.1151140377
H	2.4617555952	4.5140012399	-0.1851796112
H	0.8051586953	4.0910872521	0.2366959908
C	-0.6196612515	-0.3958583174	1.4288941557
H	-1.2291769288	-1.2877001044	1.2719080867
H	-1.1682928511	0.2532227186	2.1232527168
C	0.7486779291	-0.7603170237	1.9710496621
H	1.1973092679	-1.4948991804	1.3007872446
H	0.635016408	-1.2194365902	2.9583501121
H	1.0290724566	-1.6130999925	-1.6651621921

E(RM062X) = -1022.38700451 a.u.

Intermediate

C	3.6272935077	2.6100869899	-0.8238514924
C	2.6112567221	2.9327805284	0.2931593259
C	0.4910108578	2.0641183321	-0.5627739949
H	3.6773045135	3.4444634914	-1.5400298466
H	4.6116797939	2.5147652808	-0.3432607255
H	2.5624454369	2.0521842862	0.9329539913
O	3.2733867634	1.4302616522	-1.4632759784
C	2.9200922485	4.124704589	1.1763234271
C	2.1116354606	4.3639337392	2.2919867287
C	3.9890600563	4.9835432959	0.9211818853
C	2.3616357351	5.4400708695	3.1347731693
H	1.2760577927	3.6963288907	2.495542356
C	4.2465027347	6.0599266624	1.7692829145
H	4.628814648	4.8187336739	0.0596089718
C	3.4346685713	6.2913857642	2.8743064174
H	1.7247317608	5.6129658751	3.9966448672
H	5.0835308057	6.7187312783	1.560861928

H	3.6356944907	7.1307480016	3.5324450111
N	1.284226746	3.0823954946	-0.3619534282
C	0.7511531934	0.7637439331	0.1717799271
H	0.8200571104	1.0075049509	1.2410753201
H	1.7297869746	0.3895225431	-0.1439883902
C	-0.6136007987	2.0842337027	-1.5627087422
C	-1.9113330574	1.421721406	-1.0244771744
H	-2.584968206	2.2032662691	-0.6577020629
H	-2.4136193192	0.9663997317	-1.8849326114
C	-0.9506708175	3.4837741376	-2.0845874181
H	-1.4696970372	3.3765923382	-3.0412519321
H	-1.6395474993	3.9686231496	-1.3793461731
C	0.0336304059	1.1323661753	-2.7587463415
H	-0.6621936026	0.2909012445	-2.8320038794
C	1.4709134744	2.2695674753	-4.435675129
O	2.4653974715	2.448538185	-3.633228706
C	0.250923035	1.7434074669	-4.0895626419
C	1.6976376176	2.756978455	-5.8544782296
H	-0.5442553238	1.6902245885	-4.8256736069
H	0.8290204609	2.6050773627	-6.5003048404
H	2.5584490977	2.2313499941	-6.2799586653
H	1.9528774912	3.8223579114	-5.8384658416
H	0.9854327684	0.7297846615	-2.3972826909
C	-0.3445054995	-0.286292819	-0.0184011772
H	-0.2234011097	-1.0522230765	0.7536166944
H	-0.2308527193	-0.7891378527	-0.9822499988
C	-1.7114679708	0.3788547107	0.0855843111
H	-2.5148488527	-0.362906652	0.0542782342
H	-1.7782310879	0.869803331	1.0660043722
C	1.0313179689	4.425923335	-0.936097061
H	2.0040602219	4.9022638556	-1.066052325
H	0.4870104745	4.9949203425	-0.173188596
C	0.283208113	4.3552094892	-2.248654862
H	0.9579906996	3.9476994717	-2.9998884165
H	0.0014300405	5.3714209674	-2.5424458325
H	2.9078306364	1.7024705195	-2.3799160406

E(RM062X) = -1022.39065873 a.u.

Transition state 2

C	0.5611827707	-0.4022035433	-1.8841051271
C	0.8407182506	-1.2639082141	-0.6031481354
C	-1.3879370156	-0.6132350471	-0.1638896163
H	1.2548599714	0.4501043239	-1.8583467686
H	0.7986336879	-1.0076569316	-2.7704984675
H	0.6455002926	-2.3273667656	-0.814011782
O	-0.7622258326	0.0440851669	-1.927837253
C	2.2760273254	-1.129790135	-0.1550946058
C	3.1866901757	-2.1630213445	-0.363284739
C	2.7196426863	0.0644432997	0.4239214063
C	4.5262438693	-2.0086079543	-0.0084584293
H	2.8466810489	-3.0946822223	-0.8094546411
C	4.0528175379	0.2168527253	0.7857132497
H	1.996726965	0.8620048122	0.5855558612
C	4.9612493537	-0.8193863669	0.5671473928
H	5.2271525583	-2.8199725808	-0.1793215482
H	4.3861599465	1.1477203052	1.2353741874
H	6.0034091706	-0.6986098522	0.8463368358
N	-0.0948575853	-0.7099178526	0.3589564973
C	-2.0288048583	-1.8959952265	-0.6578513943
H	-2.1020124907	-2.5505004708	0.2294453259
H	-1.3613783004	-2.3921632928	-1.3628616298
C	-2.2617139522	0.4205755293	0.5182871055
C	-3.7209357575	0.3963840461	-0.0000563372
H	-4.3380255715	0.9579467772	0.7127501041
H	-3.754650614	0.950956805	-0.9456757566
C	-2.2544554559	0.0333657238	2.0226716825
H	-2.8078032997	0.8049991497	2.571532655
H	-2.8134143025	-0.9045076901	2.150783513

C	-1.7806394741	1.8896191934	0.2958707464
C	0.4017304135	2.7190524073	-0.5822555821
C	-0.36002193	2.3435071659	0.4595743091
C	1.7350698149	3.3930074429	-0.4472725352
H	-2.0837206092	2.1582917619	-0.7210857298
H	-2.4150949305	2.4899730136	0.9655940748
H	1.7154245807	4.354238936	-0.9714749369
H	2.516667803	2.783986691	-0.9160734322
H	1.9939497158	3.5663717004	0.6002132453
O	0.0440607115	2.5602102932	-1.878999301
C	-3.416923977	-1.7197949079	-1.2576740825
H	-3.830555964	-2.6990164376	-1.5193826707
H	-3.3271542012	-1.1381577563	-2.1826535475
C	-4.310894886	-0.984942247	-0.2654306419
H	-5.3263330905	-0.8747690108	-0.6601280193
H	-4.3953936696	-1.5687007612	0.6612792214
C	-0.0313083817	-1.1131031009	1.7634473091
H	1.0166203095	-1.1035272273	2.0708580663
H	-0.4029022098	-2.1465303278	1.8935211548
C	-0.8531733747	-0.1460723686	2.6068042684
H	-0.3327417602	0.813586482	2.6478281567
H	-0.9222401339	-0.5275035261	3.6312488774
H	-0.4727150869	1.7131539233	-1.9626023498
H	0.029900706	2.5342337269	1.4548327239

E(RM062X) = -1022.39466190 a.u.

Product

C	0.4449334309	0.452985949	-1.5965147961
C	1.2408425863	-0.5836840154	-0.7982139742
C	-0.9994594228	-0.961757875	-0.4099760044
H	0.5527776475	1.458403533	-1.1754608334
H	0.712306961	0.4778576822	-2.6569354505
H	1.3791702767	-1.4653875031	-1.4346693181
O	-0.914299114	0.0398183779	-1.4625738725
C	2.6188033624	-0.1914544792	-0.3170872035
C	3.4042858858	-1.1795605788	0.2884192437
C	3.1476007643	1.0900769592	-0.4600368967
C	4.6813973994	-0.8914241025	0.7509185303
H	2.9897533922	-2.1785568474	0.4031817749
C	4.4345783116	1.3799511547	-0.0051575136
H	2.5669176258	1.8757737721	-0.9331320701
C	5.2023097831	0.3943377711	0.6031155605
H	5.2747432112	-1.6688357038	1.2225481197
H	4.8334483893	2.3821455063	-0.1286075734
H	6.2024594313	0.622333666	0.9582476202
N	0.3010900368	-0.9650362885	0.2741852923
C	-1.1036600172	-2.3555064553	-1.0823662272
H	-0.8217012196	-3.0953025132	-0.3244165959
H	-0.3467089161	-2.402290825	-1.8697420511
C	-2.2087066712	-0.6345919736	0.5209810606
C	-3.5376582812	-1.2514722426	-0.0004752618
H	-4.279894625	-1.1502986735	0.8014926989
H	-3.9005376057	-0.6337616557	-0.8323448
C	-1.8648245219	-1.2400987319	1.8892623877
H	-2.7324472013	-1.1484151557	2.5540222764
H	-1.6800386373	-2.3136944594	1.7619291156
C	-2.5773330981	0.883330647	0.6406868626
C	-1.2185410585	2.8689810192	-0.0485149592
C	-1.6090523164	2.0035051081	0.8977484874
C	-0.4053741297	4.1000081009	0.207407316
H	-3.1189257048	1.1446929652	-0.2753212622
H	-3.3362379588	0.915628757	1.4360341388
H	-0.9387427793	4.9815424312	-0.1632393494
H	0.5457523158	4.0482615771	-0.3347369893
H	-0.204326338	4.2280873017	1.2724502767
O	-1.5194198245	2.7363995212	-1.3709480228
C	-2.4654002154	-2.7295214352	-1.6468595662
H	-2.4125934926	-3.7242939475	-2.1021281547

H	-2.755502486	-2.0243479761	-2.4367334744
C	-3.482491412	-2.6824947548	-0.5156555482
H	-4.4788507688	-2.9837785931	-0.8569466423
H	-3.1899568127	-3.3838880603	0.2770605856
C	0.4393120049	-0.1865283377	1.5094111692
H	0.4145914564	0.8933829397	1.3218574467
H	1.4276571124	-0.4122856511	1.9167309602
C	-0.6365325814	-0.5871742587	2.5345668065
H	-0.9404385645	0.3058321975	3.0909287422
H	-0.2126845218	-1.2790527288	3.2685596406
H	-1.6908701974	1.7903813018	-1.5450584835
H	-1.3047908907	2.2156905839	1.9181177814

E(RM062X) = -1022.42787006 a.u.

V) References

[1] Piccichè, M.; Pinto, A.; Griera, R.; Bosch, J.; Amat, M. *Org. Lett.* **2022**, *24*, 5356−5360.

[2] Pham, V. C.; Jossang, A.; Grellier, P.; Sévenet, T.; Nguyen, V. H.; Bodo, B. *J. Org. Chem.* **2008**, *73*, 7565–7573.

[3] Pham, V. C.; Jossang, A.; Chiaroni, A.; Sévenet, T.; Bodo, B. *Tetrahedron Lett.* **2002**, *43*, 7565-7568.

[4] Zhao, Y.; Truhlar, D. G. *Theor. Chem. Acc.* **2008**, *120*, 215–241.

[5] Hehre, W. J.; Ditchfield, R.; Pople, J. A. *J. Chem. Phys.* **1972**, *56*, 2257–2261.

[6] Hariharan, P. C.; Pople, J. A. *Theor. Chim. Acta* **1973**, *28*, 213–222.

[7] Frisch, M. J.; Trucks, G. W.; Schlegel, H. B.; Scuseria, G. E.; Robb, M. A.; Cheeseman, J. R.; Scalmani, G.; Barone, V.; Petersson, G. A.; Nakatsuji, H.; Li, X.; Caricato, M.; Marenich, A. V.; Bloino, J.; Janesko, B. G.; Gomperts, R.; Mennucci, B.; Hratchian, H. P.; Ortiz, J. V.; Izmaylov, A. F.; Sonnenberg, J. L.; Williams-Young, D.; Ding, F.; Lipparini, F.; Egidi, F.; Goings, J.; Peng, B.; Petrone, A.; Henderson, T.; Ranasinghe, D.; Zakrzewski, V. G.; Gao, J.; Rega, N.; Zheng, G.; Liang, W.; Hada, M.; Ehara, M.; Toyota, K.; Fukuda, R.; Hasegawa, J.; Ishida, M.; Nakajima, T.; Honda, Y.; Kitao, O.; Nakai, H.; Vreven, T.; Throssell, K.; Montgomery, J A.; Peralta, J. E.; Ogliaro, F.; Bearpark, M. J.; Heyd, J. J.; Brothers, E. N.; Kudin, K. N.; Staroverov, V. N.; Keith, T. A.; Kobayashi, R. Jr.; Normand, J.; Raghavachari, K.; Rendell, A. P.; Burant, J. C.; Iyengar, S. S.; Tomasi, J.; Cossi, M.; Millam, M.; Klene, M.; Adamo, C.; Cammi, R.; Ochterski, J. W.; Martin, R. L.; Morokuma, K.; Farkas, O.; Foresman, J. B.; Fox, D. J. Gaussian, Inc., Wallingford CT, 2016.

[8] Marenich, A. V.; Cramer, C. J.; Truhlar, D. G. *J. Phys. Chem. B* **2009**, *113*, 6378–6396.
